# Association between dietary habits and the risk of migraine: a Mendelian randomization study

**DOI:** 10.3389/fnut.2023.1123657

**Published:** 2023-06-07

**Authors:** Xinhui Liu, Yuanyuan Yu, Lei Hou, Yifan Yu, Yutong Wu, Sijia Wu, Yina He, Yilei Ge, Yun Wei, Qingxin Luo, Fengtong Qian, Yue Feng, Hongkai Li, Fuzhong Xue

**Affiliations:** ^1^Department of Biostatistics, School of Public Health, Cheeloo College of Medicine, Shandong University, Jinan, Shandong, China; ^2^Institute for Medical Dataology, Cheeloo College of Medicine, Shandong University, Jinan, Shandong, China

**Keywords:** migraine, dietary habit, insomnia, major depression disorder, Mendelian randomization, mediator

## Abstract

**Objective:**

The important contribution of dietary triggers to migraine pathogenesis has been recognized. However, the potential causal roles of many dietary habits on the risk of migraine in the whole population are still under debate. The objective of this study was to determine the potential causal association between dietary habits and the risk of migraine (and its subtypes) development, as well as the possible mediator roles of migraine risk factors.

**Methods:**

Based on summary statistics from large-scale genome-wide association studies, we conducted two-sample Mendelian randomization (MR) and bidirectional MR to investigate the potential causal associations between 83 dietary habits and migraine and its subtypes, and network MR was performed to explore the possible mediator roles of 8 migraine risk factors.

**Results:**

After correcting for multiple testing, we found evidence for associations of genetically predicted coffee, cheese, oily fish, alcohol (red wine), raw vegetables, muesli, and wholemeal/wholegrain bread intake with decreased risk of migraine, those odds ratios ranged from 0.78 (95% CI: 0.63–0.95) for overall cheese intake to 0.61 (95% CI: 0.47–0.80) for drinks usually with meals among current drinkers (yes + it varies vs. no); while white bread, cornflakes/frosties, and poultry intake were positively associated with the risk of migraine. Additionally, genetic liability to white bread, wholemeal/wholegrain bread, muesli, alcohol (red wine), cheese, and oily fish intake were associated with a higher risk of insomnia and (or) major depression disorder (MDD), each of them may act as a mediator in the pathway from several dietary habits to migraine. Finally, we found evidence of a negative association between genetically predicted migraine and drinking types, and positive association between migraine and cups of tea per day.

**Significance:**

Our study provides evidence about association between dietary habits and the risk of migraine and demonstrates that some associations are partly mediated through one or both insomnia and MDD. These results provide new insights for further nutritional interventions for migraine prevention.

## 1. Introduction

Migraine, mainly including migraine with aura (MA) and migraine without aura (MO) (1), is the second most common neurological disorder worldwide (2) with the overall prevalence of around 15–20% (3), contributing 45.1 million years lived with disability and 5.6% of the global disease burden (4, 5). Identification of modifiable risk factors that are causally related to migraine could contribute to the understanding of its pathogenesis and suggest directions for migraine prevention. As important elements of modifiable lifestyle, dietary habits have been shown as important triggers for migraine in abundant epidemiological observational studies (6, 7). However, most studies proposed potential dietary triggers for migraine attacks among patients with migraine, those potential causal factors for migraine development in the whole population were less studied. In addition, controversy and uncertainty widely exist in this field (6, 8), since the results obtained from observational studies are potentially biased by residual confounding and reverse causality.

Mendelian randomization (MR) using genetic variants associated with a risk factor as an instrumental variable can provide causal evidence for the association of risk factor on the health outcome. While several MR studies have provided a protective role of some dietary habits, including alcohol and coffee consumption (9), on risk of migraine, uncertainty persists about the potential causal role of other dietary habits for the development of migraine, such as the intake of different bread types and milk, etc. In addition, migraine might also influence the intake of dietary habits (8), this emphasized a potential bidirectional relationship between diet and migraine. To the best of our knowledge, a systematic and comprehensive collection of association between diet and migraine has not been established yet.

Moreover, the potential pathways involved in the association from dietary habits to migraine have not been studied. Existing evidence from MR studies suggests the potential causal association between several risk factors [including systolic blood pressure (SBP) (10), diastolic blood pressure (DBP) (10), serum total calcium (11), neuroticism (10), difficulty awakening (12), insomnia (12)], anxiety (13) and migraine, as well as major depression disorder (MDD) on headache (14). In addition, previous MR studies presented evidence that several dietary habits are associated with these risk factors for migraine, e.g., alcohol intake and blood pressure (15). Consequently, one or more of these migraine risk factors may act as potential mediators between specific dietary habits and migraine.

In this study, based on summary data obtained from large consortium, we performed a series of two-sample MR analyses to explore whether each dietary habit is a risk factor for the development of migraine and its subtypes (MA and MO) without prior hypotheses. We conducted a mediation analysis to examine possible mediators in the relationship between each genetically determined dietary habits and risk of migraine using a network MR design. Bidirectional MR was also performed to investigate if migraine is associated with dietary habits. The elucidation of these associations could provide evidence for the prevention of migraine and the development of helpful diet strategies.

## 2. Materials and methods

**FIGURE 1:**
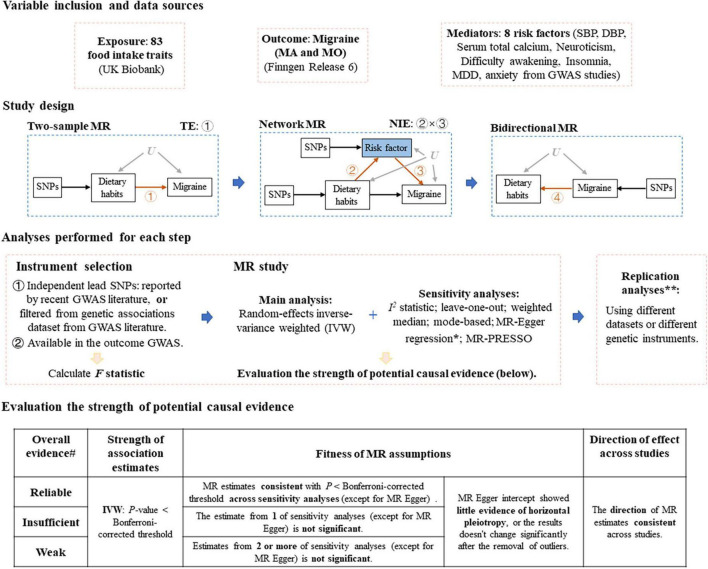
Study design of Mendelian randomization study of migraine. MR, Mendelian randomization; MA, migraine with aura; MO, migraine without aura; TE, total effect, NIE, natural indirect effect; SBP, systolic blood pressure; DBP, diastolic blood pressure; MDD, major depression disorder; GWAS, genome-wide association study. *If MR Egger intercept shows evidence of horizontal pleiotropy (*P* < 0.05), we removed single nucleotide polymorphisms (SNPs) that are most likely to display horizontal pleiotropic effects (outliers) and reran the IVW and leave-one-out analyses. **Replication analyses were only performed for several steps. ^#^If there presents *P* > 0.05 in the leave-one-out sensitivity analysis (after the removal of SNPs that are most likely to display horizontal pleiotropic effects), the evidence strength drops by one level.

A flow chart of the whole study design was provided in Figure 1.

### 2.1. Data sources

Genetic association estimates for 83 dietary habits (classified into 16 subtypes, see Supplementary Table 1) were obtained from a recent large-scale genome-wide association study (GWAS) of dietary habits in up to 449,210 Europeans from UK Biobank (16) using the imputed genetic data. Those ordinal variables were ranked and set to quantitative values, while food types or foods never eaten were converted into a series of binary variables. The genetic-association data for migraine (all subtypes) and migraine subtypes (MA and MO) were collected from the FinnGen release 6 (R6) (17) with imputed genotype data, including up to 260,405 individuals of Finnish descent. To be specific, participants with migraine were diagnosed with the International Classification of Diseases criteria: code 346 for the eighth and ninth version (ICD 9), and code G43 for the tenth version (ICD 10), and those with MA and MO were diagnosed with ICD 9: 3,460 and 3,461, or ICD 10: G431 and G430, respectively. The controls excluded participants with epilepsy (ICD 10: G40), status epilepticus (ICD 10: G41), migraine, other headache syndromes (ICD 10: G44), sleep apnoea (ICD 10: G473), narcolepsy and cataplexy (ICD 10: G474), and other sleep disorders (ICD 10: G47).

In addition, we identified 8 risk factors for migraine reported by previous MR study (10–13) to investigate their potential mediator role in the pathway from each dietary habits to migraine. Genetic association estimates for SBP, DBP, serum total calcium, neuroticism, difficulty awakening, insomnia, MDD and anxiety were taken from the International Consortium of Blood Pressure (ICBP) (18), published GWAS studies in Young et al. (19), Nagel et al. (20), Neale Lab Consortium, GWAS studies in Jansen et al. (21) and Howard et al. (22), and the Integrative Psychiatric Research (iPSYCH) consortia (23) from European ancestry. Population characteristics of these consortium and definitions relating to these genetic association estimates were presented in the Table 1 and Supplementary Table 2.

**TABLE 1:** Description of GWAS consortiums for each phenotype.

Variable	Consortium	Sample size (case/control)	Units	Population	References
**Main and sensitivity analyses**					
Dietary habits	–	449,210	–	European	(16)
Migraine	FinnGen release 6	260,405	–	European	(17)
Systolic blood pressure	International Consortium of Blood Pressure (ICBP)	458,575	SD = 20.7 mm Hg	European	(18)
Diastolic blood pressure		458,577	SD = 11.3 mm Hg		
Serum total calcium	–	305,349	mmol/L	European	(19)
Neuroticism	–	449,484	–	European	(20)
Difficulty awakening	Neale lab	451,872	–	European	–
Insomnia	–	1,331,010 (397,959/933,051)	–	European	(21)
Major depression disorder	–	807,553 (246,363/561,190)	–	European	(22)
Anxiety	Integrative Psychiatric Research (iPSYCH)	23,809 (4,584/19,225)	–	European	(23)
**Replication analyses**					
Alcohol consumption	GWAS and Sequencing Consortium of Alcohol and Nicotine use (GSCAN)	941,280	SD of log-transformed weekly alcohol drinks consumed	European	(41)
Cheese intake	MRC IEU	451,486	SD	European	–
Average weekly red wine intake	Neale lab	257,773	Glasses	European	–
Major depression disorder	–	480,359 (135,458/344,901)	–	European	(43)
Food phenotypes as well as their PCs	–	445,779	–	European	(44)

SD, standard deviation.

### 2.2. Construction of genetic instruments

To select instrumental variables for each dietary habit, the genetic association estimates from GWAS of dietary habits (16) were used as our starting point. We first extracted all genome-wide significant variants associated with each dietary habit (the significance threshold was relaxed to *P* < 5 × 10^–6^ to improve instrument power). We then filtered this dataset using a clumping distance of 1 Mb and *r*^2^ threshold of 0.001 to generate an independent set of variants for each exposure (using *ld_clump* function in the *ieugwasr* R package with linkage disequilibrium estimates from the 1,000 Genomes European reference panel integrated within this R package). The number and *F* statistics (the instruments’ strength) of selected variants for each dietary habit were provided in Supplementary Table 1.

Then we selected instruments for mediation analysis and bidirectional MR. The instrumental variables for migraine (MA and MO) were obtained from a GWAS meta-analysis of 102,084 migraine (14,624 MA and 15,055 MO) cases and 771,257 controls of European ancestry (24). This study identified 123 independent lead variants (*r*^2^ < 0.1, distinct genomic loci are > 250 kb apart) associated with migraine (as well as MA and MO) at genome-wide significance (*P* < 5 × 10^–8^) (24). To select instrumental variables for potential mediator, we utilized 130, 91, 208, 170, 248, 102 independent single nucleotide polymorphisms (SNPs) that were associated with SBP, DBP, serum total calcium, neuroticism, insomnia and depression, respectively, at genome-wide significance (*P* < 5 × 10^–8^) clumped by previous GWAS analysis (18–22). We selected instrumental variables for difficulty awakening using the same way as that for dietary habits. For each MR analysis, we restricted the instrument list to SNPs also available in the outcome GWAS and harmonized the exposure and outcome datasets by matching alleles.

### 2.3. Study design

#### 2.3.1. The associations between 83 dietary habits and migraine and the potential mediator role of 8 risk factors

We first performed a series of two-sample MR analyses to explore the associations between each genetically predicted dietary habits and migraine. For each dietary habit that is significantly associated with migraine, we then conducted mediation analysis to explore whether each risk factor mediate the pathway from dietary habits to migraine, distinguished the effects of each mediator and disentangled the contribution of each mediator to the indirect effect (25), using a network MR design (26). To be specific, based on the results in first step, we carried out a second MR model to estimate the association between each dietary habits that is significantly associated with migraine and each risk factor (potential mediator). For possible mediators that potential causal association was observed in the second MR model, we estimated the effect of each mediator on migraine, respectively, controlling for the exposure using multivariable MR (MVMR) (27, 28), the genetic instruments both mediator and exposure were included as instruments in MVMR analysis. To be noted, we pruned those SNPs again to ensure the instruments used in MVMR are mutually independent. If associations were observed in all three steps, the conclusion can be drawn that the specific risk factor is a mediator in the pathway linking this dietary habit to migraine. The indirect effect mediated through each mediator was estimated by multiplying the results from two steps behind. We divided the indirect effect by the total effect to estimate the proportion mediated by each mediator [the calculation of their 95% confidence intervals was calculated through the Delta method, which was provided in our previous work (29)].

#### 2.3.2. Bidirectional causality between dietary habits and migraine as well as dietary habits and risk factors

To examine whether migraine or each potential mediator affects dietary habits (bidirectional causality), we performed a series of two-sample MR analyses by using outcome or mediator-associated independent SNPs as instrumental variables.

We performed these analyses on migraine (all subtypes), MA and MO, respectively.

### 2.4. Statistical analysis

For each step of this MR study design, random-effects inverse-variance weighted (IVW) MR (30) was used as our main analysis, this method accounts for potential heterogeneity in the variant-specific causal estimates. For analyses with binary outcome (e.g., migraine), the results were converted to ORs expressed per genetically predicted 1-unit-higher log-odds/per unit change/per standard deviation (SD) of liability to the exposure; For those with continuous outcome (e.g., SBP), the results were SD/per unit change in outcome expressed per genetically predicted 1-unit-higher log-odds/per unit change/per SD of liability to the exposure.

Mendelian randomization (MR) analysis relies on three core assumptions: (1) Relevance: genetic variants are associated with the exposure; (2) Independence: genetic variants are not associated with any confounder of the exposure-outcome association; (3) Exclusion restriction: genetic variants do not affect outcome except through its potential effect on the exposure. In this study, a series of sensitivity analyses were conducted to verify these core assumptions and test the robustness of the causal findings. Firstly, since causal conclusions are less reliable when there is substantial heterogeneity, we evaluated the magnitude of heterogeneity among variant-specific Wald estimates using the *I*^2^ statistic (31, 32). Leave-one-out sensitivity analysis was also performed to assess the reliance of the MR results on each particular variant. Secondly, we calculated *F* statistic to ensure that the relevance assumption is satisfied. Thirdly, we performed a range of robust methods making different consistency assumptions, including weighted median (33), mode-based (34), MR-Egger regression (35), and MR pleiotropy residual sum and outlier (MR-PRESSO) method (36), the combination of which is reported to adequately assess the evidence of the causal effects of each exposure on the outcome and detect the sensitivity of the results to different patterns of violations of instrumental variable assumptions (37). To be noted, mode-based and MR-Egger regression have been shown to have low precision (37, 38). We used MR Egger intercept test (35) to detect the possibility of presence of horizontal pleiotropic effects and therefore provide evidence of certain violations of the exclusion restriction. If MR Egger intercept test show evidence of horizontal pleiotropy (*P* < 0.05), we removed of SNPs that are most likely to display horizontal pleiotropic effects (outliers) and rerun the IVW and leave-one-out analyses.

Within each subtype of dietary habits, to account for multiple testing and to preserve the type I error of the global null hypothesis of all tested associations being in fact null for this outcome (39), we used Bonferroni-corrected threshold α = 0.05/(number of dietary habits in this subtype *n*). We noted that these traits are related to each other, therefore the tests are not completely independent of each other and the Bonferroni correction may be conservative. We reported the actual *P*-value of each effect.

Rather than using *P*-value as the only criteria, we evaluated the strength of detected potential causal evidence of MR analyses using three criteria similar as Zheng et al. (40) did: (1) strength of association estimates: whether the IVW estimate passes the Bonferroni-corrected threshold; (2) fitness of MR assumptions: whether the MR estimates are consistent across MR sensitivity analyses after the removal of SNPs that are most likely to display horizontal pleiotropic effects; (3) Direction of effect across studies: whether the MR estimates show the same direction of effect across MR sensitivity analyses. According to these criteria, we graded the strength of potential causal evidence into three levels: reliable, insufficient and weak, see Figure 1 for the details.

Finally, we conducted replication analyses by using different datasets to further assess the robustness of our results. For the association between genetically predicted dietary habits and migraine as well as 8 risk factors, we used a second summary statistics data for alcohol consumption (log-transformed average number of drinks consumed each week) obtained from the largest, predominantly white European ancestry meta-analyzed GWAS, which is conducted by the GWAS and Sequencing Consortium of Alcohol and Nicotine use (GSCAN) (41). We pruned 99 conditionally independent SNPs associated with alcohol consumption (*P* < 5 × 10^–8^) identified by GSCAN with pairwise linkage disequilibrium (LD) *R*^2^ > 0.001, leaving 73 to 84 SNPs that were available in the outcome datasets. We note that the definition of alcohol consumption in this second dataset is different from those drinking habits used in our primary analysis (Supplementary Table 2). We also validated the results for cheese intake and red wine intake with a second summary statistics data obtained from the UK Biobank study (Table 1 and Supplementary Table 2), utilizing 61 to 65 independent SNPs (*r*^2^ < 0.01, distinct genomic loci are > 10,000 kb apart) associated with cheese intake at genome-wide significance (*P* < 5 × 10^–8^) selected by Hu et al. (42) and 64 to 73 independent SNPs selected using the same way as our primary analysis, respectively. These results were OR/SD or per unit change in outcome expressed per genetically predicted 1 SD increase in log-transformed weekly alcohol drinks consumed, 1 SD increase in cheese intake or per unit increase in average weekly red wine intake. In addition, we used a second MDD dataset obtained from another large GWAS meta-analysis of 480,359 individuals of European ancestry (Table 1 and Supplementary Table 2) (43) for comparison of the primary 83 dietary habits and MDD analysis.

In the main analysis for dietary habits on migraine, we relaxed the significance threshold to improve the instrument power. Therefore, we further performed replication analyses using 814 LD-independent loci (> 500 kb apart) surpassing genome-wide significance (*P* < 5 × 10^–8^) for 83 dietary habits as well as 60 principal components (PCs) that capture correlation structure among intake of single foods to take account for those complex intercorrelation among dietary intakes (16). We also used the association between the genetic variants and 29 food phenotypes as well as their PCs [see Table B in S1 Table of Pirastu et al. (44) for a detailed description] from a recent published GWAS study (44). They identified 572 genetic associations in 283 independent loci at Bonferroni corrected level of significance (*P* < 5 × 10^–8^), among which 245 genetic variants were filtered to have only direct effect [not mediated through possible mediators (e.g., body mass index (BMI)) or confounders (e.g., educational attainment) of food-migraine association] on diet. We performed replication analyses to explore the associations of each genetically predicted dietary habits with migraine using genetic instruments with and without filtering, respectively.

All statistical tests were two-sided. Analyses in this study were performed using R version 4.0.2 together with the R package *MendelianRandomization* (45), *MRPRESSO* (36).

## 3. Results

### 3.1. Association between genetically predicted dietary habits and migraine

**FIGURE 2:**
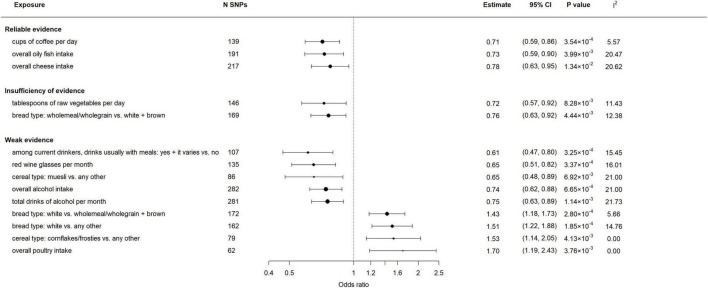
Associations between genetically predicted dietary habits and migraine. Results were obtained from the multiplicative random-effects inverse-variance weighted method. N SNPs denotes the number of instrumental SNPs used for each dietary exposure. *I*^2^ statistic quantifies the amount of heterogeneity among estimates based on individual SNPs.

The association between 83 genetically predicted dietary habits and migraine using the IVW method were displayed in Figure 2 and Supplementary Table 3. Genetically predicted cups of coffee per day (cups/day), overall oily fish and cheese intake were significantly negatively associated with risk of migraine, the ORs ranged from 0.71 (95% CI: 0.59–0.86) per cups/day increase in coffee intake to 0.78 (95% CI: 0.63–0.95) times/week increase in cheese intake. There was an insufficiency of evidence of negative associations between genetically predicted tablespoons of raw vegetables per day (OR = 0.72; 95% CI: 0.57–0.92) as well as wholemeal/wholegrain bread type (OR = 0.76; 95% CI: 0.63–0.92) and migraine. Additionally, we found weak evidences that genetically predicted drinks usually with meals among current drinkers (OR = 0.61; 95% CI: 0.47–0.80), red wine glasses (OR = 0.65; 95% CI: 0.51–0.82) and total drinks of alcohol per month (OR = 0.74; 95% CI: 0.62–0.88) were associated with a decrease in risk of migraine; For dietary habits of cereal type, genetically predicted muesli was negatively associated with migraine (OR = 0.65; 95% CI: 0.48–0.89), while cornflakes/frosties was positively associated with migraine (OR = 1.53; 95% CI: 1.14–2.05); For dietary habits of bread type, genetically predicted white bread was associated with an increase in risk of migraine (OR = 1.43; 95% CI: 1.18–1.73 vs. wholemeal/wholegrain + brown type; OR = 1.51; 95% CI: 1.22–1.88 vs. any other type); Genetically predicted overall poultry intake was positively associated with migraine (OR = 1.70; 95% CI: 1.19–2.43). To be noted, if the MR Egger intercept test of one dietary habit shows evidence of pleiotropic effect, the IVW estimate we reported was obtained after the removal of SNPs that are most likely to display horizontal pleiotropic effects. The consistency of OR estimates from main and sensitivity analyses (Supplementary Table 4) were evaluated by the strength of the potential causal evidence. In addition, there was no evidence of SNP that has a strong influence on the estimations of association (Supplementary Table 5).

When stratified by aura status, genetically predicted cups of tea (including black and green tea) (OR = 0.74; 95% CI: 0.56–0.99) and coffee per day (OR = 0.67; 95% CI: 0.50–0.90) were associated with a decrease in risk of MA, while genetically predicted glasses of water per day (OR = 1.36; 95% CI: 1.06–1.75) was associated with an increase in the risk of MA (Supplementary Figure 1 and Supplementary Tables 3–6, 7). Genetically predicted other alcohol (excluding champagne, white wine, red wine, beer, cider, spirits and fort wine), red wine glasses, total drinks of alcohol per month, overall oily fish and cheese intake, tablespoons of cooked vegetables per day, and wholemeal/wholegrain bread type were negatively associated with MO, the ORs ranged from 0.24 (95% CI: 0.10–0.57) for other alcohol to 0.73 (95% CI: 0.54–0.99) for cheese intake. Genetically predicted white bread (OR = 1.51; 95% CI: 1.12–2.02 vs. wholemeal/wholegrain + brown type; OR = 1.64; 95% CI: 1.19–2.24 vs. any other type) and cornflakes/frosties cereal (OR = 2.02; 95% CI: 1.27–3.20) were positively associated with MO (Supplementary Figure 2 and Supplementary Tables 3, 8, 9).

Finally, no statistically significant associations were observed between milk, fruit, spread type, salt, and other habits (including never eat eggs, dairy, wheat, or sugar) and migraine as well as its subtypes. The direction and magnitude of the estimated effect of alcohol consumption, cheese, weekly red wine, coffee, fish and bread intake on migraine and its subtypes in replication analysis (Supplementary Tables 10–12) were broadly similar to our primary results. However, the protective effect of cheese intake did not reach statistical significance. We cannot rule out the possibility that the significant difference in the number of instrumental SNPs included in the primary and replication analyses (217 vs. 65) leads to the difference in power.

### 3.2. Association between genetically predicted dietary habits and risk factors

The association between genetically predicted dietary habits and 8 risk factors (including SBP, DBP, serum total calcium, neuroticism, difficulty awakening, insomnia, MDD and anxiety) were displayed in Supplementary Figures 3–10 and Supplementary Tables 13–31. Genetically predicted 12 of 14 dietary habits, which are significantly associated with migraine in the first step, were observed to be significantly associated with at least one risk factor. For continuous risk factors including SBP, DBP, and serum total calcium, the results were per SD/per unit increase change in risk factor expressed per genetically predicted 1-unit-higher log-odds/per unit change/per SD of liability to the dietary habits.

For bread types, white bread was consistently positively associated with SBP (1.70; 95% CI: 0.88–2.52 vs. wholemeal/wholegrain + brown type; 2.14; 95% CI: 1.25–3.04 vs. any other type), DBP (1.09; 95% CI: 0.63–1.54 vs. wholemeal/wholegrain + brown type; 1.26; 95% CI: 0.76–1.75 vs. any other type), serum total calcium (0.01; 95% CI: 1.00–1.01), neuroticism (OR = 1.10; 95% CI: 1.04–1.16 vs. wholemeal/wholegrain + brown type; OR = 1.11; 95% CI: 1.04–1.17 vs. any other type) and insomnia (OR = 1.32; 95% CI: 1.21–1.44 vs. wholemeal/wholegrain + brown type; OR = 1.32; 95% CI: 1.20–1.45 vs. any other type), while wholemeal/wholegrain bread (vs. white + brown) was consistently negatively associated with SBP (−1.40; 95% CI: −2.32∼−0.47), DBP (−1.10; 95% CI: −1.60∼−0.60), serum total calcium (−0.01; 95% CI: 0.99–1.00), neuroticism (OR = 0.90; 95% CI: 0.85–0.95) and insomnia (OR = 0.75; 95% CI: 0.69–0.81).

For cereal types, cornflakes/frosties cereal was positively associated with SBP (2.16; 95% CI: −0.84 to 3.47), muesli cereal was negatively associated with neuroticism (OR = 0.85; 95% CI: 0.78–0.93), insomnia (OR = 0.69; 95% CI: 0.61–0.78) and MDD (OR = 0.74; 95% CI: 0.65–0.85). For drinking types, drinks usually with meals among current drinkers (vs. no) and red wine glasses per month were negatively associated with neuroticism (OR = 0.85; 95% CI: 0.79–0.92 and OR = 0.89; 95% CI: 0.83–0.94), insomnia (OR = 0.66; 95% CI: 0.59–0.75 and OR = 0.80; 95% CI: 0.72–0.88), MDD (OR = 0.72; 95% CI: 0.64–0.81 and OR = 0.82; 95% CI: 0.73–0.92), and anxiety (OR = 0.59; 95% CI: 0.45–0.78 and OR = 0.59; 95% CI: 0.42–0.83); total drinks of alcohol per month were negatively associated with difficulty awakening (OR = 0.96; 95% CI: 0.94–0.99); overall alcohol intake were negatively associated with insomnia (OR = 0.88; 95% CI: 0.82–0.95) and MDD (OR = 0.89; 95% CI: 0.83–0.95).

In addition, overall cheese intake was negatively associated with SBP (−1.80; 95% CI: −2.78∼−0.82), DBP (−0.57; 95% CI: −1.14∼−0.01), neuroticism (OR = 0.93; 95% CI: 0.88–0.98), difficulty awakening (OR = 0.96; 95% CI: 0.93–0.99), insomnia (OR = 0.85; 95% CI: 0.78–0.94), MDD (OR = 0.89; 95% CI: 0.82–0.96), and anxiety (OR = 0.55; 95% CI: 0.40–0.75); cups of coffee per day was negatively associated with DBP (−1.30; 95% CI: −2.05∼−0.55); overall oily fish intake was positively associated with DBP (0.75; 95% CI: 0.14–1.36), but negatively associated with insomnia (OR = 0.85; 95% CI: 0.78–0.93). The direction and magnitude of the estimated effect of dietary intakes on MDD (Supplementary Tables 32, 33) as well as cheese and red wine intake on 8 risk factors in replication analysis (Supplementary Tables 10, 11) were accordant with our primary results.

When stratified by aura status, genetically predicted all 3 dietary habits that are significantly associated with MA in the first step, were observed to be significantly associated with at least one risk factor. Except for the negatively association between cups of coffee per day and DBP reported above, cups of tea per day were positively associated with serum total calcium (0.01; 95% CI: 1.00–1.01), but negatively associated with awakening (OR = 0.93; 95% CI: 0.90–0.96), glasses of water per day was positively associated with awakening (OR = 1.05; 95% CI: 1.02–1.09) and anxiety (OR = 1.33; 95% CI: 1.00–1.78).

Genetically predicted 10 of 11 dietary habits that are significantly associated with MO in the first step, were observed to be significantly associated with at least one risk factor. Except for the association reported above, wholemeal/wholegrain bread (vs. any other) was consistently negatively associated with SBP (−1.63; 95% CI: −2.68∼−0.58), DBP (−0.91; 95% CI: −1.47∼−0.35), serum total calcium (−0.01; 95% CI: 0.99–1.00), neuroticism (OR = 0.89; 95% CI: 0.83–0.95) and insomnia (OR = 0.76; 95% CI: 0.69–0.83). Other alcohol glasses per month were negatively associated with DBP (−1.39; 95% CI: −2.19∼−0.60).

### 3.3. Association between genetically predicted possible mediators and migraine

We evaluated further whether each of the 8 possible mediators is associated with migraine and its subtypes. The results of MVMR adjusting for different exposure were displayed in Supplementary Tables 34–36, genetically predicted insomnia and MDD were significantly positively associated with migraine (mean OR = 1.20 and 1.49, respectively), MA (mean OR = 1.19 and 1.38, respectively) and MO (mean OR = 1.29 and 1.71, respectively).

### 3.4. The potential mediator role of insomnia and MDD

According to the results of network MR analysis, we found that one or both insomnia and MDD might act as mediators in the pathway from some dietary habits to migraine and MO, those dietary habits include drinking types (drinks usually with meals among current drinkers, overall alcohol intake and red wine glasses per month), bread types (white bread and wholemeal/wholegrain bread), muesli cereal, overall cheese intake and overall oily fish intake. The proportion of the total effect of each dietary habit on migraine or MO that each mediator accounts for was provided in Table 2.

**TABLE 2:** The proportion of the total effect of each dietary habits on migraine that each mediator accounts for.

Exposure	Mediator	Outcome	TE	NIE (95% CI)	Proportion (95% CI)
Bread type: white vs. any other	Insomnia	Migraine	1.511462	1.049 (1.024, 1.075)	11.62% (3.67%, 19.58%)
Bread type: white vs. wholemeal/wholegrain + brown	Insomnia	Migraine	1.42896	1.049 (1.025, 1.073)	13.39% (4.3%, 22.48%)
Bread type: wholemeal/wholegrain vs. white + brown	Insomnia	Migraine	0.760494	0.948 (0.925, 0.971)	19.59% (4.45%, 34.73%)
Cereal type: muesli vs. any other	Insomnia	Migraine	0.651232	0.932 (0.901, 0.963)	16.46% (2.92%, 30%)
Cereal type: muesli vs. any other	MDD	Migraine	0.651232	0.879 (0.816, 0.948)	29.96% (5.54%, 54.38%)
Among current drinkers, drinks usually with meals: yes + it varies vs. no	Insomnia	Migraine	0.609764	0.922 (0.89, 0.955)	16.42% (5.7%, 27.15%)
Among current drinkers, drinks usually with meals: yes + it varies vs. no	MDD	Migraine	0.609764	0.88 (0.823, 0.941)	25.81% (8.86%, 42.77%)
Overall alcohol intake	Insomnia	Migraine	0.740076	0.977 (0.961, 0.993)	7.76% (1.16%, 14.36%)
Overall alcohol intake	MDD	Migraine	0.740076	0.954 (0.922, 0.986)	15.8% (2.91%, 28.7%)
Red wine glasses per month	Insomnia	Migraine	0.648894	0.959 (0.937, 0.982)	9.66% (2.47%, 16.85%)
Red wine glasses per month	MDD	Migraine	0.648894	0.932 (0.887, 0.979)	16.3% (3.33%, 29.27%)
Overall cheese intake	Insomnia	Migraine	0.775472	0.97 (0.951, 0.99)	11.92% (0.18%, 23.67%)
Overall cheese intake	MDD	Migraine	0.775472	0.952 (0.917, 0.988)	19.24% (0.14%, 38.34%)
Overall oily fish intake	Insomnia	Migraine	0.735076	0.97 (0.951, 0.989)	10.05% (1.13%, 18.96%)
Bread type: white vs. any other	Insomnia	MO	1.637199	1.068 (1.032, 1.106)	13.38% (2.91%, 23.85%)
Bread type: white vs. wholemeal/wholegrain + brown	Insomnia	MO	1.505478	1.068 (1.033, 1.104)	16.06% (2.67%, 29.45%)
Bread type: wholemeal/wholegrain vs. white + brown	Insomnia	MO	0.635146	0.928 (0.896, 0.961)	16.5% (4.61%, 28.4%)
Red wine glasses per month	Insomnia	MO	0.542683	0.943 (0.912, 0.976)	9.55% (2.19%, 16.91%)
Red wine glasses per month	MDD	MO	0.542683	0.909 (0.849, 0.974)	15.56% (2.65%, 28.46%)
Overall oily fish intake	Insomnia	MO	0.65074	0.96 (0.934, 0.986)	9.61% (0.29%, 18.94%)

TE, total effect of the exposure on the outcome expressed in odds ratio (OR) scale; NIE, natural indirect effect of exposure on the outcome in OR scale; Proportion, the proportion of the total effect of exposure on outcome that mediator accounts for; CI, confidence interval; MDD, major depression disorder.

### 3.5. Association between migraine and dietary habits

The results of bidirectional MR (Table 3 and Supplementary Tables 37–44) showed evidence of negative association of genetically predicted migraine on drinking types as well as positive association of genetically predicted migraine on cups of tea per day. When stratified by aura status, genetically predicted MA was positively associated with alcohol drinker status: current (+ former) and red wine glasses per month, and genetically predicted MO was positively associated with alcohol drinker status: current (+ former) and beer/cider glasses per month, but negatively associated with cups of tea per day.

**TABLE 3:** Associations between genetically predicted migraine as well as its subtypes and dietary habits with reliable evidence.

Exposure	Outcome	N snps	OR/beta	95% CI	*P*-value	*I* ^2^
Migraine	Alcohol drinker status: current + former vs. never (BI)	119	0.98	(0.98, 0.99)	2.88E-08	27.8113
	Alcohol drinker status: current vs. never (BI)	119	0.98	(0.98, 0.99)	3.89E-09	28.43266
	Beer/cider glasses per month	113	−0.02268	(−0.04, −0.01)	0.002336	44.11665
	Champagne/white wine glasses per month	119	−0.03	(−0.05, 02)	1.31E-05	48.3337
	Total drinks of alcohol per month	117	−0.05905	(−0.07, −0.04)	2.65E-13	60.96577
	Overall alcohol intake	117	−0.0571	(−0.07, −0.04)	1.13E-12	61.41067
	Red wine glasses per month	119	−0.04	(−0.05, 02)	1.40E-09	36.16403
MA	Red wine glasses per month	118	0.040107	(0.03, 0.05)	4.02E-08	37.28982

MA, migraine with aura; MO, migraine without aura; OR, odds ratios; N snps, the number of instrumental SNPs used for each dietary exposure. *I*^2^, the amount of heterogeneity among estimates based on individual SNPs.

## 4. Discussion

### 4.1. Principal findings

In this study, we provided evidence that genetically determined coffee, cheese, oily fish (reliable evidence); raw vegetables, wholemeal/wholegrain bread intake (insufficiency evidence); alcohol (red wine), and muesli (weak evidence) were associated with decreased risk of migraine development; while white bread, cornflakes/frosties, and poultry intake (weak evidence) were positively associated with the risk of migraine development. Additionally, we concluded that genetically predicted white bread (reliable evidence) was associated with a higher risk of insomnia, while wholemeal/wholegrain bread, muesli, oily fish intake (reliable evidence); alcohol (red wine) (reliable/weak evidence); and cheese intake (insufficiency evidence) were associated with decreased risk of insomnia and (or) MDD. One or both of insomnia and MDD may act as a mediator in the pathway from several dietary habits to migraine. Moreover, we found no evidence to support a association of genetically predicted milk, fruit, spread type, salt, and other habits (including never eat eggs, dairy, wheat, or sugar) on migraine as well as its subtypes. Finally, we found evidence of negative and positive associations of genetically predicted migraine on drinking types and cups of tea per day, respectively.

### 4.2. Comparisons with other studies

Our findings for the consistent inverse effect of coffee and alcohol intake on migraine (the former mainly affects MA, while the latter mainly affects MO) as well as migraine on alcohol intake are similar to the results of previously MR study (9). Yuan et al. (9) also reported that genetic liability to migraine was not associated with coffee consumption, which was also shown in our results. We are not aware of any MR study of other dietary habits in relation to migraine.

We should be cautious when comparing our results with previous observational studies, mainly including three reasons. Firstly, there may exist variations in measurement and definition of dietary habits, or the results may base on different populations; secondly, results from observational studies may reflect reverse causation or confounding effects; and thirdly, what is more important, results from most observational studies are associations between dietary habits and the risk/aggravation/frequency of migraine attacks among individuals with migraine, rather than the associations between dietary habits and risk of migraine diagnosis. For instance, inconsistent conclusions for cheese intake (46–53), red wine intake (52, 54, 55) and the consumption of vegetables (46, 56) have been reported by previous observational studies. In addition, milk, egg, wheat, processed meat, and white bread intake were reported triggers for migraine attacks, and wholemeal/wholegrain bread intake is reported to be associated with a reduced risk of migraine attacks in several reviews (7, 57) or observation studies (57–59). However, these results are all from individuals who have migraine and attempting to predict individual attacks, which is not in line with the objective of this study.

The risk of migraine development was less studied by observational studies. One population-based observational study in Japan reported that migraineurs consumed significantly more fatty/oily foods, coffee, and tea than non-headache subjects, while vegetables, fruits, eggs, fish, meat, and milk did not show significant difference between the two groups (56). In this study, we found reliable evidence that cheese intake, red wine intake and tablespoons of raw vegetables per day were associated with a lower risk of migraine and MO diagnosis. Moreover, we showed that oily fish intake and wholemeal/wholegrain bread intake were associated with a reduced risk of migraine and MO, while white bread intake was positively associated with them. Our results supported that overall poultry intake is a potential risk factor of migraine. We found no evidence to support a association of milk, fruit, spread type, salt, and other habits (including never eat eggs, dairy, wheat, or sugar) on migraine, Those contradictions may reflect reverse causation or confounding effects of the observational studies. We are not aware of any observational study of muesli or cornflakes/frosties in relation to migraine. Our study indicated that muesli cereal intake was associated with a decreased risk of migraine, while cornflakes/frosties cereal intake was associated with an increased risk of migraine and MO.

Inconsistent conclusions for association between different definitions of alcohol intake and MDD have been reported by previous MR (60, 61) or observational studies (62, 63). Our results of the potential protective effect of several alcohol intake habits (among current drinkers: drinks usually with meals, red wine glasses per month and overall alcohol intake) on MDD are consistent with the result from a previous MR study shown that regular alcohol consumption was associated with the prevention of depression (61). In addition, Polimanti et al. (64) reported a negative correlation between alcohol consumption frequency and MDD. The association between cheese intake and depression has been inconsistent in previous observational studies (65–67). Nevertheless, we found that cheese intake was positively associated with MDD. Observational studies on the effect of other dietary habits on insomnia or MDD are limited. Our findings provide evidence about the protective or harmful effect of several habits on the risk of insomnia and (or) MDD. Insomnia (depression) and migraine are common complaints among the general population (68, 69), both observational and MR studies reported that individuals with insomnia or MDD had a higher risk of having migraine (12, 14, 70, 71). However, less is known whether they mediate the pathway between dietary habits and migraine. Our study found the potential mediator role of each of them in the pathway from several dietary habits to migraine.

### 4.3. Potential mechanisms

As indicated by Yuan et al. (9), the negative association between genetically predicted alcohol intake and migraine may be due to the stress relieving effect of alcohol as well as the fact that drinkers tend to have a higher tolerance to headache and metabolize alcohol quickly, which makes them less likely to be affected by toxic intermediates that can lead to migraines, such as acetaldehyde (9, 55). In addition, we also cannot rule out the possibility that the protective effect of alcohol consumption on migraine is attributable to reverse causality (9), since alcohol, especially red wine (72), was commonly reported as diet-related trigger of migraine attacks (7), patients with migraine are often limited their alcohol intake (46). Several possible underlying mechanisms underlying the inverse association of coffee intake with the risk of migraine may involve the antagonism of caffeine on the adenosine receptors, this can cause the inhibition of receptors, which may contribute to migraine pathophysiology (73). Some studies showed that caffeine administration increases the production of nitric oxide (NO), which is responsible for vasodilation (74, 75). Moreover, consistent coffee daily intake is possible to prevent caffeine withdrawal headache that can progress to migraines (73). The protective effect of oily fish intake on migraine is possibly due to the anti-inflammatory effect (59) and the suppression of the production of nitric oxide (76) of omega-3 polyunsaturated fatty acids (PUFAs) (77). In addition, omega-3 PUFAs may interact with serotonin and affect neurons and blood vessels (78).

As a dairy product, cheese is rich in various micronutrients and other bioactive compounds (including calcium, magnesium, B complex vitamins and vitamin D, etc.) (79), which might have a protective role in inflammation, oxidation-reduction and B complex vitamins therefore against headaches (48, 80). Leafy vegetables were shown to have a positive impact on neurogenic inflammation through interactions with the calcitonin gene-related peptide receptors (81), an essential inflammatory mediator in migraine pathophysiology. They are also major sources of vitamin C, vitamin E, dietary antioxidants, flavonoids, carotenoids, and minerals (82), and can lower levels of inflammatory biomarkers such as TNF-a (83). Poultry is a type of food with high levels of tyramine, which has been shown to be a valid trigger of headache disorders (84, 85), and cereal type like muesli was found to be inversely associated with inflammatory biomarkers (86).

Previous research indicated that regular alcohol consumption is likely to be associated with better mental health conditions, therefore lowering the risk of depression (61). As a sedative, alcohol may reduce sleep onset latency and proactively relieve insomnia (87, 88). For fish intake, a diet deficient in omega-3 PUFAs has been shown to affect the melatonin rhythm and circadian clock functions, therefore disturbing nocturnal sleep (89). Milk and dairy products such as cheese contain a high amount of tryptophan (Try), from which melatonin is synthesized (90). The level of blood melatonin can induce sleep onset in humans. In addition, cheese belongs to fermented dairy, which is associated with increased strains of beneficial bacteria within the gut microbiota (91) and can lower markers of inflammation and oxidative stress (92) and is further associated with depression (93, 94).

People with depression are more likely to be comorbid with migraine (95). The main possible mechanisms include abnormal brain development and shared genetic basis, as well as neurotransmitters, sex hormones and stress (96), these etiological hypotheses were detailed discussed elsewhere. Potential mechanisms to explain the link between insomnia and migraine may involve the neuronal modifications in the caudate nucleus that is involved in pain suppression (97–99), and the fact that insomnia increases cortical excitability (12, 100). In addition, the state of over-activation of pain-inhibitory circuits in insomnia patients has been shown to cause pain sensation (101).

### 4.4. Strength and limitations

Our study systematically explored the association between dietary habits and migraine as well as its subtypes using two-sample MR analysis, which allowed us to better avoided the limitations of bias arising from confounding, measurement error, as well as reverse causation. In addition, we quantified the proportion of the effect of dietary habits attributable to potential mediator(s) using network MR. Large numbers of participants in publicly available summarized data considerably increase the power to detect an effect. Consistent results from sensitivity analyses that make allowance for the violation of different MR assumptions and replication analyses can further strengthen confidence in causality.

Our study was also subject to several limitations. Firstly, the results from MR only indicate possible causal associations between genetically predicted dietary habits and migraine at the genetic level, we cannot rule out the existence of other pathways that certain dietary habit causes migraine. Secondly, there are overlapping sets of participants between datasets of exposure and several risk factors. Nevertheless, Minelli et al. (102) have shown that most two-sample methods can be safely used for one-sample MR with a large sample size. Thirdly, we cannot exclude the influence of weak instrument bias for two dietary types, including milk type: soy milk vs. never and milk type: other milk vs. never (*F* statistic < 10), which needed further validation in the future. Fourthly, our study conducted Bonferroni correction within each subtype of dietary habits. However, exposures in each group are related to each other, therefore the tests are not completely independent of each other and the Bonferroni correction may be conservative. Considering this, we reported the actual *P*-values of each effect in the results. Fifthly, although we did not find evidence of the potential causal role of genetically predicted milk, fruit, spread type, salt, and other habits (including never eat eggs, dairy, wheat, or sugar) on migraine, we cannot rule out the possibility that these are partly due to the measurement and definition of dietary habits or we have overlooked weak associations due to insufficient power. Sixthly, our results were conducted in individuals of European ancestry, which may limit the generalization of these results to other ethnicities. Sixthly, although MR can address unobserved confounding when the IV assumptions hold, the imperfect selection of instrumental variables in practice use and measurement error in dietary habit traits (assessed using touchscreen questionnaire) may bias the MR estimates. Finally, several dietary habits are poorly explored in this work, e.g., only “cups of tea per day” was included as tea intake-related exposure, however, different types of tea or drinking habits (such as alkaloids content) may have different effects on migraine or other risk factors. In addition, since the type and concentration of several dietary habits are not fully considered, for instance, “cups of coffee per day” can only approximate the levels of caffeine consumed per day, not strict equivalent, and the consumption of certain food (e.g., oil fish) not equal the level of active ingredients or dietary supplements in this diet (e.g., omega-3). These can be further explored in future studies. The association between dietary choices and the microbiome as well as the association between the choices and other health behaviors may also be a direction for further research to better understand the mechanisms underpinning the association between dietary habits and migraine.

## 5. Conclusion

In conclusion, our study provides evidence that genetically determined drinking (eating) more coffee, cheese, oily fish, raw vegetables, wholemeal/wholegrain bread, alcohol (red wine), and muesli intake are good for the prevention of migraine development, and more white bread, cornflakes/frosties, and poultry consumption can increase the risk of migraine. Moreover, we identified the potential mediator role of insomnia and MDD. Migraine is also associated with decreased alcohol assumption and increased tea intake. Further investigations confirming the biological rationality and clarifying the biological mechanism of several identified dietary habits expected to participate in the development of migraine are needed.

## Data availability statement

The original contributions presented in this study are included in the article/Supplementary material, further inquiries can be directed to the corresponding authors.

## Author contributions

XL, HL, and FX: conceptualization. XL: data curation and writing—original draft. HL and FX: funding acquisition. XL, YuY, LH, and YiY: investigation. FX: project administration. YuY, YWu, SW, YH, YG, YWe, QL, FQ, YF, HL, and FX: writing—review and editing. All authors contributed to the article and approved the submitted version.
