# Supplementary material for: Association between dietary habits and the risk of migraine: a Mendelian randomization study
Source: Front Nutr. 2023 Jun 7;10:1123657. doi: 10.3389/fnut.2023.1123657 (PMC10282154; doi:10.3389/fnut.2023.1123657)
Supplement: Supplementary file 1 [file Data_Sheet_1.docx]

**Supplementary materials**

**Table S1. The basic characters of summary data of 83 dietary habits.**

| **Dietary Habit** | **UKB Field** | **Sample Size** | **N snps** | ***F* statistics** | **Class.** | **Number** | **α after Bonferroni correction** | **ACE** **touchscreen question** |
| --- | --- | --- | --- | --- | --- | --- | --- | --- |
| alcohol drinker status: current + former vs. never | 20117 | 448,623 | 44 | 33.02854779 | 1 | 13 | 0.003846154 | - |
| alcohol drinker status: current vs. never | 20117 | 433,353 | 43 | 32.58506621 | 1 | 13 | 0.003846154 |
| among current drinkers, drinks usually with meals: yes + it varies vs. no | 1618 | 357,136 | 107 | 30.16489134 | 1 | 13 | 0.003846154 | "When you drink alcohol is it usually with meals?" |
| among current drinkers, drinks usually with meals: yes vs. no | 1618 | 235,312 | 164 | 24.83034593 | 1 | 13 | 0.003846154 |
| among current drinkers, drinks usually with meals: yes, it varies, no | 1618 | 357,136 | 135 | 30.48252522 | 1 | 13 | 0.003846154 |
| champagne/white wine glasses per month | 1578, 4418 | 387,404 | 75 | 32.1944627 | 1 | 13 | 0.003846154 | "In an average WEEK, how many glasses of WHITE wine or champagne would you drink? (There are six glasses in an average bottle)" |
| red wine glasses per month | 1578, 4419 | 387,404 | 135 | 32.87953952 | 1 | 13 | 0.003846154 |
| beer/cider glasses per month | 1578, 4420 | 387,404 | 115 | 33.50794187 | 1 | 13 | 0.003846154 |
| spirits measures per month | 1578, 4421 | 387,404 | 45 | 29.98630337 | 1 | 13 | 0.003846154 |
| fortwine glasses per month | 1578, 4422 | 387,404 | 15 | 29.65913707 | 1 | 13 | 0.003846154 |
| other alcohol glasses per month | 1578, 4423 | 186,160 | 11 | 20.3727014 | 1 | 13 | 0.003846154 |
| total drinks of alcohol per month | 1578, 4424 | 449,210 | 281 | 37.60142308 | 1 | 13 | 0.003846154 |
| overall alcohol intake | 1558 | 448,623 | 282 | 37.23132649 | 1 | 13 | 0.003846154 | "About how often do you drink alcohol?" (If this varies a lot, please provide an average considering your intake over the last year) |
| milk type: dairy-based milk vs. never | 1418 | 427,252 | 19 | 31.89319327 | 2 | 13 | 0.003846154 | "What type of milk do you mainly use?" (If you use more than one type of milk, please select the one that you drink the most.) |
| milk type: any milk vs. never | 1418 | 448,698 | 20 | 33.00170101 | 2 | 13 | 0.003846154 |
| milk type: full cream vs. never | 1418 | 43,995 | 26 | 10.06361688 | 2 | 13 | 0.003846154 |
| milk type: full cream vs. any other | 1418 | 448,698 | 36 | 34.00291064 | 2 | 13 | 0.003846154 |
| milk type: semi-skimmed vs. never | 1418 | 308029 | 20 | 27.31889303 | 2 | 13 | 0.003846154 |
| milk type: semi-skimmed vs. any other | 1418 | 448,698 | 31 | 32.26646045 | 2 | 13 | 0.003846154 |
| milk type: skimmed vs. never | 1418 | 108,035 | 23 | 15.97356631 | 2 | 13 | 0.003846154 |
| milk type: skimmed vs. any other | 1418 | 448,698 | 40 | 33.72704611 | 2 | 13 | 0.003846154 |
| milk type: soy milk vs. never | 1418 | 31,889 | 26 | 8.627932808 | 2 | 13 | 0.003846154 |
| milk type: soy milk vs. any other | 1418 | 448,698 | 22 | 33.16290692 | 2 | 13 | 0.003846154 |
| milk type: other milk vs. never | 1418 | 20,557 | 14 | 6.832388958 | 2 | 13 | 0.003846154 |
| milk type: other milk vs. any other | 1418 | 448,698 | 14 | 32.15750531 | 2 | 13 | 0.003846154 |
| milk type: skimmed, semi-skimmed, full cream (QT) | 1418 | 427,252 | 54 | 33.36642493 | 2 | 13 | 0.003846154 |
| pieces of fresh fruit per day | 1309 | 447,401 | 256 | 37.66935644 | 3 | 2 | 0.025 | "About how many pieces of FRESH fruit would you eat per DAY? (Count one apple, one banana, 10 grapes etc as one piece; put '0' if you do not eat any)" (Please provide an average considering your intake over the last year) |
| pieces of dried fruit per day | 1319 | 444,741 | 119 | 35.19930813 | 3 | 2 | 0.025 | "About how many pieces of DRIED fruit would you eat per DAY? (Count one prune, one dried apricot, 10 raisins as one piece; put '0' if you do not eat any)" (Please provide an average considering your intake over the last year) |
| tablespoons of cooked vegetables per day | 1289 | 444,190 | 132 | 34.46889771 | 4 | 2 | 0.025 | "On average how many heaped tablespoons of COOKED vegetables would you eat per DAY? (Do not include potatoes; put '0' if you do not eat any)" (Please provide an average considering your intake over the last year) |
| tablespoons of raw vegetables per day | 1299 | 443,633 | 146 | 34.99543191 | 4 | 2 | 0.025 | "On average how many heaped tablespoons of SALAD or RAW vegetables would you eat per DAY? (Include lettuce, tomato in sandwiches; put '0' if you do not eat any)" (Please provide an average considering your intake over the last year) |
| bread type: white vs. any other | 1448 | 434,087 | 162 | 34.51129376 | 5 | 6 | 0.008333333 | "What type of bread do you mainly eat?" (If you eat more than one type of bread, please select the one  that you eat the most.) |
| bread type: brown vs. any other | 1448 | 434,087 | 21 | 31.79980135 | 5 | 6 | 0.008333333 |
| bread type: wholemeal/wholegrain vs. any other | 1448 | 434,087 | 147 | 34.1286863 | 5 | 6 | 0.008333333 |
| bread type: white vs. wholemeal/wholegrain + brown | 1448 | 416,312 | 172 | 33.60940945 | 5 | 6 | 0.008333333 |
| bread type: wholemeal/wholegrain vs. white + brown | 1448 | 416,312 | 169 | 33.47107924 | 5 | 6 | 0.008333333 |
| cups of tea per day | 1488 | 448,060 | 179 | 36.89998217 | 7 | 1 | 0.05 |
| cereal type: biscuit cereal vs. any other | 1448 | 373,443 | 29 | 29.3654388 | 8 | 6 | 0.008333333 |
| cereal type: bran cereal vs. any other | 1448 | 373,443 | 21 | 29.59781607 | 8 | 6 | 0.008333333 |
| cereal type: oat cereal vs. any other | 1448 | 373,443 | 26 | 30.10316139 | 8 | 6 | 0.008333333 |
| cereal type: muesli vs. any other | 1448 | 373,443 | 86 | 31.32867016 | 8 | 6 | 0.008333333 |
| cereal type: cornflakes/frosties vs. any other | 1448 | 373,443 | 79 | 31.94998793 | 8 | 6 | 0.008333333 |
| slices of bread per week | 1438 | 444,230 | 133 | 35.06373486 | 5 | 6 | 0.008333333 | "How many slices of bread do you eat each WEEK?" (For other types of bread: - one bread roll = 2 slices; - one pitta bread = 2 slices) |
| coffee type: decaffeinated vs. any other | 1508 | 353,710 | 150 | 31.25302469 | 6 | 4 | 0.0125 | "What type of coffee do you usually drink?" (If you drink more than one type of coffee, please select the one that you drink the most.) |
| coffee type: ground+instant vs. other+decaff | 1508 | 353,710 | 35 | 29.33150958 | 6 | 4 | 0.0125 |
| coffee type: ground vs. any other | 1508 | 353,710 | 29 | 29.19471469 | 6 | 4 | 0.0125 |
| cups of coffee per day | 1498 | 448,204 | 139 | 39.39280821 | 6 | 4 | 0.0125 | "How many cups of coffee do you drink each DAY? (Include decaffeinated coffee)" (Please provide an average considering your intake over the last year.) |
| bowls of cereal per week | 1458 | 447,935 | 173 | 36.11189924 | 8 | 6 | 0.008333333 | "How many bowls of cereal do you eat a WEEK?" (Please provide an average considering your intake over the last year.) |
| overall processed meat intake | 1349 | 448,303 | 107 | 34.05886478 | 9 | 5 | 0.01 | "How often do you eat processed meats (such as bacon, ham, sausages, meat pies, kebabs, burgers, chicken nuggets)?" (Please provide an average considering your intake over the last year) |
| overall poultry intake | 1359 | 448,210 | 62 | 32.83683273 | 9 | 5 | 0.01 | "How often do you eat chicken, turkey or other poultry? (Do not count processed meats)" (Please provide an average considering your intake over the last year) |
| overall beef intake | 1369 | 447,441 | 107 | 34.13367217 | 9 | 5 | 0.01 | "How often do you eat beef? (Do not count processed meats)" (Please provide an average considering your intake over the last year) |
| overall lamb/mutton intake | 1379 | 446,443 | 123 | 34.79118698 | 9 | 5 | 0.01 | "How often do you eat lamb/mutton? (Do not count processed meats)" (Please provide an average considering your intake over the last year) |
| overall pork intake | 1389 | 446,607 | 82 | 33.72914247 | 9 | 5 | 0.01 | "How often do you eat pork? (Do not count processed meats such as bacon or ham)" (Please provide an average considering your intake over the last year) |
| overall oily fish intake | 1329 | 446,854 | 192 | 37.07173258 | 10 | 2 | 0.025 | "How often do you eat oily fish? (e.g. sardines, salmon, mackerel, herring)" (Please provide an average considering your intake over the last year) |
| overall non-oily fish intake | 1339 | 447,289 | 77 | 33.77877885 | 10 | 2 | 0.025 | "How often do you eat other types of fish? (e.g. cod, tinned tuna, haddock)" (Please provide an average considering your intake over the last year) |
| spread type: all spreads vs. never | 1428 | 448,696 | 50 | 33.70591595 | 11 | 17 | 0.002941176 | "What type of spread do you mainly use?" (If you use more than one type of spread, please select the one that you use the most.) |
| spread type: butter + margarine vs. never | 1428 | 281,792 | 55 | 27.0605918 | 11 | 17 | 0.002941176 |
| spread type: any oil based spread vs. never | 1428 | 186,666 | 32 | 21.5715077 | 11 | 17 | 0.002941176 |
| spread type: butter and butter-like spreads vs. oil-based spreads | 1428 | 371,119 | 44 | 29.99281964 | 11 | 17 | 0.002941176 |
| spread type: butter and margarine spreads vs. oil-based spreads | 1428 | 333,836 | 57 | 29.1322253 | 11 | 17 | 0.002941176 |
| spread type: butter vs. never | 1428 | 213,549 | 53 | 24.12381321 | 11 | 17 | 0.002941176 |
| spread type: butter vs. any other | 1428 | 448,696 | 93 | 34.03991643 | 11 | 17 | 0.002941176 |
| spread type: tub margarine vs. never | 1428 | 77,738 | 27 | 13.90560126 | 11 | 17 | 0.002941176 |
| spread type: tub margarine vs. any other | 1428 | 448,696 | 27 | 32.31877865 | 11 | 17 | 0.002941176 |
| spread type: flora + benecol vs. never | 1428 | 86,823 | 27 | 14.3655243 | 11 | 17 | 0.002941176 |
| spread type: flora + benecol vs. any other | 1428 | 448,696 | 39 | 37.41027338 | 11 | 17 | 0.002941176 |
| spread type: olive oil spread vs. never | 1428 | 106,711 | 25 | 15.87750411 | 11 | 17 | 0.002941176 |
| spread type: olive oil spread vs. any other | 1428 | 448,696 | 17 | 32.14431459 | 11 | 17 | 0.002941176 |
| spread type: other oil-based spread vs. never | 1428 | 128,390 | 38 | 17.46184098 | 11 | 17 | 0.002941176 |
| spread type: other oil-based spread vs. any other | 1428 | 448,696 | 25 | 32.32737406 | 11 | 17 | 0.002941176 |
| spread type: low fat spread vs. never | 1428 | 72,017 | 12 | 12.79871108 | 11 | 17 | 0.002941176 |
| spread type: low fat spread vs. any other | 1428 | 448,696 | 17 | 32.55715841 | 11 | 17 | 0.002941176 |
| glasses of water per day | 1528 | 445,965 | 262 | 36.2884705 | 12 | 1 | 0.05 | "How many glasses of water do you drink each DAY? " (Please provide an average considering your intake over the last year) |
| frequency of adding salt to food | 1478 | 448,890 | 230 | 37.11568515 | 13 | 1 | 0.05 | "Do you add salt to your food? (Do not include salt used in cooking)" (Please provide an average considering your intake over the last year) |
| temperature of hot drinks | 1518 | 448,817 | 184 | 36.01916627 | 14 | 1 | 0.05 | "How do you like your hot drinks? (Such as coffee or tea)" |
| overall cheese intake | 1408 | 438,453 | 217 | 34.79614814 | 15 | 1 | 0.05 | "How often do you eat cheese? (Include cheese in pizzas, quiches, cheese sauce etc)" (Please provide an average considering your intake over the last year) |
| never eat eggs vs. no eggs, dairy, wheat, or sugar restrictions | 6144 | 357,255 | 12 | 28.43567529 | 16 | 8 | 0.00625 | "Which of the following do you NEVER eat? (You can select more than one answer)" |
| never eat eggs vs. no eggs restrictions | 6144 | 447,391 | 8 | 31.83074794 | 16 | 8 | 0.00625 |
| never eat dairy vs. no eggs, dairy, wheat, or sugar restrictions | 6144 | 355,549 | 17 | 28.73166707 | 16 | 8 | 0.00625 |
| never eat dairy vs. no dairy restrictions | 6144 | 447,391 | 17 | 31.84547537 | 16 | 8 | 0.00625 |
| never eat wheat vs. no eggs, dairy, wheat, or sugar restrictions | 6144 | 357,603 | 20 | 50.52178601 | 16 | 8 | 0.00625 |
| never eat wheat vs. no wheat restrictions | 6144 | 447,391 | 21 | 55.92676941 | 16 | 8 | 0.00625 |
| never eat sugar vs. no eggs, dairy, wheat, or sugar restrictions | 6144 | 427,790 | 125 | 33.23870285 | 16 | 8 | 0.00625 |
| never eat sugar vs. no sugar restrictions | 6144 | 447,391 | 120 | 34.08726998 | 16 | 8 | 0.00625 |

N snps: the number of instrumental SNPs for each dietary exposure; Class.: Classification, including 1: drinking; 2: milk; 3: fruit; 4: vegetable; 5: bread; 6: coffee; 7: tea; 8: cereal; 9: meat; 10: fish; 11: spread type; 12: water; 13: salt; 14: hot drink; 15: cheese; 16: others.

**Table S2. The details of genome-wide association studies of 8 migraine risk factors.**

| **Trait** | **Consortium** | **Sample Size** | **Units** | **Participants** | **Study** | **Web source** |
| --- | --- | --- | --- | --- | --- | --- |
| Systolic blood pressure | The International Consortium of Blood Pressure (ICBP). (AGES, ARIC, ASPS, B58C, BHS, CHS, COLAUS, CORO ALL, CROATIA-Korcula, CROATIA-Split, CROATIA-Vis, EGCUT, EGCUT2, EPIC, ERF, Fenland, FHS, FINNRISK CASE ALL, FINRISK CTRL ALL, FUSION, GRAPHIC, H2000 ALL, Health ABC, HTO, INGI_VB, INGI-CARL, Cilento study, INGI-FVG, IPM, KORAS3, KORAS4, LBC1921, LBC1936, LOLIPOP_EW610, MESA, MICROS, MIGen, NESDA, NSPHS, NTR, ORCADES, PROSPER, PIVUS, PROCARDIS, RSI, RSII, RSIII, SHIP, STR, TRAILS, TRAILS-CC, ULSAM, WGHS, YFS, ASCOT-SC, ASCOT-UK, BRIGHT, 3C-DIJON, EPIC-CVD, GWASFenland, OMICS-Fenland, EPIC-InterAct, EPIC-Norfolk, GAPP, GoDARTS, GS:SFHS, HCS, JUPITER, Lifelines, MDC, METSIM, NEO, PREVEND, SardiNIA, TWINSUK, UKHLS, UK Biobank) | 458,575 | 1 SD = 20.7 mm Hg | European | the Million Veteran Program, Evangelou E, Warren HR, Mosen-Ansorena D, Mifsud B, Pazoki R, Gao H, Ntritsos G, Dimou N, Cabrera CP, et al. Genetic analysis of over 1 million people identifies 535 new loci associated with blood pressure traits. Nat Genet (2018) 50:1412–1425. doi: 10.1038/s41588-018-0205-x | https://grasp.nhlbi.nih.gov/FullResults.aspx |
| Diastolic blood pressure |  | 458,577 | 1 SD = 11.3 mm Hg |  |  |  |
| Serum total calcium | UK Biobank | 305,349 | mmol/L | European | Young WJ, Warren HR, Mook-Kanamori DO, Ramírez J, van Duijvenboden S, Orini M, Tinker A, van Heemst D, Lambiase PD, Jukema JW, et al. Genetically Determined Serum Calcium Levels and Markers of Ventricular Repolarization: A Mendelian Randomization Study in the UK Biobank. Circ Genomic Precis Med (2021) 14:e003231. doi: 10.1161/CIRCGEN.120.003231 | https://www.ahajournals.org/action/downloadSupplement?doi=10.1161%2FCIRCGEN.120.003231&file=CIRCGENETICS_CIRCCVG-2020-003231_supp1.pdf |
| Neuroticism | UK Biobank, 23andMe, Genetics of Personality Consortium | 449,484 | - | European | 23andMe Research Team, Nagel M, Jansen PR, Stringer S, Watanabe K, de Leeuw CA, Bryois J, Savage JE, Hammerschlag AR, Skene NG, et al. Meta-analysis of genome-wide association studies for neuroticism in 449,484 individuals identifies novel genetic loci and pathways. Nat Genet (2018) 50:920–927. doi: 10.1038/s41588-018-0151-7 | the Department of Complex Trait Genetics, CNCR (https://ctg.cncr.nl/software/summary_statistics/) |
| Difficulty awakening | UK Biobank | 451,872 | - | European | Nealelab | http://www.nealelab.is/uk-biobank/ |
| Insomnia | UK Biobank, 23andMe | 397,959 / 933,051 | - | European | The 23andMe Research Team, Jansen PR, Watanabe K, Stringer S, Skene N, Bryois J, Hammerschlag AR, de Leeuw CA, Benjamins JS, Muñoz-Manchado AB, et al. Genome-wide analysis of insomnia in 1,331,010 individuals identifies new risk loci and functional pathways. Nat Genet (2019) 51:394–403. doi: 10.1038/s41588-018-0333-3 | http://dx.doi.org/10.7488/ds/2458. |
| Major depression disorder | 23andMe_307k, UK Biobank, PGC_139k | 807,553 (246,363 / 561,190 ) | - | European | Howard DM, Adams MJ, Clarke T-K, Hafferty JD, Gibson J, Shirali M, Coleman JRI, Hagenaars SP, Ward J, Wigmore EM, et al. Genome-wide meta-analysis of depression identifies 102 independent variants and highlights the importance of the prefrontal brain regions. Nat Neurosci (2019) 22:343–352. doi: 10.1038/s41593-018-0326-7 | http://dx.doi.org/10.7488/ds/2458. |
| Anxiety | Integrative Psychiatric Research (iPSYCH) | 23,809 (4,584 / 19,225) | - | European | Meier SM, Trontti K, Purves KL, Als TD, Grove J, Laine M, Pedersen MG, Bybjerg-Grauholm J, Bækved-Hansen M, Sokolowska E, et al. Genetic Variants Associated With Anxiety and Stress-Related Disorders: A Genome-Wide Association Study and Mouse-Model Study. JAMA Psychiatry (2019) 76:924. doi: 10.1001/jamapsychiatry.2019.1119 | https://ipsych.dk/en/research/downloads |
| Alcohol consumption | GWAS and Sequencing  Consortium of Alcohol  and Nicotine use  (GSCAN) | 941,280 | SD of log-transformed weekly alcohol drinks consumed | European | 23andMe Research Team, HUNT All-In Psychiatry, Liu M, Jiang Y, Wedow R, Li Y, Brazel DM, Chen F, Datta G, Davila-Velderrain J, et al. Association studies of up to 1.2 million individuals yield new insights into the genetic etiology of tobacco and alcohol use. Nat Genet (2019) 51:237–244. doi: 10.1038/s41588-018-0307-5 | https://conservancy.umn.edu/ha  ndle/11299/201564 |
| Major depression disorder* | PGC29, deCODE, GenScotland, GERA, iPSYCH, UK Biobank, 23andMe | 135,458 / 344,901 | - | European | eQTLGen, 23andMe, the Major Depressive Disorder Working Group of the Psychiatric Genomics Consortium, Wray NR, Ripke S, Mattheisen M, Trzaskowski M, Byrne EM, Abdellaoui A, Adams MJ, et al. Genome-wide association analyses identify 44 risk variants and refine the genetic architecture of major depression. Nat Genet (2018) 50:668–681. doi: 10.1038/s41588-018-0090-3 | https://figshare.com/articles/dataset/mdd2018/14672085 |
| Cheese intake | UK biobank | 451,486 | SD | European | MRC IEU | https://gwas.mrcieu.ac.uk/ |
| Average weekly red wine intake | UK biobank | 257,773 | glasses | European | Nealelab | http://www.nealelab.is/uk-biobank/ |

SD: standard deviation.

**Table S3. Associations between genetically predicted dietary habits and migraine as well as its subtypes using IVW.**

| **Exposure** | **N snps** | **Migraine** | | | | **Migraine with aura** | | | | **Migraine without aura** | | | |
| --- | --- | --- | --- | --- | --- | --- | --- | --- | --- | --- | --- | --- | --- |
|  |  | **OR** | **95% CI** | **P value** | **I^2^** | **OR** | **95% CI** | **P value** | **I^2^** | **OR** | **95% CI** | **P value** | **I^2^** |
| alcohol drinker status: current + former vs. never | 44 | 0.939114 | [0.36,2.46] | 0.898247 | 17.59024 | 1.24577 | [0.34,4.58] | 0.740827 | 0 | 0.854428 | [0.17,4.22] | 0.846816 | 25.4869 |
| alcohol drinker status: current vs. never | 43 | 1.107373 | [0.41,2.97] | 0.839321 | 26.04834 | 1.662087 | [0.47,5.88] | 0.430465 | 0 | 0.754563 | [0.14,3.93] | 0.737984 | 34.40393 |
| among current drinkers, drinks usually with meals: yes + it varies vs. no | 107 | 0.609764 | [0.47,0.8] | 0.000325 | 15.45143 | 0.597353 | [0.4,0.88] | 0.009559 | 10.05147 | 0.588377 | [0.39,0.9] | 0.014158 | 14.72247 |
| among current drinkers, drinks usually with meals: yes vs. no | 164 | 0.831434 | [0.71,0.98] | 0.026948 | 18.36784 | 0.858019 | [0.69,1.07] | 0.172757 | 0 | 0.737038 | [0.58,0.94] | 0.012532 | 5.32628 |
| never eat eggs vs. no eggs, dairy, wheat, or sugar restrictions | 12 | 2.11749 | [0.3,15.07] | 0.453698 | 41.33578 | 1.086554 | [0.06,18.49] | 0.954221 | 37.53867 | 1.290031 | [0.06,26.52] | 0.868865 | 38.30824 |
| never eat eggs vs. no eggs restrictions | 8 | 5.107764 | [0.54,48.58] | 0.155882 | 0 | 1.524696 | [0.05,43.69] | 0.80538 | 0 | 6.878297 | [0.19,246.3] | 0.290841 | 1.171943 |
| never eat dairy vs. no eggs, dairy, wheat, or sugar restrictions | 17 | 1.130723 | [0.24,5.38] | 0.8773 | 36.25381 | 1.031982 | [0.11,9.57] | 0.977895 | 30.53238 | 0.610993 | [0.08,4.57] | 0.631192 | 4.472781 |
| never eat dairy vs. no dairy restrictions | 17 | 1.17804 | [0.17,8.28] | 0.869228 | 37.47551 | 1.031694 | [0.06,16.53] | 0.982411 | 31.30716 | 0.525092 | [0.04,6.4] | 0.61357 | 5.130025 |
| never eat wheat vs. no eggs, dairy, wheat, or sugar restrictions | 20 | 1.298814 | [0.67,2.53] | 0.442569 | 0 | 1.406895 | [0.52,3.81] | 0.502024 | 0 | 0.481892 | [0.17,1.39] | 0.176013 | 0 |
| never eat wheat vs. no wheat restrictions | 21 | 1.671073 | [0.75,3.71] | 0.207651 | 0 | 1.802636 | [0.55,5.94] | 0.332941 | 0 | 0.59889 | [0.17,2.12] | 0.427054 | 0 |
| never eat sugar vs. no eggs, dairy, wheat, or sugar restrictions | 125 | 1.104382 | [0.84,1.46] | 0.485943 | 15.70027 | 0.825353 | [0.53,1.29] | 0.399674 | 26.87026 | 1.043213 | [0.69,1.57] | 0.840051 | 3.037116 |
| never eat sugar vs. no sugar restrictions | 120 | 0.999028 | [0.75,1.34] | 0.994801 | 21.61854 | 0.809797 | [0.51,1.28] | 0.368931 | 29.70345 | 1.075077 | [0.71,1.62] | 0.728465 | 0 |
| milk type: dairy-based milk vs. never | 19 | 0.822382 | [0.17,3.96] | 0.807269 | 37.78441 | 1.881588 | [0.3,11.94] | 0.502518 | 0 | 0.379448 | [0.02,6.6] | 0.506047 | 53.13094 |
| milk type: any milk vs. never | 20 | 0.4865 | [0.11,2.17] | 0.344549 | 35.14672 | 0.810588 | [0.13,4.87] | 0.818418 | 0 | 0.629568 | [0.05,8.73] | 0.730201 | 47.9347 |
| milk type: full cream vs. never | 26 | 1.087977 | [0.93,1.28] | 0.29969 | 0 | 1.071334 | [0.84,1.37] | 0.587516 | 9.129737 | 0.962726 | [0.75,1.24] | 0.766912 | 0 |
| milk type: full cream vs. any other | 36 | 0.978477 | [0.49,1.94] | 0.950247 | 0 | 0.681886 | [0.25,1.89] | 0.461142 | 0 | 1.612074 | [0.55,4.74] | 0.38531 | 0 |
| milk type: semi-skimmed vs. never | 20 | 1.207672 | [0.44,3.3] | 0.712915 | 19.39232 | 3.408732 | [0.89,13.09] | 0.074097 | 0 | 0.345114 | [0.05,2.64] | 0.305183 | 50.94802 |
| milk type: semi-skimmed vs. any other | 31 | 0.774291 | [0.51,1.17] | 0.227163 | 0 | 0.995134 | [0.54,1.85] | 0.987664 | 0 | 0.766251 | [0.4,1.47] | 0.425355 | 0 |
| milk type: skimmed vs. never | 23 | 1.064144 | [0.7,1.63] | 0.77422 | 38.16686 | 1.01605 | [0.62,1.67] | 0.950013 | 0 | 1.252658 | [0.7,2.24] | 0.449097 | 18.40132 |
| milk type: skimmed vs. any other | 40 | 1.061395 | [0.68,1.66] | 0.793978 | 7.402346 | 0.90635 | [0.48,1.72] | 0.764522 | 0.835185 | 1.519057 | [0.69,3.36] | 0.301606 | 26.835 |
| milk type: soy milk vs. never | 26 | 1.066059 | [0.93,1.22] | 0.361096 | 0 | 0.932359 | [0.76,1.14] | 0.502198 | 0 | 1.247059 | [1,1.55]* | 0.045658 | 0 |
| milk type: soy milk vs. any other | 22 | 0.767535 | [0.25,2.39] | 0.64853 | 0 | 1.139329 | [0.21,6.21] | 0.880141 | 0 | 0.298229 | [0.05,1.79] | 0.186385 | 0 |
| milk type: other milk vs. never | 14 | 0.910643 | [0.76,1.1] | 0.322542 | 13.61347 | 0.927166 | [0.72,1.2] | 0.564404 | 0 | 0.761372 | [0.56,1.03] | 0.076604 | 18.81131 |
| milk type: other milk vs. any other | 14 | 0.806037 | [0.03,21.72] | 0.897906 | 47.6485 | 0.110576 | [0,14.31] | 0.374793 | 46.5932 | 0.117487 | [0,19.08] | 0.409617 | 45.57683 |
| spread type: all spreads vs. never | 50 | 0.731605 | [0.45,1.2] | 0.212989 | 10.78794 | 0.565062 | [0.28,1.13] | 0.105834 | 0 | 0.867341 | [0.41,1.84] | 0.711021 | 5.215228 |
| spread type: butter + margarine vs. never | 55 | 0.954232 | [0.7,1.31] | 0.770108 | 7.944454 | 0.776786 | [0.5,1.22] | 0.27028 | 0 | 1.117693 | [0.66,1.89] | 0.677533 | 17.73405 |
| spread type: any oil based spread vs. never | 32 | 1.179317 | [0.87,1.61] | 0.294865 | 11.73066 | 1.240832 | [0.78,1.96] | 0.35751 | 11.70395 | 1.350581 | [0.85,2.13] | 0.198025 | 0 |
| spread type: butter and butter-like spreads vs. oil-based spreads | 44 | 1.340925 | [0.97,1.84] | 0.071376 | 0 | 1.903685 | [1.13,3.2] | 0.014883 | 15.88621 | 0.920698 | [0.54,1.58] | 0.763188 | 12.27139 |
| spread type: butter and margarine spreads vs. oil-based spreads | 57 | 1.161115 | [0.87,1.55] | 0.30981 | 8.979156 | 1.44103 | [0.9,2.3] | 0.124955 | 22.91606 | 1.081134 | [0.7,1.68] | 0.728798 | 3.169643 |
| spread type: butter vs. never | 53 | 0.919552 | [0.72,1.18] | 0.510651 | 6.557796 | 0.889786 | [0.62,1.28] | 0.524655 | 0 | 1.09143 | [0.75,1.6] | 0.652716 | 0 |
| spread type: butter vs. any other | 93 | 1.101163 | [0.85,1.43] | 0.475093 | 8.843843 | 1.014875 | [0.69,1.5] | 0.940796 | 6.836653 | 0.862191 | [0.58,1.28] | 0.465374 | 0 |
| spread type: tub margarine vs. never | 27 | 0.876917 | [0.7,1.09] | 0.244069 | 18.30948 | 0.943321 | [0.68,1.31] | 0.730219 | 19.49209 | 1.03694 | [0.76,1.42] | 0.821404 | 0 |
| spread type: tub margarine vs. any other | 27 | 1.011707 | [0.38,2.67] | 0.981256 | 29.48946 | 1.641671 | [0.33,8.15] | 0.544263 | 42.57574 | 2.715168 | [0.66,11.25] | 0.168557 | 18.28102 |
| spread type: flora + benecol vs. never | 27 | 0.967312 | [0.79,1.19] | 0.748456 | 0 | 0.935572 | [0.69,1.27] | 0.666249 | 0 | 1.029606 | [0.75,1.42] | 0.85843 | 0 |
| spread type: flora + benecol vs. any other | 39 | 0.962158 | [0.53,1.75] | 0.89959 | 17.09431 | 1.214856 | [0.52,2.84] | 0.652711 | 8.060296 | 0.600731 | [0.24,1.49] | 0.271804 | 10.42259 |
| spread type: olive oil spread vs. never | 25 | 0.91545 | [0.71,1.17] | 0.487434 | 17.15025 | 0.943519 | [0.66,1.35] | 0.749727 | 10.47412 | 1.257741 | [0.88,1.8] | 0.209146 | 0 |
| spread type: olive oil spread vs. any other | 17 | 0.549445 | [0.27,1.11] | 0.093791 | 0 | 1.258613 | [0.44,3.56] | 0.665015 | 0 | 0.45351 | [0.13,1.61] | 0.220171 | 23.60025 |
| spread type: other oil-based spread vs. never | 38 | 1.331047 | [1.07,1.66] | 0.01053 | 0 | 1.387048 | [1,1.92]* | 0.049566 | 0 | 1.529831 | [1.08,2.17] | 0.016789 | 1.505201 |
| spread type: other oil-based spread vs. any other | 25 | 0.97809 | [0.52,1.85] | 0.945669 | 17.38085 | 0.680339 | [0.29,1.61] | 0.381294 | 0 | 1.408935 | [0.48,4.11] | 0.530179 | 27.19317 |
| spread type: low fat spread vs. never | 12 | 1.068924 | [0.78,1.46] | 0.674087 | 37.48101 | 0.894547 | [0.61,1.32] | 0.573803 | 11.20684 | 0.978183 | [0.66,1.44] | 0.911165 | 0 |
| spread type: low fat spread vs. any other | 17 | 0.387709 | [0.09,1.74] | 0.215554 | 45.21599 | 0.435855 | [0.08,2.28] | 0.324745 | 0 | 0.430542 | [0.07,2.73] | 0.370859 | 10.09707 |
| bread type: white vs. any other | 162 | 1.511462 | [1.22,1.88] | 0.000185 | 14.75844 | 1.298949 | [0.95,1.78] | 0.102452 | 9.989212 | 1.637199 | [1.19,2.24] | 0.002182 | 0 |
| bread type: brown vs. any other | 21 | 0.868759 | [0.43,1.77] | 0.699512 | 0 | 0.320205 | [0.11,0.93] | 0.035854 | 0 | 1.344955 | [0.37,4.89] | 0.652571 | 23.7962 |
| bread type: wholemeal/wholegrain vs. any other | 147 | 0.816432 | [0.66,1] | 0.053422 | 14.24542 | 0.817445 | [0.61,1.1] | 0.183753 | 8.718996 | 0.636267 | [0.47,0.86] | 0.003198 | 0 |
| bread type: white vs. wholemeal/wholegrain + brown | 172 | 1.42896 | [1.18,1.73] | 0.00028 | 5.664432 | 1.268767 | [0.95,1.7] | 0.109563 | 8.698017 | 1.505478 | [1.12,2.02] | 0.006566 | 0 |
| bread type: wholemeal/wholegrain vs. white + brown | 169 | 0.760494 | [0.63,0.92] | 0.004438 | 12.38416 | 0.778138 | [0.58,1.04] | 0.084988 | 15.11493 | 0.635146 | [0.48,0.84] | 0.001399 | 0 |
| cereal type: biscuit cereal vs. any other | 29 | 0.893782 | [0.52,1.54] | 0.685598 | 4.62906 | 0.629701 | [0.28,1.4] | 0.258326 | 2.752859 | 0.707794 | [0.3,1.69] | 0.435515 | 6.993602 |
| cereal type: bran cereal vs. any other | 21 | 0.704101 | [0.33,1.52] | 0.370477 | 26.95924 | 0.74805 | [0.28,2] | 0.562909 | 1.225333 | 1.166832 | [0.35,3.86] | 0.800566 | 25.24245 |
| cereal type: oat cereal vs. any other | 26 | 0.862271 | [0.48,1.55] | 0.621012 | 32.79806 | 1.190871 | [0.49,2.87] | 0.697558 | 33.69303 | 0.633116 | [0.3,1.35] | 0.238493 | 0 |
| cereal type: muesli vs. any other | 86 | 0.651232 | [0.48,0.89] | 0.006924 | 21.00246 | 0.852133 | [0.52,1.41] | 0.533098 | 32.8777 | 0.583438 | [0.38,0.91] | 0.016187 | 1.282631 |
| cereal type: cornflakes/frosties vs. any other | 79 | 1.532325 | [1.14,2.05] | 0.00413 | 0 | 1.172775 | [0.76,1.81] | 0.472453 | 0 | 2.018482 | [1.27,3.2] | 0.002786 | 0 |
| coffee type: decaffeinated vs. any other | 150 | 0.835221 | [0.67,1.05] | 0.11995 | 24.9291 | 1.232569 | [0.92,1.66] | 0.165779 | 1.873827 | 0.712333 | [0.51,0.99] | 0.042116 | 10.12293 |
| coffee type: ground+instant vs. other+decaff | 35 | 0.663156 | [0.4,1.11] | 0.117594 | 21.16311 | 0.593946 | [0.3,1.18] | 0.135059 | 0.90562 | 0.744887 | [0.34,1.61] | 0.454991 | 13.04556 |
| coffee type: ground vs. any other | 29 | 1.284474 | [0.74,2.24] | 0.378942 | 14.58954 | 1.189547 | [0.49,2.87] | 0.699501 | 24.08078 | 1.223891 | [0.54,2.76] | 0.626266 | 0 |
| tablespoons of cooked vegetables per day | 132 | 0.968495 | [0.74,1.26] | 0.811354 | 19.45141 | 0.992762 | [0.66,1.5] | 0.972292 | 26.51574 | 0.601959 | [0.41,0.88] | 0.008496 | 3.093926 |
| tablespoons of raw vegetables per day | 146 | 0.724156 | [0.57,0.92] | 0.008282 | 11.42848 | 0.741767 | [0.53,1.04] | 0.081345 | 0 | 0.794709 | [0.55,1.15] | 0.223667 | 7.653294 |
| pieces of fresh fruit per day | 256 | 1.018117 | [0.86,1.2] | 0.834313 | 13.3974 | 1.064908 | [0.82,1.38] | 0.629692 | 16.75147 | 1.11011 | [0.86,1.43] | 0.418091 | 4.600467 |
| pieces of dried fruit per day | 119 | 0.943361 | [0.7,1.27] | 0.697746 | 31.56001 | 1.358765 | [0.88,2.09] | 0.163112 | 29.15353 | 0.870705 | [0.59,1.28] | 0.479714 | 0 |
| slices of bread per week | 133 | 1.049721 | [0.82,1.35] | 0.70671 | 15.99789 | 1.068872 | [0.75,1.53] | 0.716945 | 8.130004 | 1.121464 | [0.76,1.64] | 0.55737 | 8.941066 |
| bowls of cereal per week | 173 | 0.922683 | [0.73,1.16] | 0.495874 | 29.9909 | 0.956357 | [0.68,1.35] | 0.800929 | 30.75987 | 0.698508 | [0.51,0.95] | 0.022591 | 1.809544 |
| cups of tea per day | 179 | 0.829594 | [0.67,1.03] | 0.086807 | 26.26525 | 0.742602 | [0.56,0.99] | 0.041329 | 8.448692 | 0.971636 | [0.71,1.33] | 0.856059 | 13.20656 |
| cups of coffee per day | 139 | 0.71282 | [0.59,0.86] | 0.000354 | 5.573063 | 0.670274 | [0.5,0.9] | 0.006947 | 14.28244 | 0.790988 | [0.6,1.05] | 0.106475 | 0 |
| glasses of water per day | 262 | 1.176582 | [0.99,1.4] | 0.065969 | 14.78699 | 1.362364 | [1.06,1.75]* | 0.015755 | 9.785568 | 1.060995 | [0.81,1.39] | 0.664005 | 10.74636 |
| champagne/white wine glasses per month | 75 | 0.7847 | [0.58,1.06] | 0.116511 | 0 | 0.891847 | [0.57,1.4] | 0.619012 | 0 | 0.693677 | [0.43,1.12] | 0.133358 | 0 |
| red wine glasses per month | 135 | 0.648894 | [0.51,0.82] | 0.000337 | 16.0074 | 0.696796 | [0.5,0.97] | 0.032698 | 5.136513 | 0.542683 | [0.38,0.77] | 0.000593 | 3.937405 |
| beer/cider glasses per month | 115 | 0.764406 | [0.6,0.97] | 0.029027 | 12.82733 | 0.733436 | [0.51,1.05] | 0.09163 | 13.28487 | 0.736396 | [0.5,1.09] | 0.125547 | 17.74378 |
| spirits measures per month | 45 | 0.936803 | [0.63,1.39] | 0.747064 | 13.05454 | 1.110594 | [0.57,2.17] | 0.758552 | 32.12593 | 0.670009 | [0.36,1.23] | 0.198168 | 8.390546 |
| fortwine glasses per month | 15 | 1.619761 | [0.86,3.06] | 0.137679 | 0 | 1.888439 | [0.73,4.88] | 0.189516 | 0 | 1.642839 | [0.5,5.36] | 0.410446 | 27.85507 |
| other alcohol glasses per month | 11 | 0.593049 | [0.34,1.03] | 0.065635 | 0 | 0.817616 | [0.36,1.87] | 0.634325 | 0 | 0.236107 | [0.1,0.57] | 0.001276 | 0 |
| total drinks of alcohol per month | 281 | 0.752859 | [0.63,0.89] | 0.00114 | 21.72832 | 0.839928 | [0.66,1.07] | 0.155269 | 12.18277 | 0.642097 | [0.49,0.84] | 0.001037 | 18.68588 |
| overall oily fish intake | 192 | 0.72709 | [0.59,0.89] | 0.002614 | 20.73046 | 1.010889 | [0.74,1.37] | 0.94481 | 19.37299 | 0.65074 | [0.47,0.91] | 0.010708 | 21.94029 |
| overall non-oily fish intake | 77 | 1.021464 | [0.72,1.44] | 0.903546 | 19.17385 | 1.486209 | [0.94,2.35] | 0.091448 | 0 | 0.706132 | [0.43,1.15] | 0.161682 | 0 |
| overall processed meat intake | 107 | 1.087385 | [0.8,1.48] | 0.594119 | 19.56893 | 1.194543 | [0.78,1.84] | 0.417179 | 8.024673 | 0.985133 | [0.61,1.59] | 0.95089 | 16.33106 |
| overall poultry intake | 62 | 1.696493 | [1.19,2.43] | 0.003757 | 0 | 1.667493 | [0.94,2.97] | 0.081846 | 14.50719 | 1.156421 | [0.65,2.06] | 0.622805 | 5.131979 |
| overall beef intake | 107 | 1.08122 | [0.81,1.45] | 0.601065 | 14.60263 | 1.332783 | [0.86,2.06] | 0.195735 | 14.24753 | 1.1691 | [0.74,1.84] | 0.498847 | 11.22721 |
| overall lamb/mutton intake | 123 | 0.915567 | [0.7,1.21] | 0.529493 | 24.69563 | 1.070292 | [0.73,1.56] | 0.725824 | 12.39521 | 0.905671 | [0.61,1.34] | 0.622413 | 8.94342 |
| overall pork intake | 82 | 0.788737 | [0.56,1.11] | 0.175245 | 22.79492 | 0.778304 | [0.5,1.22] | 0.273973 | 0 | 1.034167 | [0.64,1.67] | 0.890933 | 1.94155 |
| overall cheese intake | 217 | 0.775472 | [0.63,0.95] | 0.01337 | 20.62137 | 0.797464 | [0.6,1.06] | 0.120889 | 12.60925 | 0.727365 | [0.54,0.99] | 0.040834 | 13.90225 |
| frequency of adding salt to food | 230 | 1.017626 | [0.83,1.24] | 0.864692 | 29.31025 | 0.862377 | [0.65,1.15] | 0.316414 | 24.47964 | 0.972086 | [0.73,1.3] | 0.849628 | 17.07596 |
| temperature of hot drinks | 184 | 0.967573 | [0.77,1.21] | 0.775643 | 26.90609 | 0.885092 | [0.63,1.24] | 0.48277 | 28.26475 | 1.130261 | [0.83,1.55] | 0.442698 | 4.327426 |
| overall alcohol intake | 282 | 0.740076 | [0.62,0.88] | 0.000665 | 21.00139 | 0.822248 | [0.65,1.04] | 0.104085 | 5.353818 | 0.674622 | [0.52,0.88] | 0.004079 | 18.1438 |
| among current drinkers, drinks usually with meals: yes, it varies, no | 135 | 0.801025 | [0.62,1.04] | 0.094036 | 31.5321 | 0.731853 | [0.53,1.01] | 0.059575 | 2.894271 | 0.854256 | [0.59,1.24] | 0.411747 | 18.81976 |
| milk type: skimmed, semi-skimmed, full cream (QT) | 54 | 0.792103 | [0.52,1.22] | 0.288625 | 24.79655 | 0.990903 | [0.56,1.75] | 0.974752 | 3.424883 | 0.888672 | [0.44,1.79] | 0.741535 | 29.49391 |

IVW: inverse variance weighted; OR: odd ratio; CI: confidence interval.

**Table S4. Associations between genetically predicted dietary habits and migraine in sensitivity analysis.**

| **Exposure** | **Weighted median** | | **Mode-based** | | **MR-Egger** | | | | **MRPRESSO** | | |
| --- | --- | --- | --- | --- | --- | --- | --- | --- | --- | --- | --- |
|  | **OR (95% CI)** | **P value** | **OR (95% CI)** | **P value** | **OR (95% CI)** | **P value** | **Intercept** | **P value** | **OR** | **P value** | **No. of outliers** |
| alcohol drinker status: current + former vs. never | 1.39(0.36,5.39) | 0.634361 | 0.7(0.19,2.63) | 0.599728 | 2.1(0.26,17.17) | 0.488424 | -0.01(-0.02,0.01) | 0.397441 | 0.94 | 0.898843 | 0 |
| alcohol drinker status: current vs. never | 1.42(0.39,5.19) | 0.599539 | 0.9(0.26,3.12) | 0.874401 | 4.35(0.5,38.16) | 0.184947 | -0.01(-0.03,0) | 0.167385 | 1.11 | 0.8403 | 0 |
| among current drinkers, drinks usually with meals: yes + it varies vs. no | 0.67(0.47,0.97) | 0.033068 | 0.76(0.5,1.15) | 0.191724 | 0.76(0.29,2.01) | 0.579396 | 0(-0.02,0.01) | 0.646905 | 0.61 | 0.000495 | 0 |
| among current drinkers, drinks usually with meals: yes vs. no | 0.83(0.67,1.04) | 0.103879 | 0.85(0.64,1.13) | 0.270454 | 0.92(0.51,1.67) | 0.784325 | 0(-0.01,0.01) | 0.728079 | 0.83 | 0.028338 | 1 |
| never eat eggs vs. no eggs, dairy, wheat, or sugar restrictions | 3.56(0.4,31.77) | 0.254929 | 4.18(0.46,37.96) | 0.203332 | 2.39(0.02,363.77) | 0.734553 | 0(-0.04,0.04) | 0.959298 | 2.12 | 0.469418 | 0 |
| never eat eggs vs. no eggs restrictions | 5.92(0.29,120.87) | 0.248221 | 9.79(0.5,190.44) | 0.131985 | 0.18(0,45.26) | 0.540371 | 0.02(-0.01,0.06) | 0.193294 | 5.11 | 0.161956 | 0 |
| never eat dairy vs. no eggs, dairy, wheat, or sugar restrictions | 0.74(0.12,4.75) | 0.750646 | 1.07(0.19,6.14) | 0.935277 | 0.05(0,1) | 0.050156 | 0.03(0,0.05) | 0.02239 | 1.13 | 0.879231 | 0 |
| never eat dairy vs. no dairy restrictions | 0.68(0.07,6.9) | 0.745419 | 1.06(0.12,9.04) | 0.960154 | 0.02(0,1) | 0.049703 | 0.03(0,0.05) | 0.021203 | 1.18 | 0.87129 | 0 |
| never eat wheat vs. no eggs, dairy, wheat, or sugar restrictions | 1.77(0.71,4.44) | 0.223675 | 1.72(0.82,3.59) | 0.149068 | 3.1(0.96,10.03) | 0.0592 | -0.02(-0.05,0) | 0.077978 | 1.3 | 0.413246 | 0 |
| never eat wheat vs. no wheat restrictions | 2.08(0.69,6.25) | 0.192685 | 2.08(0.84,5.14) | 0.112242 | 1.93(0.47,7.99) | 0.364458 | 0(-0.03,0.02) | 0.809731 | 1.67 | 0.214444 | 0 |
| never eat sugar vs. no eggs, dairy, wheat, or sugar restrictions | 1.03(0.71,1.52) | 0.861353 | 0.94(0.59,1.48) | 0.782357 | 1.3(0.47,3.63) | 0.613687 | 0(-0.01,0.01) | 0.743345 | 1.1 | 0.487247 | 0 |
| never eat sugar vs. no sugar restrictions | 0.76(0.52,1.13) | 0.18018 | 0.73(0.46,1.17) | 0.19425 | 0.86(0.33,2.26) | 0.756352 | 0(-0.01,0.01) | 0.746255 | 1 | 0.994812 | 1 |
| milk type: dairy-based milk vs. never | 0.3(0.05,1.98) | 0.213318 | 0.25(0.04,1.76) | 0.164887 | 0.9(0,173.6) | 0.968073 | 0(-0.04,0.04) | 0.972522 | 0.39 | 0.21092 | 1 |
| milk type: any milk vs. never | 0.27(0.05,1.6) | 0.150141 | 0.21(0.03,1.27) | 0.088541 | 0.01(0,0.36) | 0.012044 | 0.04(0.01,0.07) | 0.022226 | 0.24 | 0.043088 | 1 |
| milk type: full cream vs. never | 1.03(0.83,1.29) | 0.77953 | 1.04(0.82,1.32) | 0.722912 | 1(0.65,1.56) | 0.986313 | 0(-0.02,0.03) | 0.701601 | 1.09 | 0.234611 | 0 |
| milk type: full cream vs. any other | 0.94(0.35,2.53) | 0.903927 | 0.9(0.33,2.49) | 0.841525 | 2.24(0.22,23.19) | 0.499934 | -0.01(-0.03,0.01) | 0.468789 | 0.98 | 0.949233 | 0 |
| milk type: semi-skimmed vs. never | 0.54(0.14,2.05) | 0.364031 | 0.38(0.1,1.43) | 0.151914 | 0.41(0.02,10.25) | 0.590256 | 0.01(-0.02,0.05) | 0.490534 | 1.21 | 0.716983 | 0 |
| milk type: semi-skimmed vs. any other | 0.63(0.34,1.19) | 0.154186 | 0.65(0.37,1.14) | 0.131676 | 0.74(0.31,1.79) | 0.509002 | 0(-0.01,0.02) | 0.917851 | 0.77 | 0.15458 | 0 |
| milk type: skimmed vs. never | 0.97(0.57,1.64) | 0.902148 | 0.8(0.47,1.37) | 0.421412 | 0.62(0.22,1.77) | 0.372929 | 0.02(-0.01,0.04) | 0.270751 | 1.06 | 0.776904 | 1 |
| milk type: skimmed vs. any other | 0.91(0.49,1.7) | 0.768377 | 0.85(0.46,1.56) | 0.601506 | 0.66(0.19,2.25) | 0.504965 | 0.01(-0.01,0.02) | 0.412889 | 1.06 | 0.795352 | 0 |
| milk type: soy milk vs. never | 1.11(0.91,1.35) | 0.297109 | 1.1(0.9,1.33) | 0.34782 | 1.61(1.05,2.46) | 0.030013 | -0.03(-0.05,0) | 0.047517 | 1.07 | 0.359006 | 0 |
| milk type: soy milk vs. any other | 0.32(0.06,1.56) | 0.157494 | 0.54(0.12,2.57) | 0.443147 | 0.62(0.02,17.76) | 0.777939 | 0(-0.02,0.03) | 0.892046 | 0.77 | 0.630547 | 0 |
| milk type: other milk vs. never | 0.9(0.7,1.15) | 0.394082 | 0.91(0.71,1.16) | 0.432651 | 0.96(0.62,1.46) | 0.83461 | 0(-0.04,0.03) | 0.802812 | 0.91 | 0.340598 | 0 |
| milk type: other milk vs. any other | 0.42(0.01,16.48) | 0.645635 | 0.32(0.01,10.25) | 0.522393 | 0.87(0,2832.19) | 0.973478 | 0(-0.06,0.05) | 0.983246 | 0.81 | 0.89987 | 1 |
| spread type: all spreads vs. never | 0.54(0.27,1.09) | 0.084411 | 0.51(0.25,1.02) | 0.056813 | 0.74(0.23,2.4) | 0.61977 | 0(-0.01,0.01) | 0.97728 | 0.73 | 0.218911 | 0 |
| spread type: butter + margarine vs. never | 0.69(0.44,1.09) | 0.114725 | 0.68(0.43,1.07) | 0.098396 | 0.84(0.39,1.83) | 0.660823 | 0(-0.01,0.02) | 0.725369 | 0.95 | 0.771228 | 0 |
| spread type: any oil based spread vs. never | 1.41(0.91,2.18) | 0.122944 | 1.3(0.85,1.97) | 0.225043 | 1.04(0.44,2.46) | 0.927646 | 0(-0.02,0.02) | 0.759048 | 1.18 | 0.302964 | 0 |
| spread type: butter and butter-like spreads vs. oil-based spreads | 0.88(0.55,1.43) | 0.612956 | 1(0.64,1.56) | 0.995082 | 1.03(0.5,2.12) | 0.939896 | 0.01(-0.01,0.02) | 0.424922 | 1.34 | 0.076306 | 0 |
| spread type: butter and margarine spreads vs. oil-based spreads | 1.15(0.77,1.72) | 0.491007 | 1.09(0.72,1.65) | 0.695928 | 1.17(0.51,2.7) | 0.707272 | 0(-0.02,0.01) | 0.979424 | 1.16 | 0.314178 | 0 |
| spread type: butter vs. never | 0.84(0.59,1.22) | 0.362621 | 0.77(0.54,1.11) | 0.159804 | 0.89(0.46,1.72) | 0.733457 | 0(-0.01,0.01) | 0.922315 | 0.92 | 0.513552 | 0 |
| spread type: butter vs. any other | 0.98(0.68,1.43) | 0.922163 | 0.98(0.63,1.52) | 0.926152 | 2.55(1.05,6.2) | 0.038312 | -0.01(-0.03,0) | 0.051856 | 1.1 | 0.476901 | 0 |
| spread type: tub margarine vs. never | 0.79(0.59,1.06) | 0.113279 | 0.84(0.64,1.11) | 0.222145 | 0.78(0.47,1.28) | 0.322978 | 0.01(-0.01,0.03) | 0.593218 | 0.88 | 0.254646 | 0 |
| spread type: tub margarine vs. any other | 0.56(0.17,1.88) | 0.346589 | 0.9(0.29,2.86) | 0.862355 | 2.27(0.19,26.43) | 0.513005 | -0.01(-0.03,0.02) | 0.481799 | 1.01 | 0.981436 | 0 |
| spread type: flora + benecol vs. never | 1.1(0.82,1.49) | 0.526794 | 1.07(0.81,1.41) | 0.625907 | 0.88(0.57,1.34) | 0.541049 | 0(-0.01,0.02) | 0.602068 | 0.97 | 0.719831 | 0 |
| spread type: flora + benecol vs. any other | 1.04(0.43,2.48) | 0.935109 | 1.14(0.51,2.55) | 0.742219 | 0.82(0.22,3.11) | 0.773975 | 0(-0.01,0.02) | 0.795763 | 0.96 | 0.900255 | 0 |
| spread type: olive oil spread vs. never | 0.84(0.6,1.19) | 0.325521 | 0.98(0.7,1.37) | 0.900728 | 1.14(0.61,2.15) | 0.680177 | -0.01(-0.03,0.01) | 0.454346 | 0.92 | 0.494102 | 0 |
| spread type: olive oil spread vs. any other | 0.64(0.24,1.7) | 0.369136 | 0.67(0.26,1.73) | 0.407487 | 0.63(0.16,2.46) | 0.506154 | 0(-0.02,0.02) | 0.817904 | 0.55 | 0.063836 | 0 |
| spread type: other oil-based spread vs. never | 1.33(0.95,1.88) | 0.099242 | 1.34(0.97,1.85) | 0.078278 | 1.78(1.02,3.1) | 0.040993 | -0.01(-0.03,0.01) | 0.260787 | 1.33 | 0.014306 | 0 |
| spread type: other oil-based spread vs. any other | 0.93(0.39,2.25) | 0.878147 | 0.89(0.41,1.96) | 0.775046 | 0.93(0.21,4.14) | 0.924383 | 0(-0.02,0.02) | 0.941682 | 0.98 | 0.946233 | 0 |
| spread type: low fat spread vs. never | 0.87(0.6,1.26) | 0.451477 | 0.98(0.72,1.34) | 0.915966 | 0.69(0.41,1.17) | 0.169429 | 0.03(0,0.05) | 0.054476 | 1.07 | 0.682185 | 0 |
| spread type: low fat spread vs. any other | 0.33(0.06,1.76) | 0.194203 | 0.4(0.08,1.92) | 0.252219 | 0.23(0.01,4.56) | 0.336881 | 0.01(-0.02,0.04) | 0.694252 | 0.39 | 0.233417 | 1 |
| bread type: white vs. any other | 1.43(1.06,1.93) | 0.018597 | 1.44(0.97,2.14) | 0.073028 | 1.29(0.56,3.01) | 0.550137 | 0(-0.01,0.01) | 0.708417 | 1.51 | 0.000257 | 0 |
| bread type: brown vs. any other | 1.13(0.42,3.03) | 0.805229 | 1.14(0.46,2.82) | 0.782995 | 0.68(0.09,4.88) | 0.700208 | 0(-0.02,0.03) | 0.792444 | 0.87 | 0.66433 | 0 |
| bread type: wholemeal/wholegrain vs. any other | 0.73(0.55,0.96) | 0.025915 | 0.69(0.48,0.98) | 0.040069 | 0.63(0.3,1.31) | 0.215244 | 0(-0.01,0.02) | 0.468623 | 0.82 | 0.055359 | 0 |
| bread type: white vs. wholemeal/wholegrain + brown | 1.41(1.07,1.87) | 0.015453 | 1.47(1.01,2.13) | 0.044121 | 1.42(0.7,2.89) | 0.333664 | 0(-0.01,0.01) | 0.984851 | 1.43 | 0.00037 | 0 |
| bread type: wholemeal/wholegrain vs. white + brown | 0.68(0.52,0.89) | 0.005243 | 0.67(0.48,0.94) | 0.019489 | 0.94(0.48,1.84) | 0.852943 | 0(-0.01,0.01) | 0.522763 | 0.76 | 0.00499 | 0 |
| cereal type: biscuit cereal vs. any other | 0.8(0.38,1.72) | 0.576764 | 0.88(0.43,1.79) | 0.715863 | 0.71(0.16,3.21) | 0.657946 | 0(-0.02,0.03) | 0.749795 | 0.89 | 0.688675 | 0 |
| cereal type: bran cereal vs. any other | 1.05(0.39,2.84) | 0.927854 | 0.85(0.33,2.16) | 0.731563 | 0.34(0.04,2.87) | 0.319342 | 0.01(-0.02,0.04) | 0.468919 | 0.7 | 0.38113 | 0 |
| cereal type: oat cereal vs. any other | 1.17(0.57,2.42) | 0.666647 | 1.11(0.54,2.26) | 0.779141 | 0.79(0.19,3.37) | 0.750779 | 0(-0.02,0.03) | 0.897434 | 0.86 | 0.625328 | 0 |
| cereal type: muesli vs. any other | 0.63(0.42,0.95) | 0.0278 | 0.61(0.38,0.98) | 0.04026 | 0.66(0.19,2.34) | 0.52254 | 0(-0.02,0.02) | 0.978183 | 0.65 | 0.008355 | 0 |
| cereal type: cornflakes/frosties vs. any other | 1.72(1.12,2.65) | 0.013023 | 1.76(1.08,2.86) | 0.022224 | 1.55(0.63,3.81) | 0.340219 | 0(-0.01,0.01) | 0.980446 | 1.53 | 0.004057 | 0 |
| coffee type: decaffeinated vs. any other | 0.77(0.57,1.03) | 0.078929 | 0.71(0.49,1.04) | 0.081074 | 1.16(0.48,2.79) | 0.747521 | 0(-0.02,0.01) | 0.454325 | 0.84 | 0.122072 | 1 |
| coffee type: ground+instant vs. other+decaff | 0.61(0.31,1.23) | 0.169405 | 0.74(0.36,1.51) | 0.404438 | 1.4(0.35,5.59) | 0.629533 | -0.01(-0.04,0.01) | 0.251346 | 0.66 | 0.126855 | 0 |
| coffee type: ground vs. any other | 1.48(0.68,3.2) | 0.31892 | 1.08(0.51,2.3) | 0.839701 | 1.11(0.24,5.02) | 0.896624 | 0(-0.02,0.03) | 0.834001 | 1.28 | 0.386431 | 0 |
| tablespoons of cooked vegetables per day | 0.89(0.62,1.27) | 0.507284 | 0.86(0.55,1.34) | 0.513291 | 1.09(0.38,3.11) | 0.877134 | 0(-0.02,0.01) | 0.824766 | 0.97 | 0.811726 | 1 |
| tablespoons of raw vegetables per day | 0.62(0.44,0.87) | 0.005358 | 0.63(0.41,0.96) | 0.030952 | 0.4(0.17,0.93) | 0.032585 | 0.01(0,0.02) | 0.147091 | 0.72 | 0.009189 | 0 |
| pieces of fresh fruit per day | 1.06(0.83,1.35) | 0.659055 | 1.09(0.8,1.49) | 0.587905 | 0.72(0.4,1.32) | 0.289531 | 0(0,0.01) | 0.243859 | 1.02 | 0.83448 | 1 |
| pieces of dried fruit per day | 1.14(0.78,1.68) | 0.491127 | 1.25(0.8,1.93) | 0.325136 | 1.98(0.7,5.58) | 0.198268 | -0.01(-0.02,0) | 0.145487 | 0.94 | 0.698446 | 1 |
| slices of bread per week | 1(0.7,1.43) | 0.993875 | 1.09(0.72,1.67) | 0.680304 | 2.07(0.87,4.92) | 0.097961 | -0.01(-0.02,0) | 0.106417 | 1.05 | 0.707315 | 0 |
| bowls of cereal per week | 1.1(0.82,1.5) | 0.518895 | 1.02(0.7,1.47) | 0.935038 | 1.66(0.75,3.68) | 0.208106 | -0.01(-0.02,0) | 0.127588 | 0.92 | 0.452756 | 2 |
| cups of tea per day | 0.65(0.47,0.91) | 0.010719 | 0.66(0.48,0.9) | 0.008797 | 0.47(0.27,0.83) | 0.008343 | 0.01(0,0.02) | 0.032428 | 0.85 | 0.132662 | 1 |
| cups of coffee per day | 0.61(0.45,0.83) | 0.001745 | 0.66(0.51,0.85) | 0.001642 | 0.6(0.42,0.87) | 0.007462 | 0(0,0.01) | 0.303872 | 0.71 | 0.000488 | 0 |
| glasses of water per day | 1.31(1.01,1.7) | 0.041608 | 1.47(1.1,1.97) | 0.010037 | 1.88(1.11,3.2) | 0.019828 | -0.01(-0.01,0) | 0.067456 | 1.18 | 0.067105 | 1 |
| champagne/white wine glasses per month | 0.79(0.52,1.22) | 0.291911 | 0.66(0.4,1.08) | 0.101138 | 0.6(0.24,1.49) | 0.273903 | 0(-0.01,0.02) | 0.545867 | 0.78 | 0.108221 | 0 |
| red wine glasses per month | 0.68(0.49,0.94) | 0.021017 | 0.72(0.49,1.05) | 0.086263 | 1.13(0.52,2.48) | 0.758187 | -0.01(-0.02,0) | 0.146075 | 0.65 | 0.000471 | 0 |
| beer/cider glasses per month | 0.68(0.49,0.96) | 0.026234 | 0.75(0.51,1.1) | 0.136127 | 1.02(0.51,2.04) | 0.964876 | 0(-0.01,0.01) | 0.393657 | 0.76 | 0.031076 | 0 |
| spirits measures per month | 1.28(0.73,2.25) | 0.381066 | 1.04(0.61,1.8) | 0.874531 | 2.22(1,4.94) | 0.05106 | -0.02(-0.03,0) | 0.016718 | 0.94 | 0.748592 | 0 |
| fortwine glasses per month | 0.89(0.36,2.17) | 0.792515 | 1.14(0.49,2.61) | 0.762403 | 1.12(0.22,5.55) | 0.892252 | 0.01(-0.02,0.03) | 0.620622 | 1.62 | 0.111704 | 0 |
| other alcohol glasses per month | 0.56(0.26,1.23) | 0.149431 | 0.63(0.3,1.3) | 0.209462 | 0.6(0.13,2.7) | 0.505529 | 0(-0.04,0.04) | 0.986538 | 0.59 | 0.085519 | 0 |
| total drinks of alcohol per month | 0.8(0.62,1.03) | 0.080606 | 0.8(0.61,1.07) | 0.131109 | 0.72(0.45,1.15) | 0.165964 | 0(-0.01,0.01) | 0.821843 | 0.78 | 0.002822 | 1 |
| overall oily fish intake | 0.71(0.54,0.95) | 0.019276 | 0.68(0.48,0.95) | 0.023804 | 0.31(0.14,0.71) | 0.005541 | 0.01(0,0.02) | 0.037772 | 0.73 | 0.002967 | 1 |
| overall non-oily fish intake | 0.71(0.44,1.13) | 0.147964 | 0.73(0.43,1.23) | 0.237206 | 0.96(0.31,2.92) | 0.936821 | 0(-0.01,0.02) | 0.902468 | 1.02 | 0.903866 | 0 |
| overall processed meat intake | 0.93(0.61,1.39) | 0.710496 | 0.88(0.53,1.47) | 0.630382 | 1.6(0.53,4.81) | 0.401898 | -0.01(-0.02,0.01) | 0.472876 | 1.03 | 0.861092 | 1 |
| overall poultry intake | 1.89(1.14,3.15) | 0.014093 | 1.69(0.97,2.96) | 0.06519 | 0.77(0.27,2.24) | 0.633042 | 0.01(0,0.02) | 0.12326 | 1.7 | 0.004377 | 0 |
| overall beef intake | 1.38(0.92,2.08) | 0.118669 | 1.28(0.81,2.02) | 0.288552 | 1.61(0.64,4.05) | 0.311461 | -0.01(-0.02,0.01) | 0.372136 | 1.08 | 0.602156 | 0 |
| overall lamb/mutton intake | 0.9(0.62,1.32) | 0.602048 | 0.74(0.48,1.12) | 0.150035 | 0.68(0.28,1.67) | 0.40113 | 0(-0.01,0.02) | 0.496637 | 0.88 | 0.325622 | 1 |
| overall pork intake | 0.81(0.52,1.27) | 0.364737 | 0.71(0.45,1.12) | 0.142254 | 1.48(0.57,3.83) | 0.421216 | -0.01(-0.02,0) | 0.1663 | 0.76 | 0.066638 | 2 |
| overall cheese intake | 0.72(0.55,0.95) | 0.020874 | 0.62(0.43,0.91) | 0.013165 | 0.97(0.5,1.89) | 0.929865 | 0(-0.01,0.01) | 0.487502 | 0.78 | 0.01414 | 1 |
| frequency of adding salt to food | 1.11(0.85,1.45) | 0.435395 | 1.21(0.86,1.69) | 0.273927 | 0.83(0.41,1.65) | 0.586591 | 0(-0.01,0.01) | 0.53513 | 0.99 | 0.937403 | 1 |
| temperature of hot drinks | 1.02(0.75,1.38) | 0.905498 | 1.1(0.74,1.63) | 0.642847 | 0.51(0.23,1.13) | 0.097551 | 0.01(0,0.02) | 0.100325 | 1 | 0.985556 | 1 |
| overall alcohol intake | 0.77(0.6,0.98) | 0.034203 | 0.81(0.61,1.07) | 0.141526 | 0.92(0.54,1.57) | 0.75712 | 0(-0.01,0) | 0.400798 | 0.76 | 0.001558 | 1 |
| among current drinkers, drinks usually with meals: yes, it varies, no | 0.83(0.6,1.16) | 0.271112 | 0.83(0.56,1.23) | 0.351992 | 0.85(0.33,2.21) | 0.742654 | 0(-0.01,0.01) | 0.893469 | 0.8 | 0.096368 | 1 |
| milk type: skimmed, semi-skimmed, full cream (QT) | 0.97(0.55,1.71) | 0.911684 | 1.03(0.58,1.82) | 0.912204 | 1.1(0.27,4.48) | 0.897942 | 0(-0.02,0.01) | 0.634249 | 0.79 | 0.293436 | 0 |

MR: mendelian randomization; MRPRESSO: MR pleiotropy residual sum and outlier; OR: odd ratio; CI: confidence interval; No.: number.

**Table S5. Leave-one-out analysis of association between genetically predicted dietary habits and migraine.**

| **Outcome** | **IVW Estimate**  **[Min, Max] ^a^** | **P value [Min, Max] ^b^** |
| --- | --- | --- |
| alcohol drinker status: current + former vs. never | [0.77,1.16] | [0.572302889412635,0.998587076536983] |
| alcohol drinker status: current vs. never | [0.91,1.35] | [0.522818925888246,0.99548573168037] |
| among current drinkers, drinks usually with meals: yes + it varies vs. no | [0.59,0.63] | [8.40268016483851e-05,0.000649696349049789] |
| among current drinkers, drinks usually with meals: yes vs. no | [0.82,0.85] | [0.0145646984529173,0.0463652780069477] |
| never eat eggs vs. no eggs, dairy, wheat, or sugar restrictions | [1.32,4.25] | [0.0817300258307891,0.770026626300354] |
| never eat eggs vs. no eggs restrictions | [3.12,14.19] | [0.0337511244006873,0.351758800162638] |
| never eat dairy vs. no eggs, dairy, wheat, or sugar restrictions | [0.59,1.42] | [0.475603926672982,0.996135010724163] |
| never eat dairy vs. no dairy restrictions | [0.53,1.55] | [0.495894055202525,0.999005083533332] |
| never eat wheat vs. no eggs, dairy, wheat, or sugar restrictions | [1.18,1.4] | [0.326119363192809,0.683664238110303] |
| never eat wheat vs. no wheat restrictions | [1.54,1.84] | [0.140971888813276,0.324708109711489] |
| never eat sugar vs. no eggs, dairy, wheat, or sugar restrictions | [1.07,1.15] | [0.327338340426796,0.647397157860649] |
| never eat sugar vs. no sugar restrictions | [0.96,1.06] | [0.679708633689041,0.998373986577733] |
| milk type: dairy-based milk vs. never | [0.39,1.15] | [0.193568222609204,0.999712629302309] |
| milk type: any milk vs. never | [0.17,0.49] | [0.00712551252819845,0.336375527191765] |
| milk type: full cream vs. never | [1.05,1.12] | [0.184681934520593,0.530179844098156] |
| milk type: full cream vs. any other | [0.85,1.11] | [0.655868566540638,0.997393560589713] |
| milk type: semi-skimmed vs. never | [0.76,1.4] | [0.498419886734319,0.961443457206407] |
| milk type: semi-skimmed vs. any other | [0.71,0.82] | [0.114573178988164,0.411198081366541] |
| milk type: skimmed vs. never | [0.89,1.18] | [0.440540225324426,0.988797969013357] |
| milk type: skimmed vs. any other | [0.97,1.17] | [0.514768496727791,0.968451478012202] |
| milk type: soy milk vs. never | [1.06,1.12] | [0.131915022399112,0.463997623650687] |
| milk type: soy milk vs. any other | [0.61,0.99] | [0.404330514884836,0.981468374132896] |
| milk type: other milk vs. never | [0.84,1] | [0.0635834982465771,0.959828945813002] |
| milk type: other milk vs. any other | [0.31,3.05] | [0.456779909507669,0.986595069443344] |
| spread type: all spreads vs. never | [0.65,0.81] | [0.0851505594744124,0.433562803715827] |
| spread type: butter + margarine vs. never | [0.89,1.04] | [0.474241203959351,0.943369089081711] |
| spread type: any oil based spread vs. never | [1.11,1.24] | [0.166200877060627,0.468360461919855] |
| spread type: butter and butter-like spreads vs. oil-based spreads | [1.28,1.44] | [0.0375963710051023,0.128145245628229] |
| spread type: butter and margarine spreads vs. oil-based spreads | [1.12,1.23] | [0.143841660015786,0.439033731739277] |
| spread type: butter vs. never | [0.87,0.97] | [0.272487496986814,0.842193529138976] |
| spread type: butter vs. any other | [1.06,1.14] | [0.320917492191722,0.650583388044046] |
| spread type: tub margarine vs. never | [0.85,0.94] | [0.12282226043442,0.617981768638358] |
| spread type: tub margarine vs. any other | [0.82,1.19] | [0.676901065844015,0.999301629638956] |
| spread type: flora + benecol vs. never | [0.93,1] | [0.510797975467716,0.994274567261039] |
| spread type: flora + benecol vs. any other | [0.87,1.1] | [0.631177658759944,0.998890759596286] |
| spread type: olive oil spread vs. never | [0.87,0.97] | [0.261733045040208,0.798575254648989] |
| spread type: olive oil spread vs. any other | [0.48,0.72] | [0.0493456228601791,0.417851073038816] |
| spread type: other oil-based spread vs. never | [1.28,1.38] | [0.00461236425051297,0.0304236143808349] |
| spread type: other oil-based spread vs. any other | [0.86,1.15] | [0.621576037211181,0.986204684450601] |
| spread type: low fat spread vs. never | [0.99,1.24] | [0.253460905495619,0.933355516407405] |
| spread type: low fat spread vs. any other | [0.29,0.59] | [0.0812509484469371,0.455238101997957] |
| bread type: white vs. any other | [1.45,1.56] | [5.34753103284319e-05,0.000641488373976904] |
| bread type: brown vs. any other | [0.78,0.97] | [0.515322478875512,0.940454070253523] |
| bread type: wholemeal/wholegrain vs. any other | [0.8,0.84] | [0.0269371447995394,0.0876529543273323] |
| bread type: white vs. wholemeal/wholegrain + brown | [1.38,1.47] | [8.10254140221136e-05,0.00097545121110619] |
| bread type: wholemeal/wholegrain vs. white + brown | [0.74,0.78] | [0.00180617832881925,0.00839671407651623] |
| cereal type: biscuit cereal vs. any other | [0.82,1.01] | [0.484617288651161,0.982926538858672] |
| cereal type: bran cereal vs. any other | [0.59,0.87] | [0.15835427887546,0.711536695933719] |
| cereal type: oat cereal vs. any other | [0.73,0.94] | [0.2789485909054,0.836759058984097] |
| cereal type: muesli vs. any other | [0.63,0.68] | [0.00305400397745845,0.012331834371528] |
| cereal type: cornflakes/frosties vs. any other | [1.46,1.6] | [0.00173063357338507,0.0115495491980933] |
| coffee type: decaffeinated vs. any other | [0.81,0.86] | [0.0652149697809257,0.185045422990051] |
| coffee type: ground+instant vs. other+decaff | [0.57,0.75] | [0.0333223595554211,0.239487161471007] |
| coffee type: ground vs. any other | [1.12,1.53] | [0.139611997028206,0.67158679485865] |
| tablespoons of cooked vegetables per day | [0.94,1] | [0.656583188277203,0.980746187906556] |
| tablespoons of raw vegetables per day | [0.71,0.75] | [0.00383708366770014,0.0183094219002069] |
| pieces of fresh fruit per day | [1,1.03] | [0.702845450834671,0.977549354436099] |
| pieces of dried fruit per day | [0.9,0.98] | [0.490805662374729,0.900456540727676] |
| slices of bread per week | [1.02,1.1] | [0.432611032770273,0.847972079608643] |
| bowls of cereal per week | [0.9,0.94] | [0.359867965371961,0.615613289623142] |
| cups of tea per day | [0.82,0.88] | [0.076156054300995,0.269464117432081] |
| cups of coffee per day | [0.7,0.74] | [0.00013861131978491,0.0021225285780141] |
| glasses of water per day | [1.14,1.2] | [0.0376549012363835,0.137513530779512] |
| champagne/white wine glasses per month | [0.75,0.81] | [0.0623920426129087,0.176617073798111] |
| red wine glasses per month | [0.62,0.67] | [9.75281056035896e-05,0.000591971998206435] |
| beer/cider glasses per month | [0.74,0.79] | [0.0120133540965234,0.0501331702166257] |
| spirits measures per month | [0.76,0.85] | [0.178557506087645,0.419397157189675] |
| fortwine glasses per month | [1.45,1.93] | [0.0684522333138106,0.268229153377299] |
| other alcohol glasses per month | [0.5,0.67] | [0.0208941137685995,0.179307927994784] |
| total drinks of alcohol per month | [0.74,0.78] | [0.000636149210822573,0.00258467442214923] |
| overall oily fish intake | [0.72,0.75] | [0.00204775586948038,0.00662758756125554] |
| overall non-oily fish intake | [0.97,1.1] | [0.551348363178106,0.992358911697931] |
| overall processed meat intake | [1.03,1.13] | [0.433092524617272,0.860754278942276] |
| overall poultry intake | [1.61,1.81] | [0.00127590483036908,0.00947047359146675] |
| overall beef intake | [1.04,1.13] | [0.390288967460472,0.768795411735371] |
| overall lamb/mutton intake | [0.88,0.95] | [0.323652690408542,0.710627145537191] |
| overall pork intake | [0.72,0.84] | [0.035724518799195,0.279412977817441] |
| overall cheese intake | [0.74,0.79] | [0.00422898185419162,0.0217923946246971] |
| frequency of adding salt to food | [0.99,1.04] | [0.683108665827343,0.995308100601978] |
| temperature of hot drinks | [0.95,1] | [0.631678352577931,0.985536049204391] |
| overall alcohol intake | [0.73,0.76] | [0.000337279623149066,0.00139773454753532] |
| among current drinkers, drinks usually with meals: yes, it varies, no | [0.77,0.83] | [0.0479195923178256,0.145949667271649] |
| milk type: skimmed, semi-skimmed, full cream (QT) | [0.74,0.85] | [0.188003368093369,0.439654669404489] |

**^a^** the minimum value and maximum value of inverse variance weighted estimate;

**^b^** the minimum value and maximum value of P value;

IVW: inverse variance weighted.

**Table S6. Associations between genetically predicted dietary habits and migraine with aura in sensitivity analysis.**

| **Exposure** | **Weighted median** | | **Mode-based** | | **MR-Egger** | | | | **MRPRESSO** | | |
| --- | --- | --- | --- | --- | --- | --- | --- | --- | --- | --- | --- |
|  | **OR (95% CI)** | **P value** | **OR (95% CI)** | **P value** | **OR (95% CI)** | **P value** | **Intercept** | **P value** | **OR** | **P value** | **No. of outliers** |
| alcohol drinker status: current + former vs. never | 1(0.15,6.61) | 0.996068 | 1.44(0.23,9.21) | 0.697827 | 0.9(0.05,15.28) | 0.941705 | 0(-0.02,0.02) | 0.799798 | 1.25 | 0.737023 | 0 |
| alcohol drinker status: current vs. never | 1.13(0.18,7.15) | 0.89558 | 1.73(0.3,9.99) | 0.539607 | 2.22(0.13,37.88) | 0.582537 | 0(-0.02,0.02) | 0.823898 | 1.66 | 0.433692 | 0 |
| among current drinkers, drinks usually with meals: yes + it varies vs. no | 0.71(0.41,1.23) | 0.216214 | 0.76(0.39,1.5) | 0.436751 | 0.56(0.14,2.28) | 0.414567 | 0(-0.02,0.02) | 0.916784 | 0.6 | 0.010905 | 0 |
| among current drinkers, drinks usually with meals: yes vs. no | 0.8(0.57,1.1) | 0.16612 | 0.89(0.58,1.38) | 0.613311 | 0.78(0.35,1.72) | 0.53346 | 0(-0.01,0.02) | 0.797233 | 0.86 | 0.169821 | 0 |
| never eat eggs vs. no eggs, dairy, wheat, or sugar restrictions | 1.57(0.06,41.94) | 0.788333 | 2.78(0.11,73.34) | 0.54046 | 27.27(0.03,27710.6) | 0.349384 | -0.03(-0.08,0.03) | 0.317363 | 1.09 | 0.95525 | 0 |
| never eat eggs vs. no eggs restrictions | 2.24(0.03,158.61) | 0.709888 | 3.6(0.06,212.07) | 0.538081 | 0.05(0,179.85) | 0.466885 | 0.03(-0.03,0.08) | 0.365028 | 1.52 | 0.68416 | 0 |
| never eat dairy vs. no eggs, dairy, wheat, or sugar restrictions | 0.42(0.03,6.55) | 0.535424 | 0.83(0.06,10.74) | 0.889313 | 0.03(0,3.88) | 0.162359 | 0.03(-0.01,0.07) | 0.114075 | 1.03 | 0.978238 | 0 |
| never eat dairy vs. no dairy restrictions | 0.32(0.01,9.69) | 0.515766 | 0.75(0.03,17.15) | 0.858216 | 0.02(0,4.93) | 0.155989 | 0.03(-0.01,0.07) | 0.107923 | 1.03 | 0.982683 | 0 |
| never eat wheat vs. no eggs, dairy, wheat, or sugar restrictions | 2.4(0.64,9.02) | 0.196141 | 2.36(0.8,6.98) | 0.119464 | 7.59(1.32,43.78) | 0.023411 | -0.04(-0.08,-0.01) | 0.021929 | 1.41 | 0.467107 | 0 |
| never eat wheat vs. no wheat restrictions | 3.07(0.61,15.34) | 0.172542 | 2.87(0.75,11) | 0.124904 | 6.77(0.82,55.58) | 0.075053 | -0.03(-0.06,0.01) | 0.135016 | 1.8 | 0.34184 | 0 |
| never eat sugar vs. no eggs, dairy, wheat, or sugar restrictions | 1.2(0.67,2.16) | 0.546871 | 1.21(0.57,2.55) | 0.61904 | 1.63(0.32,8.37) | 0.556818 | -0.01(-0.03,0.01) | 0.395225 | 0.83 | 0.401295 | 1 |
| never eat sugar vs. no sugar restrictions | 0.9(0.49,1.64) | 0.728307 | 0.95(0.45,1.99) | 0.885314 | 1.27(0.28,5.78) | 0.761405 | -0.01(-0.03,0.01) | 0.545603 | 0.81 | 0.370745 | 1 |
| milk type: dairy-based milk vs. never | 2.23(0.16,31.51) | 0.551971 | 2.48(0.17,35.27) | 0.501662 | 1.64(0,719.87) | 0.873687 | 0(-0.04,0.05) | 0.962623 | 1.88 | 0.502736 | 0 |
| milk type: any milk vs. never | 0.52(0.04,6.75) | 0.615382 | 0.55(0.04,6.85) | 0.640936 | 0.06(0,7.64) | 0.254529 | 0.02(-0.02,0.06) | 0.256457 | 0.81 | 0.796412 | 0 |
| milk type: full cream vs. never | 1(0.71,1.42) | 0.983524 | 1.06(0.73,1.54) | 0.759773 | 1.2(0.59,2.42) | 0.613073 | -0.01(-0.04,0.03) | 0.736857 | 1.07 | 0.592319 | 0 |
| milk type: full cream vs. any other | 0.53(0.12,2.38) | 0.405833 | 0.99(0.21,4.59) | 0.987744 | 1.16(0.04,37.94) | 0.933497 | 0(-0.04,0.03) | 0.754865 | 0.68 | 0.44375 | 0 |
| milk type: semi-skimmed vs. never | 3.37(0.55,20.78) | 0.190144 | 2.72(0.47,15.81) | 0.264869 | 0.23(0,16.08) | 0.498176 | 0.03(-0.02,0.08) | 0.18975 | 3.41 | 0.023881 | 0 |
| milk type: semi-skimmed vs. any other | 1.07(0.41,2.79) | 0.894337 | 1.21(0.53,2.76) | 0.650717 | 1.49(0.4,5.54) | 0.548144 | -0.01(-0.03,0.01) | 0.490637 | 1 | 0.986871 | 0 |
| milk type: skimmed vs. never | 1.02(0.49,2.12) | 0.961421 | 1.02(0.49,2.13) | 0.947461 | 0.44(0.13,1.52) | 0.194721 | 0.02(-0.01,0.06) | 0.148432 | 1.02 | 0.948769 | 0 |
| milk type: skimmed vs. any other | 0.77(0.3,1.98) | 0.593309 | 0.87(0.33,2.29) | 0.778339 | 0.18(0.03,1.03) | 0.053501 | 0.02(0,0.05) | 0.050368 | 0.91 | 0.766112 | 0 |
| milk type: soy milk vs. never | 1.09(0.81,1.46) | 0.577026 | 1.1(0.82,1.46) | 0.524261 | 1.65(0.87,3.11) | 0.125508 | -0.04(-0.07,0) | 0.065043 | 0.93 | 0.507097 | 0 |
| milk type: soy milk vs. any other | 0.83(0.08,8.96) | 0.878045 | 0.73(0.07,7.32) | 0.790583 | 0.74(0,110.45) | 0.905568 | 0(-0.03,0.04) | 0.856891 | 1.14 | 0.862971 | 0 |
| milk type: other milk vs. never | 0.82(0.57,1.18) | 0.281 | 0.82(0.58,1.16) | 0.253463 | 1.18(0.67,2.08) | 0.567516 | -0.02(-0.07,0.03) | 0.350596 | 0.93 | 0.523977 | 0 |
| milk type: other milk vs. any other | 0.02(0,2.57) | 0.111367 | 0.01(0,0.78) | 0.038789 | 1.12(0,158624.71) | 0.984892 | -0.02(-0.1,0.06) | 0.672548 | 0.02 | 0.063782 | 1 |
| spread type: all spreads vs. never | 0.45(0.15,1.32) | 0.145968 | 0.7(0.25,1.94) | 0.492329 | 1.27(0.25,6.5) | 0.774886 | -0.01(-0.03,0.01) | 0.283654 | 0.57 | 0.089442 | 0 |
| spread type: butter + margarine vs. never | 0.6(0.3,1.2) | 0.147172 | 0.82(0.42,1.61) | 0.558925 | 1.06(0.35,3.2) | 0.918743 | -0.01(-0.02,0.01) | 0.546976 | 0.78 | 0.248008 | 0 |
| spread type: any oil based spread vs. never | 1.48(0.77,2.85) | 0.237374 | 1.41(0.76,2.6) | 0.271664 | 2.82(0.81,9.75) | 0.102218 | -0.02(-0.05,0.01) | 0.164734 | 1.24 | 0.364615 | 0 |
| spread type: butter and butter-like spreads vs. oil-based spreads | 1.53(0.72,3.22) | 0.268255 | 1.63(0.81,3.26) | 0.167983 | 1.02(0.31,3.29) | 0.978071 | 0.01(-0.01,0.03) | 0.244256 | 1.9 | 0.019104 | 0 |
| spread type: butter and margarine spreads vs. oil-based spreads | 1.4(0.75,2.62) | 0.285194 | 1.14(0.59,2.2) | 0.690881 | 1.24(0.32,4.76) | 0.758295 | 0(-0.02,0.03) | 0.811589 | 1.44 | 0.130585 | 0 |
| spread type: butter vs. never | 0.78(0.45,1.35) | 0.373092 | 1.06(0.6,1.88) | 0.838523 | 0.9(0.35,2.29) | 0.817079 | 0(-0.02,0.02) | 0.988829 | 0.89 | 0.524214 | 0 |
| spread type: butter vs. any other | 0.89(0.51,1.55) | 0.685307 | 0.94(0.5,1.78) | 0.855844 | 1.36(0.36,5.16) | 0.650159 | 0(-0.02,0.02) | 0.651447 | 1.01 | 0.940957 | 0 |
| spread type: tub margarine vs. never | 0.68(0.43,1.07) | 0.095804 | 0.81(0.53,1.22) | 0.304263 | 0.67(0.32,1.41) | 0.290873 | 0.02(-0.01,0.04) | 0.313526 | 0.94 | 0.732992 | 0 |
| spread type: tub margarine vs. any other | 1.97(0.3,12.89) | 0.478758 | 2.95(0.5,17.49) | 0.234656 | 18.67(0.36,973.94) | 0.146849 | -0.03(-0.07,0.01) | 0.188648 | 1.64 | 0.549521 | 1 |
| spread type: flora + benecol vs. never | 1.06(0.68,1.64) | 0.810054 | 1.01(0.68,1.51) | 0.952751 | 1.1(0.58,2.08) | 0.762084 | -0.01(-0.03,0.02) | 0.563081 | 0.94 | 0.594564 | 0 |
| spread type: flora + benecol vs. any other | 1.08(0.33,3.6) | 0.894411 | 1.09(0.36,3.32) | 0.876007 | 1.6(0.24,10.5) | 0.622681 | 0(-0.03,0.02) | 0.745259 | 1.21 | 0.655268 | 0 |
| spread type: olive oil spread vs. never | 0.94(0.57,1.56) | 0.824495 | 0.97(0.6,1.55) | 0.884611 | 0.89(0.36,2.23) | 0.804444 | 0(-0.03,0.03) | 0.893175 | 0.94 | 0.752486 | 0 |
| spread type: olive oil spread vs. any other | 1.2(0.26,5.44) | 0.814663 | 1.53(0.41,5.81) | 0.528543 | 2.21(0.29,16.67) | 0.440847 | -0.01(-0.04,0.02) | 0.522824 | 1.26 | 0.627763 | 0 |
| spread type: other oil-based spread vs. never | 1.31(0.81,2.14) | 0.275876 | 1.29(0.81,2.05) | 0.281238 | 1.89(0.82,4.31) | 0.132658 | -0.01(-0.03,0.01) | 0.427927 | 1.39 | 0.021939 | 0 |
| spread type: other oil-based spread vs. any other | 0.83(0.24,2.84) | 0.768498 | 0.83(0.27,2.54) | 0.743542 | 0.44(0.06,3.2) | 0.418919 | 0.01(-0.02,0.03) | 0.635482 | 0.68 | 0.354038 | 0 |
| spread type: low fat spread vs. never | 0.65(0.38,1.09) | 0.104351 | 0.81(0.51,1.28) | 0.366986 | 0.54(0.27,1.07) | 0.07684 | 0.03(0,0.06) | 0.08614 | 0.89 | 0.585083 | 0 |
| spread type: low fat spread vs. any other | 0.35(0.03,3.47) | 0.36841 | 0.32(0.04,2.91) | 0.311613 | 1.97(0.08,47.88) | 0.677374 | -0.02(-0.05,0.01) | 0.278982 | 0.44 | 0.3256 | 0 |
| bread type: white vs. any other | 1.43(0.93,2.21) | 0.103443 | 1.59(0.89,2.85) | 0.114839 | 1.98(0.58,6.72) | 0.274075 | -0.01(-0.02,0.01) | 0.485216 | 1.3 | 0.104406 | 0 |
| bread type: brown vs. any other | 0.61(0.14,2.77) | 0.5235 | 0.41(0.1,1.65) | 0.209495 | 0.3(0.02,5.67) | 0.422745 | 0(-0.03,0.03) | 0.964233 | 0.32 | 0.043992 | 0 |
| bread type: wholemeal/wholegrain vs. any other | 0.76(0.5,1.16) | 0.207508 | 0.76(0.45,1.3) | 0.324159 | 0.7(0.24,2.01) | 0.506733 | 0(-0.01,0.02) | 0.762169 | 0.82 | 0.185826 | 0 |
| bread type: white vs. wholemeal/wholegrain + brown | 1.31(0.87,1.98) | 0.199399 | 1.35(0.78,2.34) | 0.281932 | 2.31(0.79,6.74) | 0.125476 | -0.01(-0.02,0.01) | 0.254446 | 1.27 | 0.111409 | 0 |
| bread type: wholemeal/wholegrain vs. white + brown | 0.77(0.52,1.15) | 0.207653 | 0.78(0.47,1.29) | 0.33286 | 0.69(0.25,1.9) | 0.467309 | 0(-0.01,0.02) | 0.799396 | 0.78 | 0.086829 | 0 |
| cereal type: biscuit cereal vs. any other | 0.6(0.2,1.83) | 0.368622 | 0.63(0.21,1.87) | 0.40709 | 0.84(0.09,7.75) | 0.875659 | 0(-0.04,0.03) | 0.787376 | 0.63 | 0.267916 | 0 |
| cereal type: bran cereal vs. any other | 1.05(0.25,4.39) | 0.944279 | 1.9(0.48,7.52) | 0.362186 | 0.23(0.01,3.46) | 0.286274 | 0.02(-0.02,0.05) | 0.357543 | 0.75 | 0.569367 | 0 |
| cereal type: oat cereal vs. any other | 0.91(0.3,2.75) | 0.867117 | 1.14(0.39,3.38) | 0.811754 | 0.93(0.11,8.19) | 0.948169 | 0(-0.03,0.04) | 0.807094 | 1.19 | 0.700848 | 1 |
| cereal type: muesli vs. any other | 0.79(0.42,1.49) | 0.458474 | 0.63(0.28,1.38) | 0.243631 | 0.48(0.06,3.71) | 0.48469 | 0.01(-0.02,0.04) | 0.573794 | 0.85 | 0.534767 | 1 |
| cereal type: cornflakes/frosties vs. any other | 1.59(0.85,2.97) | 0.150066 | 1.71(0.81,3.58) | 0.157436 | 0.9(0.23,3.43) | 0.875023 | 0(-0.02,0.02) | 0.680016 | 1.17 | 0.461228 | 0 |
| coffee type: decaffeinated vs. any other | 1.05(0.68,1.63) | 0.808995 | 0.92(0.54,1.56) | 0.747858 | 1.62(0.51,5.1) | 0.410905 | 0(-0.02,0.01) | 0.630005 | 1.23 | 0.167849 | 0 |
| coffee type: ground+instant vs. other+decaff | 0.7(0.26,1.9) | 0.482273 | 0.84(0.3,2.35) | 0.736796 | 0.96(0.15,6.2) | 0.969401 | -0.01(-0.04,0.02) | 0.582815 | 0.59 | 0.144283 | 0 |
| coffee type: ground vs. any other | 0.83(0.26,2.64) | 0.746137 | 0.72(0.24,2.18) | 0.558675 | 1.18(0.11,12.97) | 0.890027 | 0(-0.04,0.04) | 0.99669 | 1.19 | 0.702419 | 0 |
| tablespoons of cooked vegetables per day | 1.37(0.8,2.37) | 0.254742 | 1.38(0.71,2.69) | 0.346383 | 3.47(0.68,17.58) | 0.133221 | -0.02(-0.04,0) | 0.118686 | 0.94 | 0.746848 | 1 |
| tablespoons of raw vegetables per day | 0.68(0.41,1.11) | 0.124727 | 0.6(0.32,1.11) | 0.103306 | 0.56(0.17,1.85) | 0.341923 | 0(-0.01,0.02) | 0.631318 | 0.74 | 0.083024 | 0 |
| pieces of fresh fruit per day | 0.88(0.61,1.26) | 0.477807 | 0.94(0.58,1.52) | 0.80357 | 1.6(0.64,3.96) | 0.312341 | -0.01(-0.02,0.01) | 0.362066 | 1.06 | 0.630105 | 1 |
| pieces of dried fruit per day | 1.44(0.82,2.56) | 0.206542 | 1.71(0.88,3.32) | 0.113732 | 2.64(0.57,12.19) | 0.212813 | -0.01(-0.03,0.01) | 0.374028 | 1.36 | 0.165732 | 1 |
| slices of bread per week | 1.19(0.71,2) | 0.505837 | 1.25(0.67,2.33) | 0.487424 | 1.94(0.56,6.68) | 0.295017 | -0.01(-0.03,0.01) | 0.324969 | 1.07 | 0.717525 | 0 |
| bowls of cereal per week | 1.23(0.79,1.93) | 0.352177 | 1.5(0.88,2.56) | 0.139818 | 1.19(0.36,3.92) | 0.779022 | 0(-0.02,0.01) | 0.711511 | 0.96 | 0.80123 | 1 |
| cups of tea per day | 0.53(0.32,0.87) | 0.011553 | 0.61(0.38,0.98) | 0.038991 | 0.56(0.26,1.18) | 0.1289 | 0(-0.01,0.01) | 0.423586 | 0.74 | 0.042806 | 0 |
| cups of coffee per day | 0.62(0.4,0.97) | 0.037004 | 0.63(0.43,0.94) | 0.022866 | 0.74(0.41,1.32) | 0.303141 | 0(-0.01,0.01) | 0.71695 | 0.67 | 0.007816 | 0 |
| glasses of water per day | 1.69(1.12,2.55) | 0.011915 | 1.5(0.98,2.31) | 0.063846 | 1.78(0.82,3.86) | 0.143555 | 0(-0.01,0.01) | 0.472795 | 1.36 | 0.016443 | 0 |
| champagne/white wine glasses per month | 0.98(0.52,1.87) | 0.962256 | 0.84(0.41,1.72) | 0.62723 | 0.37(0.1,1.44) | 0.15226 | 0.01(-0.01,0.03) | 0.179398 | 0.89 | 0.614571 | 0 |
| red wine glasses per month | 0.74(0.46,1.2) | 0.227657 | 0.75(0.42,1.35) | 0.336926 | 1.14(0.38,3.46) | 0.814702 | -0.01(-0.02,0.01) | 0.360373 | 0.7 | 0.034516 | 0 |
| beer/cider glasses per month | 0.78(0.47,1.28) | 0.326617 | 0.94(0.54,1.64) | 0.830447 | 0.83(0.29,2.35) | 0.719411 | 0(-0.02,0.01) | 0.812095 | 0.73 | 0.094365 | 0 |
| spirits measures per month | 1.9(0.79,4.57) | 0.150557 | 1.65(0.72,3.78) | 0.235917 | 5.89(1.57,22.04) | 0.008485 | -0.03(-0.05,-0.01) | 0.004991 | 1.11 | 0.760002 | 1 |
| fortwine glasses per month | 2.13(0.56,8.11) | 0.266085 | 1.74(0.51,6) | 0.377573 | 13.78(1.26,150.49) | 0.031538 | -0.04(-0.07,0) | 0.07588 | 1.89 | 0.146881 | 0 |
| other alcohol glasses per month | 0.94(0.29,3.01) | 0.920585 | 0.91(0.31,2.67) | 0.862246 | 0.86(0.09,8.14) | 0.895517 | 0(-0.06,0.05) | 0.961858 | 0.82 | 0.633004 | 0 |
| total drinks of alcohol per month | 0.82(0.56,1.19) | 0.290111 | 0.9(0.59,1.36) | 0.616192 | 0.63(0.33,1.23) | 0.179307 | 0(-0.01,0.01) | 0.373636 | 0.84 | 0.156381 | 0 |
| overall oily fish intake | 1.04(0.68,1.58) | 0.867013 | 1.1(0.66,1.85) | 0.713886 | 0.57(0.17,1.92) | 0.361411 | 0.01(-0.01,0.02) | 0.336616 | 1.01 | 0.944883 | 1 |
| overall non-oily fish intake | 1.31(0.68,2.52) | 0.426446 | 1(0.49,2.07) | 0.997089 | 0.54(0.12,2.41) | 0.423509 | 0.01(-0.01,0.04) | 0.164396 | 1.49 | 0.091381 | 0 |
| overall processed meat intake | 0.82(0.45,1.5) | 0.516719 | 0.63(0.3,1.32) | 0.219237 | 3.73(0.81,17.07) | 0.089969 | -0.02(-0.04,0) | 0.126539 | 1.19 | 0.418997 | 0 |
| overall poultry intake | 1.7(0.77,3.71) | 0.186333 | 1.46(0.57,3.72) | 0.426602 | 0.44(0.08,2.36) | 0.33455 | 0.02(0,0.04) | 0.097975 | 1.67 | 0.086889 | 0 |
| overall beef intake | 1.46(0.8,2.66) | 0.220891 | 1.54(0.76,3.12) | 0.227111 | 2.04(0.52,8.07) | 0.309216 | -0.01(-0.02,0.01) | 0.521853 | 1.33 | 0.198547 | 0 |
| overall lamb/mutton intake | 0.86(0.5,1.49) | 0.589841 | 0.9(0.48,1.67) | 0.735614 | 0.74(0.22,2.57) | 0.639201 | 0.01(-0.01,0.02) | 0.544742 | 1.07 | 0.726428 | 0 |
| overall pork intake | 0.7(0.36,1.35) | 0.285569 | 0.66(0.33,1.33) | 0.242416 | 1.41(0.4,4.93) | 0.591471 | -0.01(-0.03,0.01) | 0.319747 | 0.78 | 0.275124 | 0 |
| overall cheese intake | 1.09(0.72,1.65) | 0.681981 | 1.25(0.74,2.12) | 0.408063 | 1.22(0.47,3.13) | 0.68181 | -0.01(-0.02,0.01) | 0.355318 | 0.8 | 0.122353 | 0 |
| frequency of adding salt to food | 0.92(0.62,1.36) | 0.670066 | 0.88(0.54,1.46) | 0.62884 | 0.9(0.33,2.44) | 0.834044 | 0(-0.01,0.01) | 0.932189 | 0.86 | 0.317471 | 1 |
| temperature of hot drinks | 0.91(0.58,1.44) | 0.697793 | 0.74(0.42,1.33) | 0.317039 | 0.25(0.08,0.82) | 0.02174 | 0.02(0,0.03) | 0.029007 | 0.89 | 0.483662 | 1 |
| overall alcohol intake | 0.7(0.48,1.01) | 0.055846 | 0.81(0.52,1.25) | 0.344682 | 0.86(0.42,1.79) | 0.694477 | 0(-0.01,0.01) | 0.888385 | 0.82 | 0.105206 | 0 |
| among current drinkers, drinks usually with meals: yes, it varies, no | 0.68(0.42,1.1) | 0.119678 | 0.68(0.38,1.24) | 0.208758 | 0.8(0.25,2.64) | 0.71997 | 0(-0.02,0.02) | 0.87078 | 0.73 | 0.061742 | 0 |
| milk type: skimmed, semi-skimmed, full cream (QT) | 1.38(0.62,3.08) | 0.431536 | 1.35(0.6,3.02) | 0.469778 | 0.91(0.14,5.83) | 0.922256 | 0(-0.02,0.03) | 0.926357 | 0.99 | 0.974871 | 0 |

MR: mendelian randomization; MRPRESSO: MR pleiotropy residual sum and outlier; OR: odd ratio; CI: confidence interval; No.: number.

**Table S7. Leave-one-out analysis of association between genetically predicted dietary habits and migraine with aura.**

| **Outcome** | **IVW Estimate**  **[Min, Max] ^a^** | **P value [Min, Max] ^b^** |
| --- | --- | --- |
| alcohol drinker status: current + former vs. never | [0.99,1.69] | [0.434276536644302,0.984271500025764] |
| alcohol drinker status: current vs. never | [1.33,2.25] | [0.214943316491034,0.660229321947482] |
| among current drinkers, drinks usually with meals: yes + it varies vs. no | [0.56,0.63] | [0.00385118633643444,0.0171864950942962] |
| among current drinkers, drinks usually with meals: yes vs. no | [0.84,0.88] | [0.118459179726585,0.258545150698845] |
| never eat eggs vs. no eggs, dairy, wheat, or sugar restrictions | [0.52,2.43] | [0.512694309535028,0.976830710797849] |
| never eat eggs vs. no eggs restrictions | [1,4.03] | [0.454173919560808,0.998131350763803] |
| never eat dairy vs. no eggs, dairy, wheat, or sugar restrictions | [0.66,2.22] | [0.424874053428296,0.990166647083907] |
| never eat dairy vs. no dairy restrictions | [0.6,2.64] | [0.436605277636198,0.994514231574355] |
| never eat wheat vs. no eggs, dairy, wheat, or sugar restrictions | [1.17,1.8] | [0.252697694676967,0.793124072684946] |
| never eat wheat vs. no wheat restrictions | [1.26,2.11] | [0.221654699083268,0.745907500768993] |
| never eat sugar vs. no eggs, dairy, wheat, or sugar restrictions | [0.79,0.87] | [0.296269079967624,0.548994684266363] |
| never eat sugar vs. no sugar restrictions | [0.77,0.86] | [0.25702653352165,0.525981141624173] |
| milk type: dairy-based milk vs. never | [0.94,3.18] | [0.231909327865164,0.952195661547697] |
| milk type: any milk vs. never | [0.4,1.13] | [0.346512762494731,0.946596364106015] |
| milk type: full cream vs. never | [1.02,1.12] | [0.352189876136866,0.869864442493932] |
| milk type: full cream vs. any other | [0.58,0.8] | [0.303549269003577,0.668499656199695] |
| milk type: semi-skimmed vs. never | [2.35,4.18] | [0.0451721058656642,0.241658481951322] |
| milk type: semi-skimmed vs. any other | [0.87,1.13] | [0.671733086313386,0.993741733125715] |
| milk type: skimmed vs. never | [0.83,1.12] | [0.493293498953315,0.99945382924869] |
| milk type: skimmed vs. any other | [0.82,1.12] | [0.546218701419795,0.951047200789197] |
| milk type: soy milk vs. never | [0.9,0.97] | [0.333400838232439,0.802092478262751] |
| milk type: soy milk vs. any other | [0.88,1.73] | [0.54203608544452,0.994065123549816] |
| milk type: other milk vs. never | [0.88,1.03] | [0.344380861840601,0.81766191176132] |
| milk type: other milk vs. any other | [0.02,0.36] | [0.0439686467940327,0.698165232812147] |
| spread type: all spreads vs. never | [0.47,0.64] | [0.0408382110607121,0.213020939701771] |
| spread type: butter + margarine vs. never | [0.7,0.85] | [0.135624280915739,0.47127141366508] |
| spread type: any oil based spread vs. never | [1.13,1.36] | [0.169187258595357,0.604438054675405] |
| spread type: butter and butter-like spreads vs. oil-based spreads | [1.79,2.09] | [0.00276904463706851,0.0263934304524056] |
| spread type: butter and margarine spreads vs. oil-based spreads | [1.37,1.55] | [0.0552150558786908,0.18959649641693] |
| spread type: butter vs. never | [0.83,0.95] | [0.322166945353839,0.801815564713935] |
| spread type: butter vs. any other | [0.97,1.07] | [0.709152910394222,0.999310699366453] |
| spread type: tub margarine vs. never | [0.89,1.03] | [0.46362966535596,0.917344412076478] |
| spread type: tub margarine vs. any other | [1.21,2.21] | [0.313194000998847,0.817108402140201] |
| spread type: flora + benecol vs. never | [0.89,0.98] | [0.494837339117106,0.874284995348906] |
| spread type: flora + benecol vs. any other | [1.06,1.43] | [0.415315202025648,0.889115369454617] |
| spread type: olive oil spread vs. never | [0.88,1.03] | [0.45067204408706,0.980691361571772] |
| spread type: olive oil spread vs. any other | [1.07,1.54] | [0.429102202704893,0.90185600059809] |
| spread type: other oil-based spread vs. never | [1.32,1.46] | [0.0258874485133643,0.0980108645480068] |
| spread type: other oil-based spread vs. any other | [0.62,0.86] | [0.282441163986504,0.728898453837542] |
| spread type: low fat spread vs. never | [0.81,1.08] | [0.28814207621121,0.827609456793889] |
| spread type: low fat spread vs. any other | [0.3,0.63] | [0.164859762610086,0.591827246914559] |
| bread type: white vs. any other | [1.25,1.35] | [0.0535939044057011,0.170001216740393] |
| bread type: brown vs. any other | [0.26,0.38] | [0.0162299119036965,0.0841345244881862] |
| bread type: wholemeal/wholegrain vs. any other | [0.79,0.84] | [0.105862597401496,0.259912698155291] |
| bread type: white vs. wholemeal/wholegrain + brown | [1.22,1.32] | [0.0596001758721329,0.179567285945776] |
| bread type: wholemeal/wholegrain vs. white + brown | [0.75,0.8] | [0.0467517415200163,0.122048234578499] |
| cereal type: biscuit cereal vs. any other | [0.54,0.73] | [0.13724429649425,0.443013523673315] |
| cereal type: bran cereal vs. any other | [0.63,1.05] | [0.368202720704317,0.928164137527864] |
| cereal type: oat cereal vs. any other | [1.04,1.37] | [0.486174216982542,0.936882149398908] |
| cereal type: muesli vs. any other | [0.8,0.91] | [0.369338816736634,0.705935254927043] |
| cereal type: cornflakes/frosties vs. any other | [1.05,1.25] | [0.318258020418723,0.825572400935355] |
| coffee type: decaffeinated vs. any other | [1.19,1.27] | [0.111602428650416,0.23547516128824] |
| coffee type: ground+instant vs. other+decaff | [0.45,0.69] | [0.0288114945818456,0.288846996736449] |
| coffee type: ground vs. any other | [0.96,1.63] | [0.278810667453463,0.994789099007358] |
| tablespoons of cooked vegetables per day | [0.94,1.04] | [0.746327887180966,0.999774301859478] |
| tablespoons of raw vegetables per day | [0.69,0.78] | [0.0337958115963947,0.149624805053578] |
| pieces of fresh fruit per day | [1.02,1.09] | [0.522156439423969,0.905209458634553] |
| pieces of dried fruit per day | [1.28,1.44] | [0.0964757411738567,0.26868233915723] |
| slices of bread per week | [1.02,1.15] | [0.45125173092529,0.899968601615743] |
| bowls of cereal per week | [0.92,1] | [0.648422462209268,0.986115575532508] |
| cups of tea per day | [0.72,0.76] | [0.0203970393924209,0.0771564809599222] |
| cups of coffee per day | [0.65,0.69] | [0.0028066410010042,0.0151540575882437] |
| glasses of water per day | [1.33,1.39] | [0.00852083026311644,0.0273990254544746] |
| champagne/white wine glasses per month | [0.83,0.94] | [0.407277256709432,0.799074948326987] |
| red wine glasses per month | [0.66,0.73] | [0.0144425141148568,0.0601937553105917] |
| beer/cider glasses per month | [0.71,0.77] | [0.0573388035072273,0.141088015071274] |
| spirits measures per month | [0.99,1.22] | [0.518238134996912,0.965837182905669] |
| fortwine glasses per month | [1.55,2.25] | [0.10439116731238,0.394180348841609] |
| other alcohol glasses per month | [0.65,1.04] | [0.336855110888249,0.953862355723333] |
| total drinks of alcohol per month | [0.82,0.87] | [0.100003310417793,0.239661803764745] |
| overall oily fish intake | [0.97,1.04] | [0.795704810250642,0.998619701311588] |
| overall non-oily fish intake | [1.36,1.61] | [0.0488348278441792,0.19551481197141] |
| overall processed meat intake | [1.11,1.25] | [0.294576145897866,0.622716334978674] |
| overall poultry intake | [1.5,1.88] | [0.0290055754262322,0.160487529077489] |
| overall beef intake | [1.24,1.42] | [0.108917146638782,0.336052186447544] |
| overall lamb/mutton intake | [1.01,1.12] | [0.572174067923413,0.944718216233059] |
| overall pork intake | [0.69,0.82] | [0.103104257070548,0.386545744407507] |
| overall cheese intake | [0.78,0.82] | [0.0808101578441682,0.163698180080023] |
| frequency of adding salt to food | [0.84,0.88] | [0.221586181064541,0.407155074084731] |
| temperature of hot drinks | [0.83,0.9] | [0.278770840868222,0.515798903545435] |
| overall alcohol intake | [0.81,0.85] | [0.0703261961568111,0.162336108937406] |
| among current drinkers, drinks usually with meals: yes, it varies, no | [0.71,0.76] | [0.0362933943294772,0.0945609804455719] |
| milk type: skimmed, semi-skimmed, full cream (QT) | [0.92,1.09] | [0.758781035964727,0.997646744073769] |

**^a^** the minimum value and maximum value of inverse variance weighted estimate;

**^b^** the minimum value and maximum value of P value;

IVW: inverse variance weighted.

**Table S8. Associations between genetically predicted dietary habits and migraine without aura in sensitivity analysis.**

| **Exposure** | **Weighted median** | | **Mode-based** | | **MR-Egger** | | | | **MRPRESSO** | | |
| --- | --- | --- | --- | --- | --- | --- | --- | --- | --- | --- | --- |
|  | **OR (95% CI)** | **P value** | **OR (95% CI)** | **P value** | **OR (95% CI)** | **P value** | **Intercept** | **P value** | **OR** | **P value** | **No. of outliers** |
| alcohol drinker status: current + former vs. never | 1.32(0.17,10.45) | 0.790724 | 0.84(0.11,6.51) | 0.86986 | 0.47(0.01,15.71) | 0.67517 | 0(-0.02,0.03) | 0.709232 | 0.85 | 0.847726 | 0 |
| alcohol drinker status: current vs. never | 1.37(0.18,10.42) | 0.763068 | 1.24(0.18,8.64) | 0.827868 | 1.31(0.03,53.6) | 0.886246 | 0(-0.03,0.02) | 0.743927 | 1.23 | 0.795533 | 1 |
| among current drinkers, drinks usually with meals: yes + it varies vs. no | 0.51(0.28,0.9) | 0.021616 | 0.58(0.28,1.17) | 0.125677 | 0.64(0.14,2.98) | 0.57158 | 0(-0.02,0.02) | 0.906861 | 0.59 | 0.015789 | 0 |
| among current drinkers, drinks usually with meals: yes vs. no | 0.61(0.43,0.86) | 0.004382 | 0.54(0.35,0.85) | 0.006757 | 0.8(0.33,1.9) | 0.60795 | 0(-0.02,0.02) | 0.85666 | 0.74 | 0.013525 | 0 |
| never eat eggs vs. no eggs, dairy, wheat, or sugar restrictions | 0.99(0.03,32.27) | 0.997423 | 0.93(0.03,25.39) | 0.964353 | 0.82(0,1898.44) | 0.959464 | 0(-0.06,0.07) | 0.89952 | 1.29 | 0.87186 | 0 |
| never eat eggs vs. no eggs restrictions | 7.34(0.06,861.11) | 0.412113 | 3.26(0.03,326.99) | 0.615266 | 0.07(0,451.39) | 0.553348 | 0.03(-0.02,0.09) | 0.262465 | 6.88 | 0.325926 | 0 |
| never eat dairy vs. no eggs, dairy, wheat, or sugar restrictions | 0.3(0.02,4.99) | 0.404806 | 0.25(0.02,3.43) | 0.301515 | 0(0,0.24) | 0.00908 | 0.05(0.01,0.08) | 0.007476 | 0.61 | 0.637689 | 0 |
| never eat dairy vs. no dairy restrictions | 0.21(0.01,6.69) | 0.376327 | 0.15(0.01,3.63) | 0.241769 | 0(0,0.15) | 0.007918 | 0.05(0.01,0.08) | 0.006535 | 0.53 | 0.620455 | 0 |
| never eat wheat vs. no eggs, dairy, wheat, or sugar restrictions | 1.02(0.24,4.23) | 0.98201 | 0.7(0.21,2.29) | 0.553277 | 1.48(0.23,9.49) | 0.678712 | -0.03(-0.07,0.01) | 0.14976 | 0.48 | 0.123721 | 0 |
| never eat wheat vs. no wheat restrictions | 1.05(0.18,5.98) | 0.95952 | 0.61(0.14,2.61) | 0.508296 | 0.32(0.03,3) | 0.318948 | 0.01(-0.02,0.05) | 0.507122 | 0.6 | 0.417072 | 0 |
| never eat sugar vs. no eggs, dairy, wheat, or sugar restrictions | 0.98(0.55,1.76) | 0.958507 | 1.01(0.51,2.02) | 0.975898 | 0.95(0.21,4.31) | 0.949707 | 0(-0.02,0.02) | 0.902392 | 1.04 | 0.840382 | 0 |
| never eat sugar vs. no sugar restrictions | 0.91(0.49,1.68) | 0.767638 | 0.86(0.42,1.73) | 0.664613 | 1.06(0.28,4.09) | 0.928386 | 0(-0.02,0.02) | 0.98707 | 1.08 | 0.725903 | 0 |
| milk type: dairy-based milk vs. never | 0.41(0.02,8.97) | 0.571359 | 0.32(0.02,5.61) | 0.437879 | 0.05(0,634.83) | 0.52726 | 0.02(-0.06,0.09) | 0.649309 | 0.17 | 0.169077 | 1 |
| milk type: any milk vs. never | 0.38(0.02,6.73) | 0.508042 | 0.57(0.05,7.22) | 0.664915 | 0(0,3.07) | 0.101368 | 0.05(-0.01,0.11) | 0.105203 | 0.31 | 0.321821 | 1 |
| milk type: full cream vs. never | 1.02(0.72,1.43) | 0.928349 | 1.03(0.71,1.48) | 0.883678 | 1.04(0.52,2.09) | 0.902772 | 0(-0.04,0.03) | 0.805694 | 0.96 | 0.718211 | 0 |
| milk type: full cream vs. any other | 1.27(0.28,5.81) | 0.754497 | 1.22(0.27,5.57) | 0.793645 | 9.74(0.24,389.16) | 0.226198 | -0.02(-0.05,0.02) | 0.31727 | 1.61 | 0.340121 | 0 |
| milk type: semi-skimmed vs. never | 0.26(0.03,2.43) | 0.23712 | 0.26(0.03,1.94) | 0.18795 | 0.02(0,14) | 0.247068 | 0.03(-0.04,0.11) | 0.379128 | 0.18 | 0.065023 | 1 |
| milk type: semi-skimmed vs. any other | 0.85(0.31,2.37) | 0.757098 | 0.81(0.34,1.94) | 0.635608 | 0.48(0.12,1.91) | 0.296065 | 0.01(-0.01,0.03) | 0.448033 | 0.77 | 0.355613 | 0 |
| milk type: skimmed vs. never | 1.67(0.75,3.68) | 0.206864 | 1.29(0.61,2.74) | 0.5119 | 1.15(0.26,5.05) | 0.85087 | 0(-0.04,0.04) | 0.903772 | 1.25 | 0.457127 | 0 |
| milk type: skimmed vs. any other | 1(0.36,2.77) | 0.99852 | 1.01(0.38,2.74) | 0.976632 | 0.33(0.04,2.79) | 0.309447 | 0.02(-0.01,0.05) | 0.132282 | 1.52 | 0.307969 | 0 |
| milk type: soy milk vs. never | 1.3(0.97,1.75) | 0.081113 | 1.26(0.94,1.69) | 0.117465 | 1.87(0.95,3.66) | 0.069491 | -0.03(-0.07,0.01) | 0.21535 | 1.25 | 0.015843 | 0 |
| milk type: soy milk vs. any other | 0.6(0.05,7.84) | 0.697769 | 0.35(0.03,4.11) | 0.403305 | 0.02(0,4.87) | 0.169285 | 0.02(-0.02,0.06) | 0.324489 | 0.3 | 0.192575 | 0 |
| milk type: other milk vs. never | 0.66(0.45,0.98) | 0.037244 | 0.65(0.45,0.94) | 0.022837 | 0.82(0.41,1.63) | 0.567677 | -0.01(-0.07,0.05) | 0.822791 | 0.76 | 0.100033 | 0 |
| milk type: other milk vs. any other | 0.01(0,4.12) | 0.138749 | 0.01(0,2.48) | 0.099189 | 0.13(0,34260.79) | 0.747121 | 0(-0.09,0.08) | 0.988203 | 0.12 | 0.424488 | 1 |
| spread type: all spreads vs. never | 0.53(0.18,1.56) | 0.248334 | 0.63(0.22,1.82) | 0.396082 | 0.71(0.12,4.26) | 0.705632 | 0(-0.02,0.02) | 0.80607 | 0.87 | 0.712618 | 0 |
| spread type: butter + margarine vs. never | 0.86(0.42,1.78) | 0.69179 | 0.86(0.43,1.72) | 0.671937 | 0.77(0.21,2.81) | 0.689861 | 0.01(-0.01,0.03) | 0.534932 | 1.12 | 0.679181 | 0 |
| spread type: any oil based spread vs. never | 1.16(0.6,2.24) | 0.664601 | 1.28(0.67,2.43) | 0.45298 | 0.89(0.25,3.14) | 0.861021 | 0.01(-0.02,0.04) | 0.489273 | 1.35 | 0.171691 | 0 |
| spread type: butter and butter-like spreads vs. oil-based spreads | 0.88(0.41,1.89) | 0.74567 | 0.74(0.35,1.56) | 0.427206 | 0.71(0.21,2.45) | 0.588546 | 0.01(-0.02,0.03) | 0.648221 | 0.92 | 0.764641 | 0 |
| spread type: butter and margarine spreads vs. oil-based spreads | 0.9(0.48,1.68) | 0.732967 | 0.99(0.52,1.9) | 0.98366 | 1.37(0.38,4.9) | 0.62714 | 0(-0.03,0.02) | 0.696345 | 1.08 | 0.730098 | 0 |
| spread type: butter vs. never | 1.23(0.7,2.17) | 0.465809 | 1.14(0.66,1.98) | 0.646072 | 1.36(0.5,3.67) | 0.548339 | 0(-0.03,0.02) | 0.642877 | 1.09 | 0.653544 | 0 |
| spread type: butter vs. any other | 0.82(0.45,1.47) | 0.499688 | 1.12(0.53,2.33) | 0.76833 | 1.56(0.4,6.03) | 0.519151 | -0.01(-0.03,0.01) | 0.368468 | 0.86 | 0.457903 | 0 |
| spread type: tub margarine vs. never | 0.9(0.57,1.42) | 0.637946 | 0.94(0.62,1.44) | 0.787787 | 1.02(0.5,2.08) | 0.945876 | 0(-0.03,0.03) | 0.971303 | 1.04 | 0.806451 | 0 |
| spread type: tub margarine vs. any other | 4.16(0.62,27.82) | 0.141186 | 3.33(0.56,19.71) | 0.185605 | 0.6(0.02,21.3) | 0.779327 | 0.02(-0.02,0.05) | 0.365935 | 2.72 | 0.180298 | 0 |
| spread type: flora + benecol vs. never | 0.87(0.54,1.41) | 0.583277 | 0.89(0.57,1.38) | 0.601493 | 1.13(0.57,2.21) | 0.728256 | 0(-0.03,0.02) | 0.765302 | 1.03 | 0.843573 | 0 |
| spread type: flora + benecol vs. any other | 0.54(0.14,1.99) | 0.351676 | 0.84(0.24,2.97) | 0.780908 | 0.16(0.02,1.16) | 0.070643 | 0.02(-0.01,0.04) | 0.144417 | 0.6 | 0.278718 | 0 |
| spread type: olive oil spread vs. never | 1.29(0.76,2.19) | 0.345804 | 1.19(0.71,1.98) | 0.50662 | 1.13(0.46,2.77) | 0.788934 | 0(-0.03,0.03) | 0.799511 | 1.26 | 0.176039 | 0 |
| spread type: olive oil spread vs. any other | 0.17(0.03,0.88) | 0.034056 | 0.24(0.05,1.09) | 0.063824 | 0.9(0.07,10.99) | 0.933343 | -0.01(-0.05,0.03) | 0.532287 | 0.45 | 0.237911 | 0 |
| spread type: other oil-based spread vs. never | 1.11(0.66,1.88) | 0.696414 | 1.2(0.74,1.95) | 0.463532 | 1.46(0.6,3.58) | 0.404589 | 0(-0.03,0.03) | 0.915646 | 1.53 | 0.021988 | 0 |
| spread type: other oil-based spread vs. any other | 1.46(0.35,6.05) | 0.60119 | 1.35(0.37,4.9) | 0.650853 | 2.43(0.2,29.51) | 0.486735 | -0.01(-0.04,0.03) | 0.635603 | 1.41 | 0.536106 | 0 |
| spread type: low fat spread vs. never | 0.9(0.51,1.58) | 0.709199 | 0.98(0.61,1.57) | 0.922449 | 0.52(0.25,1.08) | 0.079565 | 0.04(0,0.07) | 0.045004 | 0.98 | 0.895819 | 0 |
| spread type: low fat spread vs. any other | 0.24(0.02,3.03) | 0.267172 | 0.39(0.03,4.44) | 0.444338 | 0.07(0,2.55) | 0.14982 | 0.02(-0.02,0.06) | 0.255609 | 0.43 | 0.384123 | 0 |
| bread type: white vs. any other | 1.81(1.14,2.85) | 0.011071 | 2.09(1.16,3.79) | 0.014666 | 0.73(0.21,2.49) | 0.616726 | 0.01(-0.01,0.03) | 0.182451 | 1.64 | 0.002358 | 0 |
| bread type: brown vs. any other | 1.01(0.19,5.38) | 0.986737 | 0.94(0.2,4.37) | 0.93723 | 2.3(0.06,87.16) | 0.653946 | -0.01(-0.05,0.04) | 0.756804 | 1.34 | 0.657412 | 0 |
| bread type: wholemeal/wholegrain vs. any other | 0.61(0.4,0.95) | 0.028808 | 0.57(0.32,0.99) | 0.047749 | 0.49(0.17,1.43) | 0.192393 | 0(-0.01,0.02) | 0.622717 | 0.64 | 0.002465 | 0 |
| bread type: white vs. wholemeal/wholegrain + brown | 1.66(1.09,2.54) | 0.018741 | 1.95(1.13,3.39) | 0.017125 | 0.75(0.25,2.21) | 0.598124 | 0.01(-0.01,0.03) | 0.188385 | 1.51 | 0.005343 | 0 |
| bread type: wholemeal/wholegrain vs. white + brown | 0.55(0.37,0.83) | 0.004705 | 0.54(0.32,0.92) | 0.023618 | 0.93(0.34,2.5) | 0.882914 | -0.01(-0.02,0.01) | 0.43438 | 0.64 | 0.001137 | 0 |
| cereal type: biscuit cereal vs. any other | 0.86(0.26,2.89) | 0.805966 | 0.66(0.2,2.15) | 0.486291 | 0.55(0.05,6.11) | 0.625388 | 0(-0.03,0.04) | 0.823686 | 0.71 | 0.442057 | 0 |
| cereal type: bran cereal vs. any other | 1.31(0.29,6) | 0.723636 | 1.91(0.45,8.13) | 0.381429 | 0.17(0.01,4.35) | 0.281973 | 0.03(-0.02,0.07) | 0.209965 | 1.17 | 0.803144 | 0 |
| cereal type: oat cereal vs. any other | 0.47(0.16,1.33) | 0.154719 | 0.52(0.18,1.56) | 0.245457 | 1.06(0.17,6.66) | 0.952736 | -0.01(-0.04,0.02) | 0.548764 | 0.63 | 0.152139 | 0 |
| cereal type: muesli vs. any other | 0.52(0.28,0.98) | 0.044058 | 0.6(0.28,1.28) | 0.185472 | 0.79(0.13,4.69) | 0.796418 | 0(-0.03,0.02) | 0.729163 | 0.58 | 0.018358 | 0 |
| cereal type: cornflakes/frosties vs. any other | 1.35(0.7,2.6) | 0.372081 | 1.69(0.82,3.49) | 0.154181 | 2.47(0.6,10.22) | 0.211346 | 0(-0.02,0.02) | 0.767138 | 2.02 | 0.002922 | 0 |
| coffee type: decaffeinated vs. any other | 0.73(0.46,1.16) | 0.186624 | 0.6(0.34,1.07) | 0.08153 | 2.95(0.85,10.28) | 0.089251 | -0.02(-0.04,0) | 0.020867 | 0.71 | 0.043892 | 0 |
| coffee type: ground+instant vs. other+decaff | 0.93(0.33,2.65) | 0.898058 | 0.96(0.34,2.74) | 0.937109 | 1(0.12,8.26) | 0.998851 | -0.01(-0.04,0.03) | 0.7698 | 0.74 | 0.460127 | 0 |
| coffee type: ground vs. any other | 0.98(0.31,3.14) | 0.973216 | 0.98(0.33,2.95) | 0.97396 | 5.96(0.68,52.15) | 0.106645 | -0.03(-0.06,0.01) | 0.122719 | 1.22 | 0.61486 | 0 |
| tablespoons of cooked vegetables per day | 0.58(0.33,1.01) | 0.052908 | 0.65(0.33,1.3) | 0.226038 | 0.27(0.06,1.22) | 0.089063 | 0.01(-0.01,0.03) | 0.282928 | 0.6 | 0.009516 | 0 |
| tablespoons of raw vegetables per day | 0.68(0.4,1.16) | 0.154272 | 0.84(0.44,1.61) | 0.60393 | 0.27(0.07,0.99) | 0.048251 | 0.02(0,0.03) | 0.0893 | 0.79 | 0.225644 | 0 |
| pieces of fresh fruit per day | 0.92(0.63,1.36) | 0.686728 | 0.77(0.48,1.23) | 0.275928 | 0.52(0.21,1.27) | 0.151444 | 0.01(0,0.02) | 0.082942 | 1.11 | 0.418846 | 0 |
| pieces of dried fruit per day | 0.58(0.32,1.05) | 0.070087 | 0.59(0.3,1.16) | 0.123905 | 1.49(0.38,5.8) | 0.568815 | -0.01(-0.02,0.01) | 0.422759 | 0.87 | 0.475544 | 0 |
| slices of bread per week | 1.37(0.8,2.37) | 0.25573 | 1.1(0.59,2.06) | 0.755645 | 1.67(0.45,6.26) | 0.444941 | -0.01(-0.02,0.01) | 0.534946 | 1.12 | 0.558372 | 0 |
| bowls of cereal per week | 0.7(0.44,1.11) | 0.129667 | 0.75(0.43,1.3) | 0.304889 | 1.39(0.48,3.99) | 0.543621 | -0.01(-0.02,0) | 0.183388 | 0.7 | 0.023818 | 0 |
| cups of tea per day | 0.85(0.51,1.43) | 0.542653 | 0.97(0.6,1.57) | 0.914095 | 0.52(0.23,1.18) | 0.117559 | 0.01(0,0.02) | 0.105605 | 0.97 | 0.856265 | 0 |
| cups of coffee per day | 0.75(0.47,1.19) | 0.215655 | 0.67(0.45,0.99) | 0.043992 | 0.7(0.39,1.23) | 0.211142 | 0(-0.01,0.01) | 0.608084 | 0.79 | 0.077121 | 0 |
| glasses of water per day | 1.12(0.73,1.74) | 0.599884 | 1.05(0.66,1.66) | 0.836688 | 1.11(0.49,2.54) | 0.797031 | 0(-0.01,0.01) | 0.902167 | 1.06 | 0.664364 | 0 |
| champagne/white wine glasses per month | 0.66(0.33,1.32) | 0.241444 | 0.69(0.31,1.58) | 0.382695 | 0.61(0.15,2.56) | 0.502918 | 0(-0.02,0.02) | 0.858627 | 0.69 | 0.120007 | 0 |
| red wine glasses per month | 0.6(0.36,0.98) | 0.042171 | 0.7(0.39,1.26) | 0.229266 | 0.82(0.26,2.64) | 0.741715 | -0.01(-0.02,0.01) | 0.465533 | 0.54 | 0.00079 | 0 |
| beer/cider glasses per month | 0.93(0.54,1.61) | 0.79918 | 0.95(0.51,1.78) | 0.874939 | 1.35(0.44,4.17) | 0.599921 | -0.01(-0.03,0.01) | 0.259964 | 0.74 | 0.128317 | 0 |
| spirits measures per month | 0.82(0.34,1.98) | 0.661633 | 0.71(0.32,1.6) | 0.412779 | 1.11(0.3,4.08) | 0.874682 | -0.01(-0.03,0.01) | 0.388265 | 0.67 | 0.204897 | 0 |
| fortwine glasses per month | 1.13(0.26,4.92) | 0.870668 | 0.92(0.25,3.42) | 0.900635 | 0.14(0.01,2.21) | 0.164418 | 0.04(0,0.09) | 0.056736 | 1.64 | 0.424245 | 0 |
| other alcohol glasses per month | 0.19(0.06,0.63) | 0.006716 | 0.16(0.05,0.52) | 0.001938 | 0.15(0.01,1.57) | 0.11337 | 0.01(-0.05,0.07) | 0.689073 | 0.24 | 0.002321 | 0 |
| total drinks of alcohol per month | 0.75(0.5,1.11) | 0.14487 | 0.69(0.44,1.08) | 0.101142 | 0.87(0.42,1.8) | 0.703988 | 0(-0.01,0.01) | 0.387547 | 0.66 | 0.002047 | 1 |
| overall oily fish intake | 0.55(0.35,0.86) | 0.009671 | 0.59(0.33,1.03) | 0.064094 | 0.34(0.09,1.25) | 0.102851 | 0.01(-0.01,0.03) | 0.306427 | 0.65 | 0.01149 | 1 |
| overall non-oily fish intake | 0.42(0.21,0.86) | 0.017675 | 0.39(0.17,0.92) | 0.031735 | 0.95(0.2,4.58) | 0.945608 | 0(-0.03,0.02) | 0.701424 | 0.71 | 0.152009 | 0 |
| overall processed meat intake | 1.02(0.53,1.94) | 0.96281 | 1.14(0.51,2.53) | 0.748392 | 0.61(0.11,3.38) | 0.57394 | 0.01(-0.02,0.03) | 0.570164 | 0.99 | 0.951006 | 0 |
| overall poultry intake | 0.88(0.39,2) | 0.76573 | 1.13(0.45,2.84) | 0.799113 | 0.67(0.12,3.79) | 0.65151 | 0.01(-0.02,0.03) | 0.51312 | 1.16 | 0.624571 | 0 |
| overall beef intake | 1.89(1,3.58) | 0.04957 | 1.98(0.96,4.1) | 0.065334 | 1.31(0.31,5.49) | 0.710606 | 0(-0.02,0.02) | 0.868263 | 1.17 | 0.50032 | 0 |
| overall lamb/mutton intake | 0.98(0.55,1.77) | 0.956968 | 0.87(0.45,1.68) | 0.681669 | 1.6(0.44,5.79) | 0.471812 | -0.01(-0.03,0.01) | 0.360227 | 0.91 | 0.623298 | 0 |
| overall pork intake | 0.84(0.42,1.71) | 0.638077 | 0.88(0.42,1.83) | 0.731696 | 0.94(0.24,3.61) | 0.922615 | 0(-0.02,0.02) | 0.875829 | 1.03 | 0.891274 | 0 |
| overall cheese intake | 0.65(0.42,1) | 0.048825 | 0.64(0.36,1.11) | 0.109705 | 0.71(0.26,1.95) | 0.505688 | 0(-0.01,0.01) | 0.961142 | 0.73 | 0.042046 | 0 |
| frequency of adding salt to food | 0.92(0.61,1.39) | 0.688417 | 0.97(0.59,1.61) | 0.920578 | 0.66(0.24,1.79) | 0.411532 | 0.01(-0.01,0.02) | 0.423286 | 0.97 | 0.849796 | 1 |
| temperature of hot drinks | 1.08(0.68,1.71) | 0.742129 | 1.18(0.67,2.06) | 0.563537 | 1.13(0.37,3.41) | 0.828147 | 0(-0.01,0.01) | 0.999685 | 1.13 | 0.443687 | 0 |
| overall alcohol intake | 0.73(0.5,1.08) | 0.115664 | 0.68(0.42,1.08) | 0.099525 | 1.17(0.51,2.67) | 0.716189 | -0.01(-0.02,0) | 0.17061 | 0.7 | 0.007769 | 1 |
| among current drinkers, drinks usually with meals: yes, it varies, no | 0.69(0.41,1.16) | 0.163758 | 0.57(0.31,1.06) | 0.074815 | 0.76(0.19,2.99) | 0.690228 | 0(-0.02,0.02) | 0.856356 | 0.85 | 0.413205 | 1 |
| milk type: skimmed, semi-skimmed, full cream (QT) | 1.32(0.54,3.19) | 0.541147 | 1.49(0.61,3.64) | 0.379906 | 4.1(0.43,39.25) | 0.221088 | -0.02(-0.05,0.01) | 0.163425 | 0.89 | 0.742835 | 1 |

MR: mendelian randomization; MRPRESSO: MR pleiotropy residual sum and outlier; OR: odd ratio; CI: confidence interval; No.: number.

**Table S9. Leave-one-out analysis of association between genetically predicted dietary habits and migraine without aura.**

| **Outcome** | **IVW Estimate**  **[Min, Max] ^a^** | **P value [Min, Max] ^b^** |
| --- | --- | --- |
| alcohol drinker status: current + former vs. never | [0.61,1.41] | [0.520066943368676,0.978357944248832] |
| alcohol drinker status: current vs. never | [0.54,1.23] | [0.446033445287792,0.973704643588789] |
| among current drinkers, drinks usually with meals: yes + it varies vs. no | [0.56,0.63] | [0.00668076026230147,0.0303136083716142] |
| among current drinkers, drinks usually with meals: yes vs. no | [0.72,0.76] | [0.00560284595375239,0.0241076532388896] |
| never eat eggs vs. no eggs, dairy, wheat, or sugar restrictions | [0.63,3.21] | [0.40870676143831,0.994985847814776] |
| never eat eggs vs. no eggs restrictions | [2.11,27.6] | [0.0928635795154376,0.699865242265413] |
| never eat dairy vs. no eggs, dairy, wheat, or sugar restrictions | [0.85,2.4] | [0.442466465771442,0.956761732366207] |
| never eat dairy vs. no dairy restrictions | [0.79,2.91] | [0.453842226062035,0.966783362275377] |
| never eat wheat vs. no eggs, dairy, wheat, or sugar restrictions | [0.33,0.57] | [0.0825584952145393,0.311509761732177] |
| never eat wheat vs. no wheat restrictions | [0.45,0.75] | [0.287049498884305,0.657258351956581] |
| never eat sugar vs. no eggs, dairy, wheat, or sugar restrictions | [0.98,1.1] | [0.651112851159375,0.983576533310409] |
| never eat sugar vs. no sugar restrictions | [1.02,1.13] | [0.556501870345714,0.91559099806977] |
| milk type: dairy-based milk vs. never | [0.17,0.65] | [0.150929899273335,0.758857945613149] |
| milk type: any milk vs. never | [0.31,1.05] | [0.308322229120972,0.967581352085742] |
| milk type: full cream vs. never | [0.93,1] | [0.580067716006385,0.994657858019186] |
| milk type: full cream vs. any other | [1.37,2.04] | [0.201397994182981,0.577579867558037] |
| milk type: semi-skimmed vs. never | [0.18,0.5] | [0.0493981664558917,0.487325593774688] |
| milk type: semi-skimmed vs. any other | [0.66,0.85] | [0.2163846906034,0.628819815300223] |
| milk type: skimmed vs. never | [1.06,1.4] | [0.266265062386896,0.838150358447049] |
| milk type: skimmed vs. any other | [1.33,1.75] | [0.177866240709326,0.46555320938657] |
| milk type: soy milk vs. never | [1.21,1.29] | [0.0257634897436684,0.0941627967307636] |
| milk type: soy milk vs. any other | [0.21,0.44] | [0.0948994640020281,0.379535504490901] |
| milk type: other milk vs. never | [0.67,0.86] | [0.00593107212385473,0.343425337329776] |
| milk type: other milk vs. any other | [0.02,0.63] | [0.115401307314338,0.859633155161142] |
| spread type: all spreads vs. never | [0.75,1] | [0.453153869593521,0.996664520133926] |
| spread type: butter + margarine vs. never | [1.02,1.25] | [0.425905256728908,0.938819656791569] |
| spread type: any oil based spread vs. never | [1.27,1.44] | [0.121060546764209,0.310647538061234] |
| spread type: butter and butter-like spreads vs. oil-based spreads | [0.85,1.04] | [0.554182235933624,0.990135411515162] |
| spread type: butter and margarine spreads vs. oil-based spreads | [1.02,1.16] | [0.509952709196753,0.940634878320541] |
| spread type: butter vs. never | [1.01,1.18] | [0.408852544857272,0.943556341058916] |
| spread type: butter vs. any other | [0.82,0.91] | [0.32086874370388,0.639691593220039] |
| spread type: tub margarine vs. never | [0.98,1.13] | [0.482020067998933,0.982362096617905] |
| spread type: tub margarine vs. any other | [2.07,3.47] | [0.0845526029925649,0.283639647251729] |
| spread type: flora + benecol vs. never | [0.9,1.07] | [0.55345970865783,0.98931049900367] |
| spread type: flora + benecol vs. any other | [0.51,0.75] | [0.153593445085907,0.51536320550929] |
| spread type: olive oil spread vs. never | [1.19,1.35] | [0.112505443620446,0.354072950244076] |
| spread type: olive oil spread vs. any other | [0.33,0.82] | [0.0589704474447358,0.773544042140807] |
| spread type: other oil-based spread vs. never | [1.45,1.61] | [0.00740905140452294,0.0385677541038987] |
| spread type: other oil-based spread vs. any other | [1.17,2.01] | [0.206598513210938,0.773332194980248] |
| spread type: low fat spread vs. never | [0.87,1.03] | [0.503437175165317,0.972070330979347] |
| spread type: low fat spread vs. any other | [0.3,0.66] | [0.195197065738755,0.654016416189068] |
| bread type: white vs. any other | [1.59,1.72] | [0.000821211047855673,0.00421064547637968] |
| bread type: brown vs. any other | [0.96,1.64] | [0.449003173195885,0.965012292386728] |
| bread type: wholemeal/wholegrain vs. any other | [0.61,0.66] | [0.00155916310730957,0.00635677722761276] |
| bread type: white vs. wholemeal/wholegrain + brown | [1.47,1.57] | [0.00278798226398186,0.0113592963717461] |
| bread type: wholemeal/wholegrain vs. white + brown | [0.62,0.65] | [0.00071946797438296,0.00275346454808722] |
| cereal type: biscuit cereal vs. any other | [0.59,0.84] | [0.231280196366811,0.683183495618287] |
| cereal type: bran cereal vs. any other | [0.92,1.95] | [0.22835453496233,0.980106058467544] |
| cereal type: oat cereal vs. any other | [0.51,0.71] | [0.0913413054715388,0.391213998569822] |
| cereal type: muesli vs. any other | [0.54,0.64] | [0.00654354614691411,0.0486258828294137] |
| cereal type: cornflakes/frosties vs. any other | [1.81,2.15] | [0.00122161763907111,0.0125475323283459] |
| coffee type: decaffeinated vs. any other | [0.66,0.71] | [0.0115604455248295,0.0360896195261549] |
| coffee type: ground+instant vs. other+decaff | [0.6,0.88] | [0.172701452050085,0.740865242253016] |
| coffee type: ground vs. any other | [1.08,1.4] | [0.422672494309868,0.855664307352233] |
| tablespoons of cooked vegetables per day | [0.58,0.64] | [0.00417599245852863,0.018950155248182] |
| tablespoons of raw vegetables per day | [0.76,0.83] | [0.139163769834783,0.337661194667435] |
| pieces of fresh fruit per day | [1.08,1.14] | [0.294259562118228,0.540447371402156] |
| pieces of dried fruit per day | [0.82,0.91] | [0.322731316672458,0.63651510051782] |
| slices of bread per week | [1.07,1.18] | [0.388810163460496,0.735622535106398] |
| bowls of cereal per week | [0.67,0.72] | [0.0113645149835403,0.0355464227380159] |
| cups of tea per day | [0.94,1.01] | [0.671619161275052,0.984881328302678] |
| cups of coffee per day | [0.77,0.81] | [0.0732654391701341,0.149632688101672] |
| glasses of water per day | [1.03,1.09] | [0.520899236813193,0.849402314573193] |
| champagne/white wine glasses per month | [0.64,0.75] | [0.0723956210462469,0.238770220176057] |
| red wine glasses per month | [0.52,0.57] | [0.000185134637628171,0.00142755728309517] |
| beer/cider glasses per month | [0.71,0.78] | [0.0810430673123991,0.19996024496325] |
| spirits measures per month | [0.59,0.72] | [0.0833675007935803,0.282064953475015] |
| fortwine glasses per month | [1.12,2.69] | [0.109257435421521,0.827084244034209] |
| other alcohol glasses per month | [0.18,0.28] | [0.000262565454567597,0.00777278244468159] |
| total drinks of alcohol per month | [0.63,0.66] | [0.00050603982107066,0.00264136120223442] |
| overall oily fish intake | [0.63,0.68] | [0.005008131117108,0.0201073932054452] |
| overall non-oily fish intake | [0.65,0.74] | [0.0877352697305721,0.221758894400516] |
| overall processed meat intake | [0.93,1.06] | [0.762143201354656,0.999775568619491] |
| overall poultry intake | [1.07,1.27] | [0.414737447578705,0.827932039704171] |
| overall beef intake | [1.11,1.25] | [0.32464499003231,0.644391947856196] |
| overall lamb/mutton intake | [0.85,0.96] | [0.429330521463085,0.830802069299731] |
| overall pork intake | [0.95,1.11] | [0.667201820530283,0.999175684638043] |
| overall cheese intake | [0.7,0.75] | [0.0219476928211644,0.0574232020637745] |
| frequency of adding salt to food | [0.95,1] | [0.715195405436915,0.997285280600187] |
| temperature of hot drinks | [1.09,1.16] | [0.340484318890439,0.598243519939878] |
| overall alcohol intake | [0.66,0.7] | [0.0022312898977052,0.00819147237940479] |
| among current drinkers, drinks usually with meals: yes, it varies, no | [0.82,0.89] | [0.293104991111574,0.550626708229873] |
| milk type: skimmed, semi-skimmed, full cream (QT) | [0.77,1.01] | [0.448066917316501,0.988216509546381] |

**^a^** the minimum value and maximum value of inverse variance weighted estimate;

**^b^** the minimum value and maximum value of P value;

IVW: inverse variance weighted.

**Table S10. Associations between genetically predicted alcohol consumption, cheese intake and average weekly red wine intake and 3 migraine outcomes as well as 8 risk factors in replication analysis using a second dataset using IVW and MR-PRESSO.**

| **Exposure** | **Outcome** | **N snps** | **IVW** | | | **MRPRESSO** | | |
| --- | --- | --- | --- | --- | --- | --- | --- | --- |
|  |  |  | **OR/Beta** | **95% CI** | **P-value** | **OR/Beta** | **P value** | **No. of outliers** |
| **Alcohol consumption** | migraine | 80 | 0.793932 | [0.57,1.11] | 0.177434 | 0.84 | 0.282303 | 1 |
|  | MA | 80 | 0.846172 | [0.53,1.34] | 0.476722 | 0.85 | 0.478818 | 0 |
|  | MO | 80 | 0.555345 | [0.33,0.92] | 0.022668 | 0.56 | 0.025368 | 0 |
|  | SBP | 82 | 2.064738 | [0.38,3.75] | 0.016421 | 2.41 | 6.33E-06 | 20 |
|  | DBP | 83 | 1.171619 | [0.13,2.21] | 0.02761 | 0.81 | 0.003608 | 22 |
|  | serum total calcium | 84 | -0.00541 | [-0.02,0.01] | 0.466653 | 0 | 0.412322 | 9 |
|  | neuroticism | 80 | 1.03001 | [0.94,1.12] | 0.502062 | 1.02 | 0.513633 | 12 |
|  | difficulty awakening | 84 | 0.947298 | [0.9,1] | 0.04152 | 0.95 | 0.052399 | 3 |
|  | insomnia | 80 | 1.063011 | [0.93,1.22] | 0.370839 | 1.06 | 0.362568 | 4 |
|  | MDD | 83 | 1.068086 | [0.9,1.27] | 0.449519 | 1.05 | 0.5499 | 10 |
|  | anxiety | 83 | 1.107396 | [0.69,1.77] | 0.6692449 | 1.11 | 1.6703646 | 0 |
| **Cheese intake** | migraine | 62 | 0.870329 | [0.63,1.2] | 0.396892 | 0.82 | 0.205049 | 1 |
|  | MA | 62 | 0.868362 | [0.54,1.4] | 0.560336 | 0.8 | 0.327654 | 1 |
|  | MO | 62 | 0.857387 | [0.53,1.38] | 0.527317 | 0.86 | 0.529679 | 0 |
|  | SBP | 65 | -1.73252 | [-3.48,0.01] | 0.051778 | -0.95 | 0.040898 | 13 |
|  | DBP | 65 | -0.58962 | [-1.69,0.51] | 0.291799 | -0.5 | 0.084458 | 12 |
|  | serum total calcium | 64 | -0.00769 | [-0.02,0] | 0.155179 | -0.01 | 0.001148 | 5 |
|  | neuroticism | 61 | 0.96692 | [0.89,1.05] | 0.39979 | 0.92 | 0.032361 | 4 |
|  | difficulty awakening | 64 | 0.945426 | [0.9,0.99] | 0.019058 | 0.95 | 0.022224 | 1 |
|  | insomnia | 61 | 0.889964 | [0.77,1.02] | 0.102327 | 0.96 | 0.471566 | 3 |
|  | MDD | 61 | 0.838846 | [0.75,0.94] | 0.003363 | 0.82 | 0.000891 | 1 |
|  | anxiety | 63 | 0.738489 | [0.45,1.21] | 0.232316 | 0.74 | 0.236865 | 0 |
| **Average weekly red wine intake** | migraine | 72 | 0.550666 | [0.4,0.75] | 0.000201 | 0.55 | 0.000398 | 0 |
|  | MA | 72 | 0.648272 | [0.41,1.03] | 0.067864 | 0.65 | 0.072069 | 0 |
|  | MO | 72 | 0.51954 | [0.32,0.83] | 0.006615 | 0.52 | 0.002667 | 0 |
|  | SBP | 64 | -0.04424 | [-1.64,1.56] | 0.95679 | -0.06 | 0.897049 | 11 |
|  | DBP | 67 | -0.5504 | [-1.48,0.38] | 0.244364 | -0.39 | 0.197251 | 7 |
|  | serum total calcium | 73 | -0.0063 | [-0.01,0] | 0.074921 | -0.01 | 0.000266 | 4 |
|  | neuroticism | 65 | 0.947355 | [0.86,1.04] | 0.254664 | 0.96 | 0.255612 | 5 |
|  | difficulty awakening | 73 | 0.976607 | [0.91,1.04] | 0.481869 | 0.97 | 0.235566 | 5 |
|  | insomnia | 65 | 0.899801 | [0.78,1.04] | 0.158689 | 0.9 | 0.124872 | 2 |
|  | MDD | 71 | 0.984928 | [0.85,1.14] | 0.83921 | 0.96 | 0.560986 | 3 |
|  | anxiety | 67 | 0.762497 | [0.43,1.35] | 0.35033 | 0.83 | 0.509102 | 1 |

MA: migraine with aura; MO: migraine without aura; SBP: systolic blood pressure (SBP); DBP: diastolic blood pressure; MDD: Major depression disorder; IVW: inverse variance weighted; MR: mendelian randomization; MRPRESSO: MR pleiotropy residual sum and outlier; OR: odd ratio; CI: confidence interval.

**Table S11. Associations between genetically predicted alcohol consumption, cheese intake and average weekly red wine intake and 3 migraine outcomes as well as 8 risk factors in replication analysis using a second dataset using other sensitivity analysis methods.**

| **Exposure** | **Outcome** | **Weighted median** | | **Mode-based** | | **MR-Egger** | | | |
| --- | --- | --- | --- | --- | --- | --- | --- | --- | --- |
|  |  | **OR/Beta (95% CI)** | **P value** | **OR/Beta (95% CI)** | **P value** | **OR/Beta (95% CI)** | **P value** | **Intercept** | **P value** |
| **Alcohol consumption** | migraine | 0.95(0.6,1.53) | 0.842438 | 0.99(0.63,1.56) | 0.973801 | 0.76(0.33,1.73) | 0.513204 | 0(-0.01,0.01) | 0.907297 |
|  | MA | 0.93(0.47,1.87) | 0.84829 | 0.95(0.48,1.88) | 0.889757 | 0.83(0.27,2.58) | 0.7441 | 0(-0.01,0.02) | 0.966398 |
|  | MO | 0.7(0.33,1.5) | 0.36264 | 0.72(0.35,1.46) | 0.359443 | 0.68(0.2,2.38) | 0.550624 | 0(-0.02,0.01) | 0.719741 |
|  | SBP | 3.04(2.13,3.94) | 4.26E-11 | 2.25(1.48,3.02) | 9.97E-09 | 4.33(1.47,7.2) | 0.002998 | -0.04(-0.09,0) | 0.056468 |
|  | DBP | 0.46(-0.06,0.98) | 0.080323 | 0.41(0.01,0.81) | 0.046219 | 1.53(-0.29,3.34) | 0.099826 | -0.01(-0.03,0.02) | 0.640283 |
|  | serum total calcium | 0(0,0.01) | 0.371436 | 0.01(0,0.01) | 0.001166 | 0.01(-0.02,0.04) | 0.522151 | 0(0,0) | 0.218234 |
|  | neuroticism | 0.98(0.92,1.05) | 0.61831 | 0.98(0.93,1.03) | 0.520446 | 0.96(0.83,1.11) | 0.554601 | 0(0,0) | 0.222134 |
|  | difficulty awakening | 1(0.95,1.06) | 0.991575 | 0.97(0.93,1.02) | 0.22566 | 0.98(0.89,1.08) | 0.712196 | 0(0,0) | 0.383274 |
|  | insomnia | 1.05(0.9,1.22) | 0.513179 | 1.04(0.92,1.17) | 0.538974 | 1.02(0.8,1.29) | 0.901413 | 0(0,0) | 0.640758 |
|  | MDD | 0.88(0.74,1.03) | 0.116759 | 0.89(0.77,1.03) | 0.110411 | 0.69(0.42,1.14) | 0.147344 | 0.01(0,0.01) | 0.069906 |
|  | anxiety | 0.67(0.34,1.35) | 0.263996 | 0.71(0.39,1.29) | 0.258137 | 0.44(0.18,1.06) | 0.065652 | 0.02(0,0.03) | 0.015898 |
| **Cheese intake** | migraine | 0.82(0.55,1.24) | 0.350487 | 1(0.63,1.6) | 0.987461 | 1.12(0.28,4.49) | 0.872713 | 0(-0.03,0.02) | 0.713967 |
|  | MA | 1.31(0.72,2.39) | 0.376086 | 1.03(0.54,1.95) | 0.925699 | 0.48(0.06,3.72) | 0.482641 | 0.01(-0.02,0.04) | 0.559844 |
|  | MO | 0.76(0.41,1.42) | 0.390882 | 0.59(0.29,1.19) | 0.13889 | 0.98(0.12,7.71) | 0.985846 | 0(-0.04,0.03) | 0.894813 |
|  | SBP | -1.36(-2.37,-0.35) | 0.008152 | -0.31(-1.27,0.65) | 0.53056 | -0.35(-7.69,6.99) | 0.92517 | -0.02(-0.15,0.1) | 0.704034 |
|  | DBP | -0.52(-1.12,0.07) | 0.084979 | -0.34(-0.89,0.22) | 0.234612 | 1(-3.59,5.59) | 0.670574 | -0.03(-0.1,0.05) | 0.485536 |
|  | serum total calcium | -0.01(-0.01,0) | 0.146245 | -0.01(-0.01,0) | 0.102694 | 0(-0.04,0.05) | 0.92287 | 0(0,0) | 0.65223 |
|  | neuroticism | 0.95(0.89,1.02) | 0.164438 | 0.91(0.85,0.98) | 0.018089 | 0.89(0.65,1.23) | 0.491428 | 0(0,0.01) | 0.618954 |
|  | difficulty awakening | 0.99(0.94,1.04) | 0.633973 | 0.99(0.94,1.05) | 0.815014 | 1.03(0.85,1.25) | 0.788582 | 0(0,0) | 0.391061 |
|  | insomnia | 0.93(0.8,1.07) | 0.303284 | 0.94(0.8,1.1) | 0.425044 | 0.62(0.35,1.1) | 0.104114 | 0.01(0,0.02) | 0.206687 |
|  | MDD | 0.8(0.7,0.91) | 0.000735 | 0.8(0.69,0.94) | 0.005895 | 0.54(0.34,0.87) | 0.012032 | 0.01(0,0.02) | 0.065368 |
|  | anxiety | 0.66(0.33,1.31) | 0.234216 | 0.58(0.27,1.27) | 0.172559 | 0.39(0.05,3.13) | 0.373444 | 0.01(-0.02,0.05) | 0.065368 |
| **Average weekly red wine intake** | migraine | 0.57(0.37,0.89) | 0.013615 | 0.67(0.42,1.08) | 0.09944 | 1.59(0.56,4.53) | 0.387069 | -0.02(-0.03,0) | 0.532876 |
|  | MA | 0.73(0.37,1.42) | 0.350744 | 0.83(0.39,1.78) | 0.635897 | 1.67(0.34,8.12) | 0.522715 | -0.01(-0.04,0.01) | 0.218198 |
|  | MO | 0.59(0.3,1.16) | 0.128929 | 0.63(0.3,1.31) | 0.219603 | 1.05(0.21,5.27) | 0.950136 | -0.01(-0.03,0.01) | 0.368453 |
|  | SBP | 0(-1.09,1.08) | 0.993753 | -0.14(-1.25,0.96) | 0.80029 | 4.09(-1.41,9.58) | 0.145068 | -0.06(-0.14,0.02) | 0.124015 |
|  | DBP | 0.03(-0.59,0.66) | 0.917678 | -0.02(-0.67,0.63) | 0.957732 | 0.1(-2.96,3.16) | 0.94828 | -0.01(-0.05,0.03) | 0.660842 |
|  | serum total calcium | -0.01(-0.02,0) | 0.015467 | -0.01(-0.02,0) | 0.006476 | 0.01(-0.02,0.03) | 0.62322 | 0(0,0) | 0.26327 |
|  | neuroticism | 0.98(0.91,1.06) | 0.621974 | 1.01(0.93,1.09) | 0.852263 | 1.06(0.8,1.42) | 0.67608 | 0(-0.01,0) | 0.404514 |
|  | difficulty awakening | 0.98(0.93,1.03) | 0.368463 | 0.97(0.92,1.02) | 0.284074 | 0.96(0.78,1.18) | 0.676858 | 0(0,0) | 0.837862 |
|  | insomnia | 0.95(0.81,1.12) | 0.574779 | 0.97(0.81,1.15) | 0.713336 | 1.26(0.8,1.99) | 0.313702 | -0.01(-0.01,0) | 0.122257 |
|  | MDD | 1.05(0.91,1.22) | 0.503834 | 1.03(0.88,1.21) | 0.715951 | 1.14(0.68,1.91) | 0.615981 | 0(-0.01,0.01) | 0.55969 |
|  | anxiety | 0.67(0.32,1.39) | 0.276666 | 0.58 (0.25,1.31) | 0.186282 | 1.02(0.12,8.64) | 0.985199 | 0(-0.03,0.03) | 0.781399 |

MA: migraine with aura; MO: migraine without aura; SBP: systolic blood pressure (SBP); DBP: diastolic blood pressure; MDD: Major depression disorder.

**Table S12. The causal analysis results between the dietary habits and principal components (PCs) using IVW.**

| **Exposure** | **Number of SNPs** | ***OR* (95% *CI*)** | ***P* value** | Alpha |
| --- | --- | --- | --- | --- |
| **Replication analysis for 83 dietary habits as well as 60 PCs** | | | | |
| Among current drinkers, drinks usually with meals: yes vs. no | 8 | 0.52 (0.3, 0.92) | 0.02 | 0.0038 |
| PC1 (type of bread consumed: wholegrain/ wholemeal vs. white bread +) | 48 | 0.79 (0.67, 0.93) | 0.004 | 0.05 |
| Cups of coffee per day | 17 | 0.55 (0.38, 0.8) | 0.002 | 0.0125 |
| PC14 (cups of tea per day -) | 2 | 0.15 (0.03, 0.77) | 0.02 | 0.05 |
| Overall non-oily fish intake | 2 | 0.15 (0.03, 0.83) | 0.03 | 0.025 |
| PC13 (overall poultry intake -) | 4 | 0.3 (0.11, 0.83) | 0.02 | 0.05 |
| **Replication analysis for 29 food phenotypes and their PCs using SNPs after filtering** | | | | |
| Coffee PC | 6 | 0.73 (0.61,0.89) | 0.00136 | 0.0125 |
| Decaf. Coffee | 2 | 0.09 (0.02,0.53) | 0.007891 | 0.0125 |
| Ground Coffee | 3 | 0.16 (0.05,0.51) | 0.002153 | 0.0125 |
| Instant Coffee | 6 | 0.16 (0.05,0.5) | 0.001326 | 0.0125 |
| Oily Fish | 16 | 0.28 (0.14,0.57) | 0.000452 | 0.0167 |
| Overall unhealthy diet PC | 6 | 0.56 (0.32,1) | 0.048631 | 0.05 |
| Tea | 17 | 0.23 (0.08,0.67) | 0.006511 | 0.05 |
| **Replication analysis for 29 food phenotypes and their PCs using SNPs before filtering** | | | | |
| Coffee PC | 10 | 0.76 (0.63,0.92) | 0.005167 | 0.0125 |
| Decaf. Coffee | 2 | 0.09 (0.02,0.53) | 0.007891 | 0.0125 |
| Fish PC | 18 | 0.54 (0.33,0.88) | 0.013911 | 0.0167 |
| Ground Coffee | 3 | 0.16 (0.05,0.51) | 0.002153 | 0.0125 |
| Instant Coffee | 8 | 0.20 (0.06,0.68) | 0.010013 | 0.0125 |
| Oily Fish | 32 | 0.30 (0.17,0.53) | 2.44E-05 | 0.0167 |
| Psychoactive drinks PC | 12 | 0.70 (0.55,0.9) | 0.00557 | 0.05 |
| Tea | 17 | 0.23 (0.08,0.67) | 0.006511 | 0.05 |
| Vegetable PC | 15 | 0.47 (0.23,0.94) | 0.033035 | 0.025 |

Alpha: Bonferroni-corrected threshold in this subtype;

**Table S13. Associations between genetically predicted dietary habits and SBP, DBP and serum total calcium using IVW.**

| **Exposure** | **SBP** | | | | | **DBP** | | | | | **Serum total calcium** | | | | |
| --- | --- | --- | --- | --- | --- | --- | --- | --- | --- | --- | --- | --- | --- | --- | --- |
|  | **N snps** | **OR** | **95% CI** | **P value** | **I^2^** | **N snps** | **OR** | **95% CI** | **P value** | **I^2^** | **N snps** | **OR** | **95% CI** | **P value** | **I^2^** |
| alcohol drinker status: current + former vs. never | 34 | 2.149528 | [-1.76,6.06] | 0.281171 | 76.50931 | 34 | -0.68522 | [-2.67,1.3] | 0.49875 | 69.8582 | 45 | 0.012207 | [-0.01,0.03] | 0.27387 | 64.83634 |
| alcohol drinker status: current vs. never | 34 | 1.229303 | [-3.11,5.57] | 0.579127 | 82.31505 | 34 | -1.00822 | [-2.94,0.92] | 0.30613 | 70.38859 | 45 | 0.011033 | [-0.01,0.03] | 0.30681 | 64.95692 |
| among current drinkers, drinks usually with meals: yes + it varies vs. no | 97 | -0.64272 | [-1.94,0.65] | 0.330819 | 83.83349 | 98 | -0.6347 | [-1.38,0.11] | 0.093138 | 83.84625 | 122 | -0.00375 | [-0.01,0] | 0.147257 | 50.17592 |
| among current drinkers, drinks usually with meals: yes vs. no | 145 | 0.075216 | [-0.67,0.82] | 0.843122 | 82.54603 | 147 | -0.13938 | [-0.57,0.29] | 0.527005 | 83.06447 | 176 | -0.00231 | [-0.01,0] | 0.160641 | 54.75063 |
| never eat eggs vs. no eggs, dairy, wheat, or sugar restrictions | 9 | 7.160426 | [1.1,13.22] | 0.020483 | 66.52392 | 10 | 3.876265 | [-1.45,9.2] | 0.153424 | 86.93765 | 13 | 0.005396 | [-0.02,0.03] | 0.632753 | 0 |
| never eat eggs vs. no eggs restrictions | 6 | 6.439469 | [-3.38,16.26] | 0.198861 | 71.81081 | 7 | 1.691997 | [-5.67,9.05] | 0.652355 | 85.42343 | 8 | -0.00197 | [-0.04,0.03] | 0.910705 | 0.388774 |
| never eat dairy vs. no eggs, dairy, wheat, or sugar restrictions | 13 | 0.321772 | [-6.02,6.66] | 0.920759 | 76.45723 | 13 | -1.91027 | [-4.79,0.97] | 0.193161 | 62.22412 | 17 | -0.0098 | [-0.04,0.02] | 0.536478 | 57.04557 |
| never eat dairy vs. no dairy restrictions | 13 | 0.373646 | [-7.52,8.27] | 0.926069 | 76.37735 | 13 | -2.24044 | [-5.73,1.24] | 0.207649 | 59.94447 | 16 | -0.01322 | [-0.05,0.03] | 0.527684 | 59.49733 |
| never eat wheat vs. no eggs, dairy, wheat, or sugar restrictions | 17 | -1.78927 | [-3.84,0.26] | 0.086974 | 64.30838 | 17 | -7.67288 | [-9.93,-5.42] | 2.56E-11 | 90.45501 | 25 | -0.02276 | [-0.04,-0.01] | 0.003133 | 76.44199 |
| never eat wheat vs. no wheat restrictions | 18 | -2.63104 | [-4.19,-1.08] | 0.000916 | 9.57983 | 18 | -9.47388 | [-11.79,-7.16] | 1.00E-15 | 86.75784 | 27 | -0.02628 | [-0.04,-0.01] | 0.00433 | 76.22714 |
| never eat sugar vs. no eggs, dairy, wheat, or sugar restrictions | 117 | 0.784805 | [-0.75,2.32] | 0.316623 | 88.34002 | 118 | -0.11342 | [-1.02,0.79] | 0.805271 | 88.91073 | 137 | 0.001295 | [-0.01,0.01] | 0.702836 | 69.38208 |
| never eat sugar vs. no sugar restrictions | 115 | 0.557534 | [-1.07,2.18] | 0.500646 | 88.8022 | 115 | -0.27504 | [-1.23,0.68] | 0.573409 | 89.39254 | 135 | 0.004062 | [0,0.01] | 0.245272 | 68.93011 |
| milk type: dairy-based milk vs. never | 15 | 0.402907 | [-4.8,5.61] | 0.879351 | 71.7433 | 15 | -0.76121 | [-3.76,2.24] | 0.618779 | 71.832 | 22 | 0.009635 | [-0.01,0.03] | 0.306105 | 8.830977 |
| milk type: any milk vs. never | 15 | -0.91647 | [-5.88,4.05] | 0.717644 | 65.99546 | 15 | -0.74688 | [-3.76,2.26] | 0.62662 | 69.35944 | 22 | 0.008864 | [-0.01,0.03] | 0.366671 | 8.603441 |
| milk type: full cream vs. never | 21 | 0.407737 | [-0.04,0.85] | 0.072542 | 32.68993 | 21 | 0.221026 | [-0.02,0.47] | 0.078148 | 27.29303 | 27 | 0.002899 | [0,0.01] | 0.064491 | 35.80829 |
| milk type: full cream vs. any other | 31 | 2.21674 | [-1.66,6.09] | 0.262458 | 85.08811 | 31 | 1.937234 | [-0.43,4.3] | 0.108714 | 86.816 | 36 | -0.00671 | [-0.03,0.01] | 0.474668 | 65.96346 |
| milk type: semi-skimmed vs. never | 15 | 0.43769 | [-3.35,4.22] | 0.820647 | 70.93498 | 15 | -0.44695 | [-2.8,1.91] | 0.709671 | 75.15576 | 22 | 0.005023 | [-0.01,0.02] | 0.579448 | 46.1608 |
| milk type: semi-skimmed vs. any other | 25 | -0.04057 | [-1.98,1.9] | 0.967251 | 71.28755 | 25 | -0.68462 | [-1.61,0.24] | 0.146368 | 58.35363 | 32 | 0.003445 | [-0.01,0.01] | 0.459535 | 40.4647 |
| milk type: skimmed vs. never | 20 | -0.26148 | [-1.53,1] | 0.685191 | 66.22822 | 20 | -0.13334 | [-0.82,0.55] | 0.701736 | 61.7688 | 26 | 0.008003 | [0,0.01] | 0.001427 | 0.277052 |
| milk type: skimmed vs. any other | 35 | -0.5438 | [-3.23,2.14] | 0.691417 | 87.47783 | 35 | -0.77823 | [-2.29,0.74] | 0.314053 | 87.01237 | 46 | -0.00161 | [-0.01,0.01] | 0.79993 | 73.40584 |
| milk type: soy milk vs. never | 23 | 0.198605 | [-0.16,0.55] | 0.273641 | 34.60345 | 23 | 0.192501 | [-0.18,0.56] | 0.309657 | 80.26135 | 28 | 0.001144 | [0,0] | 0.494138 | 64.21169 |
| milk type: soy milk vs. any other | 19 | -1.06095 | [-5.05,2.93] | 0.602031 | 60.40519 | 19 | 0.057528 | [-1.76,1.87] | 0.950518 | 37.51652 | 25 | -0.02977 | [-0.07,0.01] | 0.107755 | 79.37033 |
| milk type: other milk vs. never | 11 | 0.249001 | [-0.4,0.9] | 0.452213 | 67.5419 | 11 | 0.29144 | [0.03,0.55] | 0.028705 | 34.14017 | 14 | 0.000255 | [0,0] | 0.871066 | 36.11431 |
| milk type: other milk vs. any other | 8 | 3.226967 | [-4.97,11.43] | 0.440517 | 39.83062 | 9 | 2.139701 | [-1.65,5.93] | 0.268618 | 17.87347 | 14 | 0.004942 | [-0.03,0.04] | 0.78441 | 0 |
| spread type: all spreads vs. never | 45 | 2.380646 | [0.36,4.4] | 0.020803 | 72.20115 | 45 | 1.841407 | [0.7,2.98] | 0.0015 | 71.10199 | 53 | 0.008287 | [0,0.02] | 0.114133 | 46.51831 |
| spread type: butter + margarine vs. never | 51 | 2.150961 | [0.73,3.58] | 0.003101 | 78.04978 | 51 | 1.871745 | [1.03,2.71] | 1.26E-05 | 79.17092 | 59 | 0.005419 | [0,0.01] | 0.146188 | 57.81994 |
| spread type: any oil based spread vs. never | 30 | 0.708134 | [-1.04,2.46] | 0.428061 | 86.7181 | 30 | 0.550969 | [-0.69,1.79] | 0.382513 | 91.20181 | 34 | 0.004783 | [0,0.01] | 0.042535 | 0 |
| spread type: butter and butter-like spreads vs. oil-based spreads | 36 | -0.58866 | [-3.64,2.46] | 0.705346 | 93.78833 | 36 | 0.911972 | [-1.31,3.13] | 0.421001 | 96.14112 | 47 | 0.001762 | [-0.01,0.01] | 0.65342 | 55.49813 |
| spread type: butter and margarine spreads vs. oil-based spreads | 48 | -1.40198 | [-3.78,0.97] | 0.247573 | 93.66246 | 48 | 0.478144 | [-1.14,2.09] | 0.562011 | 95.48286 | 58 | 0.003748 | [0,0.01] | 0.289005 | 63.34141 |
| spread type: butter vs. never | 51 | 1.331839 | [0.21,2.45] | 0.019454 | 78.34403 | 51 | 1.369406 | [0.81,1.93] | 1.87E-06 | 71.89348 | 61 | 0.004447 | [0,0.01] | 0.154492 | 64.40941 |
| spread type: butter vs. any other | 90 | -1.47461 | [-3.27,0.32] | 0.107517 | 91.69869 | 90 | -0.40232 | [-1.57,0.76] | 0.497753 | 93.47494 | 105 | -0.00041 | [-0.01,0.01] | 0.915628 | 77.87158 |
| spread type: tub margarine vs. never | 24 | 0.417031 | [-0.74,1.57] | 0.478925 | 85.2584 | 24 | 0.433334 | [-0.13,1] | 0.13311 | 79.77322 | 30 | 0.000899 | [0,0.01] | 0.688976 | 53.77632 |
| spread type: tub margarine vs. any other | 24 | 1.081904 | [-2.36,4.53] | 0.538009 | 72.14279 | 25 | 0.915011 | [-0.64,2.47] | 0.250065 | 56.85573 | 29 | 0.006559 | [-0.02,0.03] | 0.563151 | 67.56469 |
| spread type: flora + benecol vs. never | 26 | 1.45002 | [0.29,2.61] | 0.014431 | 85.73227 | 26 | 0.256831 | [-0.53,1.04] | 0.521715 | 89.72665 | 31 | 0.004034 | [0,0.01] | 0.15359 | 69.38707 |
| spread type: flora + benecol vs. any other | 38 | 2.825381 | [0.45,5.2] | 0.019695 | 78.4882 | 38 | -0.52599 | [-2.12,1.07] | 0.518125 | 84.28796 | 43 | 0.003597 | [-0.01,0.02] | 0.691505 | 80.10325 |
| spread type: olive oil spread vs. never | 20 | 1.246492 | [-0.06,2.55] | 0.06159 | 82.73481 | 22 | 0.651865 | [-0.02,1.32] | 0.057143 | 79.93356 | 26 | 0.004482 | [0,0.01] | 0.141828 | 62.45847 |
| spread type: olive oil spread vs. any other | 14 | 0.170703 | [-6.13,6.47] | 0.957656 | 91.17748 | 14 | -0.16306 | [-2.87,2.55] | 0.906146 | 84.29545 | 17 | 0.014405 | [0,0.03] | 0.08706 | 39.38593 |
| spread type: other oil-based spread vs. never | 36 | 0.386357 | [-0.74,1.51] | 0.501882 | 83.35612 | 36 | 0.304765 | [-0.51,1.12] | 0.464565 | 89.51361 | 40 | 0.002602 | [0,0.01] | 0.128021 | 0 |
| spread type: other oil-based spread vs. any other | 22 | 2.752945 | [-0.16,5.67] | 0.064025 | 79.14983 | 22 | 0.113167 | [-1.81,2.04] | 0.908377 | 84.29883 | 26 | -0.00456 | [-0.02,0.01] | 0.506071 | 51.48708 |
| spread type: low fat spread vs. never | 10 | -0.64605 | [-2.13,0.84] | 0.393465 | 80.28056 | 10 | -0.06992 | [-0.49,0.35] | 0.742314 | 17.42772 | 14 | 0.003365 | [0,0.01] | 0.188735 | 24.89634 |
| spread type: low fat spread vs. any other | 12 | -5.6235 | [-17.98,6.74] | 0.372548 | 95.20123 | 12 | -5.2941 | [-14.39,3.81] | 0.254168 | 97.08469 | 18 | -0.04187 | [-0.1,0.01] | 0.134092 | 90.26915 |
| bread type: white vs. any other | 157 | 2.14263 | [1.25,3.04] | 2.71E-06 | 79.3184 | 157 | 1.255401 | [0.76,1.75] | 8.21E-07 | 78.05353 | 177 | 0.009134 | [0,0.01] | 6.15E-05 | 56.36169 |
| bread type: brown vs. any other | 17 | -2.3405 | [-6.33,1.65] | 0.249865 | 81.50328 | 17 | -1.00866 | [-2.94,0.92] | 0.30485 | 73.94411 | 22 | -0.00802 | [-0.02,0.01] | 0.310701 | 46.79506 |
| bread type: wholemeal/wholegrain vs. any other | 125 | -1.62752 | [-2.68,-0.58] | 0.002371 | 84.22771 | 126 | -0.90769 | [-1.47,-0.35] | 0.001531 | 81.9269 | 159 | -0.00773 | [-0.01,0] | 0.000498 | 56.99978 |
| bread type: white vs. wholemeal/wholegrain + brown | 170 | 1.697737 | [0.88,2.52] | 4.98E-05 | 78.50446 | 169 | 1.088548 | [0.63,1.54] | 2.58E-06 | 76.70783 | 193 | 0.007968 | [0,0.01] | 0.00013 | 54.68479 |
| bread type: wholemeal/wholegrain vs. white + brown | 149 | -1.39545 | [-2.32,-0.47] | 0.002987 | 83.21787 | 151 | -1.09879 | [-1.6,-0.6] | 1.51E-05 | 81.21853 | 182 | -0.00811 | [-0.01,0] | 0.000554 | 67.37145 |
| cereal type: biscuit cereal vs. any other | 25 | 1.530207 | [-1.05,4.11] | 0.244342 | 80.60927 | 25 | 1.390053 | [-0.44,3.22] | 0.135724 | 87.22896 | 30 | 0.005886 | [-0.01,0.02] | 0.358223 | 59.71713 |
| cereal type: bran cereal vs. any other | 18 | -2.30915 | [-5.27,0.65] | 0.126224 | 78.19805 | 18 | -1.48047 | [-3.44,0.48] | 0.138766 | 83.70035 | 20 | -0.0073 | [-0.02,0.01] | 0.327008 | 54.88114 |
| cereal type: oat cereal vs. any other | 21 | -0.06914 | [-2.31,2.17] | 0.951841 | 76.44882 | 23 | -0.20091 | [-1.59,1.19] | 0.777199 | 81.37064 | 30 | -0.00071 | [-0.01,0.01] | 0.894525 | 55.63331 |
| cereal type: muesli vs. any other | 85 | -1.86628 | [-3.39,-0.34] | 0.01667 | 86.41315 | 87 | -0.65889 | [-1.82,0.5] | 0.263999 | 92.31525 | 104 | -0.00481 | [-0.01,0] | 0.203888 | 72.55696 |
| cereal type: cornflakes/frosties vs. any other | 68 | 2.156698 | [0.84,3.47] | 0.001276 | 77.644 | 68 | -0.12204 | [-1.2,0.96] | 0.824265 | 89.09224 | 84 | 0.000917 | [-0.01,0.01] | 0.803639 | 66.86993 |
| coffee type: decaffeinated vs. any other | 138 | -1.92898 | [-2.97,-0.88] | 0.000293 | 85.10687 | 138 | -0.89147 | [-1.69,-0.09] | 0.028475 | 91.58861 | 168 | -0.00403 | [-0.01,0] | 0.109142 | 67.18893 |
| coffee type: ground+instant vs. other+decaff | 31 | 0.684348 | [-2.51,3.88] | 0.674818 | 91.77033 | 32 | 1.508299 | [-0.68,3.69] | 0.176289 | 94.36231 | 38 | 0.011706 | [0,0.03] | 0.151291 | 84.47986 |
| coffee type: ground vs. any other | 28 | -1.97529 | [-5.31,1.36] | 0.245105 | 91.15041 | 28 | -2.43001 | [-4.85,-0.01] | 0.048763 | 94.48084 | 34 | -0.01425 | [-0.03,0] | 0.106465 | 84.41361 |
| tablespoons of cooked vegetables per day | 125 | -0.37911 | [-1.72,0.96] | 0.580122 | 86.70515 | 126 | -0.20813 | [-0.95,0.53] | 0.5803 | 85.53684 | 139 | -0.00383 | [-0.01,0] | 0.153037 | 54.35566 |
| tablespoons of raw vegetables per day | 142 | -0.01434 | [-0.94,0.92] | 0.97589 | 76.26662 | 143 | 0.445435 | [-0.25,1.14] | 0.211306 | 86.20722 | 163 | -0.00154 | [-0.01,0] | 0.525616 | 54.69344 |
| pieces of fresh fruit per day | 245 | -0.42418 | [-1.28,0.43] | 0.330547 | 85.88861 | 247 | -0.37804 | [-0.83,0.07] | 0.101005 | 83.46843 | 284 | -0.0056 | [-0.01,0] | 0.003553 | 62.32089 |
| pieces of dried fruit per day | 115 | -1.0565 | [-2.04,-0.08] | 0.03467 | 74.67525 | 116 | -0.43019 | [-1.05,0.19] | 0.171392 | 79.00966 | 134 | -0.01045 | [-0.02,0] | 0.000511 | 64.37515 |
| slices of bread per week | 126 | 0.724459 | [-0.42,1.87] | 0.215562 | 82.76846 | 127 | 0.268508 | [-0.4,0.94] | 0.434384 | 83.60715 | 149 | 0.003311 | [0,0.01] | 0.351022 | 76.60009 |
| bowls of cereal per week | 169 | 0.319402 | [-0.64,1.28] | 0.515317 | 82.55157 | 169 | -0.06937 | [-0.72,0.58] | 0.834653 | 87.4316 | 188 | 0.001425 | [0,0.01] | 0.613662 | 71.95134 |
| cups of tea per day | 167 | -0.09148 | [-0.98,0.8] | 0.840591 | 81.49333 | 167 | -0.30771 | [-0.86,0.24] | 0.274989 | 84.11248 | 201 | 0.006741 | [0,0.01] | 0.003814 | 64.78725 |
| cups of coffee per day | 123 | -1.3942 | [-2.63,-0.16] | 0.0273 | 90.02004 | 124 | -1.29857 | [-2.05,-0.55] | 0.00073 | 91.15387 | 155 | -0.00467 | [-0.01,0] | 0.170521 | 83.28813 |
| glasses of water per day | 255 | 0.09863 | [-0.68,0.88] | 0.803865 | 82.6499 | 257 | 0.232471 | [-0.28,0.74] | 0.369963 | 86.65264 | 294 | -0.00297 | [-0.01,0] | 0.227226 | 76.71695 |
| champagne/white wine glasses per month | 67 | 0.596046 | [-1.28,2.47] | 0.534037 | 89.54997 | 67 | 0.57193 | [-0.58,1.72] | 0.328747 | 90.7552 | 82 | -0.00307 | [-0.02,0.01] | 0.646407 | 89.29233 |
| red wine glasses per month | 130 | -0.41723 | [-1.59,0.76] | 0.485896 | 86.76867 | 132 | -0.28087 | [-0.96,0.4] | 0.418261 | 87.11097 | 153 | -0.00366 | [-0.01,0] | 0.294016 | 79.75127 |
| beer/cider glasses per month | 109 | 2.09184 | [0.88,3.31] | 0.000735 | 86.07344 | 110 | 1.104594 | [0.33,1.88] | 0.005362 | 88.83333 | 127 | 0.005658 | [0,0.01] | 0.235038 | 87.72943 |
| spirits measures per month | 39 | -1.2994 | [-3.68,1.08] | 0.284252 | 86.57463 | 40 | -0.6187 | [-1.55,0.31] | 0.19052 | 71.39447 | 47 | 0.001544 | [-0.01,0.01] | 0.793321 | 72.21727 |
| fortwine glasses per month | 10 | 1.682365 | [-0.35,3.71] | 0.104702 | 27.79599 | 10 | 0.890327 | [-0.62,2.4] | 0.247087 | 56.49318 | 17 | 0.004361 | [-0.01,0.02] | 0.515508 | 39.54078 |
| other alcohol glasses per month | 8 | -1.49657 | [-3.58,0.58] | 0.158189 | 55.22284 | 8 | -1.39476 | [-2.19,-0.6] | 0.000592 | 0 | 12 | -0.00549 | [-0.01,0] | 0.208274 | 0 |
| total drinks of alcohol per month | 271 | 0.318006 | [-0.48,1.12] | 0.435084 | 86.8713 | 271 | 0.243094 | [-0.2,0.68] | 0.276882 | 85.57259 | 317 | -0.00181 | [-0.01,0] | 0.448664 | 79.62798 |
| overall oily fish intake | 183 | 0.479625 | [-0.42,1.38] | 0.297952 | 83.00244 | 183 | 0.745517 | [0.14,1.35] | 0.015814 | 87.52163 | 217 | 0.001847 | [0,0.01] | 0.5214 | 78.09724 |
| overall non-oily fish intake | 71 | 1.720531 | [0.26,3.18] | 0.021034 | 79.67796 | 72 | 1.640705 | [0.54,2.74] | 0.003436 | 88.28247 | 83 | 0.006108 | [-0.01,0.02] | 0.333495 | 85.78305 |
| overall processed meat intake | 106 | -0.67272 | [-2.19,0.84] | 0.384336 | 87.29828 | 108 | -0.35876 | [-1.11,0.39] | 0.348223 | 83.15877 | 127 | 0.001047 | [0,0.01] | 0.703086 | 50.60044 |
| overall poultry intake | 55 | 0.793765 | [-0.98,2.57] | 0.379917 | 80.6893 | 57 | -0.07555 | [-1.08,0.93] | 0.882406 | 80.60466 | 68 | 0.014824 | [0,0.03] | 0.034395 | 84.83994 |
| overall beef intake | 100 | 0.903602 | [-0.27,2.07] | 0.130093 | 77.39905 | 101 | 0.636479 | [-0.03,1.31] | 0.062498 | 77.44755 | 115 | 0.006819 | [0,0.01] | 0.045937 | 64.53437 |
| overall lamb/mutton intake | 113 | -1.18421 | [-2.48,0.11] | 0.073358 | 84.45998 | 115 | -0.53037 | [-1.24,0.18] | 0.141644 | 82.99774 | 134 | 0.003367 | [0,0.01] | 0.231435 | 57.63759 |
| overall pork intake | 71 | -0.62471 | [-2.2,0.95] | 0.435589 | 82.43874 | 71 | -0.08919 | [-0.89,0.72] | 0.828045 | 77.90647 | 83 | 0.003234 | [0,0.01] | 0.374031 | 56.3641 |
| overall cheese intake | 208 | -1.79807 | [-2.78,-0.82] | 0.00032 | 85.47096 | 209 | -0.57265 | [-1.14,-0.01] | 0.046801 | 85.63941 | 241 | -0.00609 | [-0.01,0] | 0.023054 | 74.23835 |
| frequency of adding salt to food | 215 | -1.34966 | [-2.44,-0.26] | 0.015464 | 89.83534 | 215 | -0.74872 | [-1.34,-0.15] | 0.01351 | 88.66858 | 259 | 0.001297 | [0,0] | 0.492481 | 56.55171 |
| temperature of hot drinks | 174 | -0.53384 | [-1.5,0.43] | 0.277212 | 82.78172 | 174 | -0.48062 | [-1.09,0.13] | 0.122703 | 85.87333 | 208 | -0.00154 | [-0.01,0] | 0.605386 | 77.0368 |
| overall alcohol intake | 270 | 0.058867 | [-0.69,0.81] | 0.877307 | 83.97599 | 273 | 0.155923 | [-0.29,0.61] | 0.496499 | 85.43962 | 320 | -0.00351 | [-0.01,0] | 0.137758 | 78.23569 |
| among current drinkers, drinks usually with meals: yes, it varies, no | 126 | -0.30041 | [-1.45,0.85] | 0.608415 | 84.75368 | 127 | -0.27763 | [-0.89,0.34] | 0.377284 | 82.64134 | 143 | -0.00237 | [-0.01,0] | 0.327315 | 53.12729 |
| milk type: skimmed, semi-skimmed, full cream (QT) | 49 | 0.749687 | [-1.4,2.9] | 0.49487 | 87.47318 | 49 | 0.499046 | [-0.64,1.64] | 0.389928 | 85.22199 | 59 | 0.002839 | [-0.01,0.01] | 0.549114 | 67.116 |

IVW: inverse variance weighted; OR: odd ratio; CI: confidence interval.

**Table S14. Associations between genetically predicted dietary habits and neuroticism and difficulty awaking using IVW.**

| **Exposure** | **Neuroticism** | | | | | **Difficulty awaking** | | | | |
| --- | --- | --- | --- | --- | --- | --- | --- | --- | --- | --- |
|  | **N snps** | **OR** | **95% CI** | **P value** | **I^2^** | **N snps** | **OR** | **95% CI** | **P value** | **I^2^** |
| alcohol drinker status: current + former vs. never | 38 | 0.998107 | [0.8,1.25] | 0.986668 | 62.29443 | 45 | 0.926365 | [0.81,1.06] | 0.26646 | 46.48804 |
| alcohol drinker status: current vs. never | 37 | 1.000157 | [0.8,1.25] | 0.998883 | 63.12464 | 45 | 0.945642 | [0.83,1.08] | 0.3987 | 46.04878 |
| among current drinkers, drinks usually with meals: yes + it varies vs. no | 99 | 0.852355 | [0.79,0.92] | 8.90E-05 | 76.09468 | 122 | 1.046217 | [1,1.1] | 0.057185 | 65.8176 |
| among current drinkers, drinks usually with meals: yes vs. no | 146 | 0.878926 | [0.84,0.92] | 4.15E-08 | 74.21993 | 176 | 1.01806 | [0.99,1.05] | 0.200816 | 63.60347 |
| never eat eggs vs. no eggs, dairy, wheat, or sugar restrictions | 9 | 1.113447 | [0.75,1.65] | 0.589451 | 54.83776 | 13 | 1.135116 | [0.89,1.44] | 0.297224 | 50.03626 |
| never eat eggs vs. no eggs restrictions | 6 | 1.29792 | [0.88,1.92] | 0.191437 | 0 | 8 | 1.244383 | [0.96,1.62] | 0.101926 | 0 |
| never eat dairy vs. no eggs, dairy, wheat, or sugar restrictions | 15 | 1.094379 | [0.78,1.54] | 0.604221 | 60.31509 | 17 | 1.132564 | [0.87,1.47] | 0.353795 | 65.26707 |
| never eat dairy vs. no dairy restrictions | 15 | 1.12716 | [0.73,1.73] | 0.584541 | 61.15472 | 16 | 1.109161 | [0.79,1.55] | 0.544634 | 64.81992 |
| never eat wheat vs. no eggs, dairy, wheat, or sugar restrictions | 17 | 0.649304 | [0.56,0.75] | 1.70E-09 | 61.00077 | 25 | 1.222427 | [1.14,1.31] | 1.66E-08 | 36.03578 |
| never eat wheat vs. no wheat restrictions | 18 | 0.574145 | [0.49,0.68] | 5.84E-11 | 59.32756 | 27 | 1.279831 | [1.18,1.38] | 5.90E-10 | 26.3453 |
| never eat sugar vs. no eggs, dairy, wheat, or sugar restrictions | 108 | 0.994952 | [0.92,1.07] | 0.895442 | 70.34608 | 137 | 1.104238 | [1.05,1.16] | 0.000123 | 69.33257 |
| never eat sugar vs. no sugar restrictions | 102 | 0.985414 | [0.91,1.06] | 0.702405 | 66.625 | 135 | 1.094428 | [1.04,1.15] | 0.000581 | 68.00892 |
| milk type: dairy-based milk vs. never | 15 | 0.981434 | [0.79,1.22] | 0.866618 | 7.772519 | 22 | 1.04015 | [0.84,1.29] | 0.71526 | 59.81537 |
| milk type: any milk vs. never | 16 | 1.038271 | [0.81,1.34] | 0.771324 | 29.577 | 22 | 0.984537 | [0.84,1.16] | 0.849102 | 23.89589 |
| milk type: full cream vs. never | 22 | 1.001761 | [0.96,1.04] | 0.927217 | 49.8752 | 27 | 1.011286 | [0.98,1.05] | 0.516695 | 69.47312 |
| milk type: full cream vs. any other | 29 | 1.005262 | [0.79,1.28] | 0.966373 | 77.29791 | 36 | 0.960756 | [0.82,1.13] | 0.633188 | 75.272 |
| milk type: semi-skimmed vs. never | 15 | 1.009432 | [0.84,1.21] | 0.919602 | 27.13759 | 22 | 0.939278 | [0.84,1.05] | 0.267884 | 19.90059 |
| milk type: semi-skimmed vs. any other | 28 | 1.046335 | [0.92,1.19] | 0.4863 | 68.19697 | 32 | 0.973144 | [0.91,1.04] | 0.420077 | 34.35874 |
| milk type: skimmed vs. never | 19 | 1.016813 | [0.95,1.09] | 0.6454 | 37.82434 | 26 | 1.05113 | [1,1.11] | 0.058948 | 47.80647 |
| milk type: skimmed vs. any other | 37 | 1.030432 | [0.9,1.18] | 0.660266 | 72.34327 | 46 | 1.056446 | [0.97,1.15] | 0.206378 | 66.97009 |
| milk type: soy milk vs. never | 23 | 1.026956 | [1,1.05] | 0.041595 | 29.12383 | 28 | 0.977772 | [0.96,1] | 0.011683 | 26.90833 |
| milk type: soy milk vs. any other | 18 | 1.080194 | [0.84,1.38] | 0.539231 | 37.95476 | 25 | 0.868683 | [0.7,1.08] | 0.213416 | 68.01059 |
| milk type: other milk vs. never | 12 | 1.026671 | [0.99,1.06] | 0.136516 | 42.92216 | 14 | 0.974777 | [0.96,0.99] | 0.012536 | 12.58907 |
| milk type: other milk vs. any other | 10 | 1.057727 | [0.65,1.73] | 0.822262 | 29.5833 | 14 | 0.873832 | [0.67,1.14] | 0.326686 | 0 |
| spread type: all spreads vs. never | 47 | 1.132727 | [0.98,1.31] | 0.09729 | 71.27861 | 53 | 0.93669 | [0.86,1.02] | 0.151234 | 58.95694 |
| spread type: butter + margarine vs. never | 51 | 1.050102 | [0.96,1.15] | 0.302672 | 70.54643 | 59 | 0.981694 | [0.92,1.05] | 0.585044 | 70.32277 |
| spread type: any oil based spread vs. never | 31 | 1.082315 | [0.99,1.18] | 0.065488 | 67.88592 | 34 | 0.94458 | [0.91,0.98] | 0.005226 | 22.67128 |
| spread type: butter and butter-like spreads vs. oil-based spreads | 37 | 0.946327 | [0.87,1.03] | 0.191283 | 53.2522 | 47 | 1.069591 | [1.01,1.14] | 0.02707 | 57.1172 |
| spread type: butter and margarine spreads vs. oil-based spreads | 46 | 0.907824 | [0.83,0.99] | 0.036957 | 74.28566 | 58 | 1.047057 | [0.99,1.1] | 0.088805 | 63.65251 |
| spread type: butter vs. never | 50 | 1.041649 | [0.98,1.11] | 0.183018 | 56.62036 | 61 | 1.00501 | [0.97,1.05] | 0.802908 | 49.83484 |
| spread type: butter vs. any other | 88 | 0.928465 | [0.86,1] | 0.045806 | 70.30398 | 105 | 0.994486 | [0.95,1.04] | 0.820257 | 66.85148 |
| spread type: tub margarine vs. never | 25 | 1.058401 | [1,1.12] | 0.059142 | 69.48902 | 30 | 0.984576 | [0.96,1.01] | 0.241119 | 23.18753 |
| spread type: tub margarine vs. any other | 25 | 1.286649 | [1.04,1.59] | 0.018029 | 58.34316 | 29 | 0.972194 | [0.88,1.07] | 0.571482 | 2.605783 |
| spread type: flora + benecol vs. never | 26 | 1.005623 | [0.95,1.06] | 0.846916 | 66.63811 | 31 | 1.001293 | [0.97,1.04] | 0.942774 | 56.26243 |
| spread type: flora + benecol vs. any other | 35 | 0.946601 | [0.85,1.06] | 0.329573 | 40.93133 | 43 | 1.045887 | [0.97,1.13] | 0.254376 | 38.81786 |
| spread type: olive oil spread vs. never | 21 | 1.081881 | [1.01,1.16] | 0.020833 | 63.93375 | 26 | 1.022194 | [0.99,1.06] | 0.174974 | 22.62924 |
| spread type: olive oil spread vs. any other | 16 | 0.917494 | [0.76,1.1] | 0.359568 | 49.33182 | 17 | 1.002768 | [0.91,1.11] | 0.95602 | 0.840287 |
| spread type: other oil-based spread vs. never | 39 | 1.040064 | [0.98,1.11] | 0.210724 | 70.50016 | 40 | 0.975379 | [0.95,1.01] | 0.111349 | 30.92669 |
| spread type: other oil-based spread vs. any other | 22 | 0.959363 | [0.81,1.14] | 0.63596 | 66.50537 | 26 | 1.07398 | [0.98,1.18] | 0.130972 | 40.8104 |
| spread type: low fat spread vs. never | 13 | 1.013034 | [0.95,1.08] | 0.676059 | 46.71196 | 14 | 0.986031 | [0.94,1.03] | 0.56892 | 53.2075 |
| spread type: low fat spread vs. any other | 13 | 1.402412 | [1.07,1.84] | 0.01463 | 49.34081 | 18 | 1.286745 | [1.03,1.61] | 0.026262 | 65.7686 |
| bread type: white vs. any other | 153 | 1.10553 | [1.04,1.17] | 0.001007 | 72.78498 | 177 | 0.96791 | [0.93,1.01] | 0.100141 | 66.61636 |
| bread type: brown vs. any other | 14 | 1.216449 | [0.76,1.95] | 0.416113 | 91.23761 | 22 | 1.015235 | [0.85,1.21] | 0.866184 | 76.0048 |
| bread type: wholemeal/wholegrain vs. any other | 127 | 0.887986 | [0.83,0.95] | 0.000261 | 75.98885 | 159 | 0.995429 | [0.96,1.03] | 0.807677 | 65.33736 |
| bread type: white vs. wholemeal/wholegrain + brown | 165 | 1.098183 | [1.04,1.16] | 0.000684 | 70.86804 | 193 | 0.977719 | [0.94,1.01] | 0.213 | 65.21463 |
| bread type: wholemeal/wholegrain vs. white + brown | 150 | 0.902408 | [0.85,0.95] | 0.000253 | 73.15731 | 182 | 0.995966 | [0.96,1.03] | 0.816981 | 65.84927 |
| cereal type: biscuit cereal vs. any other | 27 | 1.246403 | [1.06,1.46] | 0.007123 | 73.5969 | 30 | 0.895618 | [0.83,0.97] | 0.003793 | 33.92039 |
| cereal type: bran cereal vs. any other | 18 | 1.084628 | [0.9,1.31] | 0.397764 | 72.94623 | 20 | 1.007037 | [0.89,1.14] | 0.909963 | 62.29262 |
| cereal type: oat cereal vs. any other | 21 | 1.134268 | [0.98,1.31] | 0.086333 | 68.08165 | 30 | 1.084255 | [1.01,1.17] | 0.031245 | 47.82389 |
| cereal type: muesli vs. any other | 88 | 0.851085 | [0.78,0.93] | 0.000237 | 76.57175 | 104 | 0.980166 | [0.94,1.02] | 0.33703 | 47.68451 |
| cereal type: cornflakes/frosties vs. any other | 68 | 1.000516 | [0.91,1.1] | 0.991809 | 77.37809 | 84 | 1.051645 | [1,1.11] | 0.047537 | 59.59887 |
| coffee type: decaffeinated vs. any other | 142 | 0.886526 | [0.83,0.94] | 0.000131 | 76.46689 | 168 | 0.982244 | [0.95,1.02] | 0.300276 | 59.79859 |
| coffee type: ground+instant vs. other+decaff | 32 | 0.926134 | [0.81,1.06] | 0.250829 | 73.62615 | 38 | 0.988582 | [0.93,1.05] | 0.726965 | 44.67901 |
| coffee type: ground vs. any other | 31 | 1.038081 | [0.91,1.18] | 0.576352 | 71.15792 | 34 | 1.027289 | [0.96,1.1] | 0.416538 | 35.89572 |
| tablespoons of cooked vegetables per day | 117 | 0.951334 | [0.88,1.03] | 0.195707 | 74.48256 | 139 | 1.051176 | [1.01,1.1] | 0.028149 | 63.24868 |
| tablespoons of raw vegetables per day | 138 | 1.003424 | [0.93,1.08] | 0.93103 | 80.13177 | 163 | 1.005563 | [0.96,1.05] | 0.813964 | 72.01841 |
| pieces of fresh fruit per day | 238 | 0.907577 | [0.87,0.95] | 5.38E-05 | 72.71385 | 284 | 1.121526 | [1.09,1.15] | 9.86E-16 | 60.5812 |
| pieces of dried fruit per day | 116 | 0.927123 | [0.86,1] | 0.042334 | 74.03583 | 134 | 1.02387 | [0.98,1.07] | 0.259669 | 57.34865 |
| slices of bread per week | 123 | 1.058326 | [0.98,1.14] | 0.121969 | 74.65749 | 149 | 0.961608 | [0.92,1] | 0.06651 | 62.45067 |
| bowls of cereal per week | 167 | 0.954468 | [0.9,1.01] | 0.125349 | 73.18395 | 188 | 1.051102 | [1.01,1.09] | 0.00996 | 65.37947 |
| cups of tea per day | 165 | 1.039234 | [0.98,1.1] | 0.188522 | 74.25037 | 201 | 0.928081 | [0.9,0.96]* | 9.72E-06 | 61.08923 |
| cups of coffee per day | 120 | 0.939719 | [0.89,0.99] | 0.028775 | 71.46376 | 155 | 1.012537 | [0.98,1.05] | 0.507042 | 68.06268 |
| glasses of water per day | 243 | 0.960406 | [0.92,1.01] | 0.089919 | 71.36347 | 294 | 1.053025 | [1.02,1.09] | 0.001191 | 67.83398 |
| champagne/white wine glasses per month | 69 | 0.925464 | [0.83,1.03] | 0.146546 | 81.30181 | 82 | 0.911845 | [0.87,0.96] | 0.000397 | 59.09006 |
| red wine glasses per month | 127 | 0.885938 | [0.83,0.94] | 8.93E-05 | 71.22959 | 153 | 0.954823 | [0.92,0.99] | 0.024248 | 66.13564 |
| beer/cider glasses per month | 104 | 1.002516 | [0.94,1.07] | 0.943428 | 74.55772 | 127 | 0.971951 | [0.93,1.01] | 0.196271 | 66.70229 |
| spirits measures per month | 42 | 0.996984 | [0.9,1.1] | 0.953359 | 60.47826 | 47 | 0.952949 | [0.89,1.02] | 0.175718 | 55.86678 |
| fortwine glasses per month | 10 | 0.963143 | [0.79,1.18] | 0.71873 | 58.31207 | 17 | 1.004398 | [0.9,1.12] | 0.93471 | 45.06645 |
| other alcohol glasses per month | 9 | 1.009326 | [0.87,1.17] | 0.899088 | 54.54097 | 12 | 1.006449 | [0.94,1.07] | 0.846525 | 0 |
| total drinks of alcohol per month | 261 | 0.94096 | [0.9,0.99] | 0.010875 | 77.51674 | 317 | 0.960859 | [0.94,0.99] | 0.002116 | 60.19124 |
| overall oily fish intake | 172 | 0.982749 | [0.93,1.04] | 0.561715 | 75.79991 | 217 | 0.999069 | [0.96,1.04] | 0.959354 | 68.4415 |
| overall non-oily fish intake | 73 | 1.035302 | [0.95,1.13] | 0.427344 | 65.82482 | 83 | 1.036326 | [0.98,1.1] | 0.249471 | 65.76825 |
| overall processed meat intake | 101 | 1.024522 | [0.94,1.11] | 0.55759 | 73.27493 | 127 | 0.913014 | [0.87,0.96] | 0.000149 | 62.45071 |
| overall poultry intake | 53 | 1.0034 | [0.9,1.12] | 0.951968 | 71.0789 | 68 | 1.022755 | [0.95,1.1] | 0.543293 | 68.5178 |
| overall beef intake | 96 | 0.942743 | [0.87,1.02] | 0.142479 | 70.54442 | 115 | 0.9744 | [0.92,1.03] | 0.339847 | 67.50978 |
| overall lamb/mutton intake | 116 | 0.917718 | [0.85,0.99] | 0.029566 | 75.58481 | 134 | 0.987161 | [0.94,1.03] | 0.569287 | 62.31178 |
| overall pork intake | 73 | 0.899724 | [0.83,0.98] | 0.011055 | 63.95519 | 83 | 1.055333 | [1,1.11] | 0.04519 | 53.72301 |
| overall cheese intake | 208 | 0.928321 | [0.88,0.98] | 0.003333 | 67.6505 | 241 | 0.960739 | [0.93,0.99] | 0.013616 | 59.32396 |
| frequency of adding salt to food | 209 | 1.081687 | [1.03,1.13] | 0.000563 | 64.50558 | 259 | 0.99236 | [0.96,1.02] | 0.594806 | 56.78503 |
| temperature of hot drinks | 169 | 1.032293 | [0.97,1.1] | 0.297492 | 73.81855 | 208 | 1.092371 | [1.05,1.13] | 1.45E-06 | 64.6416 |
| overall alcohol intake | 269 | 0.944769 | [0.9,0.99] | 0.009719 | 72.36953 | 320 | 0.965579 | [0.94,0.99] | 0.009609 | 61.43245 |
| among current drinkers, drinks usually with meals: yes, it varies, no | 126 | 0.872399 | [0.82,0.93] | 3.38E-05 | 72.63833 | 143 | 1.047689 | [1,1.09] | 0.03631 | 68.02857 |
| milk type: skimmed, semi-skimmed, full cream (QT) | 53 | 0.941698 | [0.83,1.07] | 0.339834 | 79.78963 | 59 | 0.908767 | [0.84,0.98] | 0.010259 | 69.19369 |

IVW: inverse variance weighted; OR: odd ratio; CI: confidence interval.

**Table S15. Associations between genetically predicted dietary habits and insomnia, MDD and anxiety using IVW.**

| **Exposure** | **Insomnia** | | | | | **MDD** | | | | | **Anxiety** | | | | |
| --- | --- | --- | --- | --- | --- | --- | --- | --- | --- | --- | --- | --- | --- | --- | --- |
|  | **N snps** | **OR** | **95% CI** | **P value** | **I^2^** | **N snps** | **OR** | **95% CI** | **P value** | **I^2^** | **N snps** | **OR** | **95% CI** | **P value** | **I^2^** |
| alcohol drinker status: current + former vs. never | 38 | 0.945189 | [0.62,1.43] | 0.789535 | 45.38122 | 42 | 1.104429 | [0.77,1.59] | 0.590653 | 49.25291 | 34 | 0.33288186 | [0.06,1.9] | 0.216319924 | 18.23204111 |
| alcohol drinker status: current vs. never | 37 | 0.960212 | [0.65,1.42] | 0.837879 | 41.03627 | 40 | 1.083916 | [0.76,1.54] | 0.655581 | 48.4753 | 33 | 0.431293477 | [0.08,2.42] | 0.339031456 | 21.17781406 |
| among current drinkers, drinks usually with meals: yes + it varies vs. no | 99 | 0.65951 | [0.59,0.74] | 3.69E-12 | 45.14973 | 107 | 0.717905 | [0.64,0.81] | 4.33E-08 | 64.33498 | 101 | 0.322811433 | [0.21,0.5]** | 2.1867E-07 | 3.652485418 |
| among current drinkers, drinks usually with meals: yes vs. no | 146 | 0.738111 | [0.69,0.79] | 1.28E-21 | 30.14171 | 156 | 0.802043 | [0.75,0.86] | 9.68E-11 | 59.0971 | 149 | 0.592388505 | [0.45,0.78]** | 0.000261249 | 19.97804142 |
| never eat eggs vs. no eggs, dairy, wheat, or sugar restrictions | 9 | 1.962954 | [0.83,4.67] | 0.127234 | 54.1116 | 11 | 1.73099 | [1.09,2.75] | 0.019965 | 7.140067 | 10 | 1.327460484 | [0.05,33.36] | 0.863281851 | 26.72824256 |
| never eat eggs vs. no eggs restrictions | 6 | 2.006615 | [0.48,8.33] | 0.337732 | 61.80607 | 7 | 1.626274 | [0.82,3.21] | 0.160744 | 0 | 7 | 0.141649118 | [0,8.32] | 0.34695338 | 0 |
| never eat dairy vs. no eggs, dairy, wheat, or sugar restrictions | 15 | 0.969873 | [0.55,1.72] | 0.916745 | 30.62267 | 17 | 0.894736 | [0.44,1.81] | 0.757057 | 71.44394 | 13 | 0.494865573 | [0.05,4.97] | 0.550181522 | 0 |
| never eat dairy vs. no dairy restrictions | 15 | 0.93721 | [0.46,1.91] | 0.858121 | 30.01858 | 17 | 0.870695 | [0.36,2.1] | 0.757985 | 71.73021 | 13 | 0.383743161 | [0.02,6.7] | 0.511505018 | 0 |
| never eat wheat vs. no eggs, dairy, wheat, or sugar restrictions | 17 | 1.022781 | [0.82,1.27] | 0.839048 | 22.08122 | 18 | 0.359952 | [0.19,0.68] | 0.001602 | 85.99556 | 15 | 0.260771413 | [0.04,1.63] | 0.150978641 | 50.0535605 |
| never eat wheat vs. no wheat restrictions | 18 | 1.036107 | [0.82,1.31] | 0.764328 | 0 | 19 | 0.299269 | [0.14,0.64] | 0.002033 | 86.06202 | 16 | 0.144996699 | [0.02,1.17] | 0.06943534 | 32.2936382 |
| never eat sugar vs. no eggs, dairy, wheat, or sugar restrictions | 108 | 1.182463 | [1.04,1.35] | 0.013211 | 51.82896 | 123 | 1.031498 | [0.93,1.15] | 0.565159 | 52.54903 | 123 | 1.770603629 | [1.09,2.88]** | 0.021641634 | 25.99273975 |
| never eat sugar vs. no sugar restrictions | 102 | 1.248063 | [1.09,1.42] | 0.001032 | 45.40839 | 121 | 1.020089 | [0.92,1.14] | 0.719528 | 51.88151 | 121 | 1.658491239 | [1.02,2.71]** | 0.043011679 | 22.51185515 |
| milk type: dairy-based milk vs. never | 15 | 0.813622 | [0.43,1.53] | 0.52113 | 43.49132 | 16 | 0.665644 | [0.32,1.38] | 0.273892 | 70.84704 | 17 | 0.486270633 | [0.03,7.91] | 0.612355822 | 36.61791158 |
| milk type: any milk vs. never | 16 | 0.774983 | [0.48,1.25] | 0.298486 | 0 | 16 | 1.356225 | [0.78,2.35] | 0.278176 | 42.75223 | 15 | 1.266527214 | [0.09,17.83] | 0.860979207 | 14.79071291 |
| milk type: full cream vs. never | 22 | 0.948645 | [0.86,1.05] | 0.315083 | 65.56068 | 25 | 0.964501 | [0.9,1.04] | 0.332563 | 57.24899 | 22 | 0.902347285 | [0.63,1.29] | 0.571383074 | 30.23660772 |
| milk type: full cream vs. any other | 29 | 1.060342 | [0.71,1.58] | 0.773989 | 58.26042 | 33 | 0.878855 | [0.54,1.43] | 0.604276 | 81.91717 | 33 | 0.332919229 | [0.09,1.23] | 0.098471636 | 17.37251294 |
| milk type: semi-skimmed vs. never | 15 | 0.756843 | [0.46,1.25] | 0.276624 | 51.13457 | 16 | 0.645972 | [0.39,1.07] | 0.088881 | 66.81631 | 17 | 0.486551322 | [0.07,3.63] | 0.48230312 | 33.18332762 |
| milk type: semi-skimmed vs. any other | 28 | 0.813216 | [0.66,1] | 0.054622 | 38.96127 | 30 | 0.932735 | [0.73,1.19] | 0.574372 | 69.63785 | 27 | 0.291486938 | [0.12,0.68]** | 0.004660725 | 10.94064107 |
| milk type: skimmed vs. never | 19 | 0.90938 | [0.78,1.06] | 0.221843 | 31.72948 | 23 | 1.122409 | [1.01,1.25] | 0.03734 | 14.71061 | 22 | 0.993262567 | [0.54,1.81] | 0.982428613 | 8.333112893 |
| milk type: skimmed vs. any other | 37 | 1.102469 | [0.9,1.34] | 0.336158 | 38.58214 | 41 | 1.181449 | [0.93,1.51] | 0.179915 | 73.20778 | 38 | 3.208701477 | [1.38,7.45]** | 0.006675954 | 21.58831811 |
| milk type: soy milk vs. never | 23 | 0.980179 | [0.93,1.03] | 0.416095 | 0 | 24 | 1.023246 | [0.98,1.07] | 0.34989 | 31.36008 | 20 | 1.082634867 | [0.84,1.4] | 0.539647215 | 3.132501966 |
| milk type: soy milk vs. any other | 18 | 0.783706 | [0.46,1.34] | 0.373796 | 36.34612 | 24 | 1.493041 | [0.82,2.71] | 0.188493 | 72.42091 | 21 | 1.174749122 | [0.11,12.1] | 0.892340596 | 30.7042596 |
| milk type: other milk vs. never | 12 | 1.027812 | [0.95,1.11] | 0.473974 | 38.99705 | 14 | 1.022355 | [0.97,1.08] | 0.38939 | 18.17158 | 11 | 1.421234756 | [1.06,1.91]** | 0.020340035 | 0 |
| milk type: other milk vs. any other | 10 | 0.846452 | [0.26,2.71] | 0.778856 | 37.20823 | 14 | 0.991171 | [0.5,1.96] | 0.979683 | 4.366636 | 8 | 32.98988596 | [0.17,6237.26] | 0.191147261 | 0 |
| spread type: all spreads vs. never | 47 | 1.051574 | [0.85,1.31] | 0.649667 | 34.66036 | 51 | 1.042901 | [0.82,1.32] | 0.726631 | 63.45038 | 45 | 1.504860138 | [0.57,3.94] | 0.405423296 | 17.22486562 |
| spread type: butter + margarine vs. never | 51 | 0.985689 | [0.84,1.16] | 0.862178 | 52.60716 | 55 | 0.98119 | [0.84,1.15] | 0.815334 | 67.30457 | 51 | 1.025784933 | [0.57,1.86] | 0.93331685 | 15.86219859 |
| spread type: any oil based spread vs. never | 31 | 1.051184 | [0.93,1.19] | 0.43999 | 30.32888 | 33 | 0.949534 | [0.78,1.16] | 0.611911 | 80.92905 | 30 | 1.187378781 | [0.7,2.01] | 0.522996817 | 0 |
| spread type: butter and butter-like spreads vs. oil-based spreads | 37 | 0.989774 | [0.87,1.13] | 0.875009 | 2.598538 | 41 | 0.94686 | [0.82,1.1] | 0.472306 | 53.68775 | 37 | 0.853933452 | [0.44,1.66] | 0.64227863 | 15.11715468 |
| spread type: butter and margarine spreads vs. oil-based spreads | 46 | 1.009348 | [0.9,1.13] | 0.875379 | 22.28298 | 52 | 0.965683 | [0.84,1.11] | 0.633916 | 67.28535 | 49 | 0.915874916 | [0.55,1.52] | 0.732419375 | 7.83195091 |
| spread type: butter vs. never | 50 | 1.037842 | [0.94,1.15] | 0.460477 | 20.46376 | 57 | 0.980877 | [0.88,1.09] | 0.719741 | 55.68674 | 52 | 0.981856655 | [0.61,1.57] | 0.939016115 | 16.53107113 |
| spread type: butter vs. any other | 87 | 0.94829 | [0.84,1.06] | 0.369327 | 41.74429 | 98 | 0.949933 | [0.84,1.08] | 0.42592 | 68.86592 | 94 | 0.757297378 | [0.49,1.17] | 0.208292952 | 9.00745919 |
| spread type: tub margarine vs. never | 25 | 1.051949 | [0.97,1.15] | 0.248928 | 30.1768 | 28 | 0.991 | [0.87,1.12] | 0.887114 | 78.41799 | 25 | 1.065503273 | [0.7,1.63] | 0.768997999 | 29.4821796 |
| spread type: tub margarine vs. any other | 25 | 1.187197 | [0.75,1.87] | 0.45922 | 56.33313 | 28 | 1.056598 | [0.75,1.48] | 0.749621 | 49.57552 | 26 | 1.428040218 | [0.22,9.18] | 0.707436043 | 39.6651248 |
| spread type: flora + benecol vs. never | 26 | 0.970417 | [0.89,1.06] | 0.52091 | 36.23187 | 27 | 0.967885 | [0.88,1.06] | 0.477273 | 54.20041 | 26 | 1.272411374 | [0.89,1.82] | 0.189005088 | 0 |
| spread type: flora + benecol vs. any other | 35 | 1.034633 | [0.84,1.27] | 0.748556 | 17.56591 | 40 | 1.066965 | [0.91,1.25] | 0.433759 | 11.56204 | 38 | 1.255878422 | [0.51,3.07] | 0.617067961 | 1.92884706 |
| spread type: olive oil spread vs. never | 21 | 0.937605 | [0.86,1.03] | 0.159123 | 0 | 26 | 1.006707 | [0.89,1.13] | 0.912655 | 67.55288 | 20 | 1.1174275 | [0.68,1.83] | 0.657542884 | 21.63001456 |
| spread type: olive oil spread vs. any other | 16 | 0.716313 | [0.45,1.14] | 0.155521 | 57.68999 | 16 | 0.765415 | [0.55,1.06] | 0.104134 | 41.33861 | 14 | 2.119725795 | [0.22,20.68] | 0.517980931 | 55.97724164 |
| spread type: other oil-based spread vs. never | 39 | 1.006479 | [0.91,1.11] | 0.897814 | 42.4703 | 40 | 0.924528 | [0.81,1.05] | 0.241775 | 77.47105 | 35 | 1.001648806 | [0.68,1.48] | 0.993443707 | 4.41035779 |
| spread type: other oil-based spread vs. any other | 22 | 1.060449 | [0.78,1.45] | 0.712177 | 48.67675 | 23 | 1.015789 | [0.76,1.35] | 0.914575 | 58.28036 | 22 | 0.48421932 | [0.16,1.43] | 0.188308491 | 0 |
| spread type: low fat spread vs. never | 13 | 1.013979 | [0.91,1.13] | 0.796441 | 10.69817 | 12 | 1.104566 | [0.98,1.25] | 0.106085 | 48.66415 | 11 | 1.09441531 | [0.65,1.83] | 0.731827485 | 0 |
| spread type: low fat spread vs. any other | 13 | 1.235828 | [0.66,2.3] | 0.503098 | 49.73737 | 15 | 1.749806 | [1.09,2.81] | 0.021083 | 46.53669 | 12 | 2.251223964 | [0.23,21.91] | 0.484616082 | 0 |
| bread type: white vs. any other | 153 | 1.317867 | [1.2,1.45] | 9.10E-09 | 45.47716 | 165 | 1.095199 | [0.99,1.21] | 0.0635 | 65.21394 | 162 | 1.328498744 | [0.89,1.97] | 0.159069101 | 31.6976051 |
| bread type: brown vs. any other | 14 | 0.801356 | [0.56,1.15] | 0.228525 | 25.78521 | 18 | 0.875173 | [0.64,1.2] | 0.412345 | 46.53838 | 15 | 1.045611215 | [0.16,6.78] | 0.962691009 | 46.8929303 |
| bread type: wholemeal/wholegrain vs. any other | 127 | 0.757976 | [0.69,0.83] | 6.67E-09 | 44.48155 | 141 | 0.945729 | [0.85,1.05] | 0.305296 | 72.26402 | 135 | 0.918688319 | [0.63,1.34] | 0.662721015 | 27.04639403 |
| bread type: white vs. wholemeal/wholegrain + brown | 165 | 1.31633 | [1.21,1.44] | 5.86E-10 | 44.02092 | 178 | 1.091191 | [1,1.19] | 0.05016 | 63.2363 | 177 | 1.546190169 | [1.09,2.2]** | 0.014850831 | 24.87335334 |
| bread type: wholemeal/wholegrain vs. white + brown | 150 | 0.745175 | [0.69,0.81]* | 3.80E-12 | 41.29937 | 166 | 0.956149 | [0.87,1.05] | 0.326903 | 67.65262 | 159 | 0.831810109 | [0.59,1.17] | 0.29513959 | 25.93404597 |
| cereal type: biscuit cereal vs. any other | 27 | 1.275617 | [1,1.63] | 0.049755 | 41.95106 | 29 | 1.406321 | [1.16,1.71] | 0.000654 | 40.65365 | 25 | 1.243732207 | [0.4,3.9] | 0.708481662 | 34.1636851 |
| cereal type: bran cereal vs. any other | 18 | 0.966127 | [0.73,1.28] | 0.811623 | 35.38236 | 19 | 1.096675 | [0.82,1.47] | 0.533254 | 58.23683 | 18 | 0.796495689 | [0.17,3.7] | 0.771718794 | 43.25650932 |
| cereal type: oat cereal vs. any other | 21 | 1.112903 | [0.82,1.51] | 0.49362 | 64.72692 | 25 | 1.200467 | [1,1.45] | 0.054072 | 41.34068 | 22 | 1.998061195 | [0.84,4.75] | 0.117086076 | 0 |
| cereal type: muesli vs. any other | 88 | 0.690501 | [0.61,0.78] | 1.38E-09 | 39.38777 | 88 | 0.742595 | [0.65,0.85] | 3.30E-05 | 68.69764 | 86 | 0.558527901 | [0.35,0.9]** | 0.01700045 | 11.72636001 |
| cereal type: cornflakes/frosties vs. any other | 68 | 1.204465 | [1.04,1.4] | 0.013754 | 50.10012 | 73 | 0.898237 | [0.75,1.08] | 0.255601 | 78.50384 | 66 | 0.449611824 | [0.26,0.78]** | 0.004662483 | 13.83488349 |
| coffee type: decaffeinated vs. any other | 142 | 0.794288 | [0.73,0.86] | 1.54E-08 | 30.0823 | 156 | 0.958664 | [0.86,1.06] | 0.430587 | 73.24185 | 151 | 0.760512286 | [0.55,1.06] | 0.105842503 | 8.480115231 |
| coffee type: ground+instant vs. other+decaff | 32 | 0.66645 | [0.51,0.87] | 0.002893 | 68.42353 | 34 | 0.855211 | [0.7,1.05] | 0.128498 | 61.67447 | 31 | 0.656673971 | [0.3,1.45] | 0.297835117 | 4.352479491 |
| coffee type: ground vs. any other | 31 | 1.259398 | [1,1.59] | 0.054964 | 55.12915 | 32 | 1.179273 | [0.97,1.43] | 0.095165 | 53.43761 | 27 | 0.671019743 | [0.25,1.83] | 0.436604132 | 30.2698375 |
| tablespoons of cooked vegetables per day | 117 | 1.000836 | [0.89,1.13] | 0.989334 | 50.05183 | 128 | 1.138746 | [1.02,1.28] | 0.025658 | 63.09761 | 125 | 1.323676719 | [0.83,2.12] | 0.242959335 | 27.77836832 |
| tablespoons of raw vegetables per day | 138 | 1.045658 | [0.93,1.17] | 0.446335 | 55.08892 | 149 | 1.095765 | [1,1.2] | 0.058948 | 56.04983 | 140 | 1.213528152 | [0.81,1.82] | 0.349021712 | 16.86637833 |
| pieces of fresh fruit per day | 238 | 0.838828 | [0.78,0.9] | 4.53E-06 | 46.46028 | 256 | 0.90455 | [0.84,0.97] | 0.007932 | 63.29075 | 257 | 0.872244771 | [0.65,1.16] | 0.352942977 | 19.49528217 |
| pieces of dried fruit per day | 116 | 0.734797 | [0.65,0.83] | 4.38E-07 | 51.99345 | 123 | 0.900025 | [0.79,1.03] | 0.118031 | 73.24384 | 121 | 0.774707881 | [0.46,1.3] | 0.336834161 | 40.51695697 |
| slices of bread per week | 123 | 0.933609 | [0.83,1.05] | 0.244593 | 51.25267 | 129 | 0.972299 | [0.87,1.08] | 0.6143 | 62.17711 | 129 | 1.126849281 | [0.74,1.71] | 0.575954838 | 15.76485233 |
| bowls of cereal per week | 167 | 0.812531 | [0.75,0.88] | 4.42E-07 | 27.27979 | 174 | 0.853291 | [0.77,0.94] | 0.001727 | 67.20659 | 171 | 0.55577453 | [0.4,0.77]** | 0.000504069 | 2.249937526 |
| cups of tea per day | 165 | 0.999046 | [0.92,1.09] | 0.98226 | 40.52848 | 182 | 1.073699 | [0.98,1.18] | 0.123519 | 66.64741 | 177 | 0.957050567 | [0.67,1.37] | 0.811093182 | 29.53921229 |
| cups of coffee per day | 120 | 0.920656 | [0.84,1.01] | 0.085557 | 50.37278 | 140 | 0.945496 | [0.86,1.04] | 0.240227 | 68.31912 | 136 | 0.850544694 | [0.63,1.16] | 0.302372942 | 3.903928907 |
| glasses of water per day | 243 | 0.992599 | [0.92,1.07] | 0.850101 | 47.96046 | 274 | 1.004669 | [0.94,1.08] | 0.898816 | 61.45889 | 268 | 1.333253409 | [1,1.78]* | 0.049417014 | 18.99057334 |
| champagne/white wine glasses per month | 69 | 0.922181 | [0.78,1.09] | 0.338207 | 62.87848 | 73 | 0.891882 | [0.77,1.03] | 0.131495 | 65.64935 | 75 | 0.595267247 | [0.33,1.07] | 0.083137341 | 30.45444653 |
| red wine glasses per month | 127 | 0.795149 | [0.72,0.88] | 7.48E-06 | 46.95947 | 137 | 0.821103 | [0.73,0.92] | 0.000507 | 68.53196 | 138 | 0.589181518 | [0.42,0.83]** | 0.002791687 | 0 |
| beer/cider glasses per month | 104 | 1.129613 | [1.02,1.25] | 0.022727 | 43.99134 | 117 | 1.005095 | [0.89,1.13] | 0.933943 | 70.71887 | 112 | 0.958758388 | [0.65,1.42] | 0.832294008 | 9.943590975 |
| spirits measures per month | 42 | 1.018949 | [0.83,1.26] | 0.861088 | 54.39233 | 46 | 0.944908 | [0.79,1.13] | 0.534508 | 58.46378 | 40 | 0.935232821 | [0.43,2.01] | 0.864054306 | 14.35341037 |
| fortwine glasses per month | 10 | 0.880024 | [0.65,1.19] | 0.407583 | 4.55575 | 15 | 0.768079 | [0.58,1.01] | 0.058797 | 45.90009 | 14 | 1.08973798 | [0.34,3.51] | 0.885528684 | 0 |
| other alcohol glasses per month | 9 | 0.941954 | [0.74,1.19] | 0.618919 | 16.59321 | 11 | 0.999263 | [0.82,1.21] | 0.994027 | 25.05744 | 7 | 0.440583848 | [0.07,2.91] | 0.394357191 | 57.93748147 |
| total drinks of alcohol per month | 261 | 0.92202 | [0.86,0.99] | 0.01894 | 45.92187 | 284 | 0.907522 | [0.84,0.98] | 0.010877 | 66.26383 | 278 | 0.914225016 | [0.7,1.2] | 0.516611007 | 21.40684135 |
| overall oily fish intake | 172 | 0.850249 | [0.78,0.93] | 0.000214 | 43.22853 | 188 | 1.051503 | [0.95,1.16] | 0.320784 | 72.03833 | 188 | 1.069936143 | [0.76,1.5] | 0.694106262 | 20.11637936 |
| overall non-oily fish intake | 73 | 0.975474 | [0.85,1.12] | 0.730516 | 37.35265 | 73 | 1.048061 | [0.91,1.21] | 0.525237 | 57.10898 | 69 | 0.915706886 | [0.48,1.73] | 0.786546144 | 26.67171495 |
| overall processed meat intake | 101 | 1.21954 | [1.08,1.38] | 0.002026 | 43.74352 | 108 | 0.941007 | [0.83,1.07] | 0.351151 | 64.26498 | 106 | 0.764073735 | [0.46,1.26] | 0.290687453 | 21.70368383 |
| overall poultry intake | 53 | 1.07714 | [0.91,1.28] | 0.393748 | 39.5177 | 61 | 1.110866 | [0.95,1.31] | 0.201768 | 57.98627 | 57 | 0.766980442 | [0.39,1.51] | 0.442059854 | 14.93895428 |
| overall beef intake | 96 | 1.048227 | [0.91,1.2] | 0.499389 | 51.39497 | 104 | 0.77092 | [0.67,0.88] | 0.00014 | 66.45782 | 97 | 0.476026663 | [0.27,0.83]** | 0.008859333 | 30.88285586 |
| overall lamb/mutton intake | 116 | 0.889038 | [0.79,1] | 0.056782 | 50.70386 | 124 | 0.913534 | [0.81,1.04] | 0.159429 | 69.68708 | 118 | 0.533039691 | [0.33,0.86]** | 0.010288086 | 28.3616336 |
| overall pork intake | 73 | 1.010516 | [0.9,1.14] | 0.865217 | 17.31662 | 77 | 0.86047 | [0.74,1.01] | 0.058628 | 66.24908 | 70 | 0.498486386 | [0.26,0.97]** | 0.041685388 | 33.80001034 |
| overall cheese intake | 208 | 0.852963 | [0.78,0.94] | 0.000721 | 52.87922 | 222 | 0.889716 | [0.82,0.96] | 0.003689 | 57.51323 | 219 | 0.547554599 | [0.4,0.75]** | 0.000169733 | 10.526478 |
| frequency of adding salt to food | 209 | 1.156345 | [1.07,1.25] | 0.000473 | 46.56455 | 232 | 1.153577 | [1.07,1.24] | 0.000142 | 57.3036 | 229 | 1.186314176 | [0.88,1.6] | 0.260608416 | 13.10702436 |
| temperature of hot drinks | 169 | 1.172899 | [1.07,1.29] | 0.000946 | 48.16889 | 186 | 0.890551 | [0.81,0.98] | 0.013156 | 63.65433 | 183 | 1.076859078 | [0.77,1.51] | 0.666551471 | 11.04176239 |
| overall alcohol intake | 269 | 0.884649 | [0.82,0.95] | 0.000923 | 50.93748 | 295 | 0.889306 | [0.83,0.95] | 0.001108 | 62.5344 | 286 | 0.691730317 | [0.53,0.9]** | 0.005281962 | 11.84758599 |
| among current drinkers, drinks usually with meals: yes, it varies, no | 126 | 0.689026 | [0.62,0.76] | 4.30E-13 | 44.25184 | 131 | 0.791582 | [0.72,0.87] | 9.36E-07 | 55.17299 | 128 | 0.608156363 | [0.4,0.92]** | 0.018429127 | 23.32488507 |
| milk type: skimmed, semi-skimmed, full cream (QT) | 53 | 0.833654 | [0.7,0.99] | 0.036697 | 47.83602 | 55 | 0.797578 | [0.64,0.99] | 0.042962 | 77.80426 | 48 | 0.508122663 | [0.26,1] | 0.050823134 | 15.75291363 |

IVW: inverse variance weighted; OR: odd ratio; CI: confidence interval.

**Table S16. Associations between genetically predicted dietary habits and systolic blood pressure in sensitivity analysis.**

| **Exposure** | **Weighted median** | | **Mode-based** | | **MR-Egger** | | | | **MRPRESSO** | | |
| --- | --- | --- | --- | --- | --- | --- | --- | --- | --- | --- | --- |
|  | **Beta (95% CI)** | **P value** | **Beta (95% CI)** | **P value** | **Beta (95% CI)** | **P value** | **Intercept** | **P value** | **Beta** | **P value** | **No. of outliers** |
| alcohol drinker status: current + former vs. never | 1.36(-1.84,4.56) | 0.404682 | -0.41(-4.24,3.43) | 0.834577 | 11.8(2.85,20.74) | 0.009777 | -0.07(-0.13,-0.01) | 0.0205 | 0.03 | 0.983383 | 5 |
| alcohol drinker status: current vs. never | 0.93(-2.17,4.04) | 0.556336 | -0.52(-4.19,3.15) | 0.781717 | 13.39(3.39,23.4) | 0.008707 | -0.09(-0.16,-0.02) | 0.009315 | 0.12 | 0.926354 | 6 |
| among current drinkers, drinks usually with meals: yes + it varies vs. no | -1.31(-2.22,-0.39) | 0.005065 | -1.44(-2.41,-0.47) | 0.003578 | 3.84(-1.86,9.54) | 0.186764 | -0.06(-0.14,0.01) | 0.113644 | -0.83 | 0.05897 | 12 |
| among current drinkers, drinks usually with meals: yes vs. no | -0.44(-0.99,0.12) | 0.123797 | -0.65(-1.27,-0.02) | 0.042776 | 0.1(-2.93,3.13) | 0.946325 | 0(-0.06,0.06) | 0.984642 | -0.06 | 0.822568 | 18 |
| never eat eggs vs. no eggs, dairy, wheat, or sugar restrictions | 11.39(5.14,17.64) | 0.000357 | 6.3(0.96,11.65) | 0.020759 | 17.88(-1.1,36.87) | 0.064784 | -0.08(-0.22,0.06) | 0.24378 | 7.16 | 0.04912 | 1 |
| never eat eggs vs. no eggs restrictions | 7.06(-0.19,14.31) | 0.056482 | 3.63(-3.25,10.51) | 0.301465 | 23.23(-2.36,48.81) | 0.075256 | -0.11(-0.26,0.05) | 0.169275 | 6.44 | 0.25516 | 1 |
| never eat dairy vs. no eggs, dairy, wheat, or sugar restrictions | -2.1(-7.07,2.87) | 0.407208 | -3.09(-7.83,1.65) | 0.201619 | 4.23(-15.09,23.55) | 0.667848 | -0.03(-0.16,0.11) | 0.673338 | -4.27 | 0.01636 | 4 |
| never eat dairy vs. no dairy restrictions | -2.64(-8.76,3.48) | 0.397752 | -3.8(-9.58,1.98) | 0.197493 | 4.58(-19.35,28.52) | 0.707339 | -0.03(-0.16,0.11) | 0.713613 | -5.28 | 0.015598 | 4 |
| never eat wheat vs. no eggs, dairy, wheat, or sugar restrictions | -2.72(-4.33,-1.11) | 0.000946 | -2.69(-3.99,-1.39) | 4.86E-05 | -5.14(-8.22,-2.06) | 0.001082 | 0.08(0.02,0.14) | 0.009493 | -2.17 | 0.004547 | 1 |
| never eat wheat vs. no wheat restrictions | -3.34(-5.29,-1.4) | 0.000751 | -3.33(-4.9,-1.75) | 3.51E-05 | -4.56(-7.16,-1.97) | 0.000573 | 0.04(0,0.08) | 0.076035 | -2.63 | 0.004095 | 0 |
| never eat sugar vs. no eggs, dairy, wheat, or sugar restrictions | 0.83(-0.1,1.77) | 0.081317 | 0.04(-1,1.08) | 0.93581 | -3.28(-9.09,2.53) | 0.26798 | 0.05(-0.02,0.13) | 0.154834 | 0.54 | 0.234869 | 16 |
| never eat sugar vs. no sugar restrictions | 0.73(-0.26,1.72) | 0.149353 | -0.2(-1.27,0.87) | 0.713917 | -1.91(-7.83,4) | 0.526192 | 0.03(-0.04,0.11) | 0.394671 | 0.24 | 0.589008 | 17 |
| milk type: dairy-based milk vs. never | 1.06(-3.3,5.41) | 0.634144 | 0.94(-3.02,4.9) | 0.642752 | -2.99(-21.03,15.05) | 0.745072 | 0.03(-0.11,0.16) | 0.699188 | 1.23 | 0.536562 | 2 |
| milk type: any milk vs. never | 1.01(-3.42,5.43) | 0.6553 | 0.35(-3.71,4.4) | 0.867159 | 13.2(-1.1,27.5) | 0.070469 | -0.12(-0.23,0) | 0.041605 | 0.89 | 0.649689 | 1 |
| milk type: full cream vs. never | 0.54(-0.02,1.09) | 0.057909 | 0.47(-0.1,1.05) | 0.102878 | -0.52(-2.11,1.07) | 0.51958 | 0.04(-0.03,0.11) | 0.232278 | 0.41 | 0.087667 | 0 |
| milk type: full cream vs. any other | 1.75(-0.8,4.3) | 0.1792 | 0.47(-1.71,2.65) | 0.672688 | 3.04(-13.38,19.46) | 0.716612 | -0.01(-0.15,0.13) | 0.919292 | 1.03 | 0.401836 | 4 |
| milk type: semi-skimmed vs. never | 1.04(-2.14,4.22) | 0.522621 | 1.39(-1.56,4.33) | 0.356653 | 5.2(-8.21,18.6) | 0.447255 | -0.05(-0.2,0.09) | 0.467468 | 1.83 | 0.238402 | 1 |
| milk type: semi-skimmed vs. any other | -0.01(-1.82,1.81) | 0.991983 | 0.51(-1.28,2.3) | 0.57617 | -1.08(-8.32,6.15) | 0.769037 | 0.02(-0.09,0.12) | 0.768912 | 0.52 | 0.533963 | 3 |
| milk type: skimmed vs. never | 0.01(-1.17,1.2) | 0.982897 | 0.06(-1.06,1.18) | 0.916562 | 1(-2.76,4.75) | 0.603292 | -0.03(-0.12,0.06) | 0.485223 | 0.12 | 0.823566 | 1 |
| milk type: skimmed vs. any other | 0.24(-1.59,2.07) | 0.795642 | -0.17(-1.72,1.38) | 0.826602 | -1.72(-13.06,9.61) | 0.765593 | 0.02(-0.13,0.16) | 0.833432 | -0.08 | 0.929214 | 6 |
| milk type: soy milk vs. never | 0.25(-0.18,0.68) | 0.262396 | 0.36(-0.08,0.81) | 0.110501 | -0.05(-1.11,1.01) | 0.924665 | 0.02(-0.05,0.08) | 0.622316 | 0.2 | 0.285482 | 0 |
| milk type: soy milk vs. any other | -1.9(-5.92,2.12) | 0.353776 | -1.37(-5.32,2.58) | 0.497661 | 2.5(-10.64,15.63) | 0.709454 | -0.03(-0.12,0.07) | 0.576582 | -1.06 | 0.608388 | 1 |
| milk type: other milk vs. never | 0.53(-0.05,1.11) | 0.075706 | 0.49(-0.08,1.05) | 0.091649 | 0.72(-0.86,2.3) | 0.37332 | -0.04(-0.17,0.09) | 0.520683 | 0.5 | 0.026079 | 3 |
| milk type: other milk vs. any other | 1.97(-6.77,10.72) | 0.658453 | 1.52(-7.08,10.12) | 0.728698 | -14.4(-31.3,2.49) | 0.094695 | 0.12(0.01,0.23) | 0.026426 | 3.23 | 0.465751 | 0 |
| spread type: all spreads vs. never | 3.23(1.45,5.01) | 0.000379 | 2.25(0.34,4.16) | 0.021208 | 3.34(-3.66,10.35) | 0.34938 | -0.01(-0.08,0.06) | 0.778217 | 2.16 | 0.011269 | 4 |
| spread type: butter + margarine vs. never | 2.82(1.7,3.93) | 7.79E-07 | 2.31(1.1,3.52) | 0.000178 | 3.82(-1.83,9.48) | 0.184762 | -0.03(-0.11,0.06) | 0.548531 | 2.11 | 7.05E-05 | 7 |
| spread type: any oil based spread vs. never | 0.75(-0.33,1.82) | 0.17414 | 0.26(-0.81,1.32) | 0.632713 | 1.03(-5.24,7.3) | 0.747571 | -0.01(-0.14,0.12) | 0.916406 | 0.73 | 0.129798 | 5 |
| spread type: butter and butter-like spreads vs. oil-based spreads | -0.23(-1.52,1.06) | 0.726655 | 0.37(-0.8,1.54) | 0.535702 | -1.75(-11.64,8.13) | 0.728016 | 0.02(-0.14,0.18) | 0.807817 | 0.08 | 0.865503 | 8 |
| spread type: butter and margarine spreads vs. oil-based spreads | -0.71(-1.85,0.42) | 0.218225 | 0.14(-0.9,1.18) | 0.790478 | -4.84(-12.81,3.12) | 0.233441 | 0.06(-0.08,0.2) | 0.37492 | -0.47 | 0.411811 | 10 |
| spread type: butter vs. never | 1.54(0.68,2.41) | 0.00049 | 1.58(0.6,2.57) | 0.001674 | 1.42(-2.97,5.82) | 0.525239 | 0(-0.09,0.08) | 0.966033 | 1.52 | 0.000294 | 6 |
| spread type: butter vs. any other | -0.99(-1.96,-0.01) | 0.047141 | -0.67(-1.62,0.28) | 0.168948 | 3.21(-3.47,9.9) | 0.346416 | -0.07(-0.17,0.03) | 0.15395 | -1.46 | 0.002932 | 16 |
| spread type: tub margarine vs. never | 0.66(-0.12,1.44) | 0.09814 | 0.51(-0.19,1.2) | 0.154467 | -0.57(-3.99,2.86) | 0.746734 | 0.04(-0.09,0.16) | 0.550392 | 0.48 | 0.202411 | 4 |
| spread type: tub margarine vs. any other | 0(-3.21,3.2) | 0.998575 | -1.01(-4.15,2.13) | 0.527576 | -4.89(-14.26,4.48) | 0.306449 | 0.06(-0.03,0.15) | 0.180414 | -0.55 | 0.725188 | 2 |
| spread type: flora + benecol vs. never | 1.07(0.27,1.88) | 0.00921 | 0.54(-0.2,1.28) | 0.150476 | 2.44(-0.7,5.58) | 0.128236 | -0.04(-0.16,0.08) | 0.506012 | 0.92 | 0.013099 | 7 |
| spread type: flora + benecol vs. any other | 2.49(0.4,4.59) | 0.019682 | 2.92(0.68,5.17) | 0.010698 | 6.99(1.75,12.23) | 0.008895 | -0.06(-0.12,0.01) | 0.082283 | 2.25 | 0.020212 | 5 |
| spread type: olive oil spread vs. never | 0.78(-0.09,1.64) | 0.078284 | 0.69(-0.1,1.49) | 0.087261 | -2.51(-7.49,2.46) | 0.322283 | 0.12(-0.03,0.27) | 0.125737 | 1.03 | 0.002939 | 3 |
| spread type: olive oil spread vs. any other | 0.18(-2.86,3.21) | 0.909305 | 0.08(-2.62,2.77) | 0.956417 | -5.66(-20.23,8.9) | 0.446208 | 0.09(-0.11,0.28) | 0.383065 | -0.27 | 0.810705 | 4 |
| spread type: other oil-based spread vs. never | -0.07(-0.81,0.67) | 0.853974 | -0.25(-0.98,0.47) | 0.494249 | 0.19(-3.5,3.89) | 0.918893 | 0.01(-0.1,0.11) | 0.913564 | 0.26 | 0.395262 | 4 |
| spread type: other oil-based spread vs. any other | 1.19(-1.11,3.5) | 0.309556 | 1(-1.09,3.1) | 0.346885 | 7.78(-1.77,17.33) | 0.110508 | -0.06(-0.18,0.05) | 0.279217 | 1.78 | 0.157881 | 3 |
| spread type: low fat spread vs. never | -0.17(-1.13,0.79) | 0.731793 | 0.23(-0.62,1.09) | 0.590046 | -2.44(-5.99,1.12) | 0.179482 | 0.07(-0.06,0.21) | 0.278717 | -0.01 | 0.979867 | 1 |
| spread type: low fat spread vs. any other | -0.17(-4.01,3.68) | 0.93223 | 0.36(-3.17,3.89) | 0.841856 | -20.11(-53.85,13.63) | 0.242724 | 0.13(-0.15,0.41) | 0.365159 | 0.71 | 0.63175 | 1 |
| bread type: white vs. any other | 2.31(1.59,3.03) | 3.15E-10 | 2.08(1.22,2.94) | 2.23E-06 | 1.64(-1.87,5.16) | 0.358901 | 0.01(-0.04,0.06) | 0.774061 | 2.47 | 8.62E-12 | 14 |
| bread type: brown vs. any other | -1.21(-3.8,1.37) | 0.356822 | -1.06(-3.47,1.35) | 0.390027 | 1.45(-10.66,13.55) | 0.814704 | -0.04(-0.18,0.09) | 0.514972 | -0.56 | 0.566425 | 3 |
| bread type: wholemeal/wholegrain vs. any other | -1.48(-2.24,-0.72) | 0.000131 | -1.12(-1.97,-0.27) | 0.009496 | -1.26(-5.87,3.35) | 0.591915 | -0.01(-0.07,0.06) | 0.872682 | -1.51 | 7.08E-05 | 16 |
| bread type: white vs. wholemeal/wholegrain + brown | 1.96(1.29,2.63) | 8.13E-09 | 1.82(0.98,2.66) | 2.01E-05 | 2.51(-0.67,5.69) | 0.122328 | -0.01(-0.06,0.03) | 0.605496 | 2.11 | 9.76E-11 | 17 |
| bread type: wholemeal/wholegrain vs. white + brown | -1.68(-2.35,-1) | 1.24E-06 | -1.32(-2.12,-0.52) | 0.001174 | -3.13(-6.78,0.53) | 0.093551 | 0.03(-0.03,0.08) | 0.337257 | -1.58 | 2.35E-06 | 18 |
| cereal type: biscuit cereal vs. any other | 1.68(-0.29,3.65) | 0.093866 | 0.55(-1.17,2.27) | 0.529732 | 6.94(-0.14,14.02) | 0.054639 | -0.09(-0.2,0.02) | 0.109383 | 0.67 | 0.455542 | 3 |
| cereal type: bran cereal vs. any other | -0.48(-2.58,1.62) | 0.653524 | -0.65(-2.63,1.33) | 0.521141 | 4.64(-3.94,13.23) | 0.289163 | -0.1(-0.21,0.02) | 0.092959 | -0.82 | 0.347737 | 1 |
| cereal type: oat cereal vs. any other | 0.12(-1.63,1.87) | 0.892846 | 0.5(-1.08,2.08) | 0.534402 | -3.57(-10.02,2.87) | 0.27738 | 0.06(-0.04,0.16) | 0.256305 | 0.72 | 0.389903 | 1 |
| cereal type: muesli vs. any other | -2.94(-3.94,-1.93) | 9.48E-09 | -2.78(-3.9,-1.65) | 1.29E-06 | 3.74(-2.25,9.72) | 0.221515 | -0.08(-0.16,0) | 0.058222 | -2.16 | 1.15E-05 | 7 |
| cereal type: cornflakes/frosties vs. any other | 2.32(1.12,3.51) | 0.000142 | 1.09(-0.15,2.33) | 0.083749 | -0.18(-4.6,4.23) | 0.935295 | 0.04(-0.03,0.1) | 0.276532 | 1.86 | 0.00188 | 7 |
| coffee type: decaffeinated vs. any other | -1.7(-2.44,-0.96) | 6.69E-06 | -1.29(-2.17,-0.41) | 0.004121 | 0.36(-4.04,4.76) | 0.873862 | -0.03(-0.1,0.03) | 0.294787 | -1.79 | 7.80E-07 | 18 |
| coffee type: ground+instant vs. other+decaff | -1.26(-2.93,0.41) | 0.140142 | -1.03(-2.56,0.5) | 0.18675 | 9.91(2.29,17.53) | 0.01081 | -0.17(-0.3,-0.04) | 0.010139 | -0.49 | 0.50579 | 6 |
| coffee type: ground vs. any other | -0.25(-1.96,1.45) | 0.769609 | 0.03(-1.6,1.66) | 0.971133 | -6.71(-14.85,1.43) | 0.106343 | 0.09(-0.05,0.23) | 0.212749 | -1.02 | 0.201064 | 5 |
| tablespoons of cooked vegetables per day | -1.15(-2.1,-0.2) | 0.017131 | -0.92(-2.05,0.21) | 0.111233 | 1.39(-4.02,6.8) | 0.615381 | -0.02(-0.09,0.05) | 0.508951 | -0.81 | 0.075149 | 14 |
| tablespoons of raw vegetables per day | -0.22(-1.01,0.56) | 0.57306 | -0.56(-1.46,0.34) | 0.221124 | 0.95(-2.61,4.51) | 0.600069 | -0.01(-0.06,0.03) | 0.581316 | -0.29 | 0.442399 | 13 |
| pieces of fresh fruit per day | -0.97(-1.61,-0.34) | 0.002622 | -1.09(-1.76,-0.41) | 0.001597 | 1.01(-2.06,4.08) | 0.520324 | -0.02(-0.06,0.02) | 0.341522 | -0.66 | 0.026768 | 28 |
| pieces of dried fruit per day | -1.61(-2.52,-0.7) | 0.000517 | -1.02(-2.14,0.1) | 0.074236 | -1.46(-5.04,2.12) | 0.424095 | 0.01(-0.04,0.05) | 0.818527 | -1.14 | 0.007582 | 12 |
| slices of bread per week | -0.17(-1.01,0.67) | 0.696159 | 0.08(-0.82,0.99) | 0.854942 | -1.03(-4.94,2.88) | 0.605436 | 0.03(-0.03,0.08) | 0.35722 | 0.06 | 0.885439 | 13 |
| bowls of cereal per week | 0.24(-0.52,1.01) | 0.531798 | -0.02(-1.08,1.04) | 0.964433 | 1.4(-2.18,4.97) | 0.444141 | -0.01(-0.06,0.03) | 0.539953 | -0.06 | 0.873588 | 16 |
| cups of tea per day | 0.26(-0.44,0.96) | 0.465573 | 0.4(-0.34,1.13) | 0.290975 | -1.38(-3.86,1.11) | 0.277074 | 0.02(-0.02,0.05) | 0.276975 | 0.36 | 0.269716 | 17 |
| cups of coffee per day | -1.67(-2.54,-0.8) | 0.000178 | -0.85(-1.44,-0.26) | 0.004732 | -0.8(-3.34,1.74) | 0.5373 | -0.01(-0.05,0.03) | 0.598923 | -1.56 | 4.78E-05 | 25 |
| glasses of water per day | -0.07(-0.69,0.55) | 0.824928 | -0.42(-1.22,0.38) | 0.302778 | -0.12(-2.63,2.39) | 0.925721 | 0(-0.03,0.04) | 0.857886 | -0.38 | 0.206825 | 21 |
| champagne/white wine glasses per month | -0.11(-1.23,1.01) | 0.845232 | -0.22(-1.59,1.15) | 0.757224 | 5.03(0.25,9.81) | 0.039356 | -0.07(-0.15,0) | 0.049129 | -0.39 | 0.451969 | 11 |
| red wine glasses per month | -0.97(-1.75,-0.18) | 0.01621 | -1.44(-2.59,-0.29) | 0.014444 | 3.71(0.6,6.83) | 0.01942 | -0.06(-0.11,-0.02) | 0.005181 | -1.07 | 0.002367 | 21 |
| beer/cider glasses per month | 2.69(1.83,3.55) | 7.99E-10 | 2.53(1.72,3.35) | 1.12E-09 | 5.32(2.24,8.4) | 0.000704 | -0.05(-0.1,-0.01) | 0.025717 | 1.7 | 2.22E-05 | 17 |
| spirits measures per month | -1.32(-2.93,0.3) | 0.109288 | -0.24(-1.76,1.27) | 0.752874 | 5.74(-0.36,11.84) | 0.065079 | -0.11(-0.2,-0.02) | 0.015012 | -0.81 | 0.276258 | 8 |
| fortwine glasses per month | 1.09(-1.32,3.5) | 0.376497 | 1.03(-1.28,3.33) | 0.382508 | -1.49(-6.98,4.01) | 0.595539 | 0.05(-0.03,0.13) | 0.225276 | 1.68 | 0.139151 | 0 |
| other alcohol glasses per month | -1.78(-3.77,0.22) | 0.081329 | -1.68(-3.64,0.28) | 0.092453 | -0.49(-7.2,6.23) | 0.887348 | -0.02(-0.17,0.13) | 0.754275 | -2.28 | 0.036562 | 1 |
| total drinks of alcohol per month | 1.59(0.94,2.24) | 1.49E-06 | 1.38(0.81,1.95) | 1.75E-06 | 3.08(1.37,4.79) | 0.000414 | -0.05(-0.07,-0.02) | 0.00037 | 0.14 | 0.631234 | 33 |
| overall oily fish intake | 0.13(-0.55,0.8) | 0.714331 | -0.18(-0.97,0.61) | 0.657363 | 1.21(-2.33,4.75) | 0.503398 | -0.01(-0.06,0.04) | 0.676458 | 0.18 | 0.567167 | 17 |
| overall non-oily fish intake | 1.1(-0.06,2.25) | 0.062278 | 0.47(-0.82,1.76) | 0.473819 | -1.72(-7.15,3.71) | 0.534772 | 0.05(-0.03,0.12) | 0.197554 | 1.27 | 0.02195 | 10 |
| overall processed meat intake | 0.05(-0.96,1.06) | 0.919558 | 0.2(-0.88,1.28) | 0.71478 | -2.43(-8.3,3.44) | 0.417194 | 0.02(-0.05,0.1) | 0.543604 | 0.03 | 0.957263 | 18 |
| overall poultry intake | 1.45(0.16,2.74) | 0.028139 | 1.65(0.34,2.95) | 0.013308 | -0.75(-6.83,5.33) | 0.8084 | 0.02(-0.06,0.1) | 0.602099 | 0.74 | 0.220664 | 6 |
| overall beef intake | 0.99(0.04,1.94) | 0.041822 | 1.05(0.02,2.07) | 0.045042 | 0.9(-3.06,4.87) | 0.655843 | 0(-0.05,0.05) | 0.99904 | 1.08 | 0.014543 | 12 |
| overall lamb/mutton intake | -0.57(-1.5,0.36) | 0.232696 | -0.41(-1.41,0.59) | 0.424495 | -1.04(-6,3.93) | 0.682132 | 0(-0.07,0.06) | 0.952 | -0.58 | 0.17848 | 17 |
| overall pork intake | -0.05(-1.29,1.2) | 0.942143 | 0.53(-0.75,1.8) | 0.419018 | -0.44(-6.21,5.33) | 0.880917 | 0(-0.08,0.07) | 0.94829 | 0.25 | 0.681506 | 11 |
| overall cheese intake | -1.47(-2.16,-0.78) | 3.01E-05 | -0.8(-1.62,0.01) | 0.052558 | -0.51(-4.06,3.05) | 0.780327 | -0.02(-0.07,0.03) | 0.458096 | -1.54 | 4.77E-06 | 24 |
| frequency of adding salt to food | -0.28(-0.94,0.38) | 0.400907 | 0.21(-0.48,0.89) | 0.553975 | -0.21(-4.26,3.85) | 0.9208 | -0.02(-0.07,0.04) | 0.565638 | -0.34 | 0.265488 | 26 |
| temperature of hot drinks | -0.94(-1.71,-0.17) | 0.016439 | -0.74(-1.73,0.24) | 0.139367 | -0.08(-3.76,3.6) | 0.966472 | -0.01(-0.05,0.04) | 0.801808 | -0.85 | 0.023329 | 19 |
| overall alcohol intake | 0.37(-0.23,0.97) | 0.228649 | 0.9(0.26,1.54) | 0.006004 | 2.3(0.49,4.11) | 0.012762 | -0.03(-0.06,-0.01) | 0.007844 | -0.27 | 0.367918 | 28 |
| among current drinkers, drinks usually with meals: yes, it varies, no | -1.03(-1.82,-0.24) | 0.010317 | -1.18(-2.05,-0.31) | 0.008089 | 0.38(-4.24,4.99) | 0.872547 | -0.01(-0.07,0.06) | 0.766177 | -0.55 | 0.136359 | 18 |
| milk type: skimmed, semi-skimmed, full cream (QT) | 0.3(-1.05,1.66) | 0.661466 | 0.68(-0.53,1.89) | 0.269339 | 1.54(-6.73,9.8) | 0.715314 | -0.01(-0.12,0.1) | 0.846343 | 0.6 | 0.342665 | 5 |

MR: mendelian randomization; MRPRESSO: MR pleiotropy residual sum and outlier; OR: odd ratio; CI: confidence interval; No.: number.

**Table S17. Leave-one-out analysis of association between genetically predicted dietary habits and systolic blood pressure.**

| **Exposure** | **IVW Estimate**  **[Min, Max] ^a^** | **P value [Min, Max] ^b^** |
| --- | --- | --- |
| alcohol drinker status: current + former vs. never | [-1.01,0.6] | [0.559617927391682,0.980833471389136] |
| alcohol drinker status: current vs. never | [-2.01,-0.15] | [0.32311803213671,0.934503126926688] |
| among current drinkers, drinks usually with meals: yes + it varies vs. no | [-0.88,-0.47] | [0.154668764804083,0.466415844642913] |
| among current drinkers, drinks usually with meals: yes vs. no | [-0.07,0.17] | [0.65335531641957,0.993300854482257] |
| never eat eggs vs. no eggs, dairy, wheat, or sugar restrictions | [5.68,8.65] | [0.00467867655696353,0.0692720723518234] |
| never eat eggs vs. no eggs restrictions | [2.98,8.82] | [0.0981610119791532,0.530262374014287] |
| never eat dairy vs. no eggs, dairy, wheat, or sugar restrictions | [-1.39,1.76] | [0.584349032138937,0.932149033684324] |
| never eat dairy vs. no dairy restrictions | [-1.7,2.01] | [0.615907945782386,0.93765296299226] |
| never eat wheat vs. no eggs, dairy, wheat, or sugar restrictions | [-2.48,-1.76] | [0.000102651758937436,0.0213781052180019] |
| never eat wheat vs. no wheat restrictions | [-2.83,-1.9] | [0.000197793309730349,0.0391626040501614] |
| never eat sugar vs. no eggs, dairy, wheat, or sugar restrictions | [0.54,1.27] | [0.0720343309200825,0.475505903027616] |
| never eat sugar vs. no sugar restrictions | [0.29,1.05] | [0.161063650489933,0.710565573591852] |
| milk type: dairy-based milk vs. never | [-0.65,2.24] | [0.284211598607173,0.985159095579287] |
| milk type: any milk vs. never | [-2.4,0.41] | [0.35438720153361,0.834259879382955] |
| milk type: full cream vs. never | [0.3,0.51] | [0.0176320634209728,0.157123291202234] |
| milk type: full cream vs. any other | [1.07,2.78] | [0.149891098676044,0.541919884195211] |
| milk type: semi-skimmed vs. never | [-0.38,1.83] | [0.216536403642692,0.967417523665016] |
| milk type: semi-skimmed vs. any other | [-0.41,0.46] | [0.630683459546625,0.987376987275694] |
| milk type: skimmed vs. never | [-0.5,0.12] | [0.424150805486107,0.954906482653324] |
| milk type: skimmed vs. any other | [-1.04,0.11] | [0.425441482842108,0.992418079098224] |
| milk type: soy milk vs. never | [0.14,0.28] | [0.0958354713787179,0.441297701451078] |
| milk type: soy milk vs. any other | [-2.05,-0.31] | [0.291134965767338,0.874425556222369] |
| milk type: other milk vs. never | [0.06,0.45] | [0.133203793658168,0.84962537253615] |
| milk type: other milk vs. any other | [-2.04,2.8] | [0.455790897105144,0.943604818973925] |
| spread type: all spreads vs. never | [1.72,2.81] | [0.00321196801002287,0.0733863977307433] |
| spread type: butter + margarine vs. never | [1.77,2.43] | [0.000424688445888104,0.0123249624834823] |
| spread type: any oil based spread vs. never | [0.27,1.36] | [0.0295720008662202,0.753073764961387] |
| spread type: butter and butter-like spreads vs. oil-based spreads | [-1.8,-0.09] | [0.0465325160131181,0.95241317898536] |
| spread type: butter and margarine spreads vs. oil-based spreads | [-2.24,-0.97] | [0.00783511168646719,0.425635579479553] |
| spread type: butter vs. never | [1.05,1.59] | [0.00472854527849568,0.0570006177056525] |
| spread type: butter vs. any other | [-1.87,-1.07] | [0.020540269679568,0.196163579860838] |
| spread type: tub margarine vs. never | [0.1,0.77] | [0.100914611443282,0.851193088089793] |
| spread type: tub margarine vs. any other | [0.27,1.65] | [0.338009181479997,0.86845409496735] |
| spread type: flora + benecol vs. never | [1.06,1.68] | [0.00303296872183973,0.0604044096114295] |
| spread type: flora + benecol vs. any other | [1.45,3.44] | [0.00219417842302906,0.216232458922151] |
| spread type: olive oil spread vs. never | [0.86,1.69] | [0.000482550153036631,0.164083330230602] |
| spread type: olive oil spread vs. any other | [-2.12,1.91] | [0.423955037298875,0.990419936410142] |
| spread type: other oil-based spread vs. never | [0.13,0.79] | [0.0471750160316058,0.810306644226494] |
| spread type: other oil-based spread vs. any other | [2.03,3.23] | [0.027536801189342,0.14056366067908] |
| spread type: low fat spread vs. never | [-0.84,-0.01] | [0.305603619886122,0.979229761312335] |
| spread type: low fat spread vs. any other | [-6.83,0.71] | [0.310359067359426,0.621228858518829] |
| bread type: white vs. any other | [2.03,2.28] | [1.97529374497729e-07,7.24331667137579e-06] |
| bread type: brown vs. any other | [-3.08,-1.12] | [0.128028080179353,0.50740566136859] |
| bread type: wholemeal/wholegrain vs. any other | [-1.74,-1.4] | [0.000944986734484766,0.00508522079300612] |
| bread type: white vs. wholemeal/wholegrain + brown | [1.59,1.81] | [6.70568838879093e-06,0.000125018296006514] |
| bread type: wholemeal/wholegrain vs. white + brown | [-1.55,-1.27] | [0.000457755930218208,0.00583585870793276] |
| cereal type: biscuit cereal vs. any other | [0.88,2.01] | [0.116353093632421,0.456721299890732] |
| cereal type: bran cereal vs. any other | [-2.77,-0.82] | [0.0672595985639158,0.333340012403474] |
| cereal type: oat cereal vs. any other | [-0.39,0.72] | [0.37890927553921,0.992218635095048] |
| cereal type: muesli vs. any other | [-2.5,-1.6] | [2.64615188840725e-05,0.029862245677367] |
| cereal type: cornflakes/frosties vs. any other | [1.9,2.32] | [0.000451834554588413,0.0034067409755078] |
| coffee type: decaffeinated vs. any other | [-2.18,-1.81] | [2.52540285214962e-06,0.000698506844658898] |
| coffee type: ground+instant vs. other+decaff | [-2.03,-1.11] | [0.0347679777834747,0.207385688321914] |
| coffee type: ground vs. any other | [-2.48,0.53] | [0.143604167859302,0.582601577514692] |
| tablespoons of cooked vegetables per day | [-0.72,-0.06] | [0.270651396250311,0.929028010401153] |
| tablespoons of raw vegetables per day | [-0.15,0.11] | [0.744625218781778,0.998390111506379] |
| pieces of fresh fruit per day | [-0.55,-0.25] | [0.196991215100735,0.538504952357487] |
| pieces of dried fruit per day | [-1.27,-0.92] | [0.0116233964570562,0.0590915125999301] |
| slices of bread per week | [0.52,0.92] | [0.108698057720655,0.346450740753003] |
| bowls of cereal per week | [0.14,0.46] | [0.346795320274787,0.778831704837653] |
| cups of tea per day | [-0.22,0.2] | [0.640421967378193,0.981203058497125] |
| cups of coffee per day | [-1.8,-1.08] | [0.0072307422163747,0.0829157458796214] |
| glasses of water per day | [-0.08,0.2] | [0.596208117551697,0.96851974612231] |
| champagne/white wine glasses per month | [0.01,0.87] | [0.358458311413234,0.989402094254381] |
| red wine glasses per month | [-1.29,-0.83] | [0.016147937599183,0.172402152514687] |
| beer/cider glasses per month | [1.37,1.98] | [0.00150473454543458,0.0132868162695052] |
| spirits measures per month | [-2.52,-1.62] | [0.0230827244193412,0.108207414017648] |
| fortwine glasses per month | [0.91,2.2] | [0.0269165010923459,0.329102804287691] |
| other alcohol glasses per month | [-2.28,-0.83] | [0.00737408603837209,0.40962276159958] |
| total drinks of alcohol per month | [-0.45,-0.2] | [0.277399536799716,0.629174922514579] |
| overall oily fish intake | [0.31,0.65] | [0.155377215556951,0.487497439582154] |
| overall non-oily fish intake | [1.5,2.27] | [0.00135467898450262,0.0383783888513776] |
| overall processed meat intake | [-0.87,-0.31] | [0.251086043114292,0.672377358336927] |
| overall poultry intake | [0.43,1.11] | [0.192182840677239,0.612727571204268] |
| overall beef intake | [0.72,1.09] | [0.0570886420058159,0.204815223068778] |
| overall lamb/mutton intake | [-1.35,-1.01] | [0.0359067055266115,0.117251395665025] |
| overall pork intake | [-0.82,-0.34] | [0.299024127302498,0.667821583893029] |
| overall cheese intake | [-2.06,-1.66] | [5.68493805598144e-06,0.000750737652099761] |
| frequency of adding salt to food | [-1.46,-1.12] | [0.00860874506353802,0.0290060026717939] |
| temperature of hot drinks | [-0.69,-0.34] | [0.137746896138799,0.487008254779884] |
| overall alcohol intake | [-0.54,-0.17] | [0.147013653621583,0.665263032808435] |
| among current drinkers, drinks usually with meals: yes, it varies, no | [-0.52,-0.12] | [0.351340016078417,0.828197261190182] |
| milk type: skimmed, semi-skimmed, full cream (QT) | [0.03,1.18] | [0.241480739909868,0.973300211778236] |

**^a^** the minimum value and maximum value of inverse variance weighted estimate;

**^b^** the minimum value and maximum value of P value;

IVW: inverse variance weighted.

**Table S18. Associations between genetically predicted dietary habits and diastolic blood pressure in sensitivity analysis.**

| **Exposure** | **Weighted median** | | **Mode-based** | | **MR-Egger** | | | | **MRPRESSO** | | |
| --- | --- | --- | --- | --- | --- | --- | --- | --- | --- | --- | --- |
|  | **Beta (95% CI)** | **P value** | **Beta (95% CI)** | **P value** | **Beta (95% CI)** | **P value** | **Intercept** | **P value** | **Beta** | **P value** | **No. of outliers** |
| alcohol drinker status: current + former vs. never | 0.71(-1.02,2.45) | 0.421063 | 0.61(-0.96,2.17) | 0.447624 | 1.3(-3.56,6.17) | 0.599613 | -0.02(-0.05,0.02) | 0.380023 | -0.34 | 0.665174 | 2 |
| alcohol drinker status: current vs. never | 0.05(-1.67,1.76) | 0.956562 | 0.15(-1.37,1.67) | 0.842639 | 1.25(-3.59,6.08) | 0.613283 | -0.02(-0.05,0.02) | 0.318892 | -1.01 | 0.175515 | 3 |
| among current drinkers, drinks usually with meals: yes + it varies vs. no | -0.63(-1.14,-0.11) | 0.017057 | -0.49(-1.02,0.05) | 0.07365 | 1.73(-1.39,4.85) | 0.276678 | -0.03(-0.07,0.01) | 0.126 | -0.4 | 0.094874 | 14 |
| among current drinkers, drinks usually with meals: yes vs. no | 0.13(-0.18,0.44) | 0.427479 | 0.16(-0.19,0.52) | 0.363515 | 0.28(-1.43,1.99) | 0.749277 | -0.01(-0.04,0.02) | 0.620129 | 0.12 | 0.386197 | 16 |
| never eat eggs vs. no eggs, dairy, wheat, or sugar restrictions | 2.85(-0.47,6.17) | 0.092542 | 1.81(-1.13,4.75) | 0.22778 | 3.12(-15.69,21.93) | 0.745016 | 0.01(-0.13,0.14) | 0.934202 | 2.24 | 0.234047 | 3 |
| never eat eggs vs. no eggs restrictions | 1.38(-2.95,5.7) | 0.533322 | 0.58(-3.37,4.53) | 0.772927 | 4.28(-19.73,28.29) | 0.726868 | -0.02(-0.16,0.13) | 0.822705 | 0.63 | 0.729146 | 2 |
| never eat dairy vs. no eggs, dairy, wheat, or sugar restrictions | -1.3(-4.24,1.65) | 0.387878 | -1.79(-4.64,1.05) | 0.217386 | 4.53(-3.29,12.36) | 0.256208 | -0.05(-0.1,0.01) | 0.086061 | -1.91 | 0.217591 | 1 |
| never eat dairy vs. no dairy restrictions | -1.43(-5.09,2.22) | 0.441937 | -2.4(-5.87,1.07) | 0.175531 | 5.44(-4,14.87) | 0.2589 | -0.05(-0.1,0.01) | 0.089599 | -2.24 | 0.231603 | 1 |
| never eat wheat vs. no eggs, dairy, wheat, or sugar restrictions | -8.74(-10.2,-7.29) | 5.61E-32 | -7.95(-8.82,-7.09) | 5.67E-73 | -11.31(-14.73,-7.89) | 9.30E-11 | 0.09(0.02,0.15) | 0.011265 | -7.98 | 1.72E-06 | 4 |
| never eat wheat vs. no wheat restrictions | -10.55(-12.36,-8.73) | 6.47E-30 | -9.81(-10.9,-8.71) | 5.97E-69 | -12.64(-16.39,-8.9) | 3.73E-11 | 0.06(0,0.12) | 0.04396 | -9.11 | 3.04E-06 | 5 |
| never eat sugar vs. no eggs, dairy, wheat, or sugar restrictions | -0.04(-0.57,0.48) | 0.868072 | -0.09(-0.68,0.5) | 0.775522 | -3.37(-6.75,0.02) | 0.051419 | 0.04(0,0.08) | 0.05105 | -0.19 | 0.464779 | 18 |
| never eat sugar vs. no sugar restrictions | -0.05(-0.61,0.52) | 0.872902 | -0.18(-0.79,0.42) | 0.556742 | -2.5(-5.97,0.97) | 0.158478 | 0.03(-0.01,0.07) | 0.191814 | -0.26 | 0.32002 | 21 |
| milk type: dairy-based milk vs. never | 0.35(-2.09,2.79) | 0.777619 | 0.08(-2.19,2.35) | 0.944854 | -1.28(-11.7,9.13) | 0.809039 | 0(-0.07,0.08) | 0.917888 | -0.98 | 0.320941 | 3 |
| milk type: any milk vs. never | 0.3(-2.26,2.87) | 0.81697 | 0.64(-1.71,2.98) | 0.59493 | 6.23(-2.85,15.31) | 0.178689 | -0.06(-0.13,0.01) | 0.112591 | -0.3 | 0.778339 | 2 |
| milk type: full cream vs. never | 0.3(-0.01,0.62) | 0.058443 | 0.2(-0.12,0.52) | 0.230514 | -0.05(-0.95,0.85) | 0.916989 | 0.01(-0.03,0.05) | 0.542334 | 0.22 | 0.093429 | 0 |
| milk type: full cream vs. any other | 1.99(0.36,3.62) | 0.016675 | 1.23(-0.13,2.6) | 0.077039 | 5.44(-4.5,15.38) | 0.283321 | -0.03(-0.12,0.05) | 0.476643 | 1.51 | 0.054644 | 5 |
| milk type: semi-skimmed vs. never | 0.62(-1.27,2.51) | 0.52134 | 0.57(-1.15,2.29) | 0.513377 | -0.52(-8.99,7.94) | 0.903292 | 0(-0.09,0.09) | 0.985012 | -0.1 | 0.917956 | 2 |
| milk type: semi-skimmed vs. any other | -1.22(-2.21,-0.22) | 0.016607 | -0.6(-1.61,0.41) | 0.246851 | -0.62(-4.07,2.83) | 0.725436 | 0(-0.05,0.05) | 0.969067 | -0.68 | 0.159318 | 1 |
| milk type: skimmed vs. never | 0.01(-0.63,0.66) | 0.967678 | 0.16(-0.45,0.78) | 0.603854 | 0.01(-2.03,2.06) | 0.988629 | 0(-0.05,0.05) | 0.880024 | 0.1 | 0.712479 | 1 |
| milk type: skimmed vs. any other | -0.57(-1.63,0.49) | 0.294 | -0.45(-1.4,0.51) | 0.360548 | -2.28(-8.65,4.09) | 0.48233 | 0.02(-0.06,0.1) | 0.633249 | -0.56 | 0.301783 | 6 |
| milk type: soy milk vs. never | 0.12(-0.15,0.38) | 0.392032 | 0.03(-0.22,0.28) | 0.832676 | 0.04(-1.07,1.15) | 0.942202 | 0.01(-0.06,0.08) | 0.776246 | 0.06 | 0.663688 | 1 |
| milk type: soy milk vs. any other | -0.46(-2.55,1.63) | 0.665823 | -0.77(-2.86,1.32) | 0.470222 | 0.52(-5.5,6.53) | 0.866415 | 0(-0.05,0.04) | 0.875051 | 0.06 | 0.951202 | 0 |
| milk type: other milk vs. never | 0.31(-0.01,0.63) | 0.061159 | 0.25(-0.05,0.56) | 0.106871 | 0.27(-0.38,0.92) | 0.418778 | 0(-0.05,0.06) | 0.94035 | 0.29 | 0.05356 | 0 |
| milk type: other milk vs. any other | 2.27(-2.62,7.17) | 0.363117 | 1.87(-2.74,6.48) | 0.42633 | -4.52(-12.67,3.62) | 0.276573 | 0.05(-0.01,0.1) | 0.07707 | 2.14 | 0.300771 | 0 |
| spread type: all spreads vs. never | 1.2(0.2,2.19) | 0.01836 | 1.36(0.3,2.41) | 0.011866 | 0.48(-3.44,4.4) | 0.809413 | 0.01(-0.03,0.06) | 0.477339 | 1.56 | 0.000611 | 5 |
| spread type: butter + margarine vs. never | 1.46(0.82,2.1) | 7.32E-06 | 1.34(0.69,2) | 5.09E-05 | 2.01(-1.33,5.35) | 0.238255 | 0(-0.05,0.05) | 0.932659 | 1.34 | 1.18E-05 | 5 |
| spread type: any oil based spread vs. never | 0.54(-0.1,1.17) | 0.096404 | 0.39(-0.22,0.99) | 0.208551 | 0.9(-3.52,5.32) | 0.688996 | -0.01(-0.1,0.08) | 0.870854 | 0.18 | 0.447506 | 8 |
| spread type: butter and butter-like spreads vs. oil-based spreads | 0.41(-0.27,1.1) | 0.23558 | 0.48(-0.2,1.16) | 0.162617 | -0.66(-7.83,6.51) | 0.856227 | 0.03(-0.09,0.14) | 0.650366 | 0.37 | 0.171248 | 6 |
| spread type: butter and margarine spreads vs. oil-based spreads | 0.26(-0.33,0.86) | 0.384163 | 0.34(-0.23,0.9) | 0.243422 | -0.83(-6.28,4.62) | 0.766484 | 0.02(-0.07,0.12) | 0.623153 | 0.61 | 0.032861 | 10 |
| spread type: butter vs. never | 1.15(0.68,1.63) | 1.79E-06 | 0.97(0.49,1.45) | 6.87E-05 | 1.18(-1.04,3.39) | 0.296729 | 0(-0.04,0.05) | 0.861471 | 1.04 | 1.74E-07 | 4 |
| spread type: butter vs. any other | -0.24(-0.79,0.3) | 0.384902 | -0.05(-0.61,0.5) | 0.847435 | 3.01(-1.3,7.33) | 0.170955 | -0.05(-0.11,0.01) | 0.107306 | -0.35 | 0.2124 | 14 |
| spread type: tub margarine vs. never | 0.51(0.09,0.94) | 0.017948 | 0.37(-0.04,0.77) | 0.073817 | 0.56(-1.13,2.24) | 0.516462 | 0(-0.07,0.06) | 0.877216 | 0.33 | 0.094956 | 4 |
| spread type: tub margarine vs. any other | -0.46(-2.08,1.16) | 0.58035 | -0.18(-1.87,1.51) | 0.83695 | 0.43(-3.75,4.61) | 0.840366 | 0(-0.03,0.04) | 0.805906 | 0.6 | 0.435375 | 1 |
| spread type: flora + benecol vs. never | 0.12(-0.31,0.55) | 0.578524 | 0.12(-0.26,0.5) | 0.537877 | 0.33(-1.81,2.47) | 0.761037 | 0(-0.08,0.08) | 0.940627 | 0.17 | 0.36148 | 6 |
| spread type: flora + benecol vs. any other | 0.31(-0.81,1.43) | 0.589544 | 0.2(-0.74,1.14) | 0.680682 | -0.14(-3.81,3.52) | 0.938767 | -0.01(-0.05,0.04) | 0.819872 | -0.19 | 0.681014 | 5 |
| spread type: olive oil spread vs. never | 0.43(-0.07,0.92) | 0.093256 | 0.23(-0.22,0.69) | 0.307664 | -1.75(-3.96,0.46) | 0.120388 | 0.08(0.01,0.14) | 0.026502 | 0.45 | 0.054924 | 3 |
| spread type: olive oil spread vs. any other | -1.25(-2.86,0.37) | 0.130332 | -1.48(-3.02,0.06) | 0.059386 | -4.37(-10.26,1.51) | 0.145362 | 0.06(-0.02,0.14) | 0.119116 | -1.42 | 0.029753 | 3 |
| spread type: other oil-based spread vs. never | 0.34(-0.1,0.77) | 0.133527 | 0.14(-0.27,0.55) | 0.499392 | 0.45(-2.23,3.12) | 0.742599 | 0(-0.08,0.07) | 0.911994 | 0.15 | 0.413449 | 7 |
| spread type: other oil-based spread vs. any other | 0.66(-0.58,1.9) | 0.297505 | 0.81(-0.33,1.95) | 0.161853 | 2.53(-3.86,8.93) | 0.437589 | -0.03(-0.11,0.05) | 0.436275 | -0.13 | 0.826993 | 3 |
| spread type: low fat spread vs. never | -0.08(-0.61,0.46) | 0.776875 | -0.06(-0.59,0.46) | 0.816678 | -0.56(-1.56,0.45) | 0.275311 | 0.02(-0.02,0.06) | 0.294689 | -0.07 | 0.749836 | 0 |
| spread type: low fat spread vs. any other | -1.55(-4.06,0.95) | 0.224445 | -0.65(-2.84,1.54) | 0.559417 | -14.2(-39.33,10.92) | 0.267901 | 0.08(-0.13,0.29) | 0.454517 | -2.43 | 0.069459 | 4 |
| bread type: white vs. any other | 1.2(0.76,1.64) | 7.29E-08 | 1.05(0.54,1.57) | 6.59E-05 | 0.93(-1.06,2.91) | 0.359421 | 0(-0.02,0.03) | 0.7371 | 1.41 | 2.58E-10 | 10 |
| bread type: brown vs. any other | -1.2(-2.7,0.3) | 0.118235 | -0.26(-1.69,1.17) | 0.723266 | -1.1(-7.02,4.81) | 0.714151 | 0(-0.06,0.07) | 0.972984 | -0.32 | 0.664279 | 1 |
| bread type: wholemeal/wholegrain vs. any other | -1.04(-1.46,-0.62) | 1.58E-06 | -1.04(-1.52,-0.56) | 2.00E-05 | -1.43(-3.88,1.03) | 0.254119 | 0.01(-0.03,0.04) | 0.66918 | -1.11 | 1.06E-07 | 16 |
| bread type: white vs. wholemeal/wholegrain + brown | 1.13(0.73,1.52) | 2.66E-08 | 0.89(0.4,1.37) | 0.000326 | 0.94(-0.84,2.72) | 0.300178 | 0(-0.02,0.03) | 0.8653 | 1.11 | 1.37E-08 | 15 |
| bread type: wholemeal/wholegrain vs. white + brown | -1.05(-1.43,-0.67) | 4.98E-08 | -1.1(-1.53,-0.66) | 6.76E-07 | -2.2(-4.14,-0.26) | 0.026234 | 0.02(-0.01,0.05) | 0.250043 | -1.29 | 2.84E-11 | 13 |
| cereal type: biscuit cereal vs. any other | 0.91(-0.3,2.12) | 0.140161 | 0.52(-0.55,1.6) | 0.340403 | 5.29(0.27,10.31) | 0.038952 | -0.06(-0.14,0.01) | 0.103939 | 1.24 | 0.064914 | 5 |
| cereal type: bran cereal vs. any other | -0.79(-2.02,0.44) | 0.209198 | -0.31(-1.45,0.83) | 0.589971 | 1.87(-4.04,7.78) | 0.534597 | -0.05(-0.13,0.03) | 0.239133 | -0.47 | 0.387934 | 1 |
| cereal type: oat cereal vs. any other | 0.16(-0.82,1.13) | 0.751211 | 0.54(-0.43,1.5) | 0.274678 | -2.47(-6.26,1.32) | 0.201991 | 0.04(-0.02,0.1) | 0.208589 | 0.64 | 0.089179 | 4 |
| cereal type: muesli vs. any other | -1.61(-2.18,-1.03) | 3.67E-08 | -1.45(-2.02,-0.88) | 5.58E-07 | 5.09(0.63,9.55) | 0.025313 | -0.08(-0.14,-0.02) | 0.009059 | -1.07 | 0.000107 | 13 |
| cereal type: cornflakes/frosties vs. any other | 0.81(0.15,1.48) | 0.016635 | 0.9(0.24,1.56) | 0.007539 | -5.28(-8.69,-1.87) | 0.002409 | 0.08(0.03,0.13) | 0.001903 | 0.48 | 0.098489 | 15 |
| coffee type: decaffeinated vs. any other | -0.99(-1.41,-0.56) | 5.90E-06 | -0.79(-1.27,-0.31) | 0.001292 | 0.24(-3.12,3.61) | 0.886586 | -0.02(-0.07,0.03) | 0.495429 | -0.89 | 2.28E-05 | 21 |
| coffee type: ground+instant vs. other+decaff | -0.33(-1.28,0.62) | 0.491245 | -0.5(-1.35,0.34) | 0.242523 | 6.28(0.79,11.78) | 0.025073 | -0.09(-0.18,0.01) | 0.065314 | 0.02 | 0.954188 | 5 |
| coffee type: ground vs. any other | -0.68(-1.68,0.32) | 0.184723 | -0.34(-1.27,0.58) | 0.465066 | -5.23(-11.2,0.74) | 0.08593 | 0.05(-0.05,0.16) | 0.314724 | -0.88 | 0.05174 | 6 |
| tablespoons of cooked vegetables per day | -0.46(-0.99,0.07) | 0.085755 | -0.37(-1,0.26) | 0.247594 | 1.77(-1.12,4.66) | 0.229123 | -0.03(-0.06,0.01) | 0.164636 | -0.18 | 0.484997 | 20 |
| tablespoons of raw vegetables per day | -0.01(-0.49,0.46) | 0.957479 | -0.24(-0.79,0.3) | 0.382979 | 2.23(-0.37,4.83) | 0.093271 | -0.02(-0.06,0.01) | 0.163444 | 0.37 | 0.083346 | 23 |
| pieces of fresh fruit per day | -0.58(-0.96,-0.21) | 0.002117 | -0.55(-0.93,-0.17) | 0.004264 | -0.11(-1.73,1.52) | 0.896697 | 0(-0.03,0.02) | 0.734201 | -0.32 | 0.062409 | 20 |
| pieces of dried fruit per day | -0.93(-1.46,-0.39) | 0.000653 | -0.72(-1.29,-0.15) | 0.0136 | -0.45(-2.7,1.81) | 0.697456 | 0(-0.03,0.03) | 0.987512 | -0.61 | 0.017024 | 9 |
| slices of bread per week | 0.16(-0.32,0.64) | 0.505648 | 0.16(-0.37,0.7) | 0.553693 | -0.36(-2.62,1.91) | 0.758559 | 0.01(-0.02,0.04) | 0.571996 | 0.06 | 0.762349 | 14 |
| bowls of cereal per week | -0.61(-1.04,-0.19) | 0.004801 | -0.62(-1.14,-0.09) | 0.020863 | 0.6(-1.82,3.02) | 0.627962 | -0.01(-0.04,0.02) | 0.574444 | -0.61 | 0.002161 | 27 |
| cups of tea per day | 0.14(-0.29,0.56) | 0.531764 | -0.12(-0.57,0.34) | 0.62037 | -1.37(-2.91,0.16) | 0.078615 | 0.02(-0.01,0.04) | 0.143524 | 0.08 | 0.666419 | 16 |
| cups of coffee per day | -1.19(-1.72,-0.66) | 1.07E-05 | -0.78(-1.12,-0.43) | 1.10E-05 | -0.8(-2.34,0.74) | 0.310724 | -0.01(-0.03,0.02) | 0.464629 | -1.23 | 1.31E-07 | 23 |
| glasses of water per day | 0.15(-0.19,0.5) | 0.37898 | 0.03(-0.44,0.5) | 0.902195 | 0.62(-1.01,2.25) | 0.456199 | -0.01(-0.03,0.02) | 0.624293 | 0 | 0.992704 | 24 |
| champagne/white wine glasses per month | 0.48(-0.16,1.12) | 0.142351 | 0.35(-0.26,0.95) | 0.266702 | 1.42(-1.59,4.43) | 0.354728 | -0.01(-0.06,0.03) | 0.549477 | 0.14 | 0.624175 | 10 |
| red wine glasses per month | -0.5(-0.96,-0.04) | 0.031882 | -0.41(-0.87,0.06) | 0.087812 | 0.73(-1.12,2.59) | 0.437799 | -0.02(-0.04,0.01) | 0.248969 | -0.42 | 0.044237 | 13 |
| beer/cider glasses per month | 0.86(0.34,1.38) | 0.001207 | 1.05(0.59,1.5) | 7.19E-06 | 2.24(0.22,4.25) | 0.02933 | -0.02(-0.05,0.01) | 0.232022 | 1.03 | 2.84E-05 | 14 |
| spirits measures per month | -0.29(-1.09,0.52) | 0.483226 | -0.22(-1.03,0.58) | 0.583616 | 0.7(-1.85,3.24) | 0.590597 | -0.02(-0.06,0.02) | 0.276367 | -0.38 | 0.268042 | 5 |
| fortwine glasses per month | 1(-0.45,2.45) | 0.177133 | 0.96(-0.41,2.33) | 0.170248 | -1.08(-5.27,3.11) | 0.613505 | 0.03(-0.03,0.09) | 0.32308 | 1.38 | 0.061563 | 1 |
| other alcohol glasses per month | -1.12(-2.16,-0.08) | 0.035544 | -1.07(-2.09,-0.05) | 0.040719 | -1.07(-3.47,1.33) | 0.383181 | -0.01(-0.06,0.05) | 0.778264 | -1.39 | 0.002781 | 0 |
| total drinks of alcohol per month | 0.35(-0.07,0.77) | 0.105743 | 0.28(-0.04,0.59) | 0.082305 | 1.29(0.34,2.24) | 0.008044 | -0.02(-0.03,0) | 0.015706 | 0.18 | 0.226412 | 30 |
| overall oily fish intake | 0.31(-0.07,0.69) | 0.112864 | 0.21(-0.27,0.7) | 0.388646 | 3.04(0.69,5.38) | 0.011114 | -0.03(-0.06,0) | 0.04747 | 0.25 | 0.153004 | 22 |
| overall non-oily fish intake | 1.02(0.3,1.73) | 0.005139 | 0.34(-0.42,1.1) | 0.381727 | -0.56(-4.66,3.55) | 0.790508 | 0.03(-0.02,0.09) | 0.276289 | 1.05 | 0.001729 | 14 |
| overall processed meat intake | -0.13(-0.69,0.43) | 0.641532 | 0.08(-0.55,0.71) | 0.805588 | -0.29(-3.09,2.5) | 0.8366 | 0(-0.04,0.04) | 0.962739 | -0.13 | 0.636702 | 15 |
| overall poultry intake | -0.43(-1.22,0.36) | 0.28351 | -0.48(-1.26,0.3) | 0.226691 | 0.78(-2.52,4.08) | 0.642986 | -0.01(-0.05,0.03) | 0.593442 | -0.18 | 0.600885 | 10 |
| overall beef intake | 0.82(0.25,1.38) | 0.004423 | 0.95(0.34,1.55) | 0.002157 | 0.52(-1.71,2.75) | 0.649296 | 0(-0.03,0.03) | 0.912393 | 0.88 | 0.000961 | 12 |
| overall lamb/mutton intake | -0.09(-0.63,0.44) | 0.726057 | 0.13(-0.44,0.7) | 0.660261 | 0.48(-2.16,3.13) | 0.719619 | -0.01(-0.05,0.02) | 0.435287 | -0.08 | 0.75563 | 13 |
| overall pork intake | 0.27(-0.39,0.93) | 0.418636 | 0.18(-0.5,0.86) | 0.605071 | 0.75(-2.2,3.69) | 0.619312 | -0.01(-0.05,0.03) | 0.562966 | 0.03 | 0.928112 | 6 |
| overall cheese intake | -0.58(-0.96,-0.19) | 0.00371 | -0.34(-0.83,0.15) | 0.177746 | -0.19(-2.24,1.85) | 0.854058 | -0.01(-0.03,0.02) | 0.704604 | -0.61 | 0.001226 | 18 |
| frequency of adding salt to food | -0.2(-0.55,0.15) | 0.267101 | -0.05(-0.46,0.36) | 0.802209 | 0.09(-2.11,2.29) | 0.937825 | -0.01(-0.04,0.02) | 0.439219 | -0.25 | 0.108182 | 24 |
| temperature of hot drinks | -0.12(-0.56,0.32) | 0.594453 | -0.17(-0.77,0.44) | 0.590157 | -2.8(-5.1,-0.5) | 0.0171 | 0.03(0,0.06) | 0.040642 | -0.2 | 0.314137 | 21 |
| overall alcohol intake | 0.4(0.01,0.8) | 0.047105 | 0.12(-0.2,0.45) | 0.448193 | 1.03(-0.07,2.13) | 0.066251 | -0.01(-0.03,0) | 0.087874 | 0.05 | 0.732384 | 29 |
| among current drinkers, drinks usually with meals: yes, it varies, no | -0.07(-0.52,0.38) | 0.755458 | -0.1(-0.62,0.42) | 0.716991 | 1.03(-1.39,3.46) | 0.403423 | -0.02(-0.05,0.02) | 0.273179 | -0.08 | 0.699457 | 14 |
| milk type: skimmed, semi-skimmed, full cream (QT) | 0.7(-0.12,1.52) | 0.092312 | 0.66(-0.07,1.4) | 0.075188 | 2.9(-1.4,7.21) | 0.186422 | -0.03(-0.09,0.02) | 0.256748 | 0.4 | 0.294208 | 7 |

MR: mendelian randomization; MRPRESSO: MR pleiotropy residual sum and outlier; OR: odd ratio; CI: confidence interval; No.: number.

**Table S19. Leave-one-out analysis of association between genetically predicted dietary habits and diastolic blood pressure.**

| **Exposure** | **IVW Estimate**  **[Min, Max] ^a^** | **P value [Min, Max] ^b^** |
| --- | --- | --- |
| alcohol drinker status: current + former vs. never | [-1.15,-0.02] | [0.289888864545421,0.980980681249566] |
| alcohol drinker status: current vs. never | [-1.51,-0.39] | [0.149667922461121,0.636146727661748] |
| among current drinkers, drinks usually with meals: yes + it varies vs. no | [-0.75,-0.5] | [0.0383476663605245,0.155492486975489] |
| among current drinkers, drinks usually with meals: yes vs. no | [-0.21,-0.07] | [0.323311534583399,0.752019239841941] |
| never eat eggs vs. no eggs, dairy, wheat, or sugar restrictions | [2.49,5.3] | [0.0297390300945096,0.350915013834716] |
| never eat eggs vs. no eggs restrictions | [-1.53,3.88] | [0.24414127978305,0.792326444356739] |
| never eat dairy vs. no eggs, dairy, wheat, or sugar restrictions | [-2.63,-1.14] | [0.0577664863253059,0.415621788375074] |
| never eat dairy vs. no dairy restrictions | [-3.13,-1.46] | [0.0612217788003714,0.39972787676931] |
| never eat wheat vs. no eggs, dairy, wheat, or sugar restrictions | [-8.16,-7.51] | [1.04053010146834e-17,3.41565962148636e-09] |
| never eat wheat vs. no wheat restrictions | [-9.91,-9.13] | [5.45493930595215e-17,2.24101397635515e-11] |
| never eat sugar vs. no eggs, dairy, wheat, or sugar restrictions | [-0.24,0.22] | [0.574039614350665,0.979076294521585] |
| never eat sugar vs. no sugar restrictions | [-0.41,0.07] | [0.394818510837614,0.872053100946294] |
| milk type: dairy-based milk vs. never | [-1.4,0.28] | [0.342198262335349,0.817435322503088] |
| milk type: any milk vs. never | [-1.41,0.34] | [0.334927028596406,0.833546896548461] |
| milk type: full cream vs. never | [0.17,0.26] | [0.0340135314584088,0.16640132743624] |
| milk type: full cream vs. any other | [1.23,2.29] | [0.0527722501425993,0.234013015349232] |
| milk type: semi-skimmed vs. never | [-0.91,0.33] | [0.445099848362786,0.965445368995186] |
| milk type: semi-skimmed vs. any other | [-0.86,-0.52] | [0.0586259025474086,0.256182922913656] |
| milk type: skimmed vs. never | [-0.27,0.1] | [0.431124566518399,0.9049578519683] |
| milk type: skimmed vs. any other | [-1.06,-0.35] | [0.14805647412231,0.5959084167232] |
| milk type: soy milk vs. never | [0.06,0.24] | [0.206227586383035,0.659180582774039] |
| milk type: soy milk vs. any other | [-0.44,0.44] | [0.60029779934625,0.997679342427106] |
| milk type: other milk vs. never | [0.23,0.37] | [0.00266230830585524,0.0814967470760027] |
| milk type: other milk vs. any other | [0.9,3.37] | [0.0694032647483392,0.63051881424225] |
| spread type: all spreads vs. never | [1.6,2.05] | [0.000249016247115524,0.00498020366127155] |
| spread type: butter + margarine vs. never | [1.65,1.99] | [2.00216684140176e-06,4.9675583890337e-05] |
| spread type: any oil based spread vs. never | [0.31,1.02] | [0.0167377584044458,0.610772590634536] |
| spread type: butter and butter-like spreads vs. oil-based spreads | [-0.09,1.14] | [0.321942228683338,0.821037304606817] |
| spread type: butter and margarine spreads vs. oil-based spreads | [-0.21,0.62] | [0.454594042167215,0.634508407575381] |
| spread type: butter vs. never | [1.19,1.44] | [1.97962805158827e-07,8.06138329128565e-06] |
| spread type: butter vs. any other | [-0.69,-0.09] | [0.166798603325721,0.858573340976685] |
| spread type: tub margarine vs. never | [0.24,0.55] | [0.0454056484113091,0.320239921329896] |
| spread type: tub margarine vs. any other | [0.6,1.19] | [0.123351950155867,0.430208653790169] |
| spread type: flora + benecol vs. never | [0.11,0.55] | [0.0685096828867661,0.775203790527413] |
| spread type: flora + benecol vs. any other | [-0.87,-0.06] | [0.316941255347537,0.933444998604369] |
| spread type: olive oil spread vs. never | [0.3,0.69] | [0.0264008533973294,0.255231936221044] |
| spread type: olive oil spread vs. any other | [-1.35,0.42] | [0.148627377671368,0.993248366975217] |
| spread type: other oil-based spread vs. never | [0.14,0.6] | [0.0384807814195023,0.720699901449481] |
| spread type: other oil-based spread vs. any other | [-0.43,0.79] | [0.32955105011953,0.99833525849223] |
| spread type: low fat spread vs. never | [-0.19,0.07] | [0.344468416840196,0.970739478732141] |
| spread type: low fat spread vs. any other | [-6.26,-0.67] | [0.203872141707011,0.608545662762171] |
| bread type: white vs. any other | [1.18,1.32] | [9.08800176878557e-08,2.00620803940974e-06] |
| bread type: brown vs. any other | [-1.42,-0.32] | [0.142492563786724,0.657961032776896] |
| bread type: wholemeal/wholegrain vs. any other | [-0.99,-0.82] | [0.000275783600181673,0.00325644616941531] |
| bread type: white vs. wholemeal/wholegrain + brown | [1.03,1.15] | [3.58812369739881e-07,6.31646478079612e-06] |
| bread type: wholemeal/wholegrain vs. white + brown | [-1.18,-1.03] | [1.26692818071231e-06,3.65129879415176e-05] |
| cereal type: biscuit cereal vs. any other | [0.81,1.66] | [0.0745896377382441,0.247727768910064] |
| cereal type: bran cereal vs. any other | [-1.75,-0.47] | [0.0888970926905757,0.374786566569386] |
| cereal type: oat cereal vs. any other | [-0.52,0.23] | [0.423694978389766,0.983745588799113] |
| cereal type: muesli vs. any other | [-1.32,-0.88] | [0.00257815666859206,0.0345521817383229] |
| cereal type: cornflakes/frosties vs. any other | [0.26,0.72] | [0.0947795639414428,0.591792339081286] |
| coffee type: decaffeinated vs. any other | [-1.07,-0.78] | [0.00295039477640071,0.051986548431971] |
| coffee type: ground+instant vs. other+decaff | [-0.07,1.76] | [0.116030771234037,0.919191097177125] |
| coffee type: ground vs. any other | [-2.71,-0.68] | [0.0297828177199073,0.369390785014449] |
| tablespoons of cooked vegetables per day | [-0.36,-0.1] | [0.333275207203079,0.792105786895721] |
| tablespoons of raw vegetables per day | [0.32,0.54] | [0.128243449995527,0.348275942464972] |
| pieces of fresh fruit per day | [-0.43,-0.29] | [0.0554468554825045,0.188472691930524] |
| pieces of dried fruit per day | [-0.54,-0.36] | [0.0739476388198842,0.242340297388769] |
| slices of bread per week | [0.14,0.4] | [0.21907284585362,0.669914546956333] |
| bowls of cereal per week | [-0.19,0] | [0.531312408508458,0.992076158228741] |
| cups of tea per day | [-0.39,-0.1] | [0.182128575589006,0.723415711382265] |
| cups of coffee per day | [-1.59,-1.13] | [9.07279923629011e-05,0.00458735675958636] |
| glasses of water per day | [0.1,0.29] | [0.25715080109805,0.690777992775722] |
| champagne/white wine glasses per month | [0.16,0.69] | [0.235187636351866,0.69365231714628] |
| red wine glasses per month | [-0.49,-0.21] | [0.0848156129389537,0.540369242381417] |
| beer/cider glasses per month | [0.82,1.21] | [0.00187172807883496,0.0103080848422065] |
| spirits measures per month | [-0.78,-0.38] | [0.0913062025114444,0.352941548316464] |
| fortwine glasses per month | [0.48,1.38] | [0.0298063971602074,0.498745251031825] |
| other alcohol glasses per month | [-1.52,-1.2] | [0.000500174615769029,0.00615838101125393] |
| total drinks of alcohol per month | [0.02,0.13] | [0.516066529852991,0.935398322103969] |
| overall oily fish intake | [0.65,0.85] | [0.00589935005183712,0.0304239685477902] |
| overall non-oily fish intake | [1.46,1.97] | [0.000333393122824261,0.00725430381065284] |
| overall processed meat intake | [-0.46,-0.2] | [0.218207755685649,0.592976502425136] |
| overall poultry intake | [-0.27,0.08] | [0.572177257607684,0.998657830774607] |
| overall beef intake | [0.53,0.72] | [0.0310425098510757,0.106268759868516] |
| overall lamb/mutton intake | [-0.62,-0.39] | [0.080965921054996,0.241338344525647] |
| overall pork intake | [-0.25,0.07] | [0.516990538964597,0.971011959508778] |
| overall cheese intake | [-0.76,-0.5] | [0.00183092296099808,0.0769825261006934] |
| frequency of adding salt to food | [-0.81,-0.61] | [0.00798630636301022,0.0251304409512209] |
| temperature of hot drinks | [-0.65,-0.41] | [0.0304050200583778,0.167973261957379] |
| overall alcohol intake | [0.01,0.21] | [0.35688611236331,0.978983889002333] |
| among current drinkers, drinks usually with meals: yes, it varies, no | [-0.39,-0.14] | [0.198653493706252,0.627438130677973] |
| milk type: skimmed, semi-skimmed, full cream (QT) | [0.14,0.71] | [0.192712735815159,0.778253619035332] |

**^a^** the minimum value and maximum value of inverse variance weighted estimate;

**^b^** the minimum value and maximum value of P value;

IVW: inverse variance weighted.

**Table S20. Associations between genetically predicted dietary habits and serum total calcium in sensitivity analysis.**

| **Exposure** | **Weighted median** | | **Mode-based** | | **MR-Egger** | | | | **MRPRESSO** | | |
| --- | --- | --- | --- | --- | --- | --- | --- | --- | --- | --- | --- |
|  | **Beta (95% CI)** | **P value** | **Beta (95% CI)** | **P value** | **Beta (95% CI)** | **P value** | **Intercept** | **P value** | **Beta** | **P value** | **No. of outliers** |
| alcohol drinker status: current + former vs. never | 0.02(-0.01,0.04) | 0.148374 | 0.01(-0.01,0.04) | 0.325328 | 0.05(0.01,0.1) | 0.024106 | 0(0,0) | 0.050573 | 0.01 | 0.347845 | 2 |
| alcohol drinker status: current vs. never | 0.01(-0.01,0.03) | 0.340442 | 0.01(-0.01,0.04) | 0.384503 | 0.06(0.01,0.1) | 0.014196 | 0(0,0) | 0.027197 | 0.01 | 0.400457 | 2 |
| among current drinkers, drinks usually with meals: yes + it varies vs. no | 0(-0.01,0) | 0.717793 | 0(-0.01,0.01) | 0.774824 | 0.01(-0.01,0.03) | 0.24191 | 0(0,0) | 0.103443 | 0 | 0.176689 | 3 |
| among current drinkers, drinks usually with meals: yes vs. no | 0(-0.01,0) | 0.04894 | 0(-0.01,0) | 0.175311 | 0(-0.01,0.01) | 0.885174 | 0(0,0) | 0.79799 | 0 | 0.090238 | 3 |
| never eat eggs vs. no eggs, dairy, wheat, or sugar restrictions | 0.02(-0.01,0.05) | 0.282216 | 0.02(-0.01,0.05) | 0.210153 | 0.01(-0.05,0.07) | 0.695569 | 0(0,0) | 0.820192 | 0.01 | 0.626021 | 0 |
| never eat eggs vs. no eggs restrictions | 0(-0.04,0.05) | 0.857523 | 0.01(-0.03,0.06) | 0.554381 | 0.01(-0.09,0.11) | 0.857376 | 0(0,0) | 0.812548 | 0 | 0.913853 | 0 |
| never eat dairy vs. no eggs, dairy, wheat, or sugar restrictions | 0.01(-0.02,0.04) | 0.465892 | 0.01(-0.02,0.04) | 0.372853 | 0.03(-0.06,0.11) | 0.556762 | 0(0,0) | 0.379683 | 0.01 | 0.464168 | 2 |
| never eat dairy vs. no dairy restrictions | 0.01(-0.03,0.05) | 0.698149 | 0.01(-0.03,0.05) | 0.57712 | 0.04(-0.07,0.15) | 0.453908 | 0(0,0) | 0.282582 | 0.01 | 0.50395 | 2 |
| never eat wheat vs. no eggs, dairy, wheat, or sugar restrictions | -0.04(-0.05,-0.03) | 8.04E-12 | -0.03(-0.04,-0.02) | 2.17E-11 | -0.04(-0.07,-0.02) | 0.00015 | 0(0,0) | 0.020446 | -0.03 | 0.000589 | 2 |
| never eat wheat vs. no wheat restrictions | -0.05(-0.06,-0.03) | 2.72E-11 | -0.04(-0.05,-0.03) | 3.13E-12 | -0.05(-0.08,-0.02) | 0.001496 | 0(0,0) | 0.07382 | -0.03 | 0.003859 | 4 |
| never eat sugar vs. no eggs, dairy, wheat, or sugar restrictions | 0.01(0,0.01) | 0.085946 | 0(0,0.01) | 0.245948 | -0.02(-0.04,0.01) | 0.169967 | 0(0,0) | 0.122605 | 0 | 0.554872 | 6 |
| never eat sugar vs. no sugar restrictions | 0.01(0,0.01) | 0.021109 | 0.01(0,0.01) | 0.082625 | -0.01(-0.04,0.01) | 0.341886 | 0(0,0) | 0.188298 | 0 | 0.09684 | 6 |
| milk type: dairy-based milk vs. never | 0(-0.02,0.03) | 0.713987 | 0(-0.02,0.03) | 0.776364 | 0.02(-0.04,0.08) | 0.451715 | 0(0,0) | 0.648897 | 0.01 | 0.31775 | 0 |
| milk type: any milk vs. never | 0.01(-0.02,0.03) | 0.640732 | 0.01(-0.02,0.03) | 0.691508 | 0.04(0,0.09) | 0.077537 | 0(0,0) | 0.127122 | 0.01 | 0.37691 | 0 |
| milk type: full cream vs. never | 0(0,0.01) | 0.01795 | 0(0,0.01) | 0.04331 | 0.01(0,0.01) | 0.181892 | 0(0,0) | 0.482469 | 0 | 0.075894 | 1 |
| milk type: full cream vs. any other | -0.01(-0.02,0.01) | 0.417055 | -0.01(-0.03,0) | 0.115554 | 0(-0.06,0.06) | 0.926304 | 0(0,0) | 0.745499 | -0.01 | 0.027454 | 1 |
| milk type: semi-skimmed vs. never | 0(-0.02,0.02) | 0.961209 | 0(-0.02,0.02) | 0.737416 | 0(-0.05,0.06) | 0.914655 | 0(0,0) | 0.938328 | 0 | 0.920824 | 1 |
| milk type: semi-skimmed vs. any other | 0(-0.01,0.01) | 0.572113 | 0.01(-0.01,0.02) | 0.320984 | 0(-0.03,0.03) | 0.959122 | 0(0,0) | 0.746568 | 0.01 | 0.153462 | 1 |
| milk type: skimmed vs. never | 0.01(0,0.01) | 0.067512 | 0.01(0,0.01) | 0.140495 | 0.01(0,0.02) | 0.079098 | 0(0,0) | 0.592017 | 0.01 | 0.003816 | 0 |
| milk type: skimmed vs. any other | -0.01(-0.02,0) | 0.08604 | -0.01(-0.02,0) | 0.109978 | -0.03(-0.07,0.01) | 0.141269 | 0(0,0) | 0.143705 | 0 | 0.376323 | 4 |
| milk type: soy milk vs. never | 0(0,0) | 0.480137 | 0(0,0) | 0.449975 | 0(-0.01,0.01) | 0.830171 | 0(0,0) | 0.980238 | 0 | 0.54176 | 2 |
| milk type: soy milk vs. any other | 0(-0.03,0.02) | 0.833125 | 0(-0.03,0.02) | 0.805459 | 0.01(-0.1,0.12) | 0.849095 | 0(0,0) | 0.444578 | -0.02 | 0.204929 | 1 |
| milk type: other milk vs. never | 0(0,0) | 0.429299 | 0(0,0) | 0.507861 | 0(-0.01,0.01) | 0.755605 | 0(0,0) | 0.667027 | 0 | 0.873562 | 0 |
| milk type: other milk vs. any other | 0.01(-0.04,0.06) | 0.738661 | 0(-0.05,0.05) | 0.888253 | 0.02(-0.06,0.1) | 0.601263 | 0(0,0) | 0.654666 | 0 | 0.765192 | 0 |
| spread type: all spreads vs. never | 0(-0.01,0.01) | 0.617515 | 0(-0.01,0.02) | 0.740682 | 0.02(-0.01,0.05) | 0.295218 | 0(0,0) | 0.576594 | 0.01 | 0.049869 | 1 |
| spread type: butter + margarine vs. never | 0.01(0,0.01) | 0.132974 | 0.01(0,0.01) | 0.176038 | 0.01(-0.01,0.03) | 0.364595 | 0(0,0) | 0.636589 | 0.01 | 0.019458 | 3 |
| spread type: any oil based spread vs. never | 0(0,0.01) | 0.295996 | 0(0,0.01) | 0.329606 | 0.01(0,0.03) | 0.091344 | 0(0,0) | 0.27101 | 0 | 0.029591 | 0 |
| spread type: butter and butter-like spreads vs. oil-based spreads | 0(-0.01,0.01) | 0.898994 | 0(-0.01,0.01) | 0.832771 | 0(-0.03,0.02) | 0.797582 | 0(0,0) | 0.660256 | 0 | 0.607837 | 2 |
| spread type: butter and margarine spreads vs. oil-based spreads | 0(-0.01,0) | 0.50891 | 0(-0.01,0.01) | 0.677336 | 0(-0.02,0.02) | 0.99865 | 0(0,0) | 0.697542 | 0 | 0.635456 | 4 |
| spread type: butter vs. never | 0.01(0,0.01) | 0.02803 | 0.01(0,0.01) | 0.075935 | 0.01(-0.01,0.03) | 0.418623 | 0(0,0) | 0.687661 | 0 | 0.05221 | 4 |
| spread type: butter vs. any other | 0(-0.01,0) | 0.643791 | 0(-0.01,0.01) | 0.922801 | 0.01(-0.01,0.04) | 0.342335 | 0(0,0) | 0.301836 | 0 | 0.903763 | 6 |
| spread type: tub margarine vs. never | 0(-0.01,0) | 0.704346 | 0(0,0.01) | 0.820647 | -0.01(-0.02,0.01) | 0.343149 | 0(0,0) | 0.240622 | 0 | 0.529156 | 2 |
| spread type: tub margarine vs. any other | 0(-0.02,0.02) | 0.995047 | -0.01(-0.02,0.01) | 0.565332 | -0.01(-0.07,0.04) | 0.643559 | 0(0,0) | 0.450381 | 0 | 0.568325 | 2 |
| spread type: flora + benecol vs. never | 0(0,0.01) | 0.042373 | 0(0,0.01) | 0.03794 | 0(-0.01,0.01) | 0.882835 | 0(0,0) | 0.642005 | 0 | 0.027962 | 2 |
| spread type: flora + benecol vs. any other | 0(-0.01,0.02) | 0.618024 | 0(-0.01,0.01) | 0.811576 | -0.02(-0.06,0.01) | 0.221951 | 0(0,0) | 0.115233 | 0 | 0.648819 | 6 |
| spread type: olive oil spread vs. never | 0.01(0,0.01) | 0.049645 | 0(0,0.01) | 0.156839 | -0.01(-0.02,0.01) | 0.542103 | 0(0,0) | 0.241375 | 0 | 0.10243 | 2 |
| spread type: olive oil spread vs. any other | 0.02(0,0.03) | 0.084583 | 0.01(0,0.03) | 0.140269 | 0.01(-0.03,0.05) | 0.5729 | 0(0,0) | 0.884615 | 0.01 | 0.106373 | 0 |
| spread type: other oil-based spread vs. never | 0(0,0.01) | 0.222069 | 0(0,0.01) | 0.298195 | 0(-0.01,0.01) | 0.758482 | 0(0,0) | 0.795319 | 0 | 0.119834 | 0 |
| spread type: other oil-based spread vs. any other | 0(-0.01,0.02) | 0.926351 | 0(-0.01,0.02) | 0.515139 | 0.01(-0.02,0.05) | 0.519413 | 0(0,0) | 0.33588 | 0 | 0.512159 | 1 |
| spread type: low fat spread vs. never | 0(0,0.01) | 0.438761 | 0(0,0.01) | 0.587913 | 0(-0.01,0.01) | 0.925528 | 0(0,0) | 0.58041 | 0 | 0.211453 | 0 |
| spread type: low fat spread vs. any other | -0.02(-0.04,0.01) | 0.163929 | -0.02(-0.04,0) | 0.107592 | -0.07(-0.19,0.05) | 0.274149 | 0(0,0) | 0.646855 | -0.02 | 0.020006 | 2 |
| bread type: white vs. any other | 0.01(0,0.01) | 0.00082 | 0.01(0,0.01) | 0.037719 | 0.02(0,0.03) | 0.043861 | 0(0,0) | 0.316946 | 0.01 | 0.000447 | 3 |
| bread type: brown vs. any other | 0(-0.02,0.02) | 0.85781 | 0(-0.02,0.01) | 0.761474 | -0.03(-0.07,0.01) | 0.167793 | 0(0,0) | 0.289976 | 0 | 0.661027 | 1 |
| bread type: wholemeal/wholegrain vs. any other | -0.01(-0.01,0) | 0.00305 | 0(-0.01,0) | 0.099373 | -0.01(-0.03,0.01) | 0.213613 | 0(0,0) | 0.723794 | -0.01 | 0.000667 | 4 |
| bread type: white vs. wholemeal/wholegrain + brown | 0.01(0,0.01) | 0.002198 | 0.01(0,0.01) | 0.093654 | 0.02(0,0.03) | 0.02309 | 0(0,0) | 0.189007 | 0.01 | 1.70E-05 | 4 |
| bread type: wholemeal/wholegrain vs. white + brown | -0.01(-0.01,0) | 0.000187 | -0.01(-0.01,0) | 0.026507 | -0.01(-0.03,0.01) | 0.269535 | 0(0,0) | 0.864827 | -0.01 | 0.000436 | 8 |
| cereal type: biscuit cereal vs. any other | 0.01(0,0.02) | 0.127617 | 0.01(0,0.02) | 0.217588 | 0.01(-0.02,0.05) | 0.427271 | 0(0,0) | 0.614325 | 0.01 | 0.128698 | 2 |
| cereal type: bran cereal vs. any other | -0.01(-0.03,0) | 0.154221 | -0.01(-0.03,0) | 0.118079 | 0.01(-0.03,0.05) | 0.638113 | 0(0,0) | 0.351321 | -0.01 | 0.133461 | 1 |
| cereal type: oat cereal vs. any other | 0(-0.01,0.01) | 0.601803 | 0(-0.01,0.01) | 0.909353 | 0.01(-0.02,0.04) | 0.438468 | 0(0,0) | 0.364545 | 0 | 0.481434 | 1 |
| cereal type: muesli vs. any other | -0.01(-0.01,0) | 0.02354 | 0(-0.01,0) | 0.272417 | 0.02(-0.01,0.05) | 0.11294 | 0(0,0) | 0.046689 | -0.01 | 0.006124 | 2 |
| cereal type: cornflakes/frosties vs. any other | 0(0,0.01) | 0.317274 | 0.01(0,0.02) | 0.134039 | -0.02(-0.04,0) | 0.03231 | 0(0,0) | 0.017512 | 0.01 | 0.00998 | 4 |
| coffee type: decaffeinated vs. any other | -0.01(-0.01,0) | 0.002199 | -0.01(-0.01,0) | 0.010152 | 0(-0.02,0.02) | 0.991019 | 0(0,0) | 0.6648 | -0.01 | 0.000226 | 5 |
| coffee type: ground+instant vs. other+decaff | 0(-0.01,0.01) | 0.789887 | 0(-0.01,0.01) | 0.616761 | 0.05(0.01,0.08) | 0.017728 | 0(0,0) | 0.052726 | 0 | 0.685847 | 2 |
| coffee type: ground vs. any other | -0.01(-0.02,0) | 0.080719 | 0(-0.02,0.01) | 0.494076 | -0.04(-0.09,0) | 0.037214 | 0(0,0) | 0.120447 | 0 | 0.567761 | 2 |
| tablespoons of cooked vegetables per day | 0(-0.01,0) | 0.320788 | 0(-0.01,0.01) | 0.613208 | -0.02(-0.04,0.01) | 0.145047 | 0(0,0) | 0.259353 | -0.01 | 0.023636 | 3 |
| tablespoons of raw vegetables per day | 0(-0.01,0) | 0.589292 | 0(-0.01,0) | 0.176656 | -0.01(-0.02,0.01) | 0.565442 | 0(0,0) | 0.678903 | 0 | 0.202178 | 5 |
| pieces of fresh fruit per day | 0(-0.01,0) | 0.06255 | 0(-0.01,0) | 0.141757 | -0.01(-0.03,0) | 0.043668 | 0(0,0) | 0.218177 | -0.01 | 0.001425 | 7 |
| pieces of dried fruit per day | -0.01(-0.02,-0.01) | 6.44E-05 | -0.01(-0.02,-0.01) | 0.000677 | -0.01(-0.03,0.02) | 0.594655 | 0(0,0) | 0.646021 | -0.01 | 0.001677 | 5 |
| slices of bread per week | 0(0,0.01) | 0.806169 | 0(-0.01,0.01) | 0.692403 | 0.01(-0.01,0.04) | 0.260291 | 0(0,0) | 0.374541 | 0 | 0.25194 | 5 |
| bowls of cereal per week | 0(0,0.01) | 0.57403 | 0(-0.01,0.01) | 0.711654 | 0(-0.02,0.02) | 0.640296 | 0(0,0) | 0.73242 | 0 | 0.709282 | 9 |
| cups of tea per day | 0.01(0,0.02) | 0.000252 | 0.01(0,0.01) | 0.002017 | 0.01(0,0.03) | 0.04645 | 0(0,0) | 0.313448 | 0 | 0.004933 | 8 |
| cups of coffee per day | 0.01(0,0.01) | 0.009483 | 0(0,0.01) | 0.21406 | 0(-0.01,0.02) | 0.745827 | 0(0,0) | 0.263853 | 0 | 0.162244 | 9 |
| glasses of water per day | -0.01(-0.01,0) | 4.58E-05 | -0.01(-0.01,-0.01) | 4.46E-05 | -0.01(-0.03,0) | 0.145408 | 0(0,0) | 0.255852 | -0.01 | 0.000233 | 14 |
| champagne/white wine glasses per month | 0(-0.01,0) | 0.480128 | -0.01(-0.02,0) | 0.150369 | 0.03(0,0.07) | 0.086333 | 0(0,0) | 0.043775 | 0 | 0.224601 | 8 |
| red wine glasses per month | 0(-0.01,0) | 0.190829 | -0.01(-0.01,0) | 0.081362 | 0.01(-0.01,0.03) | 0.277429 | 0(0,0) | 0.124229 | -0.01 | 0.003582 | 5 |
| beer/cider glasses per month | 0.01(0,0.01) | 0.008508 | 0(0,0.01) | 0.132342 | 0(-0.02,0.03) | 0.848697 | 0(0,0) | 0.788313 | 0.01 | 0.009806 | 10 |
| spirits measures per month | 0(-0.01,0.01) | 0.725117 | 0(-0.01,0.01) | 0.848106 | 0.01(-0.02,0.04) | 0.530982 | 0(0,0) | 0.568689 | 0 | 0.317629 | 4 |
| fortwine glasses per month | 0(-0.02,0.01) | 0.707148 | 0(-0.02,0.01) | 0.850778 | 0(-0.04,0.04) | 0.966308 | 0(0,0) | 0.769158 | 0 | 0.524736 | 0 |
| other alcohol glasses per month | 0(-0.01,0.01) | 0.603122 | 0(-0.02,0.01) | 0.449054 | -0.02(-0.04,0.01) | 0.143547 | 0(0,0) | 0.28417 | -0.01 | 0.207249 | 0 |
| total drinks of alcohol per month | 0(0,0.01) | 0.30257 | 0.01(0,0.01) | 0.000675 | 0(-0.01,0.01) | 0.511734 | 0(0,0) | 0.276351 | 0 | 0.667764 | 14 |
| overall oily fish intake | 0(0,0) | 0.864823 | 0(-0.01,0) | 0.666539 | 0(-0.02,0.02) | 0.87683 | 0(0,0) | 0.7385 | 0 | 0.430063 | 11 |
| overall non-oily fish intake | 0(-0.01,0.01) | 0.821017 | 0(-0.01,0.01) | 0.86725 | -0.01(-0.05,0.03) | 0.670427 | 0(0,0) | 0.451586 | 0 | 0.526697 | 6 |
| overall processed meat intake | 0(-0.01,0) | 0.491016 | 0(-0.01,0) | 0.182881 | 0.01(-0.01,0.03) | 0.341585 | 0(0,0) | 0.379605 | 0 | 0.930338 | 3 |
| overall poultry intake | 0.01(0,0.02) | 0.082779 | 0.01(0,0.02) | 0.261315 | -0.01(-0.06,0.03) | 0.61708 | 0(0,0) | 0.229838 | 0.01 | 0.007425 | 2 |
| overall beef intake | 0(0,0.01) | 0.403298 | 0(-0.01,0.01) | 0.829974 | 0.02(-0.01,0.04) | 0.159313 | 0(0,0) | 0.414199 | 0 | 0.332172 | 6 |
| overall lamb/mutton intake | 0(0,0.01) | 0.316387 | 0(0,0.01) | 0.369671 | 0.01(-0.01,0.03) | 0.356097 | 0(0,0) | 0.535457 | 0 | 0.251941 | 6 |
| overall pork intake | 0(-0.01,0) | 0.45001 | 0(-0.01,0) | 0.248283 | 0(-0.02,0.02) | 0.925329 | 0(0,0) | 0.691064 | 0 | 0.775191 | 4 |
| overall cheese intake | 0(-0.01,0) | 0.40478 | 0(-0.01,0) | 0.36024 | 0.01(-0.01,0.03) | 0.268645 | 0(0,0) | 0.06776 | -0.01 | 0.000451 | 10 |
| frequency of adding salt to food | 0(0,0) | 0.89307 | 0(-0.01,0) | 0.635552 | 0.01(-0.01,0.02) | 0.332719 | 0(0,0) | 0.422374 | 0 | 0.512243 | 9 |
| temperature of hot drinks | 0(-0.01,0) | 0.575978 | 0(-0.01,0) | 0.660177 | 0(-0.02,0.02) | 0.809425 | 0(0,0) | 0.690589 | 0 | 0.286254 | 9 |
| overall alcohol intake | 0(0,0) | 0.802421 | 0(0,0.01) | 0.30354 | 0(-0.01,0.01) | 0.734793 | 0(0,0) | 0.327588 | 0 | 0.225394 | 13 |
| among current drinkers, drinks usually with meals: yes, it varies, no | 0(-0.01,0) | 0.318814 | 0(-0.01,0) | 0.378154 | 0(-0.01,0.02) | 0.734501 | 0(0,0) | 0.52382 | 0 | 0.675525 | 2 |
| milk type: skimmed, semi-skimmed, full cream (QT) | 0.01(0,0.02) | 0.080016 | 0.01(0,0.02) | 0.146721 | 0.02(-0.01,0.05) | 0.133737 | 0(0,0) | 0.168873 | 0 | 0.259792 | 2 |

MR: mendelian randomization; MRPRESSO: MR pleiotropy residual sum and outlier; OR: odd ratio; CI: confidence interval; No.: number.

**Table S21. Leave-one-out analysis of association between genetically predicted dietary habits and serum total calcium.**

| **Exposure** | **IVW Estimate**  **[Min, Max] ^a^** | **P value [Min, Max] ^b^** |
| --- | --- | --- |
| alcohol drinker status: current + former vs. never | [1,1.02] | [0.0634711668377349,0.816891640047028] |
| alcohol drinker status: current vs. never | [1,1.01] | [0.395641039504026,0.995421177871817] |
| among current drinkers, drinks usually with meals: yes + it varies vs. no | [1,1] | [0.0702967804228851,0.22853944427739] |
| among current drinkers, drinks usually with meals: yes vs. no | [1,1] | [0.0299229742539211,0.243702932754367] |
| never eat eggs vs. no eggs, dairy, wheat, or sugar restrictions | [1,1.01] | [0.246730485513699,0.869247298245518] |
| never eat eggs vs. no eggs restrictions | [0.99,1.01] | [0.471494263886579,0.991935406235429] |
| never eat dairy vs. no eggs, dairy, wheat, or sugar restrictions | [0.98,1] | [0.331030898453135,0.949594643389145] |
| never eat dairy vs. no dairy restrictions | [0.98,1] | [0.319494932311847,0.921051979472813] |
| never eat wheat vs. no eggs, dairy, wheat, or sugar restrictions | [0.97,0.98] | [0.000214361999316422,0.0436366461552854] |
| never eat wheat vs. no wheat restrictions | [0.97,0.98] | [0.000940569768242447,0.0854138941645944] |
| never eat sugar vs. no eggs, dairy, wheat, or sugar restrictions | [1,1] | [0.19536961023357,0.926339745605972] |
| never eat sugar vs. no sugar restrictions | [1,1.01] | [0.0249763166189392,0.370274976760468] |
| milk type: dairy-based milk vs. never | [1.01,1.01] | [0.145308961773513,0.58192387791378] |
| milk type: any milk vs. never | [1,1.01] | [0.185657100520311,0.648540334137573] |
| milk type: full cream vs. never | [1,1] | [0.0139793109556896,0.125151633497655] |
| milk type: full cream vs. any other | [0.99,1] | [0.0212152455009526,0.614776136661237] |
| milk type: semi-skimmed vs. never | [1,1.01] | [0.247753743914993,0.919822258002807] |
| milk type: semi-skimmed vs. any other | [1,1.01] | [0.143060705516192,0.708243404108016] |
| milk type: skimmed vs. never | [1.01,1.01] | [0.000685284037761187,0.00806424783945487] |
| milk type: skimmed vs. any other | [1,1] | [0.470969197026346,0.998637982348105] |
| milk type: soy milk vs. never | [1,1] | [0.261997759106117,0.911350744171685] |
| milk type: soy milk vs. any other | [0.97,0.98] | [0.0625538310672396,0.195406404533489] |
| milk type: other milk vs. never | [1,1] | [0.445999910283173,0.973616733296956] |
| milk type: other milk vs. any other | [1,1.01] | [0.527709003930811,0.979427614933988] |
| spread type: all spreads vs. never | [1.01,1.01] | [0.0445605948384069,0.187262947042006] |
| spread type: butter + margarine vs. never | [1,1.01] | [0.0306889875428213,0.25924304218778] |
| spread type: any oil based spread vs. never | [1,1.01] | [0.0219644480823411,0.0990504911790881] |
| spread type: butter and butter-like spreads vs. oil-based spreads | [1,1] | [0.394362511200836,0.910964421054115] |
| spread type: butter and margarine spreads vs. oil-based spreads | [1,1] | [0.159008357430883,0.467209560586265] |
| spread type: butter vs. never | [1,1.01] | [0.0432906598448838,0.284137551622332] |
| spread type: butter vs. any other | [1,1] | [0.414754339973364,0.996366166543978] |
| spread type: tub margarine vs. never | [1,1] | [0.255247488224334,0.933654536351011] |
| spread type: tub margarine vs. any other | [1,1.01] | [0.253956269508798,0.921168916405683] |
| spread type: flora + benecol vs. never | [1,1.01] | [0.0160153906572864,0.298816600306348] |
| spread type: flora + benecol vs. any other | [1,1.01] | [0.271430516001356,0.999670923597478] |
| spread type: olive oil spread vs. never | [1,1.01] | [0.0522084280594291,0.311657484592431] |
| spread type: olive oil spread vs. any other | [1.01,1.02] | [0.00206119420335213,0.214532424258641] |
| spread type: other oil-based spread vs. never | [1,1] | [0.0630614579146361,0.247970760603826] |
| spread type: other oil-based spread vs. any other | [0.99,1] | [0.261250864079539,0.783208817196484] |
| spread type: low fat spread vs. never | [1,1] | [0.085614164839746,0.429780235377698] |
| spread type: low fat spread vs. any other | [0.95,0.99] | [0.0756309332664571,0.188735524411131] |
| bread type: white vs. any other | [1.01,1.01] | [2.30690547383548e-05,0.000120017045702162] |
| bread type: brown vs. any other | [0.99,1] | [0.164263103141812,0.656247080706238] |
| bread type: wholemeal/wholegrain vs. any other | [0.99,0.99] | [8.65266046828377e-05,0.0010228627342286] |
| bread type: white vs. wholemeal/wholegrain + brown | [1.01,1.01] | [4.62961032654304e-05,0.000247872744340786] |
| bread type: wholemeal/wholegrain vs. white + brown | [0.99,0.99] | [2.809113266657e-05,0.000994580071688194] |
| cereal type: biscuit cereal vs. any other | [1,1.01] | [0.0651476628810477,0.648617133674306] |
| cereal type: bran cereal vs. any other | [0.99,1] | [0.116049410426872,0.553995732805128] |
| cereal type: oat cereal vs. any other | [1,1] | [0.47552860457159,0.996820683420687] |
| cereal type: muesli vs. any other | [0.99,1] | [0.00236830687659981,0.262374623724965] |
| cereal type: cornflakes/frosties vs. any other | [1,1.01] | [0.0679768034033363,0.520740779087231] |
| coffee type: decaffeinated vs. any other | [0.99,1] | [0.0110330886542366,0.166537826904767] |
| coffee type: ground+instant vs. other+decaff | [1,1.01] | [0.0888369324853047,0.997671968392787] |
| coffee type: ground vs. any other | [0.98,1] | [0.0674338602495088,0.831733175883454] |
| tablespoons of cooked vegetables per day | [1,1] | [0.0571472626391285,0.210244284018487] |
| tablespoons of raw vegetables per day | [1,1] | [0.303044512162975,0.71677554658379] |
| pieces of fresh fruit per day | [0.99,1] | [0.00155619348765402,0.00819058383118672] |
| pieces of dried fruit per day | [0.99,0.99] | [0.000219603973869421,0.00148461575596545] |
| slices of bread per week | [1,1] | [0.236932624682731,0.99127636137942] |
| bowls of cereal per week | [1,1] | [0.469842689662531,0.899193038487205] |
| cups of tea per day | [1.01,1.01] | [0.000401092263187042,0.00927119047652476] |
| cups of coffee per day | [0.99,1] | [0.0646901205796976,0.563111814530203] |
| glasses of water per day | [1,1] | [0.0533359645455904,0.335703170697911] |
| champagne/white wine glasses per month | [0.99,1] | [0.248069195432721,0.734305141910472] |
| red wine glasses per month | [0.99,1] | [0.0788375036424019,0.877234023056965] |
| beer/cider glasses per month | [1,1.01] | [0.0222024739245045,0.449385851245224] |
| spirits measures per month | [1,1] | [0.391176760326388,0.957074659705268] |
| fortwine glasses per month | [1,1.01] | [0.186130008678609,0.827085685256701] |
| other alcohol glasses per month | [0.99,1] | [0.0677458818662483,0.439812957852195] |
| total drinks of alcohol per month | [1,1] | [0.113934666488973,0.69074772909079] |
| overall oily fish intake | [1,1] | [0.377676176258656,0.863076594170166] |
| overall non-oily fish intake | [1,1.01] | [0.262444572623881,0.888505329255892] |
| overall processed meat intake | [1,1] | [0.53383489224357,0.940197364741404] |
| overall poultry intake | [1.01,1.02] | [0.017961900783847,0.0532977306514394] |
| overall beef intake | [1.01,1.01] | [0.0226243710693277,0.0846525983120685] |
| overall lamb/mutton intake | [1,1] | [0.126247518314264,0.383904584556753] |
| overall pork intake | [1,1] | [0.240623421428256,0.552463651294343] |
| overall cheese intake | [0.99,1] | [0.00169126451524668,0.0533386192265543] |
| frequency of adding salt to food | [1,1] | [0.334739045440709,0.656867713998253] |
| temperature of hot drinks | [1,1] | [0.426540769518718,0.815604832566864] |
| overall alcohol intake | [0.99,1] | [0.0246057697878934,0.906390063800942] |
| among current drinkers, drinks usually with meals: yes, it varies, no | [1,1] | [0.243337382485194,0.503286068142925] |
| milk type: skimmed, semi-skimmed, full cream (QT) | [1,1.01] | [0.141357381710312,0.776275248518513] |

**^a^** the minimum value and maximum value of inverse variance weighted estimate;

**^b^** the minimum value and maximum value of P value;

IVW: inverse variance weighted.

**Table S22. Associations between genetically predicted dietary habits and neuroticism in sensitivity analysis.**

| **Exposure** | **Weighted median** | | **Mode-based** | | **MR-Egger** | | | | **MRPRESSO** | | |
| --- | --- | --- | --- | --- | --- | --- | --- | --- | --- | --- | --- |
|  | **OR (95% CI)** | **P value** | **OR (95% CI)** | **P value** | **OR (95% CI)** | **P value** | **Intercept** | **P value** | **OR** | **P value** | **No. of outliers** |
| alcohol drinker status: current + former vs. never | 0.95(0.74,1.21) | 0.671183 | 0.95(0.77,1.18) | 0.675521 | 1.05(0.65,1.67) | 0.853078 | 0(0,0) | 0.825987 | 1 | 0.986758 | 1 |
| alcohol drinker status: current vs. never | 0.94(0.74,1.19) | 0.614038 | 0.97(0.78,1.2) | 0.775953 | 0.99(0.61,1.59) | 0.95062 | 0(0,0) | 0.94361 | 1 | 0.99889 | 1 |
| among current drinkers, drinks usually with meals: yes + it varies vs. no | 0.9(0.84,0.96) | 0.00205 | 0.95(0.87,1.03) | 0.186816 | 0.96(0.71,1.29) | 0.767144 | 0(-0.01,0) | 0.435162 | 0.9 | 0.001128 | 8 |
| among current drinkers, drinks usually with meals: yes vs. no | 0.87(0.83,0.91) | 4.90E-11 | 0.91(0.86,0.96) | 0.000775 | 0.96(0.81,1.13) | 0.617183 | 0(-0.01,0) | 0.303602 | 0.9 | 1.22E-06 | 10 |
| never eat eggs vs. no eggs, dairy, wheat, or sugar restrictions | 1.43(0.99,2.07) | 0.058038 | 1.42(0.99,2.04) | 0.058694 | 1.84(0.51,6.58) | 0.350012 | 0(-0.01,0.01) | 0.417886 | 1.33 | 0.050578 | 1 |
| never eat eggs vs. no eggs restrictions | 1.51(0.92,2.48) | 0.102873 | 1.43(0.88,2.34) | 0.151781 | 1.1(0.36,3.3) | 0.87085 | 0(-0.01,0.01) | 0.747202 | 1.3 | 0.205211 | 0 |
| never eat dairy vs. no eggs, dairy, wheat, or sugar restrictions | 1.14(0.83,1.57) | 0.42114 | 1.02(0.76,1.36) | 0.92017 | 0.58(0.26,1.31) | 0.186876 | 0.01(0,0.01) | 0.094645 | 0.96 | 0.703726 | 1 |
| never eat dairy vs. no dairy restrictions | 1.16(0.78,1.73) | 0.462527 | 0.97(0.67,1.4) | 0.872978 | 0.49(0.18,1.33) | 0.160589 | 0.01(0,0.01) | 0.074898 | 0.95 | 0.745873 | 1 |
| never eat wheat vs. no eggs, dairy, wheat, or sugar restrictions | 0.66(0.57,0.76) | 2.33E-09 | 0.63(0.57,0.7) | 5.62E-19 | 0.57(0.45,0.72) | 3.86E-06 | 0(0,0.01) | 0.185641 | 0.65 | 1.77E-05 | 1 |
| never eat wheat vs. no wheat restrictions | 0.6(0.51,0.71) | 2.74E-09 | 0.56(0.5,0.64) | 1.57E-19 | 0.55(0.41,0.74) | 8.50E-05 | 0(0,0.01) | 0.725356 | 0.57 | 4.97E-06 | 1 |
| never eat sugar vs. no eggs, dairy, wheat, or sugar restrictions | 0.99(0.92,1.07) | 0.831277 | 0.98(0.89,1.08) | 0.637554 | 1.06(0.81,1.39) | 0.656125 | 0(0,0) | 0.615324 | 1 | 0.943104 | 4 |
| never eat sugar vs. no sugar restrictions | 0.98(0.91,1.06) | 0.680343 | 0.99(0.9,1.09) | 0.830812 | 1.05(0.8,1.37) | 0.724967 | 0(0,0) | 0.630858 | 1 | 0.903861 | 3 |
| milk type: dairy-based milk vs. never | 0.98(0.74,1.3) | 0.906466 | 1.01(0.76,1.33) | 0.964654 | 0.97(0.48,1.99) | 0.944318 | 0(-0.01,0.01) | 0.98444 | 0.98 | 0.86902 | 0 |
| milk type: any milk vs. never | 1.05(0.78,1.42) | 0.72995 | 1.06(0.79,1.43) | 0.6845 | 0.99(0.5,1.96) | 0.982926 | 0(-0.01,0.01) | 0.888245 | 1.04 | 0.775304 | 0 |
| milk type: full cream vs. never | 0.99(0.95,1.03) | 0.720198 | 1(0.96,1.04) | 0.955692 | 1.05(0.95,1.16) | 0.304186 | 0(-0.01,0) | 0.283643 | 1.01 | 0.505126 | 1 |
| milk type: full cream vs. any other | 0.94(0.77,1.14) | 0.515269 | 0.9(0.75,1.09) | 0.296575 | 0.62(0.25,1.54) | 0.304143 | 0(0,0.01) | 0.280597 | 0.93 | 0.376552 | 3 |
| milk type: semi-skimmed vs. never | 0.97(0.78,1.21) | 0.775536 | 0.97(0.78,1.21) | 0.818034 | 1.02(0.55,1.9) | 0.942507 | 0(-0.01,0.01) | 0.964465 | 1.01 | 0.921034 | 0 |
| milk type: semi-skimmed vs. any other | 1.07(0.95,1.2) | 0.290742 | 1.05(0.94,1.18) | 0.387495 | 0.98(0.7,1.37) | 0.892055 | 0(0,0.01) | 0.667079 | 1.02 | 0.771113 | 3 |
| milk type: skimmed vs. never | 1.02(0.93,1.1) | 0.718505 | 1.02(0.94,1.11) | 0.583083 | 1(0.84,1.2) | 0.966904 | 0(0,0.01) | 0.876114 | 1.02 | 0.65091 | 0 |
| milk type: skimmed vs. any other | 1.13(1,1.28) | 0.048659 | 1.1(0.98,1.25) | 0.105819 | 1.06(0.68,1.67) | 0.793765 | 0(-0.01,0.01) | 0.890693 | 1.1 | 0.092187 | 2 |
| milk type: soy milk vs. never | 1.02(0.99,1.05) | 0.300038 | 1.02(0.99,1.05) | 0.217792 | 1(0.93,1.07) | 0.959975 | 0(0,0.01) | 0.387059 | 1.03 | 0.053799 | 0 |
| milk type: soy milk vs. any other | 1.16(0.88,1.53) | 0.286782 | 1.18(0.9,1.55) | 0.220715 | 1.58(0.73,3.44) | 0.247542 | 0(-0.01,0) | 0.310609 | 1.19 | 0.054782 | 1 |
| milk type: other milk vs. never | 1.02(0.98,1.06) | 0.357308 | 1.02(0.98,1.05) | 0.369837 | 1(0.93,1.09) | 0.921399 | 0(0,0.01) | 0.536191 | 1.03 | 0.164617 | 0 |
| milk type: other milk vs. any other | 0.72(0.4,1.3) | 0.273118 | 0.8(0.46,1.38) | 0.420744 | 0.94(0.31,2.86) | 0.917412 | 0(-0.01,0.01) | 0.818732 | 1.06 | 0.82728 | 0 |
| spread type: all spreads vs. never | 1.04(0.91,1.19) | 0.539208 | 1.02(0.89,1.16) | 0.810658 | 1.08(0.69,1.69) | 0.738623 | 0(0,0.01) | 0.820751 | 1.05 | 0.391592 | 4 |
| spread type: butter + margarine vs. never | 1.02(0.94,1.1) | 0.664159 | 0.97(0.89,1.06) | 0.528755 | 1.26(0.93,1.7) | 0.134571 | 0(-0.01,0) | 0.215705 | 1 | 0.978884 | 4 |
| spread type: any oil based spread vs. never | 1.08(1,1.17) | 0.049176 | 1.04(0.95,1.13) | 0.417822 | 0.99(0.75,1.31) | 0.957217 | 0(0,0.01) | 0.521441 | 1.06 | 0.180046 | 1 |
| spread type: butter and butter-like spreads vs. oil-based spreads | 0.95(0.87,1.04) | 0.261422 | 0.95(0.86,1.05) | 0.302872 | 1.06(0.84,1.35) | 0.617132 | 0(-0.01,0) | 0.307005 | 0.96 | 0.37677 | 1 |
| spread type: butter and margarine spreads vs. oil-based spreads | 0.92(0.85,1) | 0.041914 | 0.95(0.87,1.04) | 0.287907 | 1.08(0.83,1.42) | 0.561393 | 0(-0.01,0) | 0.176401 | 0.93 | 0.054723 | 6 |
| spread type: butter vs. never | 1.02(0.96,1.09) | 0.567542 | 1.03(0.97,1.1) | 0.350414 | 1.2(0.98,1.47) | 0.07641 | 0(-0.01,0) | 0.15015 | 1.02 | 0.448461 | 1 |
| spread type: butter vs. any other | 0.96(0.89,1.02) | 0.205172 | 0.97(0.9,1.05) | 0.495992 | 0.96(0.74,1.25) | 0.784078 | 0(0,0) | 0.764886 | 0.95 | 0.158635 | 5 |
| spread type: tub margarine vs. never | 1.05(0.99,1.11) | 0.124789 | 1.01(0.96,1.07) | 0.650906 | 1.02(0.86,1.2) | 0.844165 | 0(0,0.01) | 0.611209 | 1.04 | 0.158286 | 1 |
| spread type: tub margarine vs. any other | 1.31(1.06,1.63) | 0.014301 | 1.15(0.93,1.42) | 0.199137 | 1.02(0.58,1.8) | 0.945004 | 0(0,0.01) | 0.390328 | 1.29 | 0.026454 | 1 |
| spread type: flora + benecol vs. never | 1.01(0.96,1.06) | 0.719061 | 1.01(0.96,1.07) | 0.637775 | 0.97(0.84,1.11) | 0.640984 | 0(0,0.01) | 0.547057 | 1 | 0.910463 | 4 |
| spread type: flora + benecol vs. any other | 0.87(0.76,1) | 0.043574 | 0.89(0.79,1) | 0.054074 | 0.83(0.65,1.05) | 0.120342 | 0(0,0.01) | 0.213946 | 0.95 | 0.336459 | 1 |
| spread type: olive oil spread vs. never | 1.08(1.01,1.15) | 0.01854 | 1.04(0.98,1.11) | 0.193635 | 0.94(0.77,1.15) | 0.540057 | 0(0,0.01) | 0.142361 | 1.04 | 0.15544 | 2 |
| spread type: olive oil spread vs. any other | 0.9(0.74,1.09) | 0.276853 | 0.98(0.81,1.19) | 0.815912 | 1.24(0.83,1.87) | 0.295198 | 0(-0.01,0) | 0.106553 | 0.97 | 0.713196 | 1 |
| spread type: other oil-based spread vs. never | 1.02(0.96,1.08) | 0.489249 | 0.98(0.93,1.04) | 0.51405 | 0.91(0.77,1.07) | 0.251789 | 0(0,0.01) | 0.081602 | 1 | 0.963872 | 3 |
| spread type: other oil-based spread vs. any other | 0.96(0.82,1.13) | 0.639455 | 0.96(0.82,1.13) | 0.647114 | 1.13(0.71,1.79) | 0.608834 | 0(-0.01,0) | 0.457131 | 0.88 | 0.093062 | 2 |
| spread type: low fat spread vs. never | 1.01(0.94,1.08) | 0.873283 | 0.99(0.92,1.05) | 0.692538 | 1.04(0.9,1.2) | 0.627647 | 0(-0.01,0) | 0.73176 | 1.01 | 0.683439 | 1 |
| spread type: low fat spread vs. any other | 1.4(1.03,1.9) | 0.031229 | 1.38(1.03,1.86) | 0.033365 | 1.44(0.74,2.82) | 0.285755 | 0(-0.01,0.01) | 0.930835 | 1.4 | 0.031078 | 1 |
| bread type: white vs. any other | 1.08(1.02,1.14) | 0.004545 | 1.07(1,1.14) | 0.060249 | 0.99(0.79,1.23) | 0.921385 | 0(0,0) | 0.304958 | 1.09 | 0.000289 | 10 |
| bread type: brown vs. any other | 0.9(0.73,1.1) | 0.303471 | 0.94(0.78,1.13) | 0.493557 | 0.79(0.22,2.87) | 0.718396 | 0.01(-0.01,0.02) | 0.478425 | 0.96 | 0.604626 | 3 |
| bread type: wholemeal/wholegrain vs. any other | 0.91(0.86,0.96) | 0.000764 | 0.93(0.87,0.99) | 0.028602 | 1.08(0.85,1.38) | 0.516008 | 0(-0.01,0) | 0.095497 | 0.88 | 1.82E-06 | 8 |
| bread type: white vs. wholemeal/wholegrain + brown | 1.07(1.01,1.12) | 0.011203 | 1.05(0.98,1.12) | 0.163038 | 1.06(0.87,1.29) | 0.569502 | 0(0,0) | 0.717819 | 1.07 | 0.001887 | 9 |
| bread type: wholemeal/wholegrain vs. white + brown | 0.9(0.86,0.95) | 3.21E-05 | 0.92(0.87,0.98) | 0.012748 | 1.07(0.88,1.3) | 0.487515 | 0(-0.01,0) | 0.072061 | 0.9 | 5.05E-06 | 7 |
| cereal type: biscuit cereal vs. any other | 1.21(1.04,1.39) | 0.01077 | 1.12(0.98,1.29) | 0.095722 | 1.31(0.84,2.04) | 0.23422 | 0(-0.01,0.01) | 0.814028 | 1.15 | 0.055756 | 2 |
| cereal type: bran cereal vs. any other | 1.02(0.87,1.19) | 0.838314 | 1.03(0.89,1.18) | 0.689926 | 0.87(0.58,1.31) | 0.516095 | 0(0,0.01) | 0.243517 | 1.05 | 0.469337 | 2 |
| cereal type: oat cereal vs. any other | 1.16(1.01,1.32) | 0.029725 | 1.16(1.02,1.31) | 0.0197 | 1.03(0.69,1.52) | 0.899341 | 0(0,0.01) | 0.590253 | 1.18 | 0.006252 | 3 |
| cereal type: muesli vs. any other | 0.88(0.81,0.94) | 0.00048 | 0.93(0.85,1.02) | 0.110903 | 0.96(0.7,1.3) | 0.774229 | 0(-0.01,0) | 0.443619 | 0.88 | 0.000526 | 6 |
| cereal type: cornflakes/frosties vs. any other | 1.04(0.96,1.14) | 0.35334 | 1.07(0.96,1.2) | 0.234599 | 0.63(0.48,0.84) | 0.001332 | 0.01(0,0.01) | 0.000683 | 1.05 | 0.279198 | 8 |
| coffee type: decaffeinated vs. any other | 0.87(0.83,0.92) | 1.07E-06 | 0.9(0.84,0.96) | 0.00139 | 0.96(0.75,1.21) | 0.720625 | 0(0,0) | 0.511864 | 0.89 | 2.25E-05 | 13 |
| coffee type: ground+instant vs. other+decaff | 0.9(0.8,1.01) | 0.067921 | 0.9(0.81,1) | 0.046038 | 0.89(0.63,1.26) | 0.52238 | 0(-0.01,0.01) | 0.826048 | 0.94 | 0.265063 | 3 |
| coffee type: ground vs. any other | 1.04(0.92,1.18) | 0.537456 | 1.08(0.96,1.21) | 0.219551 | 1.04(0.74,1.44) | 0.83147 | 0(-0.01,0.01) | 0.992595 | 1.07 | 0.242884 | 3 |
| tablespoons of cooked vegetables per day | 0.93(0.87,1) | 0.040157 | 0.93(0.85,1.01) | 0.073113 | 0.79(0.59,1.04) | 0.096211 | 0(0,0.01) | 0.171837 | 0.95 | 0.103164 | 9 |
| tablespoons of raw vegetables per day | 1.05(0.98,1.11) | 0.153841 | 1.08(1,1.16) | 0.060416 | 1.16(0.88,1.54) | 0.28674 | 0(-0.01,0) | 0.278517 | 1 | 0.955788 | 13 |
| pieces of fresh fruit per day | 0.92(0.88,0.96) | 0.0002 | 0.95(0.9,1) | 0.04092 | 0.95(0.81,1.12) | 0.530061 | 0(0,0) | 0.56734 | 0.91 | 5.39E-06 | 12 |
| pieces of dried fruit per day | 0.95(0.89,1.01) | 0.112924 | 0.94(0.87,1.02) | 0.129528 | 1.01(0.78,1.3) | 0.960782 | 0(0,0) | 0.51787 | 0.94 | 0.034666 | 9 |
| slices of bread per week | 1.08(1.01,1.15) | 0.017948 | 1.04(0.97,1.12) | 0.272402 | 1.13(0.89,1.43) | 0.302271 | 0(0,0) | 0.557078 | 1.04 | 0.192728 | 10 |
| bowls of cereal per week | 0.94(0.89,0.99) | 0.029418 | 0.96(0.89,1.04) | 0.312944 | 0.81(0.65,1.01) | 0.05714 | 0(0,0.01) | 0.121686 | 0.95 | 0.037764 | 13 |
| cups of tea per day | 0.96(0.91,1.02) | 0.17107 | 0.98(0.92,1.03) | 0.375944 | 0.99(0.85,1.15) | 0.89022 | 0(0,0) | 0.496143 | 1.02 | 0.39225 | 11 |
| cups of coffee per day | 0.95(0.88,1.01) | 0.12148 | 0.98(0.94,1.03) | 0.457848 | 0.99(0.89,1.11) | 0.85055 | 0(0,0) | 0.295309 | 0.93 | 0.004464 | 7 |
| glasses of water per day | 0.94(0.9,0.99) | 0.010117 | 0.93(0.88,0.98) | 0.008366 | 0.9(0.78,1.04) | 0.140544 | 0(0,0) | 0.331223 | 0.94 | 0.000641 | 14 |
| champagne/white wine glasses per month | 0.96(0.89,1.05) | 0.376377 | 1(0.93,1.08) | 0.971669 | 0.92(0.7,1.2) | 0.541666 | 0(0,0) | 0.961331 | 0.95 | 0.135116 | 6 |
| red wine glasses per month | 0.94(0.89,1) | 0.041626 | 0.94(0.89,0.99) | 0.032049 | 0.97(0.83,1.13) | 0.677002 | 0(0,0) | 0.241783 | 0.89 | 2.33E-06 | 7 |
| beer/cider glasses per month | 0.97(0.9,1.05) | 0.465614 | 0.95(0.9,1.01) | 0.111643 | 0.96(0.81,1.14) | 0.620738 | 0(0,0) | 0.566089 | 0.96 | 0.176437 | 10 |
| spirits measures per month | 1.03(0.94,1.14) | 0.495512 | 1.04(0.94,1.14) | 0.459273 | 1.09(0.83,1.42) | 0.526673 | 0(-0.01,0) | 0.478435 | 1.05 | 0.200027 | 2 |
| fortwine glasses per month | 0.88(0.73,1.06) | 0.174177 | 0.84(0.7,1) | 0.048015 | 0.84(0.46,1.53) | 0.569074 | 0(-0.01,0.01) | 0.632586 | 0.9 | 0.223886 | 1 |
| other alcohol glasses per month | 0.97(0.84,1.13) | 0.718796 | 0.99(0.87,1.14) | 0.942758 | 0.93(0.6,1.42) | 0.724326 | 0(-0.01,0.01) | 0.673026 | 1.07 | 0.268833 | 1 |
| total drinks of alcohol per month | 0.98(0.93,1.03) | 0.435071 | 0.96(0.92,1) | 0.034408 | 0.95(0.86,1.05) | 0.337241 | 0(0,0) | 0.780309 | 0.94 | 0.000366 | 24 |
| overall oily fish intake | 1(0.95,1.05) | 0.976128 | 1.03(0.97,1.11) | 0.326378 | 1.23(0.99,1.54) | 0.066671 | 0(-0.01,0) | 0.039467 | 0.99 | 0.695045 | 14 |
| overall non-oily fish intake | 1(0.92,1.08) | 0.960943 | 0.98(0.89,1.07) | 0.620803 | 1.1(0.83,1.47) | 0.504705 | 0(0,0) | 0.652804 | 1.01 | 0.768312 | 6 |
| overall processed meat intake | 1.02(0.95,1.09) | 0.597737 | 1(0.92,1.09) | 0.90847 | 0.98(0.73,1.32) | 0.894474 | 0(0,0) | 0.76019 | 1.05 | 0.155251 | 9 |
| overall poultry intake | 1.06(0.96,1.17) | 0.252989 | 1.03(0.93,1.14) | 0.626269 | 1.25(0.89,1.76) | 0.199088 | 0(-0.01,0) | 0.181758 | 1.04 | 0.413891 | 3 |
| overall beef intake | 0.92(0.85,0.99) | 0.020984 | 0.96(0.88,1.04) | 0.352928 | 1.05(0.82,1.35) | 0.681691 | 0(-0.01,0) | 0.356048 | 0.95 | 0.128258 | 8 |
| overall lamb/mutton intake | 0.89(0.83,0.96) | 0.001033 | 0.88(0.81,0.96) | 0.003271 | 1.02(0.78,1.34) | 0.877976 | 0(-0.01,0) | 0.416852 | 0.91 | 0.003178 | 8 |
| overall pork intake | 0.94(0.87,1.03) | 0.172587 | 0.94(0.85,1.03) | 0.164424 | 0.93(0.73,1.18) | 0.544008 | 0(0,0) | 0.794368 | 0.9 | 0.005997 | 4 |
| overall cheese intake | 0.93(0.89,0.98) | 0.006824 | 0.93(0.88,0.99) | 0.023758 | 0.94(0.79,1.11) | 0.446665 | 0(0,0) | 0.929984 | 0.91 | 6.39E-05 | 11 |
| frequency of adding salt to food | 1.08(1.03,1.13) | 0.00187 | 1.06(1,1.11) | 0.05167 | 1.05(0.9,1.23) | 0.553715 | 0(0,0) | 0.682999 | 1.06 | 0.001786 | 7 |
| temperature of hot drinks | 1.06(1,1.11) | 0.043485 | 1.06(0.99,1.14) | 0.085981 | 0.87(0.7,1.08) | 0.205084 | 0(0,0.01) | 0.107154 | 1.07 | 0.00561 | 14 |
| overall alcohol intake | 0.98(0.94,1.03) | 0.459123 | 0.96(0.93,1) | 0.069989 | 0.91(0.82,1) | 0.058794 | 0(0,0) | 0.37798 | 0.94 | 0.000283 | 14 |
| among current drinkers, drinks usually with meals: yes, it varies, no | 0.9(0.84,0.95) | 0.000313 | 0.97(0.9,1.04) | 0.378186 | 1.05(0.82,1.34) | 0.698133 | 0(-0.01,0) | 0.125676 | 0.9 | 0.000154 | 7 |
| milk type: skimmed, semi-skimmed, full cream (QT) | 0.94(0.86,1.03) | 0.20148 | 0.93(0.85,1.01) | 0.100136 | 0.91(0.61,1.37) | 0.655268 | 0(-0.01,0.01) | 0.870819 | 0.94 | 0.158035 | 6 |

MR: mendelian randomization; MRPRESSO: MR pleiotropy residual sum and outlier; OR: odd ratio; CI: confidence interval; No.: number.

**Table S23. Leave-one-out analysis of association between genetically predicted dietary habits and neuroticism.**

| **Exposure** | **IVW Estimate**  **[Min, Max] ^a^** | **P value [Min, Max] ^b^** |
| --- | --- | --- |
| alcohol drinker status: current + former vs. never | [0.96,1.03] | [0.73672536512531,0.990642669114647] |
| alcohol drinker status: current vs. never | [0.97,1.03] | [0.762340469781723,0.998424161866281] |
| among current drinkers, drinks usually with meals: yes + it varies vs. no | [0.84,0.87] | [8.22770521186827e-06,0.000195653728638242] |
| among current drinkers, drinks usually with meals: yes vs. no | [0.87,0.89] | [2.59853764815572e-09,1.06587047590244e-07] |
| never eat eggs vs. no eggs, dairy, wheat, or sugar restrictions | [1.04,1.33] | [0.0476389195085619,0.839842205914492] |
| never eat eggs vs. no eggs restrictions | [1.17,1.52] | [0.0601841779921536,0.468470871335497] |
| never eat dairy vs. no eggs, dairy, wheat, or sugar restrictions | [0.96,1.17] | [0.361804522747033,0.776983180957852] |
| never eat dairy vs. no dairy restrictions | [0.95,1.23] | [0.343980438023421,0.748075962888127] |
| never eat wheat vs. no eggs, dairy, wheat, or sugar restrictions | [0.63,0.67] | [8.0624304529931e-11,4.31192097971055e-07] |
| never eat wheat vs. no wheat restrictions | [0.55,0.59] | [9.24909915106024e-13,1.6672658419852e-08] |
| never eat sugar vs. no eggs, dairy, wheat, or sugar restrictions | [0.99,1] | [0.730083098147129,0.999620165867111] |
| never eat sugar vs. no sugar restrictions | [0.98,0.99] | [0.53702552559931,0.891961681858148] |
| milk type: dairy-based milk vs. never | [0.93,1.05] | [0.533973491317649,0.992269203416373] |
| milk type: any milk vs. never | [0.99,1.13] | [0.286063292039592,0.994194608582123] |
| milk type: full cream vs. never | [0.99,1.01] | [0.497348146542365,0.981169130522685] |
| milk type: full cream vs. any other | [0.93,1.06] | [0.527480476930404,0.997695960690855] |
| milk type: semi-skimmed vs. never | [0.97,1.06] | [0.442535035459201,0.934511165696871] |
| milk type: semi-skimmed vs. any other | [1.02,1.07] | [0.280385384752003,0.744332879039651] |
| milk type: skimmed vs. never | [1,1.03] | [0.354194789595821,0.9289165404236] |
| milk type: skimmed vs. any other | [1,1.07] | [0.27463126605428,0.955336684118619] |
| milk type: soy milk vs. never | [1.02,1.03] | [0.00838108934724058,0.109715413147919] |
| milk type: soy milk vs. any other | [1.02,1.19] | [0.0851978213524892,0.861993266305785] |
| milk type: other milk vs. never | [1.02,1.03] | [0.0664880500058888,0.29607114341126] |
| milk type: other milk vs. any other | [0.92,1.18] | [0.538158886520282,0.981736744427243] |
| spread type: all spreads vs. never | [1.09,1.16] | [0.0344875691473673,0.245680932366599] |
| spread type: butter + margarine vs. never | [1.02,1.07] | [0.156381870067136,0.637444544404595] |
| spread type: any oil based spread vs. never | [1.06,1.1] | [0.030821161870502,0.169519445436725] |
| spread type: butter and butter-like spreads vs. oil-based spreads | [0.93,0.96] | [0.0862344458983466,0.37065427987864] |
| spread type: butter and margarine spreads vs. oil-based spreads | [0.89,0.93] | [0.00946852881026191,0.0718171664686421] |
| spread type: butter vs. never | [1.02,1.05] | [0.102218189945225,0.444720399927377] |
| spread type: butter vs. any other | [0.92,0.94] | [0.0239705963554125,0.0743615625767579] |
| spread type: tub margarine vs. never | [1.04,1.07] | [0.0221451643680426,0.144764826133773] |
| spread type: tub margarine vs. any other | [1.23,1.33] | [0.0063045201932984,0.0393224833484656] |
| spread type: flora + benecol vs. never | [0.99,1.02] | [0.424430532668168,0.992020518219229] |
| spread type: flora + benecol vs. any other | [0.93,1] | [0.194497108428286,0.971990011456977] |
| spread type: olive oil spread vs. never | [1.06,1.1] | [0.00579826083249179,0.0618667626209944] |
| spread type: olive oil spread vs. any other | [0.87,0.97] | [0.116440169941107,0.707574108869508] |
| spread type: other oil-based spread vs. never | [1.02,1.05] | [0.136278823549111,0.419692862177534] |
| spread type: other oil-based spread vs. any other | [0.92,0.99] | [0.289511781122322,0.895959025340906] |
| spread type: low fat spread vs. never | [0.99,1.03] | [0.419859960583157,0.919957331709392] |
| spread type: low fat spread vs. any other | [1.32,1.51] | [0.000869231518515818,0.0463712402793276] |
| bread type: white vs. any other | [1.09,1.11] | [0.000330572553738386,0.00219454525263769] |
| bread type: brown vs. any other | [1,1.32] | [0.255411312975955,0.975726179953465] |
| bread type: wholemeal/wholegrain vs. any other | [0.88,0.9] | [2.645904722113e-05,0.000562493816690064] |
| bread type: white vs. wholemeal/wholegrain + brown | [1.09,1.11] | [0.000213137169151813,0.00156043135777576] |
| bread type: wholemeal/wholegrain vs. white + brown | [0.89,0.91] | [2.47698088939948e-05,0.000545906405218665] |
| cereal type: biscuit cereal vs. any other | [1.19,1.28] | [0.00228079555765504,0.0210384749277287] |
| cereal type: bran cereal vs. any other | [1.02,1.12] | [0.220029562447167,0.802462779342046] |
| cereal type: oat cereal vs. any other | [1.1,1.17] | [0.0197761735269033,0.191352922917302] |
| cereal type: muesli vs. any other | [0.84,0.87] | [7.40292529508726e-05,0.000553969229924302] |
| cereal type: cornflakes/frosties vs. any other | [1.05,1.08] | [0.073455236564459,0.256386787522681] |
| coffee type: decaffeinated vs. any other | [0.88,0.9] | [3.65293587978425e-05,0.000264070483636435] |
| coffee type: ground+instant vs. other+decaff | [0.9,0.95] | [0.0826543439859701,0.472051700736712] |
| coffee type: ground vs. any other | [1,1.07] | [0.28361373130116,0.947566510924961] |
| tablespoons of cooked vegetables per day | [0.94,0.96] | [0.11360624075522,0.33676481798846] |
| tablespoons of raw vegetables per day | [0.99,1.02] | [0.560414124001654,0.995831103621374] |
| pieces of fresh fruit per day | [0.9,0.91] | [1.60192741681761e-05,0.000128225201905591] |
| pieces of dried fruit per day | [0.92,0.94] | [0.0176449568013441,0.0758485334757055] |
| slices of bread per week | [1.04,1.07] | [0.0612116391882566,0.228191104868652] |
| bowls of cereal per week | [0.95,0.96] | [0.0631251899718337,0.193775880719925] |
| cups of tea per day | [1.03,1.05] | [0.094500701267203,0.377992225702014] |
| cups of coffee per day | [0.91,0.95] | [0.0010947537959107,0.0638567402125905] |
| glasses of water per day | [0.95,0.97] | [0.0317660320214674,0.199187650554096] |
| champagne/white wine glasses per month | [0.91,0.97] | [0.0432863663475949,0.493250289201954] |
| red wine glasses per month | [0.87,0.9] | [4.66514046369365e-06,0.000196510090869135] |
| beer/cider glasses per month | [0.99,1.01] | [0.717607848587485,0.999238646913947] |
| spirits measures per month | [0.98,1.02] | [0.587138065389742,0.994189942221322] |
| fortwine glasses per month | [0.9,1] | [0.187394214119307,0.965136626142056] |
| other alcohol glasses per month | [0.97,1.07] | [0.229777958490179,0.976163595494321] |
| total drinks of alcohol per month | [0.93,0.95] | [0.00387492377056997,0.0318349062376883] |
| overall oily fish intake | [0.97,0.99] | [0.253612983091951,0.636880902231467] |
| overall non-oily fish intake | [1.02,1.05] | [0.251182287539381,0.598518859484507] |
| overall processed meat intake | [1.01,1.04] | [0.3700394492419,0.836745953695322] |
| overall poultry intake | [0.99,1.03] | [0.485302910400916,0.999232058662615] |
| overall beef intake | [0.93,0.95] | [0.0576514733106381,0.231500161928345] |
| overall lamb/mutton intake | [0.91,0.93] | [0.00855951247401299,0.0615599369302767] |
| overall pork intake | [0.89,0.91] | [0.00297488300263436,0.0236150888505265] |
| overall cheese intake | [0.92,0.93] | [0.00124651176520073,0.00589970728357677] |
| frequency of adding salt to food | [1.07,1.09] | [0.000253675558046253,0.0012443019030246] |
| temperature of hot drinks | [1.02,1.04] | [0.166971095569062,0.430512208959812] |
| overall alcohol intake | [0.94,0.95] | [0.00350846097787266,0.022663457195698] |
| among current drinkers, drinks usually with meals: yes, it varies, no | [0.86,0.89] | [4.40682670046944e-06,9.00069305723081e-05] |
| milk type: skimmed, semi-skimmed, full cream (QT) | [0.92,0.96] | [0.174172366309932,0.540278017985262] |

**^a^** the minimum value and maximum value of inverse variance weighted estimate;

**^b^** the minimum value and maximum value of P value;

IVW: inverse variance weighted.

**Table S24. Associations between genetically predicted dietary habits and difficulty awakening in sensitivity analysis.**

| **Exposure** | **Weighted median** | | **Mode-based** | | **MR-Egger** | | | | **MRPRESSO** | | |
| --- | --- | --- | --- | --- | --- | --- | --- | --- | --- | --- | --- |
|  | **OR (95% CI)** | **P value** | **OR (95% CI)** | **P value** | **OR (95% CI)** | **P value** | **Intercept** | **P value** | **OR** | **P value** | **No. of outliers** |
| alcohol drinker status: current + former vs. never | 0.94(0.81,1.1) | 0.441946 | 0.96(0.82,1.12) | 0.595212 | 0.81(0.61,1.09) | 0.1649 | 0(0,0) | 0.323656 | 0.9 | 0.125323 | 1 |
| alcohol drinker status: current vs. never | 1.01(0.87,1.17) | 0.903991 | 1(0.85,1.16) | 0.962876 | 0.88(0.66,1.19) | 0.410578 | 0(0,0) | 0.615568 | 0.94 | 0.333526 | 2 |
| among current drinkers, drinks usually with meals: yes + it varies vs. no | 1.05(1,1.1) | 0.029715 | 1.05(1,1.11) | 0.06868 | 1.04(0.87,1.23) | 0.691765 | 0(0,0) | 0.901986 | 1.07 | 0.001498 | 5 |
| among current drinkers, drinks usually with meals: yes vs. no | 1.03(1,1.06) | 0.023519 | 1.02(0.98,1.06) | 0.282833 | 0.99(0.9,1.09) | 0.83496 | 0(0,0) | 0.556753 | 1.03 | 0.013484 | 5 |
| never eat eggs vs. no eggs, dairy, wheat, or sugar restrictions | 1.18(0.91,1.52) | 0.203043 | 1.22(0.95,1.56) | 0.117572 | 1.12(0.58,2.17) | 0.727382 | 0(-0.01,0.01) | 0.974475 | 1.23 | 0.065761 | 1 |
| never eat eggs vs. no eggs restrictions | 1.24(0.89,1.74) | 0.204722 | 1.24(0.89,1.73) | 0.197525 | 1.15(0.58,2.29) | 0.6922 | 0(0,0) | 0.807185 | 1.24 | 0.060465 | 0 |
| never eat dairy vs. no eggs, dairy, wheat, or sugar restrictions | 1.17(0.92,1.49) | 0.205483 | 1.09(0.86,1.38) | 0.484242 | 1.23(0.59,2.55) | 0.582472 | 0(-0.01,0) | 0.816056 | 1.11 | 0.31437 | 2 |
| never eat dairy vs. no dairy restrictions | 1.11(0.82,1.49) | 0.505758 | 1.08(0.81,1.45) | 0.60142 | 1.3(0.53,3.2) | 0.566559 | 0(-0.01,0) | 0.706573 | 1.08 | 0.551186 | 2 |
| never eat wheat vs. no eggs, dairy, wheat, or sugar restrictions | 1.28(1.19,1.38) | 2.98E-10 | 1.27(1.2,1.35) | 6.39E-15 | 1.36(1.23,1.51) | 5.02E-09 | 0(0,0) | 0.010105 | 1.22 | 8.22E-06 | 0 |
| never eat wheat vs. no wheat restrictions | 1.35(1.23,1.48) | 4.15E-10 | 1.34(1.24,1.44) | 6.04E-15 | 1.42(1.25,1.61) | 9.67E-08 | 0(0,0) | 0.053901 | 1.28 | 1.50E-06 | 0 |
| never eat sugar vs. no eggs, dairy, wheat, or sugar restrictions | 1.13(1.08,1.18) | 2.70E-07 | 1.14(1.08,1.2) | 7.22E-06 | 1.19(0.99,1.42) | 0.061571 | 0(0,0) | 0.413638 | 1.09 | 4.25E-05 | 9 |
| never eat sugar vs. no sugar restrictions | 1.12(1.07,1.17) | 4.44E-06 | 1.12(1.05,1.19) | 0.000227 | 1.05(0.87,1.27) | 0.638316 | 0(0,0) | 0.632705 | 1.09 | 0.000196 | 8 |
| milk type: dairy-based milk vs. never | 1.03(0.84,1.26) | 0.797615 | 1.05(0.86,1.27) | 0.644298 | 0.59(0.31,1.09) | 0.093298 | 0(0,0.01) | 0.057133 | 0.93 | 0.372823 | 2 |
| milk type: any milk vs. never | 1.06(0.87,1.31) | 0.555084 | 1.12(0.9,1.39) | 0.298616 | 0.87(0.57,1.34) | 0.5355 | 0(0,0) | 0.553178 | 0.98 | 0.850929 | 0 |
| milk type: full cream vs. never | 1.02(0.99,1.05) | 0.1831 | 1.03(1,1.06) | 0.059932 | 0.99(0.9,1.08) | 0.800721 | 0(0,0.01) | 0.596545 | 1.03 | 0.027007 | 2 |
| milk type: full cream vs. any other | 0.98(0.85,1.12) | 0.765785 | 0.97(0.84,1.13) | 0.728091 | 0.55(0.33,0.92) | 0.021303 | 0.01(0,0.01) | 0.024033 | 0.98 | 0.774355 | 4 |
| milk type: semi-skimmed vs. never | 1(0.86,1.15) | 0.971089 | 1.04(0.9,1.2) | 0.59025 | 0.91(0.65,1.28) | 0.579229 | 0(0,0) | 0.83718 | 0.94 | 0.280413 | 0 |
| milk type: semi-skimmed vs. any other | 0.97(0.9,1.06) | 0.523406 | 0.96(0.88,1.04) | 0.318251 | 0.94(0.78,1.14) | 0.547862 | 0(0,0) | 0.728323 | 0.97 | 0.42622 | 1 |
| milk type: skimmed vs. never | 1.08(1.02,1.14) | 0.006849 | 1.07(1.02,1.14) | 0.011256 | 1.09(0.96,1.24) | 0.188644 | 0(0,0) | 0.537758 | 1.05 | 0.030286 | 2 |
| milk type: skimmed vs. any other | 1.06(0.98,1.15) | 0.143024 | 1.05(0.96,1.15) | 0.279871 | 1.31(1,1.73) | 0.052063 | 0(-0.01,0) | 0.103268 | 1.02 | 0.62866 | 3 |
| milk type: soy milk vs. never | 0.97(0.95,0.99) | 0.015177 | 0.98(0.95,1) | 0.043726 | 0.99(0.94,1.04) | 0.76391 | 0(0,0) | 0.533155 | 0.98 | 0.01789 | 0 |
| milk type: soy milk vs. any other | 0.91(0.75,1.11) | 0.36953 | 0.88(0.73,1.07) | 0.203951 | 1(0.51,1.96) | 0.994818 | 0(-0.01,0) | 0.670933 | 0.88 | 0.1277 | 3 |
| milk type: other milk vs. never | 0.96(0.94,0.99) | 0.00361 | 0.97(0.95,1) | 0.043456 | 0.97(0.92,1.01) | 0.153482 | 0(0,0) | 0.712902 | 0.97 | 0.026756 | 0 |
| milk type: other milk vs. any other | 0.93(0.64,1.34) | 0.68096 | 0.89(0.6,1.3) | 0.530204 | 0.71(0.39,1.29) | 0.255972 | 0(0,0.01) | 0.437 | 0.87 | 0.315844 | 0 |
| spread type: all spreads vs. never | 0.98(0.9,1.08) | 0.730901 | 1(0.91,1.1) | 0.963079 | 0.98(0.75,1.29) | 0.912283 | 0(0,0) | 0.701492 | 0.99 | 0.687453 | 3 |
| spread type: butter + margarine vs. never | 1(0.94,1.06) | 0.983308 | 1.01(0.95,1.07) | 0.853882 | 1.02(0.83,1.26) | 0.837638 | 0(0,0) | 0.690655 | 1.01 | 0.772439 | 4 |
| spread type: any oil based spread vs. never | 0.97(0.92,1.02) | 0.213325 | 0.97(0.92,1.03) | 0.341321 | 1.04(0.92,1.18) | 0.517232 | 0(0,0) | 0.100401 | 0.94 | 0.00863 | 0 |
| spread type: butter and butter-like spreads vs. oil-based spreads | 1.11(1.04,1.18) | 0.001567 | 1.09(1.02,1.17) | 0.014226 | 0.98(0.83,1.16) | 0.835285 | 0(0,0) | 0.295328 | 1.07 | 0.017142 | 2 |
| spread type: butter and margarine spreads vs. oil-based spreads | 1.09(1.03,1.15) | 0.001422 | 1.08(1.02,1.15) | 0.014391 | 1.01(0.87,1.18) | 0.861505 | 0(0,0) | 0.663471 | 1.07 | 0.008526 | 4 |
| spread type: butter vs. never | 1(0.95,1.04) | 0.838037 | 0.99(0.94,1.05) | 0.81988 | 0.89(0.78,1.01) | 0.061854 | 0(0,0.01) | 0.041441 | 1.01 | 0.576148 | 2 |
| spread type: butter vs. any other | 1.02(0.97,1.07) | 0.432905 | 1.02(0.97,1.08) | 0.412368 | 0.99(0.84,1.15) | 0.850337 | 0(0,0) | 0.900442 | 1 | 0.996714 | 4 |
| spread type: tub margarine vs. never | 0.99(0.96,1.02) | 0.521321 | 1(0.97,1.04) | 0.972872 | 0.98(0.91,1.05) | 0.559529 | 0(0,0) | 0.858769 | 0.98 | 0.250659 | 0 |
| spread type: tub margarine vs. any other | 1(0.87,1.15) | 0.981167 | 0.94(0.81,1.09) | 0.392325 | 0.87(0.68,1.11) | 0.253592 | 0(0,0) | 0.317905 | 0.97 | 0.575989 | 0 |
| spread type: flora + benecol vs. never | 1(0.96,1.03) | 0.954966 | 1.01(0.97,1.05) | 0.736463 | 1.04(0.96,1.14) | 0.344819 | 0(-0.01,0) | 0.316353 | 1 | 0.99806 | 2 |
| spread type: flora + benecol vs. any other | 1.04(0.95,1.15) | 0.399689 | 1.07(0.98,1.17) | 0.132568 | 1.2(1.01,1.41) | 0.032774 | 0(0,0) | 0.071816 | 1.06 | 0.10147 | 1 |
| spread type: olive oil spread vs. never | 1.04(1,1.08) | 0.083485 | 1.02(0.98,1.07) | 0.284108 | 1.09(1,1.2) | 0.061033 | 0(-0.01,0) | 0.134862 | 1.02 | 0.187098 | 0 |
| spread type: olive oil spread vs. any other | 0.98(0.85,1.12) | 0.742546 | 0.99(0.86,1.14) | 0.888169 | 1.06(0.83,1.34) | 0.649998 | 0(0,0) | 0.634774 | 1 | 0.956703 | 0 |
| spread type: other oil-based spread vs. never | 0.97(0.94,1.01) | 0.181356 | 0.98(0.94,1.02) | 0.385972 | 0.98(0.9,1.07) | 0.624609 | 0(0,0) | 0.933285 | 0.97 | 0.030082 | 1 |
| spread type: other oil-based spread vs. any other | 1.07(0.96,1.19) | 0.241452 | 1(0.89,1.12) | 0.985534 | 0.9(0.71,1.15) | 0.395049 | 0(0,0.01) | 0.122796 | 1.07 | 0.143509 | 1 |
| spread type: low fat spread vs. never | 1(0.95,1.05) | 0.966461 | 0.99(0.94,1.03) | 0.55841 | 0.93(0.84,1.03) | 0.185442 | 0(0,0.01) | 0.233497 | 0.99 | 0.393534 | 2 |
| spread type: low fat spread vs. any other | 1.11(0.9,1.36) | 0.333207 | 1.07(0.88,1.3) | 0.494568 | 0.92(0.59,1.44) | 0.714739 | 0(0,0.01) | 0.096701 | 1.17 | 0.088285 | 1 |
| bread type: white vs. any other | 0.99(0.95,1.03) | 0.609319 | 1(0.96,1.05) | 0.843066 | 0.92(0.79,1.07) | 0.272192 | 0(0,0) | 0.488874 | 0.98 | 0.199597 | 8 |
| bread type: brown vs. any other | 1.01(0.89,1.15) | 0.869411 | 1.04(0.92,1.19) | 0.513577 | 1.02(0.66,1.6) | 0.919531 | 0(-0.01,0.01) | 0.969983 | 1.05 | 0.378786 | 3 |
| bread type: wholemeal/wholegrain vs. any other | 0.99(0.96,1.03) | 0.563231 | 0.99(0.95,1.04) | 0.754185 | 1.09(0.95,1.25) | 0.237681 | 0(0,0) | 0.197586 | 0.98 | 0.223174 | 8 |
| bread type: white vs. wholemeal/wholegrain + brown | 0.99(0.95,1.02) | 0.521611 | 1(0.96,1.05) | 0.929661 | 0.9(0.79,1.03) | 0.133413 | 0(0,0) | 0.223783 | 0.99 | 0.405309 | 6 |
| bread type: wholemeal/wholegrain vs. white + brown | 0.99(0.96,1.02) | 0.529432 | 0.99(0.95,1.03) | 0.523108 | 1.05(0.93,1.19) | 0.415428 | 0(0,0) | 0.361659 | 0.99 | 0.382809 | 9 |
| cereal type: biscuit cereal vs. any other | 0.88(0.8,0.97) | 0.006996 | 0.92(0.83,1.01) | 0.085191 | 0.93(0.75,1.15) | 0.481654 | 0(0,0) | 0.739051 | 0.9 | 0.007136 | 1 |
| cereal type: bran cereal vs. any other | 1.04(0.93,1.16) | 0.463979 | 1.07(0.96,1.19) | 0.244745 | 1.07(0.78,1.46) | 0.690405 | 0(-0.01,0) | 0.699459 | 1.09 | 0.037722 | 2 |
| cereal type: oat cereal vs. any other | 1.03(0.95,1.11) | 0.494222 | 1.01(0.93,1.1) | 0.807357 | 1.11(0.93,1.34) | 0.256677 | 0(0,0) | 0.765303 | 1.04 | 0.185839 | 1 |
| cereal type: muesli vs. any other | 0.96(0.92,1.01) | 0.123256 | 0.96(0.9,1.01) | 0.10526 | 0.83(0.71,0.97) | 0.020434 | 0(0,0) | 0.032537 | 0.96 | 0.060737 | 3 |
| cereal type: cornflakes/frosties vs. any other | 1.04(0.98,1.09) | 0.16714 | 1.04(0.98,1.11) | 0.178738 | 1.25(1.09,1.43) | 0.001828 | 0(-0.01,0) | 0.010258 | 1.06 | 0.004697 | 3 |
| coffee type: decaffeinated vs. any other | 0.98(0.95,1.02) | 0.38238 | 0.98(0.93,1.03) | 0.340221 | 0.88(0.77,0.99) | 0.038705 | 0(0,0) | 0.063541 | 0.99 | 0.512106 | 5 |
| coffee type: ground+instant vs. other+decaff | 1.02(0.95,1.1) | 0.559216 | 1.05(0.97,1.14) | 0.25682 | 0.92(0.79,1.09) | 0.338869 | 0(0,0) | 0.372599 | 1 | 0.975006 | 1 |
| coffee type: ground vs. any other | 0.97(0.89,1.05) | 0.391309 | 0.94(0.85,1.03) | 0.173509 | 1.07(0.91,1.26) | 0.439886 | 0(0,0) | 0.622951 | 1 | 0.927147 | 1 |
| tablespoons of cooked vegetables per day | 1.06(1.01,1.11) | 0.009934 | 1.05(0.99,1.11) | 0.09465 | 0.99(0.83,1.18) | 0.919592 | 0(0,0) | 0.492437 | 1.04 | 0.020705 | 7 |
| tablespoons of raw vegetables per day | 1.01(0.97,1.05) | 0.80207 | 1.02(0.97,1.07) | 0.470385 | 0.85(0.72,1) | 0.056584 | 0(0,0) | 0.039829 | 1.02 | 0.410514 | 13 |
| pieces of fresh fruit per day | 1.12(1.09,1.15) | 9.70E-14 | 1.11(1.06,1.15) | 1.71E-07 | 1.05(0.95,1.15) | 0.369425 | 0(0,0) | 0.140372 | 1.11 | 1.97E-16 | 10 |
| pieces of dried fruit per day | 1(0.96,1.04) | 0.938422 | 0.99(0.94,1.05) | 0.848085 | 1.07(0.93,1.24) | 0.356563 | 0(0,0) | 0.52767 | 1.01 | 0.543567 | 3 |
| slices of bread per week | 0.95(0.91,0.99) | 0.010669 | 0.97(0.93,1.02) | 0.214137 | 0.93(0.81,1.07) | 0.295758 | 0(0,0) | 0.601048 | 0.96 | 0.038765 | 7 |
| bowls of cereal per week | 1.06(1.02,1.1) | 0.003085 | 1.05(1,1.1) | 0.045185 | 1.14(0.99,1.31) | 0.066064 | 0(0,0) | 0.240578 | 1.04 | 0.012061 | 7 |
| cups of tea per day | 0.94(0.9,0.98) | 0.001926 | 0.92(0.89,0.96) | 0.00019 | 0.87(0.8,0.96) | 0.003426 | 0(0,0) | 0.158313 | 0.95 | 0.000537 | 7 |
| cups of coffee per day | 0.97(0.92,1.02) | 0.220756 | 0.97(0.94,1) | 0.040968 | 0.93(0.86,1) | 0.064149 | 0(0,0) | 0.013275 | 1.01 | 0.760658 | 9 |
| glasses of water per day | 1.05(1.01,1.09) | 0.011008 | 1.05(1.01,1.1) | 0.013738 | 1.13(1.02,1.25) | 0.019779 | 0(0,0) | 0.164904 | 1.04 | 0.004217 | 11 |
| champagne/white wine glasses per month | 0.96(0.91,1.01) | 0.144992 | 0.98(0.93,1.04) | 0.50342 | 0.91(0.79,1.05) | 0.21043 | 0(0,0) | 0.992814 | 0.94 | 0.009656 | 3 |
| red wine glasses per month | 0.99(0.95,1.03) | 0.567904 | 0.99(0.95,1.04) | 0.703684 | 0.99(0.88,1.12) | 0.933347 | 0(0,0) | 0.467505 | 0.98 | 0.190967 | 7 |
| beer/cider glasses per month | 0.98(0.93,1.02) | 0.31502 | 0.98(0.93,1.03) | 0.461406 | 0.88(0.79,0.99) | 0.032844 | 0(0,0) | 0.075619 | 0.99 | 0.450604 | 9 |
| spirits measures per month | 0.97(0.9,1.05) | 0.469661 | 0.97(0.89,1.05) | 0.401796 | 1.05(0.89,1.25) | 0.542039 | 0(0,0) | 0.198815 | 0.94 | 0.076272 | 1 |
| fortwine glasses per month | 1.07(0.95,1.2) | 0.265277 | 1.1(0.98,1.24) | 0.100722 | 1.02(0.76,1.37) | 0.912091 | 0(0,0) | 0.930319 | 1 | 0.935726 | 1 |
| other alcohol glasses per month | 0.99(0.91,1.07) | 0.816463 | 0.99(0.91,1.08) | 0.847198 | 1.07(0.9,1.27) | 0.464867 | 0(-0.01,0) | 0.477804 | 1.01 | 0.757863 | 0 |
| total drinks of alcohol per month | 1(0.97,1.03) | 0.844764 | 0.97(0.94,1.01) | 0.101166 | 0.95(0.9,1.01) | 0.089037 | 0(0,0) | 0.674835 | 0.98 | 0.062895 | 6 |
| overall oily fish intake | 0.98(0.95,1.02) | 0.385477 | 0.96(0.92,1) | 0.078983 | 0.94(0.82,1.08) | 0.381557 | 0(0,0) | 0.371994 | 1 | 0.804104 | 7 |
| overall non-oily fish intake | 1.03(0.97,1.09) | 0.349118 | 1.02(0.96,1.09) | 0.560374 | 0.95(0.78,1.17) | 0.650726 | 0(0,0) | 0.400913 | 1.01 | 0.787049 | 7 |
| overall processed meat intake | 0.94(0.89,0.98) | 0.005853 | 0.95(0.9,1.01) | 0.108659 | 0.95(0.81,1.12) | 0.529939 | 0(0,0) | 0.631039 | 0.94 | 0.001638 | 6 |
| overall poultry intake | 1.01(0.95,1.07) | 0.825881 | 0.99(0.92,1.06) | 0.676002 | 0.98(0.77,1.25) | 0.865327 | 0(0,0) | 0.711111 | 1.01 | 0.568741 | 5 |
| overall beef intake | 1(0.95,1.05) | 0.868303 | 0.99(0.93,1.06) | 0.786453 | 0.97(0.82,1.15) | 0.742544 | 0(0,0) | 0.977628 | 1.02 | 0.376137 | 10 |
| overall lamb/mutton intake | 1(0.95,1.04) | 0.918295 | 1.02(0.96,1.08) | 0.51385 | 0.99(0.84,1.17) | 0.937635 | 0(0,0) | 0.936214 | 0.99 | 0.567137 | 4 |
| overall pork intake | 1.07(1.01,1.13) | 0.021144 | 1.09(1.02,1.16) | 0.010356 | 0.99(0.84,1.17) | 0.945365 | 0(0,0) | 0.455297 | 1.06 | 0.017075 | 4 |
| overall cheese intake | 0.99(0.96,1.02) | 0.537418 | 1(0.96,1.05) | 0.838461 | 0.97(0.87,1.09) | 0.602299 | 0(0,0) | 0.85491 | 0.96 | 0.00931 | 7 |
| frequency of adding salt to food | 0.98(0.95,1.01) | 0.270063 | 0.98(0.94,1.02) | 0.29438 | 0.99(0.9,1.09) | 0.889307 | 0(0,0) | 0.985775 | 0.99 | 0.287596 | 5 |
| temperature of hot drinks | 1.08(1.04,1.11) | 6.56E-05 | 1.06(1.01,1.11) | 0.013122 | 1.08(0.95,1.24) | 0.230847 | 0(0,0) | 0.899306 | 1.09 | 1.74E-07 | 6 |
| overall alcohol intake | 1(0.97,1.04) | 0.867678 | 1(0.97,1.03) | 0.961172 | 0.94(0.88,1.01) | 0.100951 | 0(0,0) | 0.47993 | 0.97 | 0.033401 | 9 |
| among current drinkers, drinks usually with meals: yes, it varies, no | 1.04(1,1.09) | 0.045725 | 1.03(0.98,1.09) | 0.220348 | 1.06(0.91,1.25) | 0.431315 | 0(0,0) | 0.831484 | 1.06 | 0.00146 | 6 |
| milk type: skimmed, semi-skimmed, full cream (QT) | 0.93(0.87,1) | 0.064424 | 0.94(0.87,1.02) | 0.130965 | 0.74(0.6,0.9) | 0.003559 | 0(0,0.01) | 0.032721 | 0.91 | 0.007897 | 3 |

MR: mendelian randomization; MRPRESSO: MR pleiotropy residual sum and outlier; OR: odd ratio; CI: confidence interval; No.: number.

**Table S25. Leave-one-out analysis of association between genetically predicted dietary habits and difficulty awakening.**

| **Exposure** | **IVW Estimate**  **[Min, Max] ^a^** | **P value [Min, Max] ^b^** |
| --- | --- | --- |
| alcohol drinker status: current + former vs. never | [0.9,0.95] | [0.117992288098223,0.411242910394892] |
| alcohol drinker status: current vs. never | [0.92,0.97] | [0.201703620981585,0.591677504357307] |
| among current drinkers, drinks usually with meals: yes + it varies vs. no | [1.04,1.05] | [0.0199281064171497,0.0928747649238615] |
| among current drinkers, drinks usually with meals: yes vs. no | [1.01,1.02] | [0.0778556871818314,0.28744377338864] |
| never eat eggs vs. no eggs, dairy, wheat, or sugar restrictions | [1.08,1.23] | [0.0410502890861359,0.521838399248591] |
| never eat eggs vs. no eggs restrictions | [1.17,1.32] | [0.0549396033523956,0.265954961238863] |
| never eat dairy vs. no eggs, dairy, wheat, or sugar restrictions | [1.04,1.21] | [0.125871208464902,0.712308522001921] |
| never eat dairy vs. no dairy restrictions | [0.99,1.21] | [0.236782752299982,0.967742663986623] |
| never eat wheat vs. no eggs, dairy, wheat, or sugar restrictions | [1.2,1.24] | [2.51875500644288e-12,1.37149632331359e-06] |
| never eat wheat vs. no wheat restrictions | [1.24,1.29] | [2.43267903334149e-12,8.69863686529856e-07] |
| never eat sugar vs. no eggs, dairy, wheat, or sugar restrictions | [1.09,1.11] | [3.81022587041005e-05,0.000252892155845263] |
| never eat sugar vs. no sugar restrictions | [1.08,1.1] | [0.000202407526911391,0.00113280326540419] |
| milk type: dairy-based milk vs. never | [0.98,1.08] | [0.468420006020091,0.941975386233037] |
| milk type: any milk vs. never | [0.95,1.02] | [0.554826422780434,0.994000778309348] |
| milk type: full cream vs. never | [1.01,1.02] | [0.126846841398542,0.716311181434834] |
| milk type: full cream vs. any other | [0.99,1.06] | [0.435417990392199,0.977454188914701] |
| milk type: semi-skimmed vs. never | [0.91,0.96] | [0.107590243820641,0.476983357806342] |
| milk type: semi-skimmed vs. any other | [0.96,0.99] | [0.257359388457643,0.658756187783814] |
| milk type: skimmed vs. never | [1.03,1.06] | [0.00964321446692893,0.140114091240032] |
| milk type: skimmed vs. any other | [1.02,1.08] | [0.0680768919477262,0.668618963203233] |
| milk type: soy milk vs. never | [0.97,0.98] | [0.00318054329298508,0.0255003423955451] |
| milk type: soy milk vs. any other | [0.81,0.91] | [0.0241206905578863,0.376611021002309] |
| milk type: other milk vs. never | [0.97,0.98] | [0.0014253874970363,0.0351723543855054] |
| milk type: other milk vs. any other | [0.82,0.92] | [0.152947206740144,0.575841719847533] |
| spread type: all spreads vs. never | [0.93,0.96] | [0.0916721753910281,0.275909294483487] |
| spread type: butter + margarine vs. never | [0.97,0.99] | [0.367959025849301,0.856502730318421] |
| spread type: any oil based spread vs. never | [0.94,0.95] | [0.00112717370374169,0.0114663738202442] |
| spread type: butter and butter-like spreads vs. oil-based spreads | [1.06,1.08] | [0.00706635487029402,0.0539139355930851] |
| spread type: butter and margarine spreads vs. oil-based spreads | [1.04,1.06] | [0.0288025828586263,0.143742510708465] |
| spread type: butter vs. never | [0.99,1.02] | [0.407434501295156,0.996268659280689] |
| spread type: butter vs. any other | [0.99,1.01] | [0.596183214191528,0.998196938083475] |
| spread type: tub margarine vs. never | [0.98,0.99] | [0.13114803831441,0.424484000215719] |
| spread type: tub margarine vs. any other | [0.95,0.99] | [0.3332817994536,0.863016645286811] |
| spread type: flora + benecol vs. never | [0.99,1.01] | [0.530035422818476,0.986268253968536] |
| spread type: flora + benecol vs. any other | [1.01,1.06] | [0.0938583233004147,0.763889114833986] |
| spread type: olive oil spread vs. never | [1.02,1.03] | [0.0651463716855797,0.296726151038804] |
| spread type: olive oil spread vs. any other | [0.98,1.03] | [0.548843132769292,0.953564086004646] |
| spread type: other oil-based spread vs. never | [0.97,0.98] | [0.0242306556226619,0.186745064712647] |
| spread type: other oil-based spread vs. any other | [1.06,1.09] | [0.0706941206635179,0.242971965904254] |
| spread type: low fat spread vs. never | [0.97,1] | [0.167585139059141,0.920866465207249] |
| spread type: low fat spread vs. any other | [1.17,1.33] | [0.0116145417839754,0.0694955569777645] |
| bread type: white vs. any other | [0.96,0.98] | [0.0503280927905028,0.196916264021047] |
| bread type: brown vs. any other | [0.96,1.06] | [0.49489520420428,0.989387195763751] |
| bread type: wholemeal/wholegrain vs. any other | [0.99,1] | [0.44025609072667,0.975304229107018] |
| bread type: white vs. wholemeal/wholegrain + brown | [0.97,0.98] | [0.125726222411998,0.375212255451054] |
| bread type: wholemeal/wholegrain vs. white + brown | [0.99,1] | [0.450570464930544,0.97307138094075] |
| cereal type: biscuit cereal vs. any other | [0.88,0.91] | [0.00030910952872762,0.0106989772929667] |
| cereal type: bran cereal vs. any other | [0.99,1.05] | [0.279303211744603,0.993692450712234] |
| cereal type: oat cereal vs. any other | [1.04,1.1] | [0.0136244624234542,0.175615421843997] |
| cereal type: muesli vs. any other | [0.97,0.98] | [0.123747741602369,0.341722054732161] |
| cereal type: cornflakes/frosties vs. any other | [1.04,1.06] | [0.00653546935200912,0.0757001775207811] |
| coffee type: decaffeinated vs. any other | [0.98,0.99] | [0.209754380132875,0.464688476079663] |
| coffee type: ground+instant vs. other+decaff | [0.98,1.01] | [0.515423670575684,0.974831237392419] |
| coffee type: ground vs. any other | [1,1.04] | [0.236706039411246,0.926572762943815] |
| tablespoons of cooked vegetables per day | [1.04,1.06] | [0.00748203018137262,0.0502451575209698] |
| tablespoons of raw vegetables per day | [1,1.01] | [0.736720751812879,0.999163620638517] |
| pieces of fresh fruit per day | [1.12,1.13] | [6.45906557501499e-18,3.29754396437935e-15] |
| pieces of dried fruit per day | [1.01,1.03] | [0.17988251618618,0.528042262514259] |
| slices of bread per week | [0.96,0.97] | [0.0331876953042349,0.13543577691699] |
| bowls of cereal per week | [1.05,1.06] | [0.00401906228700331,0.0206737373885814] |
| cups of tea per day | [0.93,0.94] | [3.51618020074527e-06,0.000100228591124834] |
| cups of coffee per day | [1.02,1.04] | [0.035193680553175,0.193421688384901] |
| glasses of water per day | [1.05,1.06] | [0.000513260983371622,0.00572678230256372] |
| champagne/white wine glasses per month | [0.91,0.93] | [0.000170102327289425,0.00185831026681143] |
| red wine glasses per month | [0.95,0.96] | [0.0139620414381139,0.0413170058459957] |
| beer/cider glasses per month | [0.97,0.98] | [0.121393498316684,0.444221763779203] |
| spirits measures per month | [0.94,0.96] | [0.0696018966733722,0.278153568233376] |
| fortwine glasses per month | [0.98,1.03] | [0.551340102854138,0.990619626972651] |
| other alcohol glasses per month | [0.99,1.01] | [0.689413235261051,0.986797483489568] |
| total drinks of alcohol per month | [0.96,0.97] | [0.00112059730711276,0.00545966287212684] |
| overall oily fish intake | [0.99,1] | [0.670130735327169,0.999441331229494] |
| overall non-oily fish intake | [1.02,1.05] | [0.124121377090957,0.512935075106324] |
| overall processed meat intake | [0.91,0.92] | [5.24786665220384e-05,0.000311550031115594] |
| overall poultry intake | [1.01,1.04] | [0.263466929482003,0.795163095939085] |
| overall beef intake | [0.97,0.99] | [0.229411610967562,0.605611341894778] |
| overall lamb/mutton intake | [0.98,0.99] | [0.382046338707769,0.779702520032363] |
| overall pork intake | [1.05,1.07] | [0.0113475413853286,0.0830803127639589] |
| overall cheese intake | [0.96,0.96] | [0.00694140627404552,0.0213739208376173] |
| frequency of adding salt to food | [0.99,0.99] | [0.46334432279072,0.727374105882869] |
| temperature of hot drinks | [1.08,1.1] | [1.36340300616256e-07,3.91636689794e-06] |
| overall alcohol intake | [0.96,0.97] | [0.00431780057395925,0.0182123168169633] |
| among current drinkers, drinks usually with meals: yes, it varies, no | [1.04,1.06] | [0.00786857256273947,0.0620271831602821] |
| milk type: skimmed, semi-skimmed, full cream (QT) | [0.89,0.93] | [0.00121877598540653,0.0394594079193354] |

**^a^** the minimum value and maximum value of inverse variance weighted estimate;

**^b^** the minimum value and maximum value of P value;

IVW: inverse variance weighted.

**Table S26. Associations between genetically predicted dietary habits and insomnia in sensitivity analysis.**

| **Exposure** | **Weighted median** | | **Mode-based** | | **MR-Egger** | | | | **MRPRESSO** | | |
| --- | --- | --- | --- | --- | --- | --- | --- | --- | --- | --- | --- |
|  | **OR (95% CI)** | **P value** | **OR (95% CI)** | **P value** | **OR (95% CI)** | **P value** | **Intercept** | **P value** | **OR** | **P value** | **No. of outliers** |
| alcohol drinker status: current + former vs. never | 0.87(0.53,1.42) | 0.578911 | 0.89(0.55,1.43) | 0.619669 | 0.93(0.38,2.27) | 0.872741 | 0(-0.01,0.01) | 0.967108 | 0.95 | 0.791017 | 1 |
| alcohol drinker status: current vs. never | 0.91(0.57,1.46) | 0.706351 | 0.9(0.57,1.43) | 0.664194 | 1.35(0.57,3.16) | 0.495321 | 0(-0.01,0) | 0.383559 | 0.96 | 0.839031 | 1 |
| among current drinkers, drinks usually with meals: yes + it varies vs. no | 0.68(0.59,0.78) | 4.18E-08 | 0.67(0.56,0.8) | 6.87E-06 | 1.01(0.65,1.57) | 0.969443 | -0.01(-0.01,0) | 0.049707 | 0.67 | 1.58E-10 | 1 |
| among current drinkers, drinks usually with meals: yes vs. no | 0.76(0.7,0.82) | 7.98E-12 | 0.73(0.66,0.82) | 3.61E-08 | 0.83(0.66,1.04) | 0.108603 | 0(-0.01,0) | 0.315584 | 0.74 | 4.51E-17 | 1 |
| never eat eggs vs. no eggs, dairy, wheat, or sugar restrictions | 1.72(0.67,4.44) | 0.258901 | 1.73(0.76,3.94) | 0.189811 | 3.3(0.17,64.29) | 0.429936 | 0(-0.03,0.02) | 0.717596 | 1.96 | 0.165744 | 1 |
| never eat eggs vs. no eggs restrictions | 1.93(0.55,6.84) | 0.306986 | 1.62(0.49,5.38) | 0.429624 | 6.13(0.08,482.92) | 0.41562 | -0.01(-0.04,0.02) | 0.592135 | 2.01 | 0.381759 | 1 |
| never eat dairy vs. no eggs, dairy, wheat, or sugar restrictions | 0.99(0.5,1.96) | 0.969015 | 1.24(0.64,2.38) | 0.523038 | 0.56(0.13,2.54) | 0.454822 | 0(-0.01,0.02) | 0.442881 | 0.97 | 0.918228 | 0 |
| never eat dairy vs. no dairy restrictions | 0.93(0.4,2.18) | 0.867841 | 1.24(0.55,2.76) | 0.602417 | 0.56(0.09,3.63) | 0.543706 | 0(-0.01,0.01) | 0.55822 | 0.94 | 0.860682 | 0 |
| never eat wheat vs. no eggs, dairy, wheat, or sugar restrictions | 0.88(0.68,1.15) | 0.353541 | 0.97(0.78,1.21) | 0.812081 | 0.87(0.59,1.28) | 0.482833 | 0(0,0.01) | 0.320468 | 1.02 | 0.841608 | 0 |
| never eat wheat vs. no wheat restrictions | 0.86(0.62,1.18) | 0.353009 | 0.95(0.72,1.24) | 0.689232 | 0.91(0.61,1.37) | 0.656937 | 0(0,0.01) | 0.455554 | 1.04 | 0.750896 | 0 |
| never eat sugar vs. no eggs, dairy, wheat, or sugar restrictions | 1.12(0.97,1.3) | 0.132025 | 1.05(0.87,1.27) | 0.59651 | 1.57(0.98,2.52) | 0.062127 | 0(-0.01,0) | 0.222288 | 1.16 | 0.029206 | 1 |
| never eat sugar vs. no sugar restrictions | 1.21(1.03,1.41) | 0.018415 | 1.18(0.96,1.46) | 0.112679 | 1.61(1.01,2.57) | 0.044208 | 0(-0.01,0) | 0.260344 | 1.22 | 0.003052 | 1 |
| milk type: dairy-based milk vs. never | 1.13(0.57,2.24) | 0.721975 | 0.9(0.47,1.71) | 0.740454 | 2.03(0.28,15.02) | 0.486383 | -0.01(-0.02,0.01) | 0.343701 | 0.98 | 0.943505 | 1 |
| milk type: any milk vs. never | 1.02(0.53,1.98) | 0.952654 | 0.91(0.47,1.78) | 0.791517 | 1.04(0.3,3.64) | 0.951645 | 0(-0.01,0.01) | 0.618664 | 0.77 | 0.243213 | 0 |
| milk type: full cream vs. never | 0.96(0.88,1.05) | 0.402776 | 0.99(0.91,1.08) | 0.809153 | 0.98(0.74,1.31) | 0.904206 | 0(-0.02,0.01) | 0.794781 | 0.98 | 0.6386 | 1 |
| milk type: full cream vs. any other | 1.07(0.72,1.58) | 0.744333 | 1.06(0.71,1.57) | 0.783447 | 0.43(0.1,1.9) | 0.266355 | 0.01(0,0.02) | 0.217173 | 1.04 | 0.815903 | 2 |
| milk type: semi-skimmed vs. never | 1.11(0.66,1.87) | 0.691388 | 1.1(0.67,1.81) | 0.695277 | 1.88(0.36,9.76) | 0.45148 | -0.01(-0.03,0.01) | 0.255243 | 1.03 | 0.863185 | 2 |
| milk type: semi-skimmed vs. any other | 0.85(0.67,1.08) | 0.178472 | 0.81(0.64,1.04) | 0.100191 | 0.52(0.28,0.96) | 0.036291 | 0.01(0,0.02) | 0.129074 | 0.77 | 0.007609 | 1 |
| milk type: skimmed vs. never | 0.98(0.82,1.17) | 0.82278 | 0.97(0.81,1.16) | 0.757513 | 0.91(0.62,1.33) | 0.624024 | 0(-0.01,0.01) | 0.997393 | 0.91 | 0.237601 | 0 |
| milk type: skimmed vs. any other | 1.11(0.87,1.41) | 0.407645 | 1.07(0.83,1.38) | 0.603524 | 1.26(0.65,2.46) | 0.488904 | 0(-0.01,0.01) | 0.671345 | 1.16 | 0.108552 | 1 |
| milk type: soy milk vs. never | 0.98(0.92,1.05) | 0.577376 | 0.97(0.9,1.05) | 0.445704 | 0.94(0.83,1.07) | 0.36375 | 0(-0.01,0.01) | 0.51246 | 0.98 | 0.415017 | 0 |
| milk type: soy milk vs. any other | 0.62(0.33,1.17) | 0.14167 | 0.57(0.29,1.11) | 0.098629 | 1.32(0.23,7.42) | 0.754453 | 0(-0.02,0.01) | 0.534511 | 0.78 | 0.386208 | 0 |
| milk type: other milk vs. never | 1.05(0.97,1.14) | 0.250419 | 1.05(0.96,1.14) | 0.269413 | 1(0.84,1.18) | 0.956049 | 0(-0.01,0.02) | 0.683427 | 1.03 | 0.488896 | 0 |
| milk type: other milk vs. any other | 0.39(0.1,1.44) | 0.155785 | 0.4(0.11,1.41) | 0.153984 | 2.89(0.24,34.54) | 0.402535 | -0.01(-0.03,0.01) | 0.2742 | 0.85 | 0.785205 | 0 |
| spread type: all spreads vs. never | 1.04(0.8,1.35) | 0.786186 | 1(0.75,1.33) | 0.978948 | 0.89(0.46,1.71) | 0.724494 | 0(-0.01,0.01) | 0.593866 | 1.05 | 0.651803 | 1 |
| spread type: butter + margarine vs. never | 1(0.84,1.18) | 1 | 0.99(0.83,1.18) | 0.913621 | 1.09(0.64,1.85) | 0.756221 | 0(-0.01,0.01) | 0.702391 | 1.03 | 0.668056 | 1 |
| spread type: any oil based spread vs. never | 1.06(0.91,1.24) | 0.435674 | 1.05(0.89,1.24) | 0.540963 | 0.96(0.63,1.47) | 0.854671 | 0(-0.01,0.01) | 0.664682 | 1.05 | 0.446031 | 0 |
| spread type: butter and butter-like spreads vs. oil-based spreads | 1.11(0.93,1.33) | 0.263059 | 1.14(0.94,1.39) | 0.17992 | 1.14(0.79,1.66) | 0.478618 | 0(-0.01,0) | 0.41597 | 0.99 | 0.875888 | 0 |
| spread type: butter and margarine spreads vs. oil-based spreads | 1.12(0.97,1.31) | 0.132079 | 1.16(0.99,1.37) | 0.071184 | 1.06(0.74,1.52) | 0.735525 | 0(-0.01,0.01) | 0.761452 | 1.01 | 0.876081 | 0 |
| spread type: butter vs. never | 1.05(0.92,1.19) | 0.487422 | 1.04(0.89,1.21) | 0.618616 | 1.19(0.85,1.66) | 0.313775 | 0(-0.01,0) | 0.408242 | 1.04 | 0.463997 | 0 |
| spread type: butter vs. any other | 0.99(0.86,1.14) | 0.916065 | 0.98(0.82,1.16) | 0.796371 | 1.06(0.7,1.61) | 0.778043 | 0(-0.01,0) | 0.580235 | 0.95 | 0.371833 | 1 |
| spread type: tub margarine vs. never | 1.08(0.97,1.2) | 0.161499 | 1.03(0.92,1.15) | 0.619888 | 1.03(0.81,1.31) | 0.806069 | 0(-0.01,0.01) | 0.859524 | 1.05 | 0.260282 | 0 |
| spread type: tub margarine vs. any other | 1.23(0.77,1.96) | 0.379067 | 1.07(0.67,1.7) | 0.778084 | 1.25(0.36,4.39) | 0.72834 | 0(-0.01,0.01) | 0.931748 | 1.15 | 0.444427 | 2 |
| spread type: flora + benecol vs. never | 0.98(0.88,1.09) | 0.711553 | 0.98(0.88,1.1) | 0.734477 | 0.84(0.68,1.04) | 0.117463 | 0.01(0,0.02) | 0.155042 | 0.97 | 0.526753 | 1 |
| spread type: flora + benecol vs. any other | 0.95(0.71,1.28) | 0.748727 | 0.99(0.75,1.3) | 0.918223 | 0.84(0.53,1.33) | 0.459068 | 0(0,0.01) | 0.319188 | 1.03 | 0.750518 | 0 |
| spread type: olive oil spread vs. never | 0.98(0.86,1.11) | 0.755044 | 1(0.88,1.15) | 0.941433 | 0.83(0.63,1.11) | 0.210393 | 0(-0.01,0.01) | 0.395683 | 0.94 | 0.172787 | 0 |
| spread type: olive oil spread vs. any other | 0.62(0.4,0.96) | 0.031563 | 0.62(0.41,0.94) | 0.023693 | 0.31(0.11,0.84) | 0.020764 | 0.01(0,0.03) | 0.066421 | 0.59 | 0.003868 | 1 |
| spread type: other oil-based spread vs. never | 1.02(0.91,1.15) | 0.700964 | 1(0.88,1.14) | 0.994585 | 0.99(0.75,1.31) | 0.955341 | 0(-0.01,0.01) | 0.912802 | 1.01 | 0.898491 | 1 |
| spread type: other oil-based spread vs. any other | 1.12(0.81,1.55) | 0.506288 | 1.28(0.93,1.75) | 0.128299 | 1.59(0.69,3.64) | 0.272401 | -0.01(-0.02,0.01) | 0.300901 | 1.18 | 0.193375 | 1 |
| spread type: low fat spread vs. never | 1.06(0.92,1.23) | 0.390265 | 1.06(0.92,1.22) | 0.396164 | 1.06(0.82,1.36) | 0.654901 | 0(-0.01,0.01) | 0.706559 | 1.01 | 0.800812 | 0 |
| spread type: low fat spread vs. any other | 1.08(0.56,2.06) | 0.822746 | 1.09(0.57,2.08) | 0.797182 | 2.38(0.51,11.12) | 0.270398 | -0.01(-0.02,0.01) | 0.362308 | 1.49 | 0.179786 | 1 |
| bread type: white vs. any other | 1.35(1.21,1.5) | 1.09E-07 | 1.34(1.15,1.55) | 0.000112 | 1.2(0.85,1.71) | 0.297571 | 0(0,0.01) | 0.598605 | 1.36 | 3.14E-10 | 3 |
| bread type: brown vs. any other | 0.89(0.57,1.39) | 0.603857 | 0.99(0.64,1.52) | 0.950405 | 1.33(0.5,3.51) | 0.567074 | -0.01(-0.02,0) | 0.273382 | 0.8 | 0.249992 | 0 |
| bread type: wholemeal/wholegrain vs. any other | 0.77(0.69,0.86) | 3.49E-06 | 0.76(0.65,0.89) | 0.000452 | 1.13(0.79,1.61) | 0.510632 | -0.01(-0.01,0) | 0.024008 | 0.77 | 7.30E-08 | 1 |
| bread type: white vs. wholemeal/wholegrain + brown | 1.34(1.2,1.48) | 5.27E-08 | 1.32(1.15,1.52) | 0.000111 | 1.2(0.87,1.66) | 0.265861 | 0(0,0.01) | 0.564412 | 1.33 | 8.93E-10 | 1 |
| bread type: wholemeal/wholegrain vs. white + brown | 0.74(0.67,0.82) | 4.35E-09 | 0.75(0.65,0.86) | 6.10E-05 | 0.98(0.73,1.31) | 0.869145 | 0(-0.01,0) | 0.063418 | 0.74 | 1.26E-11 | 1 |
| cereal type: biscuit cereal vs. any other | 1.28(0.97,1.7) | 0.085942 | 1.12(0.82,1.51) | 0.482815 | 1.24(0.63,2.45) | 0.529341 | 0(-0.01,0.01) | 0.935875 | 1.28 | 0.060542 | 1 |
| cereal type: bran cereal vs. any other | 0.96(0.69,1.34) | 0.810163 | 0.89(0.64,1.24) | 0.483766 | 1.45(0.74,2.86) | 0.277779 | -0.01(-0.02,0) | 0.193566 | 0.97 | 0.814472 | 0 |
| cereal type: oat cereal vs. any other | 1.11(0.82,1.51) | 0.501015 | 1.13(0.85,1.51) | 0.387896 | 0.97(0.41,2.28) | 0.946202 | 0(-0.01,0.02) | 0.736145 | 1.19 | 0.247489 | 1 |
| cereal type: muesli vs. any other | 0.67(0.58,0.78) | 8.96E-08 | 0.7(0.58,0.85) | 0.000212 | 0.89(0.57,1.39) | 0.609716 | 0(-0.01,0) | 0.237635 | 0.67 | 1.33E-09 | 1 |
| cereal type: cornflakes/frosties vs. any other | 1.09(0.93,1.29) | 0.288085 | 1.15(0.95,1.38) | 0.149378 | 0.79(0.5,1.24) | 0.300713 | 0.01(0,0.01) | 0.050914 | 1.23 | 0.006803 | 1 |
| coffee type: decaffeinated vs. any other | 0.76(0.68,0.84) | 1.09E-07 | 0.76(0.67,0.88) | 0.000151 | 0.75(0.55,1.02) | 0.068485 | 0(0,0.01) | 0.703034 | 0.78 | 3.78E-09 | 1 |
| coffee type: ground+instant vs. other+decaff | 0.71(0.54,0.93) | 0.01184 | 0.84(0.65,1.08) | 0.172721 | 0.76(0.37,1.53) | 0.435837 | 0(-0.01,0.01) | 0.702272 | 0.69 | 0.009736 | 1 |
| coffee type: ground vs. any other | 1.07(0.82,1.38) | 0.627059 | 1.04(0.8,1.34) | 0.782349 | 1.41(0.78,2.55) | 0.261346 | 0(-0.01,0.01) | 0.691281 | 1.2 | 0.118973 | 1 |
| tablespoons of cooked vegetables per day | 1(0.87,1.15) | 1 | 1.03(0.86,1.23) | 0.752384 | 0.76(0.48,1.2) | 0.237506 | 0(0,0.01) | 0.219258 | 1.05 | 0.381071 | 3 |
| tablespoons of raw vegetables per day | 1.07(0.94,1.21) | 0.299221 | 1.05(0.9,1.23) | 0.524947 | 1.15(0.76,1.75) | 0.517171 | 0(-0.01,0) | 0.647944 | 1.07 | 0.194303 | 4 |
| pieces of fresh fruit per day | 0.87(0.79,0.95) | 0.00209 | 0.89(0.8,0.99) | 0.039415 | 0.85(0.66,1.1) | 0.218221 | 0(0,0) | 0.912652 | 0.84 | 6.72E-06 | 3 |
| pieces of dried fruit per day | 0.72(0.63,0.83) | 2.90E-06 | 0.75(0.63,0.88) | 0.000741 | 0.65(0.43,1) | 0.048854 | 0(0,0.01) | 0.571754 | 0.72 | 2.00E-07 | 1 |
| slices of bread per week | 1(0.88,1.14) | 1 | 1.02(0.88,1.19) | 0.782487 | 1(0.68,1.47) | 0.99405 | 0(-0.01,0) | 0.719828 | 0.96 | 0.492359 | 4 |
| bowls of cereal per week | 0.85(0.76,0.95) | 0.003095 | 0.91(0.79,1.05) | 0.211386 | 0.82(0.61,1.11) | 0.197515 | 0(0,0) | 0.954656 | 0.81 | 1.15E-06 | 1 |
| cups of tea per day | 1(0.88,1.13) | 1 | 1.01(0.91,1.13) | 0.795492 | 0.97(0.77,1.21) | 0.782899 | 0(0,0) | 0.773184 | 1.02 | 0.576845 | 2 |
| cups of coffee per day | 1(0.88,1.14) | 1 | 1(0.91,1.1) | 0.989398 | 1.03(0.85,1.24) | 0.756527 | 0(-0.01,0) | 0.172412 | 0.91 | 0.04755 | 1 |
| glasses of water per day | 1(0.9,1.11) | 1 | 0.97(0.87,1.09) | 0.643336 | 0.94(0.74,1.19) | 0.590857 | 0(0,0) | 0.614563 | 0.98 | 0.534253 | 2 |
| champagne/white wine glasses per month | 1.04(0.88,1.23) | 0.648083 | 1.04(0.88,1.24) | 0.617882 | 1.04(0.67,1.6) | 0.867266 | 0(-0.01,0) | 0.564779 | 0.96 | 0.594179 | 4 |
| red wine glasses per month | 0.86(0.76,0.97) | 0.011933 | 0.89(0.78,1.01) | 0.069143 | 1.01(0.77,1.33) | 0.916689 | 0(-0.01,0) | 0.057648 | 0.8 | 2.84E-05 | 1 |
| beer/cider glasses per month | 1.1(0.95,1.27) | 0.19653 | 1.09(0.96,1.25) | 0.196748 | 0.98(0.76,1.28) | 0.902199 | 0(0,0.01) | 0.258699 | 1.16 | 0.002967 | 2 |
| spirits measures per month | 1.17(0.93,1.46) | 0.178388 | 1.27(1,1.6) | 0.046534 | 1.55(0.9,2.67) | 0.114941 | -0.01(-0.01,0) | 0.103133 | 1.11 | 0.269695 | 2 |
| fortwine glasses per month | 0.89(0.59,1.33) | 0.572145 | 0.82(0.56,1.22) | 0.338299 | 1.21(0.5,2.94) | 0.674411 | 0(-0.02,0.01) | 0.453603 | 0.88 | 0.428997 | 0 |
| other alcohol glasses per month | 0.83(0.61,1.13) | 0.240759 | 0.94(0.7,1.27) | 0.70183 | 0.51(0.28,0.95) | 0.033275 | 0.01(0,0.03) | 0.038548 | 0.94 | 0.632299 | 0 |
| total drinks of alcohol per month | 0.96(0.88,1.06) | 0.431508 | 0.98(0.89,1.07) | 0.599042 | 1.05(0.91,1.21) | 0.528264 | 0(0,0) | 0.053338 | 0.91 | 0.008866 | 1 |
| overall oily fish intake | 0.85(0.77,0.94) | 0.00214 | 0.86(0.76,0.98) | 0.023311 | 0.97(0.7,1.35) | 0.850874 | 0(-0.01,0) | 0.418639 | 0.85 | 0.000139 | 2 |
| overall non-oily fish intake | 1.05(0.88,1.25) | 0.570002 | 1.05(0.85,1.28) | 0.651691 | 1.62(1.03,2.57) | 0.038676 | -0.01(-0.01,0) | 0.022728 | 0.98 | 0.73152 | 1 |
| overall processed meat intake | 1.2(1.03,1.38) | 0.016103 | 1.14(0.95,1.38) | 0.150013 | 1.01(0.63,1.59) | 0.979501 | 0(0,0.01) | 0.394707 | 1.19 | 0.003152 | 3 |
| overall poultry intake | 1.16(0.96,1.42) | 0.132176 | 1.21(0.97,1.51) | 0.085399 | 1.3(0.75,2.23) | 0.346741 | 0(-0.01,0) | 0.478349 | 1.16 | 0.047258 | 2 |
| overall beef intake | 1.13(0.98,1.31) | 0.091054 | 1.14(0.97,1.35) | 0.118498 | 1(0.65,1.56) | 0.983067 | 0(-0.01,0.01) | 0.841511 | 1.08 | 0.261264 | 1 |
| overall lamb/mutton intake | 0.93(0.81,1.06) | 0.259879 | 0.96(0.83,1.13) | 0.647389 | 0.97(0.64,1.49) | 0.900838 | 0(-0.01,0) | 0.662512 | 0.84 | 0.002059 | 5 |
| overall pork intake | 0.98(0.84,1.15) | 0.829264 | 1.02(0.85,1.23) | 0.822093 | 1.13(0.78,1.64) | 0.531145 | 0(-0.01,0) | 0.545753 | 1.01 | 0.865693 | 0 |
| overall cheese intake | 0.85(0.77,0.95) | 0.002779 | 0.91(0.8,1.04) | 0.182268 | 0.72(0.52,0.99) | 0.045696 | 0(0,0.01) | 0.283397 | 0.86 | 0.000996 | 3 |
| frequency of adding salt to food | 1.11(1.01,1.23) | 0.031224 | 1.14(1.01,1.3) | 0.040999 | 1.14(0.85,1.53) | 0.372201 | 0(0,0) | 0.928488 | 1.14 | 0.000878 | 3 |
| temperature of hot drinks | 1.14(1.02,1.27) | 0.021801 | 1.08(0.95,1.23) | 0.26259 | 1.15(0.81,1.63) | 0.434677 | 0(0,0) | 0.908989 | 1.16 | 0.0018 | 1 |
| overall alcohol intake | 0.93(0.84,1.02) | 0.106962 | 0.93(0.85,1.03) | 0.153704 | 0.99(0.83,1.18) | 0.887834 | 0(0,0) | 0.182488 | 0.89 | 0.000413 | 5 |
| among current drinkers, drinks usually with meals: yes, it varies, no | 0.69(0.61,0.78) | 1.86E-09 | 0.73(0.62,0.85) | 3.98E-05 | 0.71(0.48,1.06) | 0.090961 | 0(-0.01,0.01) | 0.86308 | 0.66 | 1.20E-14 | 3 |
| milk type: skimmed, semi-skimmed, full cream (QT) | 0.91(0.75,1.11) | 0.364047 | 0.94(0.77,1.15) | 0.536422 | 1.55(0.91,2.64) | 0.105397 | -0.01(-0.02,0) | 0.016065 | 0.83 | 0.041608 | 1 |

MR: mendelian randomization; MRPRESSO: MR pleiotropy residual sum and outlier; OR: odd ratio; CI: confidence interval; No.: number.

**Table S27. Leave-one-out analysis of association between genetically predicted dietary habits and insomnia.**

| **Exposure** | **IVW Estimate**  **[Min, Max] ^a^** | **P value [Min, Max] ^b^** |
| --- | --- | --- |
| alcohol drinker status: current + former vs. never | [0.87,1.01] | [0.503108497516908,0.996386181072174] |
| alcohol drinker status: current vs. never | [0.88,1.03] | [0.506661588785753,0.989184423801434] |
| among current drinkers, drinks usually with meals: yes + it varies vs. no | [0.65,0.68] | [8.76834802483661e-13,5.19251605892128e-11] |
| among current drinkers, drinks usually with meals: yes vs. no | [0.73,0.74] | [2.75969439410262e-23,9.46603662504371e-21] |
| never eat eggs vs. no eggs, dairy, wheat, or sugar restrictions | [1.55,2.49] | [0.0286467963201152,0.302573828000832] |
| never eat eggs vs. no eggs restrictions | [1.24,3.12] | [0.100992069943751,0.738838579319702] |
| never eat dairy vs. no eggs, dairy, wheat, or sugar restrictions | [0.89,1.23] | [0.418828396639016,0.969049513065835] |
| never eat dairy vs. no dairy restrictions | [0.83,1.25] | [0.484023492309239,0.989078153415937] |
| never eat wheat vs. no eggs, dairy, wheat, or sugar restrictions | [0.96,1.1] | [0.48948174998501,0.972280957155962] |
| never eat wheat vs. no wheat restrictions | [0.96,1.13] | [0.389381788193803,0.966164991789508] |
| never eat sugar vs. no eggs, dairy, wheat, or sugar restrictions | [1.16,1.2] | [0.00570062953576257,0.0270572157640943] |
| never eat sugar vs. no sugar restrictions | [1.22,1.27] | [0.000408703588235055,0.00239291525715289] |
| milk type: dairy-based milk vs. never | [0.69,0.98] | [0.191111473223946,0.942404882505598] |
| milk type: any milk vs. never | [0.72,0.84] | [0.188865254637247,0.478094615605947] |
| milk type: full cream vs. never | [0.93,0.98] | [0.170413500579504,0.633430529656362] |
| milk type: full cream vs. any other | [0.94,1.17] | [0.39209079291506,0.951372891093239] |
| milk type: semi-skimmed vs. never | [0.69,0.88] | [0.155311594504862,0.557197127112864] |
| milk type: semi-skimmed vs. any other | [0.77,0.86] | [0.00380897408169376,0.122579068318396] |
| milk type: skimmed vs. never | [0.88,0.95] | [0.0818665630354425,0.469959173388762] |
| milk type: skimmed vs. any other | [1.07,1.16] | [0.0996008262125598,0.497001431482487] |
| milk type: soy milk vs. never | [0.97,0.99] | [0.191592952557071,0.699043615915049] |
| milk type: soy milk vs. any other | [0.66,0.86] | [0.109144071195077,0.569157044851457] |
| milk type: other milk vs. never | [1.01,1.05] | [0.124496381176273,0.803416520410109] |
| milk type: other milk vs. any other | [0.61,1.06] | [0.347739213514681,0.997554739538603] |
| spread type: all spreads vs. never | [1.01,1.08] | [0.452014758894297,0.917050287936396] |
| spread type: butter + margarine vs. never | [0.96,1.03] | [0.646317763910504,0.983885557190031] |
| spread type: any oil based spread vs. never | [1.03,1.08] | [0.198806510303311,0.688401385753774] |
| spread type: butter and butter-like spreads vs. oil-based spreads | [0.97,1.02] | [0.649449709365971,0.970209902562172] |
| spread type: butter and margarine spreads vs. oil-based spreads | [0.99,1.03] | [0.608513416157651,0.99101029006669] |
| spread type: butter vs. never | [1,1.05] | [0.297653796172525,0.939778322799807] |
| spread type: butter vs. any other | [0.93,0.96] | [0.231292865850017,0.53743884737523] |
| spread type: tub margarine vs. never | [1.03,1.07] | [0.0891780540331353,0.457814680753591] |
| spread type: tub margarine vs. any other | [1.04,1.32] | [0.20869268895386,0.837835687305609] |
| spread type: flora + benecol vs. never | [0.95,0.99] | [0.258841890077042,0.779287031227019] |
| spread type: flora + benecol vs. any other | [0.99,1.1] | [0.394636730945976,0.976847854964393] |
| spread type: olive oil spread vs. never | [0.92,0.96] | [0.0587365383522628,0.34588280987815] |
| spread type: olive oil spread vs. any other | [0.59,0.78] | [0.000924609849914415,0.298421712684722] |
| spread type: other oil-based spread vs. never | [0.99,1.02] | [0.648615836685118,0.986231040629276] |
| spread type: other oil-based spread vs. any other | [1.01,1.18] | [0.178315512545678,0.951857125805692] |
| spread type: low fat spread vs. never | [0.99,1.05] | [0.371675720640875,0.977643750917376] |
| spread type: low fat spread vs. any other | [1.04,1.49] | [0.151985444325923,0.897506802140418] |
| bread type: white vs. any other | [1.31,1.33] | [1.29958852172758e-09,3.3702593444342e-08] |
| bread type: brown vs. any other | [0.75,0.89] | [0.130599242128295,0.471524217153379] |
| bread type: wholemeal/wholegrain vs. any other | [0.75,0.76] | [5.12348804541308e-10,1.66107555347507e-08] |
| bread type: white vs. wholemeal/wholegrain + brown | [1.3,1.33] | [6.72161438042084e-11,2.34253255091925e-09] |
| bread type: wholemeal/wholegrain vs. white + brown | [0.74,0.75] | [2.0266660519643e-13,1.4669739474592e-11] |
| cereal type: biscuit cereal vs. any other | [1.22,1.33] | [0.0165106764383679,0.105940345824816] |
| cereal type: bran cereal vs. any other | [0.89,1.03] | [0.372007646806088,0.99148220313757] |
| cereal type: oat cereal vs. any other | [1.05,1.19] | [0.232801881665426,0.755353499657068] |
| cereal type: muesli vs. any other | [0.67,0.7] | [1.07381327432528e-11,6.09076722530218e-09] |
| cereal type: cornflakes/frosties vs. any other | [1.18,1.23] | [0.00520231825147436,0.0265709248713438] |
| coffee type: decaffeinated vs. any other | [0.78,0.8] | [3.155054284136e-10,5.06463761847353e-08] |
| coffee type: ground+instant vs. other+decaff | [0.64,0.69] | [0.000852883587568095,0.00874419114270204] |
| coffee type: ground vs. any other | [1.2,1.3] | [0.0252039484030123,0.146702742815008] |
| tablespoons of cooked vegetables per day | [0.99,1.02] | [0.711645800174211,0.999226589595412] |
| tablespoons of raw vegetables per day | [1.03,1.06] | [0.309949762333389,0.60505421620907] |
| pieces of fresh fruit per day | [0.83,0.85] | [9.38301131780344e-07,1.01092264683355e-05] |
| pieces of dried fruit per day | [0.72,0.75] | [3.08953847428806e-08,2.69624959531738e-06] |
| slices of bread per week | [0.92,0.95] | [0.158614357259423,0.364507331935063] |
| bowls of cereal per week | [0.81,0.82] | [1.27579765760414e-07,1.8203768878091e-06] |
| cups of tea per day | [0.99,1.01] | [0.727481296605484,0.999857874502604] |
| cups of coffee per day | [0.9,0.93] | [0.0388058432787847,0.141102555966047] |
| glasses of water per day | [0.98,1] | [0.680197963206957,0.974128201932152] |
| champagne/white wine glasses per month | [0.9,0.95] | [0.180075562974925,0.488074787811884] |
| red wine glasses per month | [0.77,0.8] | [8.19764968060924e-07,1.52020394783212e-05] |
| beer/cider glasses per month | [1.12,1.15] | [0.00706330668784054,0.043032741978878] |
| spirits measures per month | [1,1.07] | [0.514309972067916,0.997780336858075] |
| fortwine glasses per month | [0.8,0.95] | [0.150782983183125,0.72990051278061] |
| other alcohol glasses per month | [0.84,0.98] | [0.147341314947123,0.88496567578461] |
| total drinks of alcohol per month | [0.9,0.93] | [0.00638222850168905,0.0352779831595719] |
| overall oily fish intake | [0.84,0.86] | [5.24930666778567e-05,0.000415904438054325] |
| overall non-oily fish intake | [0.94,0.98] | [0.386058233493054,0.754392740810653] |
| overall processed meat intake | [1.19,1.24] | [0.00054016367275093,0.00432565887553593] |
| overall poultry intake | [1.05,1.12] | [0.167039022161239,0.588798817821483] |
| overall beef intake | [1.03,1.08] | [0.258388768900067,0.680772321318861] |
| overall lamb/mutton intake | [0.87,0.9] | [0.0228177033702783,0.0946082044598494] |
| overall pork intake | [0.98,1.03] | [0.625351030434067,0.99441687901716] |
| overall cheese intake | [0.85,0.86] | [0.000272030688347686,0.00135885957523822] |
| frequency of adding salt to food | [1.14,1.18] | [9.87916228255026e-05,0.00106224475428532] |
| temperature of hot drinks | [1.16,1.18] | [0.000440515396191937,0.00191853578084182] |
| overall alcohol intake | [0.87,0.89] | [0.00024963572355983,0.00173075609303977] |
| among current drinkers, drinks usually with meals: yes, it varies, no | [0.68,0.7] | [7.73557302807683e-15,1.68255328793427e-12] |
| milk type: skimmed, semi-skimmed, full cream (QT) | [0.81,0.86] | [0.011793814283698,0.061200665852211] |

**^a^** the minimum value and maximum value of inverse variance weighted estimate;

**^b^** the minimum value and maximum value of P value;

IVW: inverse variance weighted.

**Table S28. Associations between genetically predicted dietary habits and major depression disorder in sensitivity analysis.**

| **Exposure** | **Weighted median** | | **Mode-based** | | **MR-Egger** | | | | **MRPRESSO** | | |
| --- | --- | --- | --- | --- | --- | --- | --- | --- | --- | --- | --- |
|  | **OR (95% CI)** | **P value** | **OR (95% CI)** | **P value** | **OR (95% CI)** | **P value** | **Intercept** | **P value** | **OR** | **P value** | **No. of outliers** |
| alcohol drinker status: current + former vs. never | 0.84(0.56,1.25) | 0.39665 | 0.92(0.6,1.41) | 0.704538 | 1.69(0.69,4.12) | 0.250727 | 0(-0.01,0) | 0.308763 | 1 | 0.976167 | 1 |
| alcohol drinker status: current vs. never | 0.78(0.53,1.15) | 0.204471 | 0.74(0.49,1.12) | 0.1608 | 1.65(0.66,4.12) | 0.283745 | 0(-0.01,0) | 0.329341 | 0.99 | 0.926114 | 1 |
| among current drinkers, drinks usually with meals: yes + it varies vs. no | 0.75(0.67,0.84) | 5.17E-07 | 0.78(0.68,0.89) | 0.000299 | 1.01(0.66,1.55) | 0.961583 | 0(-0.01,0) | 0.101563 | 0.75 | 9.11E-08 | 7 |
| among current drinkers, drinks usually with meals: yes vs. no | 0.79(0.74,0.85) | 4.69E-11 | 0.8(0.72,0.88) | 2.21E-06 | 0.87(0.68,1.12) | 0.278005 | 0(-0.01,0) | 0.477832 | 0.83 | 6.64E-10 | 8 |
| never eat eggs vs. no eggs, dairy, wheat, or sugar restrictions | 1.36(0.71,2.58) | 0.353808 | 1.23(0.66,2.28) | 0.508518 | 1.01(0.33,3.09) | 0.987222 | 0(0,0.01) | 0.300476 | 1.73 | 0.042266 | 0 |
| never eat eggs vs. no eggs restrictions | 1.19(0.46,3.06) | 0.722334 | 1.38(0.57,3.34) | 0.479249 | 0.77(0.14,4.2) | 0.763392 | 0.01(-0.01,0.02) | 0.346038 | 1.63 | 0.203312 | 0 |
| never eat dairy vs. no eggs, dairy, wheat, or sugar restrictions | 0.55(0.32,0.95) | 0.031442 | 0.5(0.29,0.86) | 0.012735 | 0.16(0.04,0.62) | 0.007911 | 0.01(0,0.02) | 0.005511 | 0.69 | 0.1699 | 1 |
| never eat dairy vs. no dairy restrictions | 0.46(0.24,0.91) | 0.025589 | 0.43(0.22,0.83) | 0.012194 | 0.1(0.02,0.55) | 0.007985 | 0.01(0,0.02) | 0.005467 | 0.63 | 0.172074 | 1 |
| never eat wheat vs. no eggs, dairy, wheat, or sugar restrictions | 0.32(0.16,0.64) | 0.001373 | 1.07(0.33,3.46) | 0.910735 | 0.16(0.05,0.46) | 0.000738 | 0.01(0,0.03) | 0.069782 | 1.08 | 0.60664 | 5 |
| never eat wheat vs. no wheat restrictions | 0.22(0.1,0.47) | 0.0001 | 1.02(0.4,2.61) | 0.968856 | 0.12(0.03,0.46) | 0.001894 | 0.01(0,0.03) | 0.110338 | 1.19 | 0.439779 | 4 |
| never eat sugar vs. no eggs, dairy, wheat, or sugar restrictions | 1.04(0.93,1.18) | 0.466308 | 1(0.85,1.18) | 0.987565 | 1.26(0.87,1.82) | 0.214077 | 0(-0.01,0) | 0.26093 | 1.05 | 0.318709 | 1 |
| never eat sugar vs. no sugar restrictions | 1.03(0.91,1.16) | 0.631458 | 0.98(0.83,1.16) | 0.815462 | 1.24(0.85,1.82) | 0.268446 | 0(-0.01,0) | 0.29472 | 1.04 | 0.441366 | 1 |
| milk type: dairy-based milk vs. never | 0.67(0.35,1.28) | 0.224572 | 0.62(0.31,1.21) | 0.162574 | 3.33(0.35,31.4) | 0.293848 | -0.01(-0.03,0) | 0.139065 | 0.66 | 0.194545 | 2 |
| milk type: any milk vs. never | 1.19(0.63,2.26) | 0.585433 | 1.2(0.64,2.28) | 0.567465 | 1.74(0.43,7.09) | 0.438814 | 0(-0.01,0.01) | 0.702918 | 1.36 | 0.295299 | 1 |
| milk type: full cream vs. never | 0.95(0.89,1.03) | 0.219111 | 0.95(0.88,1.03) | 0.232342 | 1.08(0.87,1.33) | 0.483093 | -0.01(-0.02,0) | 0.267003 | 0.95 | 0.132306 | 1 |
| milk type: full cream vs. any other | 0.78(0.54,1.12) | 0.177035 | 0.74(0.52,1.05) | 0.087736 | 0.42(0.07,2.36) | 0.32416 | 0.01(-0.01,0.02) | 0.381241 | 0.8 | 0.211226 | 5 |
| milk type: semi-skimmed vs. never | 0.71(0.45,1.13) | 0.149427 | 0.65(0.4,1.05) | 0.076612 | 1.03(0.18,5.79) | 0.971391 | -0.01(-0.02,0.01) | 0.576729 | 0.62 | 0.030601 | 2 |
| milk type: semi-skimmed vs. any other | 0.91(0.73,1.13) | 0.392739 | 0.95(0.77,1.18) | 0.652928 | 0.73(0.38,1.42) | 0.361363 | 0(-0.01,0.01) | 0.446956 | 0.91 | 0.367205 | 2 |
| milk type: skimmed vs. never | 1.16(1,1.34) | 0.049892 | 1.16(1,1.35) | 0.047435 | 1.08(0.84,1.4) | 0.539949 | 0(-0.01,0.01) | 0.771576 | 1.12 | 0.049181 | 0 |
| milk type: skimmed vs. any other | 1.16(0.95,1.42) | 0.154459 | 1.22(0.99,1.51) | 0.057429 | 1.07(0.46,2.51) | 0.870319 | 0(-0.01,0.01) | 0.816895 | 1.17 | 0.072848 | 3 |
| milk type: soy milk vs. never | 1.02(0.96,1.08) | 0.508092 | 1.02(0.95,1.08) | 0.613677 | 0.97(0.86,1.11) | 0.674114 | 0(0,0.01) | 0.402744 | 1.02 | 0.359601 | 0 |
| milk type: soy milk vs. any other | 1.4(0.88,2.24) | 0.152171 | 1.1(0.7,1.73) | 0.671245 | 1.28(0.21,7.88) | 0.793488 | 0(-0.01,0.01) | 0.857226 | 1.06 | 0.72357 | 1 |
| milk type: other milk vs. never | 1.03(0.96,1.09) | 0.445588 | 1.02(0.96,1.09) | 0.518769 | 1.04(0.93,1.15) | 0.499278 | 0(-0.01,0.01) | 0.759029 | 1.02 | 0.404986 | 0 |
| milk type: other milk vs. any other | 0.85(0.33,2.19) | 0.736053 | 0.99(0.38,2.6) | 0.991424 | 1.69(0.38,7.56) | 0.494552 | 0(-0.02,0.01) | 0.433502 | 0.99 | 0.980069 | 0 |
| spread type: all spreads vs. never | 1.02(0.81,1.28) | 0.870733 | 1.04(0.81,1.32) | 0.774265 | 0.91(0.43,1.89) | 0.791718 | 0(-0.01,0.01) | 0.690825 | 1.08 | 0.474834 | 2 |
| spread type: butter + margarine vs. never | 0.98(0.84,1.13) | 0.753049 | 0.99(0.85,1.16) | 0.936718 | 1.16(0.69,1.94) | 0.571936 | 0(-0.01,0.01) | 0.502039 | 0.99 | 0.914164 | 4 |
| spread type: any oil based spread vs. never | 1.03(0.9,1.19) | 0.635726 | 1.08(0.94,1.23) | 0.2778 | 1.26(0.67,2.36) | 0.469928 | -0.01(-0.02,0.01) | 0.350863 | 1.05 | 0.399819 | 6 |
| spread type: butter and butter-like spreads vs. oil-based spreads | 0.97(0.82,1.13) | 0.672397 | 1.03(0.86,1.23) | 0.75742 | 0.94(0.61,1.43) | 0.761293 | 0(-0.01,0.01) | 0.956708 | 0.98 | 0.753576 | 1 |
| spread type: butter and margarine spreads vs. oil-based spreads | 1.03(0.9,1.17) | 0.709724 | 1.1(0.96,1.26) | 0.191699 | 1.15(0.74,1.77) | 0.532335 | 0(-0.01,0) | 0.40713 | 1.03 | 0.67034 | 4 |
| spread type: butter vs. never | 1.01(0.91,1.13) | 0.842242 | 1(0.89,1.13) | 0.986233 | 1.16(0.81,1.66) | 0.414235 | 0(-0.01,0) | 0.334073 | 0.99 | 0.756488 | 2 |
| spread type: butter vs. any other | 0.97(0.86,1.09) | 0.596148 | 1(0.87,1.16) | 0.999463 | 1.17(0.74,1.87) | 0.503809 | 0(-0.01,0) | 0.357851 | 0.94 | 0.269692 | 8 |
| spread type: tub margarine vs. never | 0.98(0.9,1.08) | 0.699855 | 0.99(0.91,1.09) | 0.890739 | 0.98(0.69,1.38) | 0.886039 | 0(-0.01,0.01) | 0.921183 | 1.01 | 0.764477 | 3 |
| spread type: tub margarine vs. any other | 1.14(0.79,1.64) | 0.48773 | 1.16(0.79,1.7) | 0.452885 | 0.96(0.4,2.27) | 0.920907 | 0(-0.01,0.01) | 0.806502 | 1.17 | 0.31816 | 1 |
| spread type: flora + benecol vs. never | 0.94(0.86,1.03) | 0.187652 | 0.94(0.86,1.03) | 0.195261 | 0.95(0.76,1.19) | 0.685234 | 0(-0.01,0.01) | 0.896684 | 0.94 | 0.044907 | 3 |
| spread type: flora + benecol vs. any other | 1.01(0.81,1.28) | 0.909483 | 1.02(0.82,1.26) | 0.880376 | 0.81(0.57,1.14) | 0.227756 | 0(0,0.01) | 0.079333 | 1.07 | 0.438486 | 0 |
| spread type: olive oil spread vs. never | 1.07(0.96,1.21) | 0.222671 | 1.06(0.95,1.2) | 0.288424 | 1.05(0.73,1.51) | 0.780831 | 0(-0.01,0.01) | 0.797175 | 1.04 | 0.481439 | 3 |
| spread type: olive oil spread vs. any other | 0.72(0.5,1.03) | 0.075876 | 0.72(0.5,1.05) | 0.089508 | 1.15(0.55,2.43) | 0.709567 | -0.01(-0.02,0) | 0.234122 | 0.68 | 0.014333 | 1 |
| spread type: other oil-based spread vs. never | 0.93(0.84,1.04) | 0.195906 | 0.99(0.89,1.1) | 0.902749 | 0.94(0.65,1.37) | 0.758951 | 0(-0.01,0.01) | 0.907751 | 0.97 | 0.462218 | 5 |
| spread type: other oil-based spread vs. any other | 1(0.74,1.35) | 0.979981 | 0.95(0.7,1.29) | 0.743928 | 1.04(0.47,2.3) | 0.919043 | 0(-0.01,0.01) | 0.946092 | 0.95 | 0.711118 | 1 |
| spread type: low fat spread vs. never | 1.15(1.01,1.31) | 0.029396 | 1.11(0.98,1.26) | 0.106646 | 1.1(0.81,1.5) | 0.522134 | 0(-0.01,0.01) | 0.998979 | 1.05 | 0.332092 | 1 |
| spread type: low fat spread vs. any other | 1.5(0.85,2.63) | 0.158358 | 1.41(0.82,2.42) | 0.213919 | 1.91(0.62,5.89) | 0.261997 | 0(-0.01,0.01) | 0.868472 | 1.75 | 0.036892 | 1 |
| bread type: white vs. any other | 1.07(0.97,1.18) | 0.178387 | 1.07(0.94,1.22) | 0.281854 | 0.9(0.62,1.29) | 0.555914 | 0(0,0.01) | 0.266804 | 1.12 | 0.007262 | 3 |
| bread type: brown vs. any other | 0.92(0.65,1.31) | 0.652907 | 0.83(0.58,1.19) | 0.321763 | 0.96(0.43,2.17) | 0.929564 | 0(-0.01,0.01) | 0.798271 | 0.88 | 0.42369 | 1 |
| bread type: wholemeal/wholegrain vs. any other | 0.94(0.86,1.04) | 0.245791 | 0.95(0.84,1.08) | 0.44909 | 1.44(0.96,2.15) | 0.075426 | -0.01(-0.01,0) | 0.033488 | 0.9 | 0.022121 | 6 |
| bread type: white vs. wholemeal/wholegrain + brown | 1.05(0.96,1.15) | 0.264499 | 1.02(0.9,1.15) | 0.736399 | 0.94(0.67,1.31) | 0.709076 | 0(0,0.01) | 0.358651 | 1.1 | 0.017376 | 3 |
| bread type: wholemeal/wholegrain vs. white + brown | 0.91(0.83,0.99) | 0.0288 | 0.88(0.79,0.99) | 0.034828 | 1.29(0.93,1.79) | 0.125394 | 0(-0.01,0) | 0.061241 | 0.92 | 0.044044 | 5 |
| cereal type: biscuit cereal vs. any other | 1.25(0.99,1.57) | 0.060242 | 1.25(0.98,1.58) | 0.069437 | 0.84(0.51,1.41) | 0.514717 | 0.01(0,0.02) | 0.036017 | 1.34 | 0.003654 | 1 |
| cereal type: bran cereal vs. any other | 1.15(0.86,1.54) | 0.345953 | 0.98(0.74,1.3) | 0.90126 | 0.95(0.45,1.99) | 0.892396 | 0(-0.01,0.01) | 0.677905 | 1 | 0.985746 | 1 |
| cereal type: oat cereal vs. any other | 1.19(0.95,1.49) | 0.131474 | 1.23(0.98,1.54) | 0.080714 | 1.47(0.91,2.37) | 0.115859 | 0(-0.01,0) | 0.370091 | 1.2 | 0.065996 | 1 |
| cereal type: muesli vs. any other | 0.74(0.65,0.85) | 1.86E-05 | 0.83(0.71,0.97) | 0.021736 | 0.73(0.44,1.22) | 0.226377 | 0(-0.01,0.01) | 0.937246 | 0.78 | 0.000124 | 4 |
| cereal type: cornflakes/frosties vs. any other | 0.96(0.83,1.12) | 0.641597 | 0.99(0.82,1.2) | 0.928061 | 0.52(0.31,0.89) | 0.017895 | 0.01(0,0.02) | 0.035838 | 1.04 | 0.592304 | 9 |
| coffee type: decaffeinated vs. any other | 0.94(0.85,1.03) | 0.160775 | 0.93(0.83,1.05) | 0.237961 | 1.16(0.79,1.71) | 0.44271 | 0(-0.01,0) | 0.306988 | 0.92 | 0.041643 | 10 |
| coffee type: ground+instant vs. other+decaff | 0.81(0.66,1.01) | 0.056802 | 0.74(0.6,0.92) | 0.006254 | 1.29(0.79,2.08) | 0.306668 | -0.01(-0.02,0) | 0.06983 | 0.82 | 0.055688 | 1 |
| coffee type: ground vs. any other | 1.24(1,1.53) | 0.045479 | 1.3(1.04,1.61) | 0.018757 | 0.9(0.56,1.44) | 0.658076 | 0.01(0,0.01) | 0.21608 | 1.18 | 0.105235 | 1 |
| tablespoons of cooked vegetables per day | 1.11(0.99,1.25) | 0.070144 | 1.09(0.93,1.26) | 0.289619 | 0.75(0.48,1.16) | 0.195065 | 0.01(0,0.01) | 0.051648 | 1.21 | 0.000389 | 7 |
| tablespoons of raw vegetables per day | 1.06(0.96,1.17) | 0.267503 | 1.04(0.91,1.18) | 0.576392 | 1.37(0.99,1.92) | 0.060684 | 0(-0.01,0) | 0.163313 | 1.06 | 0.193245 | 4 |
| pieces of fresh fruit per day | 0.9(0.83,0.99) | 0.021526 | 0.92(0.83,1.01) | 0.089101 | 0.99(0.77,1.28) | 0.957063 | 0(0,0) | 0.454748 | 0.91 | 0.008791 | 4 |
| pieces of dried fruit per day | 0.91(0.81,1.03) | 0.151976 | 0.9(0.78,1.04) | 0.136363 | 0.97(0.61,1.54) | 0.905909 | 0(-0.01,0) | 0.731671 | 0.84 | 0.001326 | 8 |
| slices of bread per week | 0.99(0.89,1.11) | 0.910657 | 0.99(0.88,1.13) | 0.929996 | 0.99(0.69,1.42) | 0.96707 | 0(-0.01,0) | 0.905563 | 1.02 | 0.648567 | 7 |
| bowls of cereal per week | 0.84(0.76,0.92) | 0.000412 | 0.81(0.71,0.91) | 0.000717 | 0.72(0.5,1.03) | 0.073379 | 0(0,0.01) | 0.333989 | 0.84 | 0.000161 | 5 |
| cups of tea per day | 1.14(1.03,1.27) | 0.010973 | 1.07(0.98,1.17) | 0.14873 | 1.04(0.81,1.33) | 0.762238 | 0(0,0) | 0.773307 | 1.04 | 0.276209 | 6 |
| cups of coffee per day | 1.1(0.98,1.23) | 0.116796 | 1.02(0.94,1.11) | 0.592993 | 0.99(0.82,1.2) | 0.939984 | 0(0,0) | 0.573178 | 0.94 | 0.180323 | 6 |
| glasses of water per day | 0.96(0.88,1.04) | 0.294115 | 0.94(0.86,1.03) | 0.16486 | 0.88(0.7,1.11) | 0.276065 | 0(0,0) | 0.234591 | 1.01 | 0.874757 | 11 |
| champagne/white wine glasses per month | 0.84(0.73,0.97) | 0.016118 | 0.88(0.75,1.04) | 0.132157 | 1.03(0.64,1.67) | 0.902576 | 0(-0.01,0) | 0.537136 | 0.86 | 0.011901 | 4 |
| red wine glasses per month | 0.78(0.7,0.86) | 1.98E-06 | 0.81(0.71,0.92) | 0.00099 | 0.85(0.55,1.31) | 0.459265 | 0(-0.01,0.01) | 0.876919 | 0.81 | 8.38E-06 | 10 |
| beer/cider glasses per month | 0.95(0.84,1.07) | 0.383982 | 0.91(0.79,1.04) | 0.17566 | 0.76(0.52,1.12) | 0.169325 | 0(0,0.01) | 0.140837 | 0.95 | 0.336532 | 7 |
| spirits measures per month | 1.02(0.85,1.22) | 0.843972 | 0.99(0.81,1.2) | 0.888436 | 1.16(0.74,1.83) | 0.522573 | 0(-0.01,0) | 0.336606 | 1.03 | 0.710917 | 2 |
| fortwine glasses per month | 0.79(0.59,1.07) | 0.133207 | 0.81(0.61,1.09) | 0.162616 | 1.44(0.75,2.79) | 0.276937 | -0.01(-0.02,0) | 0.043554 | 0.77 | 0.079684 | 1 |
| other alcohol glasses per month | 1.13(0.88,1.44) | 0.342287 | 1.2(0.95,1.53) | 0.125164 | 0.84(0.53,1.33) | 0.449745 | 0(-0.01,0.02) | 0.406281 | 1 | 0.994174 | 0 |
| total drinks of alcohol per month | 0.89(0.82,0.97) | 0.005341 | 0.85(0.77,0.94) | 0.000919 | 0.98(0.78,1.23) | 0.845113 | 0(0,0) | 0.493522 | 0.9 | 0.001714 | 9 |
| overall oily fish intake | 1.04(0.95,1.14) | 0.397629 | 1.01(0.9,1.14) | 0.879505 | 1.27(0.86,1.86) | 0.228816 | 0(-0.01,0) | 0.326933 | 0.99 | 0.865372 | 10 |
| overall non-oily fish intake | 1.01(0.87,1.17) | 0.89766 | 0.99(0.83,1.17) | 0.90794 | 1.03(0.64,1.65) | 0.908016 | 0(-0.01,0.01) | 0.932904 | 1 | 0.945334 | 1 |
| overall processed meat intake | 1(0.89,1.13) | 1 | 1(0.86,1.15) | 0.973874 | 0.97(0.62,1.51) | 0.891527 | 0(-0.01,0.01) | 0.891463 | 0.95 | 0.400552 | 2 |
| overall poultry intake | 1.16(0.98,1.36) | 0.086102 | 1.18(0.98,1.41) | 0.07681 | 0.95(0.57,1.6) | 0.85701 | 0(0,0.01) | 0.543073 | 1.21 | 0.009272 | 3 |
| overall beef intake | 0.84(0.73,0.95) | 0.006392 | 0.89(0.77,1.04) | 0.155278 | 1.05(0.7,1.59) | 0.806697 | 0(-0.01,0) | 0.119083 | 0.8 | 0.000135 | 3 |
| overall lamb/mutton intake | 0.93(0.83,1.04) | 0.202235 | 0.95(0.82,1.09) | 0.44067 | 1.03(0.65,1.62) | 0.897513 | 0(-0.01,0) | 0.588474 | 0.89 | 0.033549 | 6 |
| overall pork intake | 0.88(0.75,1.02) | 0.085164 | 0.95(0.79,1.13) | 0.532167 | 1.16(0.73,1.85) | 0.523071 | 0(-0.01,0) | 0.17661 | 0.92 | 0.194118 | 2 |
| overall cheese intake | 0.88(0.81,0.96) | 0.004039 | 0.89(0.78,1) | 0.058957 | 0.79(0.6,1.03) | 0.085654 | 0(0,0.01) | 0.354172 | 0.87 | 0.000463 | 3 |
| frequency of adding salt to food | 1.12(1.03,1.21) | 0.006199 | 1.1(0.99,1.22) | 0.070486 | 1.16(0.89,1.52) | 0.265978 | 0(0,0) | 0.942832 | 1.1 | 0.004397 | 6 |
| temperature of hot drinks | 0.86(0.79,0.95) | 0.002065 | 0.92(0.81,1.04) | 0.171783 | 0.9(0.64,1.28) | 0.554884 | 0(0,0) | 0.949836 | 0.9 | 0.008394 | 8 |
| overall alcohol intake | 0.89(0.82,0.96) | 0.004159 | 0.85(0.78,0.94) | 0.001106 | 0.82(0.65,1.04) | 0.099801 | 0(0,0) | 0.483977 | 0.85 | 7.96E-07 | 11 |
| among current drinkers, drinks usually with meals: yes, it varies, no | 0.79(0.72,0.88) | 8.22E-06 | 0.83(0.73,0.95) | 0.006683 | 0.96(0.67,1.38) | 0.813546 | 0(-0.01,0) | 0.286945 | 0.81 | 3.96E-06 | 4 |
| milk type: skimmed, semi-skimmed, full cream (QT) | 0.82(0.69,0.98) | 0.028845 | 0.82(0.69,0.97) | 0.019159 | 0.85(0.43,1.67) | 0.633119 | 0(-0.01,0.01) | 0.848676 | 0.76 | 0.000561 | 7 |

MR: mendelian randomization; MRPRESSO: MR pleiotropy residual sum and outlier; OR: odd ratio; CI: confidence interval; No.: number.

**Table S29. Leave-one-out analysis of association between genetically predicted dietary habits and major depression disorder.**

| **Exposure** | **IVW Estimate**  **[Min, Max] ^a^** | **P value [Min, Max] ^b^** |
| --- | --- | --- |
| alcohol drinker status: current + former vs. never | [1,1.17] | [0.365562577705616,0.976017652235445] |
| alcohol drinker status: current vs. never | [0.99,1.15] | [0.415540056323778,0.925623179534817] |
| among current drinkers, drinks usually with meals: yes + it varies vs. no | [0.7,0.73] | [2.83144491445076e-09,1.25896131785529e-07] |
| among current drinkers, drinks usually with meals: yes vs. no | [0.79,0.81] | [4.11385046375678e-12,3.15035825418663e-10] |
| never eat eggs vs. no eggs, dairy, wheat, or sugar restrictions | [1.54,1.91] | [0.00636777684169182,0.0681981834025388] |
| never eat eggs vs. no eggs restrictions | [1.3,1.93] | [0.0780675723369815,0.478214837495329] |
| never eat dairy vs. no eggs, dairy, wheat, or sugar restrictions | [0.46,0.72] | [0.000357837211595815,0.391074213830153] |
| never eat dairy vs. no dairy restrictions | [0.38,0.66] | [0.00034721184138517,0.390613182408498] |
| never eat wheat vs. no eggs, dairy, wheat, or sugar restrictions | [0.33,0.5] | [0.000704941987711929,0.0275801005318842] |
| never eat wheat vs. no wheat restrictions | [0.27,0.44] | [0.000974572231473791,0.0327549664105924] |
| never eat sugar vs. no eggs, dairy, wheat, or sugar restrictions | [1.02,1.05] | [0.316710941952299,0.728231884867355] |
| never eat sugar vs. no sugar restrictions | [1.01,1.04] | [0.439834642213871,0.897627775255436] |
| milk type: dairy-based milk vs. never | [0.53,0.82] | [0.0609582046670271,0.576969715207066] |
| milk type: any milk vs. never | [1.13,1.51] | [0.137712385020962,0.619447045193097] |
| milk type: full cream vs. never | [0.95,0.98] | [0.118650233518406,0.555255825152295] |
| milk type: full cream vs. any other | [0.78,1.01] | [0.289477036763273,0.962743716309586] |
| milk type: semi-skimmed vs. never | [0.54,0.75] | [0.00469394323790336,0.225551466178751] |
| milk type: semi-skimmed vs. any other | [0.87,0.97] | [0.208710465009385,0.825052974150536] |
| milk type: skimmed vs. never | [1.1,1.16] | [0.0041692088771336,0.0767413760475311] |
| milk type: skimmed vs. any other | [1.07,1.24] | [0.0829546471437163,0.459111182996575] |
| milk type: soy milk vs. never | [1.01,1.03] | [0.153913405792852,0.554535853032092] |
| milk type: soy milk vs. any other | [1.06,1.58] | [0.141153182616279,0.734366989041055] |
| milk type: other milk vs. never | [1.01,1.04] | [0.135240289759554,0.653266638652594] |
| milk type: other milk vs. any other | [0.79,1.16] | [0.504453914474906,0.997101617012332] |
| spread type: all spreads vs. never | [1,1.12] | [0.31509225799703,0.974060780176954] |
| spread type: butter + margarine vs. never | [0.95,1.02] | [0.533040411309876,0.997900290831355] |
| spread type: any oil based spread vs. never | [0.91,1.02] | [0.366412005924991,0.829015796614297] |
| spread type: butter and butter-like spreads vs. oil-based spreads | [0.92,0.98] | [0.304422401650647,0.751890254544295] |
| spread type: butter and margarine spreads vs. oil-based spreads | [0.95,1] | [0.442324579774408,0.983528514492199] |
| spread type: butter vs. never | [0.96,1.01] | [0.382006249072033,0.924172472875997] |
| spread type: butter vs. any other | [0.93,0.97] | [0.290224587824265,0.650115586221106] |
| spread type: tub margarine vs. never | [0.97,1.05] | [0.22384149086773,0.974931034077762] |
| spread type: tub margarine vs. any other | [1,1.17] | [0.30878250628232,0.994672853525069] |
| spread type: flora + benecol vs. never | [0.94,0.99] | [0.159128321143208,0.765992003963491] |
| spread type: flora + benecol vs. any other | [1.04,1.11] | [0.171201550053926,0.638743847197002] |
| spread type: olive oil spread vs. never | [0.98,1.04] | [0.528949699587011,0.994718296420864] |
| spread type: olive oil spread vs. any other | [0.68,0.82] | [0.0051968581449947,0.228334726164376] |
| spread type: other oil-based spread vs. never | [0.9,0.97] | [0.114423993578259,0.601628116723786] |
| spread type: other oil-based spread vs. any other | [0.95,1.07] | [0.605171859399212,0.989341661961038] |
| spread type: low fat spread vs. never | [1.05,1.14] | [0.0183872738795362,0.308066336412429] |
| spread type: low fat spread vs. any other | [1.58,1.91] | [0.00728907448323877,0.0597792563846346] |
| bread type: white vs. any other | [1.08,1.12] | [0.0088554886347254,0.0969438784799587] |
| bread type: brown vs. any other | [0.82,0.94] | [0.198863076923354,0.697043419598719] |
| bread type: wholemeal/wholegrain vs. any other | [0.93,0.97] | [0.124667524675005,0.52290781005365] |
| bread type: white vs. wholemeal/wholegrain + brown | [1.08,1.12] | [0.00631013862954594,0.0787562606661262] |
| bread type: wholemeal/wholegrain vs. white + brown | [0.93,0.97] | [0.0981505771431558,0.429752431498013] |
| cereal type: biscuit cereal vs. any other | [1.31,1.42] | [0.000600233303441702,0.00424188398753263] |
| cereal type: bran cereal vs. any other | [1,1.15] | [0.334455073814826,0.985534821100434] |
| cereal type: oat cereal vs. any other | [1.15,1.24] | [0.0157792892524585,0.146103069186711] |
| cereal type: muesli vs. any other | [0.73,0.76] | [5.23854348138611e-06,9.13957109403703e-05] |
| cereal type: cornflakes/frosties vs. any other | [0.92,0.99] | [0.299868831911446,0.930412402095221] |
| coffee type: decaffeinated vs. any other | [0.93,0.97] | [0.171213435026204,0.560478149041613] |
| coffee type: ground+instant vs. other+decaff | [0.82,0.89] | [0.0470636278652107,0.286951628067711] |
| coffee type: ground vs. any other | [1.12,1.22] | [0.0300134125939843,0.244548845147882] |
| tablespoons of cooked vegetables per day | [1.12,1.16] | [0.0104728760483846,0.0405463809321226] |
| tablespoons of raw vegetables per day | [1.07,1.11] | [0.0356160886515993,0.130643090565647] |
| pieces of fresh fruit per day | [0.89,0.91] | [0.00187788409644578,0.0135304863432805] |
| pieces of dried fruit per day | [0.87,0.92] | [0.0197356365042728,0.19977024418019] |
| slices of bread per week | [0.96,0.99] | [0.445281075803294,0.836980605781244] |
| bowls of cereal per week | [0.84,0.86] | [0.000152936271743646,0.00338907746062714] |
| cups of tea per day | [1.06,1.09] | [0.055605711254652,0.207233258686193] |
| cups of coffee per day | [0.91,0.96] | [0.0817419843249401,0.3614225463549] |
| glasses of water per day | [1,1.02] | [0.689583303350565,0.997526322905842] |
| champagne/white wine glasses per month | [0.86,0.93] | [0.0317304898982321,0.278044849776548] |
| red wine glasses per month | [0.8,0.83] | [7.40507160890633e-05,0.000991861688820336] |
| beer/cider glasses per month | [0.99,1.02] | [0.723938679712614,0.999593524331191] |
| spirits measures per month | [0.93,0.99] | [0.392408669076911,0.879369939702435] |
| fortwine glasses per month | [0.78,0.87] | [0.0253002104491489,0.263622698865931] |
| other alcohol glasses per month | [0.96,1.06] | [0.543620213099697,0.953103189487869] |
| total drinks of alcohol per month | [0.9,0.92] | [0.00398034310131471,0.0222014882319118] |
| overall oily fish intake | [1.03,1.06] | [0.208152401462267,0.54287939337633] |
| overall non-oily fish intake | [1,1.06] | [0.392645648527943,0.945140495111971] |
| overall processed meat intake | [0.92,0.97] | [0.194686044737789,0.658099891640107] |
| overall poultry intake | [1.09,1.15] | [0.0715499664791727,0.301186126873393] |
| overall beef intake | [0.75,0.81] | [9.11586653348282e-06,0.000545762165334646] |
| overall lamb/mutton intake | [0.9,0.94] | [0.0773780139342677,0.288931840060499] |
| overall pork intake | [0.84,0.9] | [0.0345989402060929,0.118204284838658] |
| overall cheese intake | [0.88,0.9] | [0.0016304776386493,0.0083341664790542] |
| frequency of adding salt to food | [1.14,1.16] | [5.42693182192166e-05,0.000379008407975759] |
| temperature of hot drinks | [0.88,0.9] | [0.0044930814969474,0.0250150350474838] |
| overall alcohol intake | [0.88,0.9] | [0.000344793750565031,0.00251975619370847] |
| among current drinkers, drinks usually with meals: yes, it varies, no | [0.78,0.8] | [8.84102998643438e-08,2.44033479656598e-06] |
| milk type: skimmed, semi-skimmed, full cream (QT) | [0.77,0.86] | [0.016084194272921,0.142439221663476] |

**^a^** the minimum value and maximum value of inverse variance weighted estimate;

**^b^** the minimum value and maximum value of P value;

IVW: inverse variance weighted.

**Table S30. Associations between genetically predicted dietary habits and anxiety in sensitivity analysis.**

| **Exposure** | **Weighted median** | | **Mode-based** | | **MR-Egger** | | | | **MRPRESSO** | | |
| --- | --- | --- | --- | --- | --- | --- | --- | --- | --- | --- | --- |
|  | **OR (95% CI)** | **P value** | **OR (95% CI)** | **P value** | **OR (95% CI)** | **P value** | **Intercept** | **P value** | **OR** | **P value** | **No. of outliers** |
| alcohol drinker status: current + former vs. never | 0.16(0.02,1.71) | 0.130243 | 0.13(0.01,1.43) | 0.096241 | 0.02(0,1.99) | 0.097459 | 0.02(-0.01,0.05) | 0.202753 | 0.33 | 0.225048 | 0 |
| alcohol drinker status: current vs. never | 0.4(0.04,3.98) | 0.433607 | 0.25(0.02,2.46) | 0.232649 | 0.03(0,3.22) | 0.144967 | 0.02(-0.01,0.05) | 0.236893 | 0.43 | 0.346199 | 0 |
| among current drinkers, drinks usually with meals: yes + it varies vs. no | 0.33(0.18,0.62) | 0.000454 | 0.37(0.17,0.83) | 0.015422 | 0.37(0.05,2.78) | 0.333419 | 0(-0.03,0.02) | 0.892604 | 0.32 | 1.14E-06 | 0 |
| among current drinkers, drinks usually with meals: yes vs. no | 0.51(0.35,0.74) | 0.000405 | 0.48(0.3,0.79) | 0.003483 | 0.52(0.16,1.68) | 0.273703 | 0(-0.02,0.02) | 0.823605 | 0.57 | 0.000109 | 1 |
| never eat eggs vs. no eggs, dairy, wheat, or sugar restrictions | 1.06(0.02,45.45) | 0.974185 | 0.37(0.01,14.47) | 0.593357 | 0(0,28.61) | 0.219376 | 0.05(-0.02,0.12) | 0.169739 | 1.33 | 0.867092 | 0 |
| never eat eggs vs. no eggs restrictions | 0.03(0,6.56) | 0.202437 | 0.11(0,18.08) | 0.394387 | 0.01(0,232.43) | 0.350566 | 0.02(-0.04,0.09) | 0.538998 | 0.14 | 0.264184 | 0 |
| never eat dairy vs. no eggs, dairy, wheat, or sugar restrictions | 1(0.04,26.05) | 0.9989 | 0.67(0.03,13.39) | 0.793175 | 0.36(0,39.28) | 0.671342 | 0(-0.03,0.04) | 0.881251 | 0.49 | 0.499018 | 0 |
| never eat dairy vs. no dairy restrictions | 1(0.02,56.26) | 0.999798 | 0.54(0.01,21.78) | 0.746799 | 0.26(0,78.75) | 0.643869 | 0(-0.03,0.04) | 0.877209 | 0.38 | 0.452403 | 0 |
| never eat wheat vs. no eggs, dairy, wheat, or sugar restrictions | 0.1(0.02,0.61) | 0.012335 | 0.1(0.02,0.44) | 0.002301 | 0.01(0,0.23) | 0.003996 | 0.06(0.01,0.1) | 0.018087 | 0.19 | 0.070702 | 1 |
| never eat wheat vs. no wheat restrictions | 0.03(0,0.35) | 0.005136 | 0.05(0.01,0.39) | 0.003892 | 0.02(0,1.02) | 0.051366 | 0.03(-0.02,0.07) | 0.260763 | 0.14 | 0.089476 | 0 |
| never eat sugar vs. no eggs, dairy, wheat, or sugar restrictions | 1.59(0.84,3.02) | 0.151912 | 1.33(0.6,2.94) | 0.485275 | 1.48(0.2,10.89) | 0.702077 | 0(-0.02,0.03) | 0.854351 | 1.64 | 0.04011 | 1 |
| never eat sugar vs. no sugar restrictions | 1.29(0.67,2.48) | 0.445635 | 1.03(0.45,2.37) | 0.945911 | 0.73(0.11,4.86) | 0.744913 | 0.01(-0.01,0.03) | 0.37993 | 1.53 | 0.078448 | 1 |
| milk type: dairy-based milk vs. never | 1.27(0.05,34.15) | 0.887105 | 0.35(0.01,8.65) | 0.517667 | 84191.92(7.07,1002845149.21) | 0.017867 | -0.09(-0.16,-0.02) | 0.009193 | 0.49 | 0.619267 | 0 |
| milk type: any milk vs. never | 1.81(0.06,59.39) | 0.739619 | 4.44(0.16,122.58) | 0.378399 | 0.13(0,1449.94) | 0.667216 | 0.02(-0.05,0.09) | 0.616085 | 1.27 | 0.863487 | 0 |
| milk type: full cream vs. never | 0.86(0.56,1.32) | 0.48089 | 0.75(0.48,1.17) | 0.203729 | 0.97(0.24,3.9) | 0.970655 | 0(-0.06,0.06) | 0.910479 | 0.9 | 0.577382 | 0 |
| milk type: full cream vs. any other | 0.25(0.04,1.44) | 0.120472 | 0.23(0.04,1.35) | 0.103826 | 0.05(0,14.93) | 0.298375 | 0.02(-0.03,0.07) | 0.494189 | 0.33 | 0.108252 | 0 |
| milk type: semi-skimmed vs. never | 1.15(0.1,13.06) | 0.912063 | 0.43(0.04,4.3) | 0.472514 | 495.53(0.19,1277073.32) | 0.121497 | -0.08(-0.16,0.01) | 0.075016 | 0.49 | 0.4924 | 0 |
| milk type: semi-skimmed vs. any other | 0.36(0.11,1.16) | 0.087844 | 0.32(0.1,1.09) | 0.069135 | 0.09(0.01,0.89) | 0.039781 | 0.02(-0.02,0.05) | 0.283251 | 0.29 | 0.008864 | 0 |
| milk type: skimmed vs. never | 1.13(0.49,2.6) | 0.771405 | 0.85(0.37,1.95) | 0.693922 | 1.34(0.25,7.33) | 0.732728 | -0.01(-0.05,0.03) | 0.70786 | 0.99 | 0.982637 | 0 |
| milk type: skimmed vs. any other | 3.01(0.97,9.39) | 0.056911 | 4.36(1.32,14.41) | 0.01568 | 2.42(0.09,65.82) | 0.600501 | 0(-0.04,0.05) | 0.862014 | 3.21 | 0.010071 | 0 |
| milk type: soy milk vs. never | 1.03(0.72,1.47) | 0.862058 | 1.04(0.72,1.49) | 0.850048 | 1.96(0.94,4.09) | 0.07414 | -0.04(-0.08,0.01) | 0.094211 | 1.08 | 0.54692 | 0 |
| milk type: soy milk vs. any other | 0.18(0.01,3.39) | 0.249857 | 0.1(0,2.28) | 0.150512 | 0.59(0,1598.87) | 0.896509 | 0.01(-0.05,0.06) | 0.858421 | 1.17 | 0.893694 | 0 |
| milk type: other milk vs. never | 1.52(1.03,2.23) | 0.034528 | 1.5(1.02,2.21) | 0.039209 | 2.08(1.14,3.8) | 0.017389 | -0.04(-0.09,0.01) | 0.155792 | 1.42 | 0.008064 | 0 |
| milk type: other milk vs. any other | 51.09(0.06,45587.51) | 0.256439 | 87.29(0.11,72511.38) | 0.192556 | 2.02(0,7546950.78) | 0.927476 | 0.02(-0.07,0.11) | 0.699762 | 32.99 | 0.161522 | 0 |
| spread type: all spreads vs. never | 1.28(0.35,4.66) | 0.711165 | 1.47(0.36,5.99) | 0.593271 | 1.94(0.06,68.18) | 0.714624 | 0(-0.04,0.03) | 0.883834 | 1.5 | 0.409916 | 0 |
| spread type: butter + margarine vs. never | 1.23(0.56,2.71) | 0.608056 | 1.24(0.53,2.92) | 0.624113 | 3.81(0.39,37.44) | 0.250998 | -0.02(-0.05,0.01) | 0.243524 | 1.03 | 0.933651 | 0 |
| spread type: any oil based spread vs. never | 1.18(0.58,2.41) | 0.655774 | 1.11(0.52,2.35) | 0.794921 | 3.06(0.45,20.95) | 0.253792 | -0.02(-0.06,0.02) | 0.315099 | 1.19 | 0.481132 | 0 |
| spread type: butter and butter-like spreads vs. oil-based spreads | 0.85(0.35,2.1) | 0.728789 | 0.96(0.35,2.6) | 0.929152 | 0.55(0.06,5.38) | 0.608137 | 0.01(-0.03,0.04) | 0.693114 | 0.85 | 0.645076 | 0 |
| spread type: butter and margarine spreads vs. oil-based spreads | 0.86(0.42,1.75) | 0.677735 | 0.96(0.44,2.11) | 0.921772 | 1.5(0.27,8.5) | 0.645272 | -0.01(-0.04,0.02) | 0.558307 | 0.92 | 0.733912 | 0 |
| spread type: butter vs. never | 1.29(0.69,2.39) | 0.425809 | 1.39(0.7,2.75) | 0.345275 | 2.1(0.33,13.46) | 0.435548 | -0.01(-0.05,0.02) | 0.408864 | 0.98 | 0.939315 | 0 |
| spread type: butter vs. any other | 0.6(0.33,1.1) | 0.100435 | 0.57(0.27,1.24) | 0.156294 | 1.83(0.32,10.44) | 0.494875 | -0.01(-0.04,0.01) | 0.303925 | 0.76 | 0.211443 | 0 |
| spread type: tub margarine vs. never | 1.2(0.71,2.05) | 0.494131 | 1.12(0.64,1.98) | 0.686987 | 1.73(0.48,6.27) | 0.405754 | -0.02(-0.06,0.03) | 0.436052 | 1.07 | 0.771523 | 0 |
| spread type: tub margarine vs. any other | 1.38(0.17,11.56) | 0.765696 | 1.3(0.16,10.66) | 0.808439 | 2.17(0.01,421.94) | 0.773366 | 0(-0.05,0.04) | 0.86764 | 1.43 | 0.710601 | 1 |
| spread type: flora + benecol vs. never | 1.68(1,2.81) | 0.049942 | 1.79(1.06,3.01) | 0.028036 | 0.82(0.31,2.21) | 0.696963 | 0.02(-0.02,0.05) | 0.351608 | 1.27 | 0.182736 | 0 |
| spread type: flora + benecol vs. any other | 0.65(0.17,2.5) | 0.525826 | 0.93(0.29,3) | 0.899993 | 1.54(0.2,11.57) | 0.676632 | 0(-0.03,0.02) | 0.826579 | 1.26 | 0.620029 | 0 |
| spread type: olive oil spread vs. never | 1.15(0.63,2.11) | 0.651494 | 1.15(0.63,2.09) | 0.644679 | 1.72(0.26,11.46) | 0.573515 | -0.01(-0.07,0.04) | 0.642235 | 1.12 | 0.662549 | 0 |
| spread type: olive oil spread vs. any other | 6.48(0.67,62.76) | 0.106479 | 4.96(0.57,42.79) | 0.145554 | 4.5(0.02,1022) | 0.586761 | -0.01(-0.08,0.06) | 0.762455 | 4.82 | 0.106314 | 1 |
| spread type: other oil-based spread vs. never | 1.16(0.67,1.99) | 0.59849 | 1.22(0.67,2.19) | 0.516538 | 0.6(0.14,2.49) | 0.479912 | 0.01(-0.02,0.05) | 0.460755 | 1 | 0.993492 | 0 |
| spread type: other oil-based spread vs. any other | 0.3(0.07,1.35) | 0.117004 | 0.44(0.09,2.04) | 0.290881 | 1.22(0.04,38.99) | 0.912002 | -0.01(-0.05,0.03) | 0.583916 | 0.48 | 0.184311 | 0 |
| spread type: low fat spread vs. never | 1.15(0.59,2.22) | 0.680208 | 1.11(0.57,2.18) | 0.7547 | 1.85(0.5,6.85) | 0.357609 | -0.02(-0.07,0.03) | 0.393111 | 1.09 | 0.574549 | 0 |
| spread type: low fat spread vs. any other | 2.2(0.1,47.03) | 0.613274 | 1.16(0.06,21.87) | 0.923341 | 2.87(0,3953.53) | 0.774676 | 0(-0.06,0.06) | 0.944206 | 2.25 | 0.49236 | 0 |
| bread type: white vs. any other | 1.4(0.84,2.33) | 0.192622 | 1.22(0.6,2.47) | 0.588444 | 2.09(0.41,10.67) | 0.375453 | -0.01(-0.03,0.02) | 0.574395 | 1.33 | 0.160997 | 1 |
| bread type: brown vs. any other | 1.73(0.21,14.32) | 0.610831 | 1.86(0.25,13.62) | 0.540445 | 0.08(0,23.63) | 0.386744 | 0.03(-0.03,0.09) | 0.350427 | 1.05 | 0.963352 | 1 |
| bread type: wholemeal/wholegrain vs. any other | 0.89(0.54,1.46) | 0.636576 | 0.94(0.5,1.79) | 0.859582 | 2.71(0.53,13.99) | 0.233023 | -0.02(-0.04,0.01) | 0.183474 | 0.92 | 0.663423 | 1 |
| bread type: white vs. wholemeal/wholegrain + brown | 1.88(1.18,2.99) | 0.007642 | 1.78(0.93,3.41) | 0.084089 | 1.53(0.38,6.11) | 0.548327 | 0(-0.02,0.02) | 0.986793 | 1.55 | 0.015847 | 1 |
| bread type: wholemeal/wholegrain vs. white + brown | 0.88(0.55,1.39) | 0.572721 | 0.9(0.49,1.66) | 0.728643 | 1.9(0.46,7.83) | 0.375729 | -0.01(-0.03,0.01) | 0.239749 | 0.83 | 0.296738 | 1 |
| cereal type: biscuit cereal vs. any other | 1.59(0.4,6.29) | 0.50575 | 1.7(0.44,6.57) | 0.438612 | 0.8(0.03,19.31) | 0.891242 | 0.01(-0.04,0.06) | 0.770832 | 1.24 | 0.711764 | 0 |
| cereal type: bran cereal vs. any other | 0.66(0.11,3.81) | 0.638577 | 0.88(0.16,4.98) | 0.887845 | 2.34(0.02,325.58) | 0.735797 | -0.01(-0.08,0.05) | 0.651736 | 1.21 | 0.798156 | 1 |
| cereal type: oat cereal vs. any other | 1.97(0.59,6.62) | 0.271107 | 1.31(0.41,4.17) | 0.64509 | 1.61(0.14,17.97) | 0.700762 | 0(-0.04,0.04) | 0.849269 | 2 | 0.094393 | 0 |
| cereal type: muesli vs. any other | 0.6(0.31,1.16) | 0.128335 | 0.78(0.35,1.72) | 0.542545 | 1.13(0.19,6.63) | 0.890901 | -0.01(-0.04,0.01) | 0.415917 | 0.56 | 0.01922 | 0 |
| cereal type: cornflakes/frosties vs. any other | 0.55(0.26,1.17) | 0.119697 | 0.49(0.21,1.1) | 0.082736 | 0.57(0.09,3.77) | 0.560846 | 0(-0.03,0.02) | 0.795242 | 0.45 | 0.006195 | 0 |
| coffee type: decaffeinated vs. any other | 0.79(0.49,1.27) | 0.334301 | 0.81(0.45,1.45) | 0.473233 | 0.57(0.14,2.28) | 0.430234 | 0(-0.02,0.02) | 0.680788 | 0.76 | 0.107944 | 0 |
| coffee type: ground+instant vs. other+decaff | 0.77(0.26,2.31) | 0.639276 | 0.78(0.25,2.47) | 0.675596 | 3.86(0.52,28.76) | 0.187147 | -0.03(-0.07,0) | 0.060844 | 0.66 | 0.306153 | 0 |
| coffee type: ground vs. any other | 0.84(0.24,2.97) | 0.792278 | 0.67(0.18,2.55) | 0.560597 | 0.65(0.05,8.51) | 0.743343 | 0(-0.04,0.04) | 0.979826 | 0.67 | 0.443626 | 0 |
| tablespoons of cooked vegetables per day | 1.14(0.62,2.1) | 0.663232 | 1.35(0.62,2.98) | 0.450901 | 6.82(0.86,54.06) | 0.068947 | -0.02(-0.05,0) | 0.110831 | 1.41 | 0.144458 | 1 |
| tablespoons of raw vegetables per day | 1.39(0.8,2.4) | 0.245941 | 1.78(0.88,3.6) | 0.110635 | 1.64(0.36,7.45) | 0.52507 | 0(-0.02,0.02) | 0.689114 | 1.21 | 0.350646 | 0 |
| pieces of fresh fruit per day | 1.3(0.87,1.95) | 0.192488 | 1.6(0.98,2.6) | 0.059566 | 1.86(0.66,5.19) | 0.238458 | -0.01(-0.02,0) | 0.133759 | 0.9 | 0.453598 | 1 |
| pieces of dried fruit per day | 1.2(0.64,2.24) | 0.565076 | 1.24(0.58,2.64) | 0.578985 | 1.26(0.16,10.23) | 0.828815 | -0.01(-0.03,0.02) | 0.638324 | 0.82 | 0.45016 | 1 |
| slices of bread per week | 1.03(0.57,1.85) | 0.923651 | 1.04(0.49,2.2) | 0.90987 | 0.88(0.21,3.67) | 0.857172 | 0(-0.02,0.02) | 0.719393 | 1.13 | 0.576932 | 0 |
| bowls of cereal per week | 0.5(0.3,0.82) | 0.005963 | 0.53(0.29,0.98) | 0.041361 | 0.97(0.29,3.31) | 0.967586 | -0.01(-0.02,0.01) | 0.349239 | 0.56 | 0.00064 | 0 |
| cups of tea per day | 1(0.57,1.78) | 0.986728 | 1.08(0.65,1.78) | 0.775563 | 1.99(0.74,5.33) | 0.171433 | -0.01(-0.02,0) | 0.118295 | 1 | 0.991373 | 1 |
| cups of coffee per day | 0.99(0.56,1.75) | 0.961839 | 1.07(0.69,1.66) | 0.75047 | 0.91(0.49,1.72) | 0.781185 | 0(-0.01,0.01) | 0.794984 | 0.85 | 0.304217 | 0 |
| glasses of water per day | 1.02(0.64,1.63) | 0.925947 | 1.08(0.65,1.78) | 0.761442 | 0.83(0.33,2.12) | 0.704416 | 0.01(-0.01,0.02) | 0.301332 | 1.3 | 0.068028 | 1 |
| champagne/white wine glasses per month | 0.54(0.25,1.16) | 0.116434 | 0.36(0.15,0.86) | 0.021235 | 0.54(0.1,3.03) | 0.4842 | 0(-0.02,0.03) | 0.907006 | 0.6 | 0.087303 | 1 |
| red wine glasses per month | 0.49(0.29,0.85) | 0.010199 | 0.5(0.27,0.93) | 0.027228 | 0.47(0.16,1.37) | 0.168008 | 0(-0.01,0.02) | 0.670932 | 0.59 | 0.003202 | 0 |
| beer/cider glasses per month | 0.57(0.32,0.99) | 0.047292 | 0.49(0.27,0.88) | 0.0174 | 0.27(0.09,0.84) | 0.023634 | 0.02(0,0.04) | 0.019847 | 0.96 | 0.832682 | 0 |
| spirits measures per month | 0.95(0.33,2.7) | 0.921704 | 0.75(0.23,2.39) | 0.621055 | 2.11(0.23,19.79) | 0.513152 | -0.01(-0.04,0.02) | 0.44787 | 0.94 | 0.86494 | 0 |
| fortwine glasses per month | 1.35(0.27,6.68) | 0.710649 | 1.45(0.28,7.53) | 0.655197 | 0.13(0.01,2.17) | 0.156186 | 0.04(-0.01,0.08) | 0.103951 | 1.09 | 0.874809 | 0 |
| other alcohol glasses per month | 1.16(0.18,7.39) | 0.873749 | 1.23(0.22,6.76) | 0.811894 | 0.01(0,1.5) | 0.072635 | 0.09(-0.02,0.19) | 0.118647 | 0.44 | 0.427039 | 1 |
| total drinks of alcohol per month | 0.66(0.44,1.01) | 0.05685 | 0.69(0.46,1.04) | 0.077167 | 0.69(0.36,1.32) | 0.257308 | 0(0,0.01) | 0.342596 | 0.88 | 0.360339 | 1 |
| overall oily fish intake | 1.17(0.74,1.86) | 0.500478 | 1.11(0.61,2.03) | 0.730707 | 0.85(0.22,3.27) | 0.814579 | 0(-0.01,0.02) | 0.7308 | 1.12 | 0.501633 | 1 |
| overall non-oily fish intake | 0.9(0.39,2.09) | 0.80313 | 0.88(0.34,2.28) | 0.798148 | 0.48(0.05,4.37) | 0.517411 | 0.01(-0.02,0.04) | 0.552059 | 0.92 | 0.787366 | 1 |
| overall processed meat intake | 0.66(0.34,1.27) | 0.214408 | 0.67(0.29,1.54) | 0.343001 | 0.97(0.15,6.13) | 0.970795 | 0(-0.03,0.02) | 0.796021 | 0.76 | 0.293112 | 1 |
| overall poultry intake | 0.61(0.24,1.55) | 0.300663 | 0.5(0.17,1.45) | 0.20298 | 0.61(0.05,8.1) | 0.710416 | 0(-0.03,0.04) | 0.860209 | 0.77 | 0.44529 | 0 |
| overall beef intake | 0.51(0.25,1.02) | 0.056125 | 0.5(0.21,1.15) | 0.100558 | 0.46(0.07,2.99) | 0.41966 | 0(-0.02,0.03) | 0.977569 | 0.44 | 0.003083 | 1 |
| overall lamb/mutton intake | 0.51(0.27,0.95) | 0.034361 | 0.45(0.2,0.99) | 0.047532 | 1.56(0.25,9.62) | 0.63446 | -0.01(-0.04,0.01) | 0.232268 | 0.56 | 0.0173 | 1 |
| overall pork intake | 0.53(0.23,1.21) | 0.128785 | 0.51(0.19,1.36) | 0.180676 | 0.34(0.03,4.61) | 0.420124 | 0(-0.03,0.04) | 0.771642 | 0.45 | 0.017127 | 1 |
| overall cheese intake | 0.64(0.4,1) | 0.05163 | 0.72(0.38,1.35) | 0.299208 | 0.78(0.25,2.42) | 0.671792 | 0(-0.02,0.01) | 0.515112 | 0.55 | 0.000218 | 0 |
| frequency of adding salt to food | 1.07(0.69,1.66) | 0.771648 | 0.95(0.53,1.71) | 0.866185 | 0.59(0.19,1.81) | 0.355434 | 0.01(-0.01,0.03) | 0.204929 | 1.19 | 0.261791 | 0 |
| temperature of hot drinks | 0.97(0.6,1.57) | 0.911238 | 1.1(0.59,2.06) | 0.756867 | 0.91(0.25,3.35) | 0.886094 | 0(-0.01,0.02) | 0.792178 | 1.08 | 0.667061 | 0 |
| overall alcohol intake | 0.64(0.42,0.97) | 0.034496 | 0.62(0.39,0.99) | 0.046149 | 0.51(0.25,1.03) | 0.059352 | 0(-0.01,0.01) | 0.351648 | 0.69 | 0.005638 | 0 |
| among current drinkers, drinks usually with meals: yes, it varies, no | 0.67(0.39,1.15) | 0.144569 | 0.55(0.27,1.12) | 0.101466 | 3.14(0.56,17.51) | 0.191063 | -0.02(-0.05,0) | 0.053522 | 0.61 | 0.019958 | 1 |
| milk type: skimmed, semi-skimmed, full cream (QT) | 0.47(0.18,1.23) | 0.122012 | 0.58(0.22,1.56) | 0.282185 | 0.59(0.03,9.99) | 0.712813 | 0(-0.04,0.04) | 0.917806 | 0.51 | 0.056792 | 0 |

MR: mendelian randomization; MRPRESSO: MR pleiotropy residual sum and outlier; OR: odd ratio; CI: confidence interval; No.: number.

**Table S31. Leave-one-out analysis of association between genetically predicted dietary habits and anxiety.**

| **Exposure** | **IVW Estimate**  **[Min, Max] ^a^** | **P value [Min, Max] ^b^** |
| --- | --- | --- |
| alcohol drinker status: current + former vs. never | [0.25,0.47] | [0.108787296343723,0.417841806435046] |
| alcohol drinker status: current vs. never | [0.31,0.62] | [0.169958749983572,0.603995242051345] |
| among current drinkers, drinks usually with meals: yes + it varies vs. no | [0.3,0.34] | [0.0000000260176326401041,0.000000612903682666329] |
| among current drinkers, drinks usually with meals: yes vs. no | [0.57,0.61] | [0.0000662412788883023,0.000507505166335728] |
| never eat eggs vs. no eggs, dairy, wheat, or sugar restrictions | [0.31,2.7] | [0.430002217229791,0.97520627065173] |
| never eat eggs vs. no eggs restrictions | [0.05,0.36] | [0.181635878388151,0.651271231919112] |
| never eat dairy vs. no eggs, dairy, wheat, or sugar restrictions | [0.34,0.79] | [0.372372318192722,0.843060233076132] |
| never eat dairy vs. no dairy restrictions | [0.24,0.68] | [0.340127338469151,0.801719191511915] |
| never eat wheat vs. no eggs, dairy, wheat, or sugar restrictions | [0.15,0.28] | [0.00922558329687822,0.163087874817547] |
| never eat wheat vs. no wheat restrictions | [0.1,0.25] | [0.0158202771569772,0.237693154710869] |
| never eat sugar vs. no eggs, dairy, wheat, or sugar restrictions | [1.64,1.86] | [0.0111109640799902,0.0379905598743043] |
| never eat sugar vs. no sugar restrictions | [1.53,1.75] | [0.0233668566130439,0.0758911688554303] |
| milk type: dairy-based milk vs. never | [0.19,0.66] | [0.215115413534119,0.752426051339958] |
| milk type: any milk vs. never | [0.71,2.51] | [0.473746091064107,0.984769666865768] |
| milk type: full cream vs. never | [0.83,0.98] | [0.274887269025749,0.912211347384604] |
| milk type: full cream vs. any other | [0.25,0.43] | [0.0291635545948074,0.180990212390346] |
| milk type: semi-skimmed vs. never | [0.32,0.71] | [0.22991383139991,0.726033477425494] |
| milk type: semi-skimmed vs. any other | [0.24,0.35] | [0.00074236178886311,0.0121347793379171] |
| milk type: skimmed vs. never | [0.88,1.09] | [0.678861011871618,0.988362060943323] |
| milk type: skimmed vs. any other | [2.81,3.74] | [0.00149599480368631,0.0157967602031829] |
| milk type: soy milk vs. never | [1.01,1.15] | [0.295168309043819,0.938309883338318] |
| milk type: soy milk vs. any other | [0.52,1.88] | [0.550346298624655,0.980097551018234] |
| milk type: other milk vs. never | [1.34,1.51] | [0.00929421912569431,0.0631809015586743] |
| milk type: other milk vs. any other | [10.42,178.11] | [0.0703282642633086,0.410230769444353] |
| spread type: all spreads vs. never | [1.31,1.79] | [0.217067626645097,0.578123582112438] |
| spread type: butter + margarine vs. never | [0.94,1.15] | [0.626136704277743,0.995770609801965] |
| spread type: any oil based spread vs. never | [1.05,1.35] | [0.271884081494173,0.862176312635895] |
| spread type: butter and butter-like spreads vs. oil-based spreads | [0.72,0.95] | [0.30193761417885,0.875167481766911] |
| spread type: butter and margarine spreads vs. oil-based spreads | [0.82,0.98] | [0.419471890394839,0.928694425984609] |
| spread type: butter vs. never | [0.91,1.09] | [0.698812302118305,0.999986300072621] |
| spread type: butter vs. any other | [0.71,0.8] | [0.119479248836122,0.305139620563462] |
| spread type: tub margarine vs. never | [1,1.19] | [0.403069140127642,0.997090028756224] |
| spread type: tub margarine vs. any other | [0.96,2.03] | [0.445376779686193,0.99755424000854] |
| spread type: flora + benecol vs. never | [1.21,1.37] | [0.0940577961878616,0.318452280006217] |
| spread type: flora + benecol vs. any other | [1.1,1.43] | [0.472272799169988,0.836986538356233] |
| spread type: olive oil spread vs. never | [1.02,1.33] | [0.210858192114701,0.931131699516333] |
| spread type: olive oil spread vs. any other | [1.25,4.82] | [0.0807902838718328,0.847161949791892] |
| spread type: other oil-based spread vs. never | [0.93,1.1] | [0.622597185773425,0.993445922565934] |
| spread type: other oil-based spread vs. any other | [0.39,0.64] | [0.0942154418127065,0.42807342920264] |
| spread type: low fat spread vs. never | [0.96,1.17] | [0.567464995916957,0.891788522961263] |
| spread type: low fat spread vs. any other | [0.83,3.28] | [0.329774953715219,0.881321827604676] |
| bread type: white vs. any other | [1.28,1.39] | [0.0960432798102067,0.21944896378802] |
| bread type: brown vs. any other | [0.74,1.67] | [0.57625188739437,0.965136346695824] |
| bread type: wholemeal/wholegrain vs. any other | [0.88,0.96] | [0.496731876483256,0.830499396857488] |
| bread type: white vs. wholemeal/wholegrain + brown | [1.49,1.61] | [0.00632491282917843,0.0232276638452047] |
| bread type: wholemeal/wholegrain vs. white + brown | [0.8,0.86] | [0.196634406353431,0.394857400587238] |
| cereal type: biscuit cereal vs. any other | [0.98,1.69] | [0.276129211149275,0.978212732812235] |
| cereal type: bran cereal vs. any other | [0.53,1.21] | [0.412463307682602,0.944646828410388] |
| cereal type: oat cereal vs. any other | [1.62,2.26] | [0.0724181681160278,0.285060771807251] |
| cereal type: muesli vs. any other | [0.52,0.59] | [0.0070348657760109,0.0303498329524454] |
| cereal type: cornflakes/frosties vs. any other | [0.41,0.49] | [0.00077462726832568,0.00928460240575963] |
| coffee type: decaffeinated vs. any other | [0.73,0.8] | [0.0570509389153146,0.178852391406337] |
| coffee type: ground+instant vs. other+decaff | [0.55,0.77] | [0.132257271186204,0.5243437516543] |
| coffee type: ground vs. any other | [0.54,0.82] | [0.211656781790551,0.693954005228132] |
| tablespoons of cooked vegetables per day | [1.25,1.41] | [0.141904624617343,0.356753414849008] |
| tablespoons of raw vegetables per day | [1.13,1.27] | [0.251044939220949,0.54535548454977] |
| pieces of fresh fruit per day | [0.83,0.9] | [0.202131525127431,0.457901697682308] |
| pieces of dried fruit per day | [0.73,0.82] | [0.222961370369596,0.448662307898932] |
| slices of bread per week | [1.08,1.21] | [0.369842537755884,0.726898872618132] |
| bowls of cereal per week | [0.53,0.58] | [0.000188602784452255,0.0011844715832048] |
| cups of tea per day | [0.92,1] | [0.65390137366507,0.991360237374836] |
| cups of coffee per day | [0.79,0.89] | [0.147619113631178,0.448173876429211] |
| glasses of water per day | [1.3,1.39] | [0.0276602089036436,0.0707337474576002] |
| champagne/white wine glasses per month | [0.54,0.64] | [0.034284977992397,0.150884159594831] |
| red wine glasses per month | [0.56,0.62] | [0.00132761892780923,0.00839918194940898] |
| beer/cider glasses per month | [0.98,1.07] | [0.720951357073837,0.982015579260919] |
| spirits measures per month | [0.82,1.07] | [0.602110425965811,0.999187829416514] |
| fortwine glasses per month | [0.87,1.96] | [0.294843924087835,0.986476214989021] |
| other alcohol glasses per month | [0.28,0.84] | [0.216325828676547,0.830339659078245] |
| total drinks of alcohol per month | [0.88,0.97] | [0.359538925192054,0.846233703320727] |
| overall oily fish intake | [1.04,1.12] | [0.500797029392173,0.838313905979711] |
| overall non-oily fish intake | [0.84,1.04] | [0.580717601268362,0.969569905923445] |
| overall processed meat intake | [0.72,0.81] | [0.198962379010944,0.400618003861704] |
| overall poultry intake | [0.68,0.83] | [0.237196051501867,0.599082650973833] |
| overall beef intake | [0.44,0.52] | [0.00238856611863787,0.0194823668384748] |
| overall lamb/mutton intake | [0.5,0.56] | [0.00432116129394946,0.0173269304648558] |
| overall pork intake | [0.45,0.55] | [0.0145279208585107,0.0746349009338749] |
| overall cheese intake | [0.53,0.57] | [0.0000700040076294289,0.000376867193633867] |
| frequency of adding salt to food | [1.16,1.23] | [0.171364205961573,0.334492220820066] |
| temperature of hot drinks | [1.02,1.11] | [0.531648322449265,0.890714178138127] |
| overall alcohol intake | [0.67,0.72] | [0.00254456982031582,0.0143462673740236] |
| among current drinkers, drinks usually with meals: yes, it varies, no | [0.58,0.64] | [0.00799351882512427,0.0315888777442124] |
| milk type: skimmed, semi-skimmed, full cream (QT) | [0.46,0.58] | [0.0249214589315825,0.114540676668897] |

**^a^** the minimum value and maximum value of inverse variance weighted estimate;

**^b^** the minimum value and maximum value of P value;

IVW: inverse variance weighted.

**Table S32. Associations between genetically predicted dietary habits and MDD using IVW** **in replication analysis using a second MDD dataset and the comparation with primary analysis.**

| **Exposure** | **MDD* (Replication)** | | | | | **MDD (Primary)** | | | | |
| --- | --- | --- | --- | --- | --- | --- | --- | --- | --- | --- |
|  | **N snps** | **OR** | **95% CI** | **P value** | **I^2^** | **N snps** | **OR** | **95% CI** | **P value** | **I^2^** |
| alcohol drinker status: current + former vs. never | 37 | 1.595696 | [0.9,2.84] | 0.113136 | 21.21149 | 42 | 1.104429 | [0.77,1.59] | 0.590653 | 49.25291 |
| alcohol drinker status: current vs. never | 36 | 1.426701 | [0.83,2.44] | 0.195302 | 12.81641 | 40 | 1.083916 | [0.76,1.54] | 0.655581 | 48.4753 |
| among current drinkers, drinks usually with meals: yes + it varies vs. no | 80 | 0.632587 | [0.52,0.78] | 1.18E-05 | 40.67687 | 107 | 0.717905 | [0.64,0.81] | 4.33E-08 | 64.33498 |
| among current drinkers, drinks usually with meals: yes vs. no | 125 | 0.785821 | [0.7,0.89] | 7.66E-05 | 40.17761 | 156 | 0.802043 | [0.75,0.86] | 9.68E-11 | 59.0971 |
| never eat eggs vs. no eggs, dairy, wheat, or sugar restrictions | 7 | 1.249962 | [0.31,5.04] | 0.753955 | 44.57506 | 11 | 1.73099 | [1.09,2.75] | 0.019965 | 7.140067 |
| never eat eggs vs. no eggs restrictions | 5 | 0.549229 | [0.1,2.88] | 0.478402 | 19.73782 | 7 | 1.626274 | [0.82,3.21] | 0.160744 | 0 |
| never eat dairy vs. no eggs, dairy, wheat, or sugar restrictions | 12 | 0.729546 | [0.3,1.78] | 0.489685 | 0 | 17 | 0.894736 | [0.44,1.81] | 0.757057 | 71.44394 |
| never eat dairy vs. no dairy restrictions | 11 | 0.598863 | [0.19,1.86] | 0.374583 | 0 | 17 | 0.870695 | [0.36,2.1] | 0.757985 | 71.73021 |
| never eat wheat vs. no eggs, dairy, wheat, or sugar restrictions | 22 | 0.273148 | [0.13,0.57] | 0.000603 | 71.70099 | 18 | 0.359952 | [0.19,0.68] | 0.001602 | 85.99556 |
| never eat wheat vs. no wheat restrictions | 22 | 0.181633 | [0.08,0.42] | 7.20E-05 | 67.92594 | 19 | 0.299269 | [0.14,0.64] | 0.002033 | 86.06202 |
| never eat sugar vs. no eggs, dairy, wheat, or sugar restrictions | 93 | 1.155469 | [0.95,1.4] | 0.140541 | 30.7145 | 123 | 1.031498 | [0.93,1.15] | 0.565159 | 52.54903 |
| never eat sugar vs. no sugar restrictions | 93 | 1.126205 | [0.93,1.36] | 0.223057 | 25.76 | 121 | 1.020089 | [0.92,1.14] | 0.719528 | 51.88151 |
| milk type: dairy-based milk vs. never | 16 | 0.568222 | [0.21,1.56] | 0.27387 | 33.76819 | 16 | 0.665644 | [0.32,1.38] | 0.273892 | 70.84704 |
| milk type: any milk vs. never | 17 | 0.809132 | [0.3,2.17] | 0.673844 | 24.75275 | 16 | 1.356225 | [0.78,2.35] | 0.278176 | 42.75223 |
| milk type: full cream vs. never | 16 | 1.004756 | [0.86,1.18] | 0.953283 | 45.57099 | 25 | 0.964501 | [0.9,1.04] | 0.332563 | 57.24899 |
| milk type: full cream vs. any other | 22 | 0.36059 | [0.16,0.8] | 0.012282 | 64.20404 | 33 | 0.878855 | [0.54,1.43] | 0.604276 | 81.91717 |
| milk type: semi-skimmed vs. never | 17 | 0.642012 | [0.33,1.25] | 0.19313 | 24.64472 | 16 | 0.645972 | [0.39,1.07] | 0.088881 | 66.81631 |
| milk type: semi-skimmed vs. any other | 23 | 1.011414 | [0.68,1.5] | 0.954685 | 42.1777 | 30 | 0.932735 | [0.73,1.19] | 0.574372 | 69.63785 |
| milk type: skimmed vs. never | 19 | 0.925847 | [0.74,1.16] | 0.503793 | 9.364623 | 23 | 1.122409 | [1.01,1.25] | 0.03734 | 14.71061 |
| milk type: skimmed vs. any other | 34 | 1.372654 | [0.9,2.09] | 0.137947 | 59.3741 | 41 | 1.181449 | [0.93,1.51] | 0.179915 | 73.20778 |
| milk type: soy milk vs. never | 18 | 1.017097 | [0.92,1.13] | 0.74721 | 19.26825 | 24 | 1.023246 | [0.98,1.07] | 0.34989 | 31.36008 |
| milk type: soy milk vs. any other | 15 | 2.278507 | [0.64,8.05] | 0.201086 | 66.27838 | 24 | 1.493041 | [0.82,2.71] | 0.188493 | 72.42091 |
| milk type: other milk vs. never | 7 | 1.061826 | [0.93,1.21] | 0.381866 | 0 | 14 | 1.022355 | [0.97,1.08] | 0.38939 | 18.17158 |
| milk type: other milk vs. any other | 9 | 1.298706 | [0.11,15.04] | 0.834351 | 50.88648 | 14 | 0.991171 | [0.5,1.96] | 0.979683 | 4.366636 |
| spread type: all spreads vs. never | 34 | 0.979823 | [0.62,1.55] | 0.930387 | 45.30331 | 51 | 1.042901 | [0.82,1.32] | 0.726631 | 63.45038 |
| spread type: butter + margarine vs. never | 30 | 1.034375 | [0.74,1.45] | 0.845079 | 49.76514 | 55 | 0.98119 | [0.84,1.15] | 0.815334 | 67.30457 |
| spread type: any oil based spread vs. never | 23 | 0.879343 | [0.62,1.25] | 0.47519 | 67.2139 | 33 | 0.949534 | [0.78,1.16] | 0.611911 | 80.92905 |
| spread type: butter and butter-like spreads vs. oil-based spreads | 33 | 0.78777 | [0.6,1.04] | 0.092022 | 35.38861 | 41 | 0.94686 | [0.82,1.1] | 0.472306 | 53.68775 |
| spread type: butter and margarine spreads vs. oil-based spreads | 43 | 0.79819 | [0.61,1.05] | 0.10285 | 56.91529 | 52 | 0.965683 | [0.84,1.11] | 0.633916 | 67.28535 |
| spread type: butter vs. never | 33 | 1.005454 | [0.8,1.26] | 0.96204 | 32.65688 | 57 | 0.980877 | [0.88,1.09] | 0.719741 | 55.68674 |
| spread type: butter vs. any other | 72 | 0.844939 | [0.68,1.05] | 0.123627 | 46.60589 | 98 | 0.949933 | [0.84,1.08] | 0.42592 | 68.86592 |
| spread type: tub margarine vs. never | 23 | 1.003883 | [0.84,1.2] | 0.966436 | 54.72172 | 28 | 0.991 | [0.87,1.12] | 0.887114 | 78.41799 |
| spread type: tub margarine vs. any other | 22 | 1.613572 | [0.79,3.3] | 0.190851 | 49.72528 | 28 | 1.056598 | [0.75,1.48] | 0.749621 | 49.57552 |
| spread type: flora + benecol vs. never | 20 | 1.017971 | [0.85,1.22] | 0.848077 | 41.82869 | 27 | 0.967885 | [0.88,1.06] | 0.477273 | 54.20041 |
| spread type: flora + benecol vs. any other | 30 | 1.103123 | [0.73,1.68] | 0.646262 | 25.52742 | 40 | 1.066965 | [0.91,1.25] | 0.433759 | 11.56204 |
| spread type: olive oil spread vs. never | 16 | 0.970181 | [0.76,1.24] | 0.811561 | 53.1505 | 26 | 1.006707 | [0.89,1.13] | 0.912655 | 67.55288 |
| spread type: olive oil spread vs. any other | 11 | 0.96455 | [0.53,1.75] | 0.905912 | 15.14185 | 16 | 0.765415 | [0.55,1.06] | 0.104134 | 41.33861 |
| spread type: other oil-based spread vs. never | 28 | 0.857686 | [0.68,1.09] | 0.207179 | 63.46518 | 40 | 0.924528 | [0.81,1.05] | 0.241775 | 77.47105 |
| spread type: other oil-based spread vs. any other | 21 | 0.861996 | [0.53,1.39] | 0.543199 | 38.54773 | 23 | 1.015789 | [0.76,1.35] | 0.914575 | 58.28036 |
| spread type: low fat spread vs. never | 10 | 1.183065 | [0.98,1.42] | 0.073655 | 0 | 12 | 1.104566 | [0.98,1.25] | 0.106085 | 48.66415 |
| spread type: low fat spread vs. any other | 13 | 1.22216 | [0.54,2.76] | 0.629346 | 16.86971 | 15 | 1.749806 | [1.09,2.81] | 0.021083 | 46.53669 |
| bread type: white vs. any other | 122 | 1.003098 | [0.84,1.2] | 0.973205 | 52.14186 | 165 | 1.095199 | [0.99,1.21] | 0.0635 | 65.21394 |
| bread type: brown vs. any other | 17 | 0.515346 | [0.27,0.98] | 0.043424 | 47.80009 | 18 | 0.875173 | [0.64,1.2] | 0.412345 | 46.53838 |
| bread type: wholemeal/wholegrain vs. any other | 110 | 0.983154 | [0.82,1.18] | 0.857872 | 56.52315 | 141 | 0.945729 | [0.85,1.05] | 0.305296 | 72.26402 |
| bread type: white vs. wholemeal/wholegrain + brown | 136 | 1.000784 | [0.85,1.17] | 0.992357 | 48.38794 | 178 | 1.091191 | [1,1.19] | 0.05016 | 63.2363 |
| bread type: wholemeal/wholegrain vs. white + brown | 125 | 1.003747 | [0.85,1.19] | 0.965635 | 56.66783 | 166 | 0.956149 | [0.87,1.05] | 0.326903 | 67.65262 |
| cereal type: biscuit cereal vs. any other | 17 | 1.760937 | [1.14,2.72] | 0.010989 | 25.46029 | 29 | 1.406321 | [1.16,1.71] | 0.000654 | 40.65365 |
| cereal type: bran cereal vs. any other | 14 | 0.712768 | [0.41,1.24] | 0.232351 | 39.07162 | 19 | 1.096675 | [0.82,1.47] | 0.533254 | 58.23683 |
| cereal type: oat cereal vs. any other | 16 | 1.408436 | [0.96,2.08] | 0.083472 | 19.92672 | 25 | 1.200467 | [1,1.45] | 0.054072 | 41.34068 |
| cereal type: muesli vs. any other | 67 | 0.693373 | [0.55,0.87] | 0.001451 | 42.71302 | 88 | 0.742595 | [0.65,0.85] | 3.30E-05 | 68.69764 |
| cereal type: cornflakes/frosties vs. any other | 54 | 0.631855 | [0.45,0.89] | 0.00867 | 69.12886 | 73 | 0.898237 | [0.75,1.08] | 0.255601 | 78.50384 |
| coffee type: decaffeinated vs. any other | 115 | 1.03678 | [0.87,1.24] | 0.686748 | 53.4758 | 156 | 0.958664 | [0.86,1.06] | 0.430587 | 73.24185 |
| coffee type: ground+instant vs. other+decaff | 23 | 1.242768 | [0.87,1.77] | 0.232041 | 29.53281 | 34 | 0.855211 | [0.7,1.05] | 0.128498 | 61.67447 |
| coffee type: ground vs. any other | 22 | 0.869635 | [0.61,1.25] | 0.449133 | 25.43291 | 32 | 1.179273 | [0.97,1.43] | 0.095165 | 53.43761 |
| tablespoons of cooked vegetables per day | 97 | 1.075975 | [0.9,1.29] | 0.428184 | 32.16581 | 128 | 1.138746 | [1.02,1.28] | 0.025658 | 63.09761 |
| tablespoons of raw vegetables per day | 106 | 1.17122 | [0.95,1.44] | 0.129553 | 50.46158 | 149 | 1.095765 | [1,1.2] | 0.058948 | 56.04983 |
| pieces of fresh fruit per day | 195 | 0.964421 | [0.85,1.1] | 0.581414 | 40.98579 | 256 | 0.90455 | [0.84,0.97] | 0.007932 | 63.29075 |
| pieces of dried fruit per day | 92 | 0.842906 | [0.66,1.07] | 0.163594 | 58.61278 | 123 | 0.900025 | [0.79,1.03] | 0.118031 | 73.24384 |
| slices of bread per week | 101 | 0.979571 | [0.8,1.19] | 0.838229 | 45.81525 | 129 | 0.972299 | [0.87,1.08] | 0.6143 | 62.17711 |
| bowls of cereal per week | 121 | 0.81352 | [0.67,0.98] | 0.030527 | 50.81627 | 174 | 0.853291 | [0.77,0.94] | 0.001727 | 67.20659 |
| cups of tea per day | 139 | 0.924458 | [0.8,1.06] | 0.270453 | 36.3903 | 182 | 1.073699 | [0.98,1.18] | 0.123519 | 66.64741 |
| cups of coffee per day | 105 | 1.066465 | [0.92,1.23] | 0.3817 | 36.99902 | 140 | 0.945496 | [0.86,1.04] | 0.240227 | 68.31912 |
| glasses of water per day | 199 | 1.105765 | [0.97,1.26] | 0.139376 | 45.56322 | 274 | 1.004669 | [0.94,1.08] | 0.898816 | 61.45889 |
| champagne/white wine glasses per month | 59 | 1.01018 | [0.8,1.27] | 0.931893 | 39.85678 | 73 | 0.891882 | [0.77,1.03] | 0.131495 | 65.64935 |
| red wine glasses per month | 97 | 0.85463 | [0.71,1.03] | 0.097577 | 50.01013 | 137 | 0.821103 | [0.73,0.92] | 0.000507 | 68.53196 |
| beer/cider glasses per month | 80 | 1.071769 | [0.89,1.3] | 0.473036 | 45.53465 | 117 | 1.005095 | [0.89,1.13] | 0.933943 | 70.71887 |
| spirits measures per month | 29 | 1.011668 | [0.72,1.42] | 0.945977 | 35.84766 | 46 | 0.944908 | [0.79,1.13] | 0.534508 | 58.46378 |
| fortwine glasses per month | 7 | 0.633682 | [0.35,1.15] | 0.13543 | 16.84715 | 15 | 0.768079 | [0.58,1.01] | 0.058797 | 45.90009 |
| other alcohol glasses per month | 5 | 0.68438 | [0.36,1.31] | 0.25177 | 47.71966 | 11 | 0.999263 | [0.82,1.21] | 0.994027 | 25.05744 |
| total drinks of alcohol per month | 217 | 0.963036 | [0.86,1.08] | 0.525462 | 42.76472 | 284 | 0.907522 | [0.84,0.98] | 0.010877 | 66.26383 |
| overall oily fish intake | 144 | 1.113021 | [0.94,1.31] | 0.201802 | 48.66557 | 188 | 1.051503 | [0.95,1.16] | 0.320784 | 72.03833 |
| overall non-oily fish intake | 57 | 1.199947 | [0.89,1.61] | 0.227384 | 50.98018 | 73 | 1.048061 | [0.91,1.21] | 0.525237 | 57.10898 |
| overall processed meat intake | 69 | 0.882594 | [0.68,1.14] | 0.346492 | 47.88729 | 108 | 0.941007 | [0.83,1.07] | 0.351151 | 64.26498 |
| overall poultry intake | 43 | 1.0448 | [0.77,1.42] | 0.780381 | 40.8678 | 61 | 1.110866 | [0.95,1.31] | 0.201768 | 57.98627 |
| overall beef intake | 79 | 0.707786 | [0.59,0.86] | 0.000374 | 19.54756 | 104 | 0.77092 | [0.67,0.88] | 0.00014 | 66.45782 |
| overall lamb/mutton intake | 92 | 0.772347 | [0.64,0.93] | 0.006309 | 29.37774 | 124 | 0.913534 | [0.81,1.04] | 0.159429 | 69.68708 |
| overall pork intake | 54 | 0.782706 | [0.57,1.08] | 0.134269 | 56.72211 | 77 | 0.86047 | [0.74,1.01] | 0.058628 | 66.24908 |
| overall cheese intake | 161 | 0.902744 | [0.78,1.04] | 0.15889 | 36.7216 | 222 | 0.889716 | [0.82,0.96] | 0.003689 | 57.51323 |
| frequency of adding salt to food | 163 | 1.174325 | [1.03,1.34] | 0.014752 | 30.10034 | 232 | 1.153577 | [1.07,1.24] | 0.000142 | 57.3036 |
| temperature of hot drinks | 132 | 0.846694 | [0.72,1] | 0.051381 | 43.87668 | 186 | 0.890551 | [0.81,0.98] | 0.013156 | 63.65433 |
| overall alcohol intake | 238 | 0.875448 | [0.78,0.98] | 0.017327 | 35.64425 | 295 | 0.889306 | [0.83,0.95] | 0.001108 | 62.5344 |
| among current drinkers, drinks usually with meals: yes, it varies, no | 107 | 0.756056 | [0.63,0.9] | 0.00181 | 42.32647 | 131 | 0.791582 | [0.72,0.87] | 9.36E-07 | 55.17299 |
| milk type: skimmed, semi-skimmed, full cream (QT) | 41 | 0.553844 | [0.41,0.75] | 0.00012 | 44.71736 | 55 | 0.797578 | [0.64,0.99] | 0.042962 | 77.80426 |

MDD: Major depression disorder; IVW: inverse variance weighted; OR: odd ratio; CI: confidence interval.

**Table S33. Associations between genetically predicted dietary habits and MDD using other sensitivity analysis methods** **in replication analysis using a second MDD dataset.**

| **Exposure** | **Weighted median** | | **Mode-based** | | **MR-Egger** | | | | **MRPRESSO** | | |
| --- | --- | --- | --- | --- | --- | --- | --- | --- | --- | --- | --- |
|  | **OR (95% CI)** | **P value** | **OR (95% CI)** | **P value** | **OR (95% CI)** | **P value** | **Intercept** | **P value** | **OR** | **P value** | **No. of outliers** |
| alcohol drinker status: current + former vs. never | 1.14(0.52,2.5) | 0.737221 | 1.19(0.56,2.5) | 0.653177 | 2.17(0.61,7.71) | 0.229401 | 0(-0.01,0.01) | 0.589726 | 1.6 | 0.121882 | 0 |
| alcohol drinker status: current vs. never | 1.03(0.48,2.2) | 0.935322 | 1.16(0.58,2.32) | 0.683983 | 2.13(0.65,7.01) | 0.21374 | 0(-0.01,0.01) | 0.459634 | 1.43 | 0.20378 | 0 |
| among current drinkers, drinks usually with meals: yes + it varies vs. no | 0.58(0.45,0.74) | 1.11E-05 | 0.57(0.43,0.75) | 7.64E-05 | 0.94(0.42,2.07) | 0.87289 | -0.01(-0.02,0.01) | 0.31346 | 0.6 | 1.65E-06 | 1 |
| among current drinkers, drinks usually with meals: yes vs. no | 0.77(0.67,0.89) | 0.000467 | 0.8(0.67,0.96) | 0.014779 | 0.88(0.55,1.4) | 0.595161 | 0(-0.01,0.01) | 0.617079 | 0.78 | 2.41E-05 | 2 |
| never eat eggs vs. no eggs, dairy, wheat, or sugar restrictions | 2.64(0.57,12.14) | 0.213593 | 1.15(0.29,4.61) | 0.839105 | 0.26(0,73.26) | 0.64194 | 0.01(-0.03,0.05) | 0.573766 | 1.25 | 0.764565 | 0 |
| never eat eggs vs. no eggs restrictions | 0.27(0.03,2.22) | 0.222577 | 0.35(0.05,2.39) | 0.287448 | 0.05(0,18.46) | 0.326328 | 0.01(-0.02,0.05) | 0.413862 | 0.55 | 0.517532 | 0 |
| never eat dairy vs. no eggs, dairy, wheat, or sugar restrictions | 0.79(0.24,2.59) | 0.701002 | 0.62(0.19,2.01) | 0.428289 | 0.21(0.02,2.34) | 0.203439 | 0.01(-0.01,0.03) | 0.273864 | 0.73 | 0.337515 | 0 |
| never eat dairy vs. no dairy restrictions | 0.59(0.14,2.56) | 0.479839 | 0.55(0.13,2.4) | 0.429874 | 0.14(0.01,2.76) | 0.194781 | 0.01(-0.01,0.03) | 0.298986 | 0.6 | 0.184927 | 0 |
| never eat wheat vs. no eggs, dairy, wheat, or sugar restrictions | 0.26(0.12,0.55) | 0.000442 | 0.19(0.11,0.32) | 5.13E-10 | 0.12(0.04,0.41) | 0.000603 | 0.02(0,0.03) | 0.105726 | 0.53 | 0.092657 | 2 |
| never eat wheat vs. no wheat restrictions | 0.18(0.07,0.44) | 0.000206 | 0.13(0.07,0.24) | 1.39E-10 | 0.11(0.03,0.44) | 0.001888 | 0.01(-0.01,0.03) | 0.347814 | 0.38 | 0.029673 | 2 |
| never eat sugar vs. no eggs, dairy, wheat, or sugar restrictions | 1.17(0.92,1.49) | 0.199226 | 1.06(0.8,1.41) | 0.673539 | 1.04(0.51,2.14) | 0.907283 | 0(-0.01,0.01) | 0.772672 | 1.18 | 0.072757 | 2 |
| never eat sugar vs. no sugar restrictions | 1.09(0.85,1.39) | 0.489516 | 1.03(0.77,1.38) | 0.825456 | 0.8(0.4,1.6) | 0.532409 | 0(0,0.01) | 0.315606 | 1.18 | 0.075926 | 1 |
| milk type: dairy-based milk vs. never | 0.63(0.19,2.14) | 0.463192 | 0.74(0.24,2.28) | 0.598295 | 0.37(0.01,14.95) | 0.594863 | 0(-0.02,0.03) | 0.80785 | 0.57 | 0.29113 | 0 |
| milk type: any milk vs. never | 0.71(0.21,2.4) | 0.576474 | 0.82(0.26,2.54) | 0.726227 | 0.29(0.02,3.28) | 0.314335 | 0.01(-0.01,0.03) | 0.360207 | 0.81 | 0.679443 | 0 |
| milk type: full cream vs. never | 1.07(0.89,1.27) | 0.482783 | 1.11(0.93,1.31) | 0.246469 | 1.15(0.76,1.74) | 0.51551 | -0.01(-0.03,0.01) | 0.49556 | 1 | 0.954057 | 1 |
| milk type: full cream vs. any other | 0.33(0.15,0.73) | 0.005745 | 0.37(0.17,0.81) | 0.012753 | 0.16(0.01,4.14) | 0.272938 | 0.01(-0.02,0.04) | 0.622634 | 0.36 | 0.010643 | 2 |
| milk type: semi-skimmed vs. never | 0.72(0.31,1.67) | 0.445048 | 0.78(0.36,1.7) | 0.530477 | 0.48(0.05,4.4) | 0.51676 | 0(-0.02,0.03) | 0.787621 | 0.64 | 0.211558 | 0 |
| milk type: semi-skimmed vs. any other | 1.23(0.77,1.95) | 0.381118 | 1.08(0.71,1.64) | 0.715091 | 0.93(0.34,2.55) | 0.882424 | 0(-0.01,0.02) | 0.853355 | 1.01 | 0.955198 | 1 |
| milk type: skimmed vs. never | 0.87(0.64,1.17) | 0.354385 | 0.93(0.69,1.26) | 0.652431 | 1.25(0.7,2.24) | 0.446203 | -0.01(-0.02,0.01) | 0.267938 | 0.93 | 0.512276 | 0 |
| milk type: skimmed vs. any other | 1.44(0.93,2.24) | 0.105517 | 1.28(0.84,1.95) | 0.257042 | 1.35(0.31,5.89) | 0.691801 | 0(-0.02,0.02) | 0.979566 | 1.2 | 0.342184 | 1 |
| milk type: soy milk vs. never | 1.05(0.92,1.2) | 0.461306 | 1.06(0.93,1.2) | 0.389102 | 0.99(0.74,1.33) | 0.95005 | 0(-0.02,0.02) | 0.850452 | 1.02 | 0.75114 | 0 |
| milk type: soy milk vs. any other | 1.48(0.49,4.45) | 0.481545 | 1.46(0.5,4.27) | 0.493612 | 5.65(0.08,382.03) | 0.420367 | -0.01(-0.04,0.02) | 0.656761 | 1.62 | 0.254464 | 2 |
| milk type: other milk vs. never | 1.02(0.86,1.21) | 0.833832 | 1.02(0.86,1.21) | 0.818346 | 1.04(0.73,1.48) | 0.837901 | 0(-0.03,0.03) | 0.890171 | 1.06 | 0.346616 | 0 |
| milk type: other milk vs. any other | 1.82(0.16,21.39) | 0.633118 | 0.75(0.07,7.78) | 0.812232 | 91.86(0.38,22441.06) | 0.107116 | -0.03(-0.06,0.01) | 0.097078 | 0.46 | 0.419628 | 1 |
| spread type: all spreads vs. never | 1.26(0.75,2.13) | 0.380825 | 1.17(0.66,2.07) | 0.586006 | 3.02(0.71,12.86) | 0.134777 | -0.01(-0.03,0) | 0.109334 | 0.98 | 0.930915 | 1 |
| spread type: butter + margarine vs. never | 1.22(0.84,1.76) | 0.302334 | 1.23(0.83,1.83) | 0.306583 | 2.26(0.74,6.88) | 0.149945 | -0.01(-0.03,0) | 0.148042 | 1.24 | 0.144582 | 2 |
| spread type: any oil based spread vs. never | 1.11(0.82,1.52) | 0.495019 | 1.13(0.84,1.52) | 0.432316 | 2.13(0.71,6.42) | 0.177534 | -0.02(-0.04,0) | 0.097323 | 1 | 0.999646 | 1 |
| spread type: butter and butter-like spreads vs. oil-based spreads | 0.89(0.64,1.23) | 0.467144 | 0.98(0.7,1.36) | 0.891331 | 1.24(0.52,2.95) | 0.632458 | -0.01(-0.02,0.01) | 0.283827 | 0.85 | 0.199707 | 1 |
| spread type: butter and margarine spreads vs. oil-based spreads | 0.85(0.65,1.12) | 0.243329 | 0.96(0.72,1.26) | 0.748137 | 0.94(0.34,2.63) | 0.913533 | 0(-0.02,0.01) | 0.737544 | 0.93 | 0.476763 | 2 |
| spread type: butter vs. never | 1.05(0.8,1.39) | 0.720803 | 1.03(0.77,1.38) | 0.839974 | 1.36(0.65,2.88) | 0.415989 | -0.01(-0.02,0.01) | 0.40197 | 1.08 | 0.464593 | 1 |
| spread type: butter vs. any other | 0.87(0.68,1.11) | 0.259851 | 0.83(0.63,1.11) | 0.208442 | 0.74(0.33,1.65) | 0.46155 | 0(-0.01,0.01) | 0.735291 | 0.84 | 0.128072 | 1 |
| spread type: tub margarine vs. never | 1.09(0.91,1.3) | 0.359358 | 1.12(0.94,1.33) | 0.207549 | 1.05(0.63,1.75) | 0.844531 | 0(-0.02,0.02) | 0.845799 | 1.1 | 0.130678 | 1 |
| spread type: tub margarine vs. any other | 1.74(0.79,3.85) | 0.172552 | 1.1(0.5,2.41) | 0.807681 | 0.34(0.07,1.6) | 0.17153 | 0.02(0,0.03) | 0.028964 | 1.32 | 0.406434 | 1 |
| spread type: flora + benecol vs. never | 1.07(0.87,1.31) | 0.548145 | 1.11(0.9,1.36) | 0.321829 | 0.86(0.51,1.46) | 0.577621 | 0.01(-0.01,0.03) | 0.506137 | 1.02 | 0.85011 | 1 |
| spread type: flora + benecol vs. any other | 1.03(0.6,1.79) | 0.902915 | 0.99(0.59,1.68) | 0.977678 | 0.94(0.31,2.84) | 0.912062 | 0(-0.01,0.02) | 0.757707 | 1.1 | 0.649684 | 0 |
| spread type: olive oil spread vs. never | 1.12(0.87,1.44) | 0.385812 | 1.11(0.86,1.43) | 0.411341 | 1.43(0.68,3.04) | 0.348401 | -0.01(-0.04,0.01) | 0.281682 | 0.97 | 0.814787 | 1 |
| spread type: olive oil spread vs. any other | 1.19(0.56,2.5) | 0.655895 | 1.22(0.59,2.54) | 0.587585 | 0.97(0.22,4.29) | 0.964018 | 0(-0.02,0.02) | 0.997935 | 0.96 | 0.908253 | 0 |
| spread type: other oil-based spread vs. never | 0.95(0.76,1.19) | 0.660985 | 1.09(0.87,1.36) | 0.475185 | 1.1(0.56,2.16) | 0.790075 | -0.01(-0.03,0.01) | 0.447275 | 0.94 | 0.55471 | 1 |
| spread type: other oil-based spread vs. any other | 1.02(0.57,1.8) | 0.95706 | 0.99(0.56,1.77) | 0.98207 | 1.57(0.45,5.46) | 0.47722 | -0.01(-0.02,0.01) | 0.306483 | 0.86 | 0.550042 | 1 |
| spread type: low fat spread vs. never | 1.19(0.94,1.51) | 0.15482 | 1.22(0.97,1.54) | 0.096494 | 1.15(0.78,1.7) | 0.483217 | 0(-0.01,0.02) | 0.87257 | 1.18 | 0.026646 | 0 |
| spread type: low fat spread vs. any other | 0.98(0.34,2.81) | 0.966819 | 0.73(0.25,2.16) | 0.570036 | 0.47(0.07,3.2) | 0.437717 | 0.01(-0.01,0.03) | 0.280265 | 1.22 | 0.63803 | 0 |
| bread type: white vs. any other | 1.11(0.91,1.36) | 0.313143 | 1.1(0.86,1.41) | 0.455002 | 0.77(0.38,1.56) | 0.47372 | 0(-0.01,0.01) | 0.453164 | 1.05 | 0.531601 | 3 |
| bread type: brown vs. any other | 0.57(0.28,1.15) | 0.114909 | 0.55(0.27,1.12) | 0.097803 | 0.41(0.09,1.92) | 0.259611 | 0(-0.01,0.02) | 0.756057 | 0.52 | 0.060499 | 1 |
| bread type: wholemeal/wholegrain vs. any other | 0.92(0.75,1.12) | 0.393696 | 0.91(0.71,1.16) | 0.434282 | 1.12(0.52,2.44) | 0.766759 | 0(-0.01,0.01) | 0.726464 | 0.94 | 0.447042 | 5 |
| bread type: white vs. wholemeal/wholegrain + brown | 1.11(0.92,1.33) | 0.266562 | 1.13(0.89,1.43) | 0.322274 | 0.83(0.46,1.52) | 0.553886 | 0(-0.01,0.01) | 0.537435 | 1.05 | 0.550203 | 3 |
| bread type: wholemeal/wholegrain vs. white + brown | 0.93(0.78,1.12) | 0.449078 | 0.89(0.71,1.12) | 0.330233 | 1.72(0.9,3.29) | 0.098767 | -0.01(-0.02,0) | 0.089391 | 0.98 | 0.836897 | 4 |
| cereal type: biscuit cereal vs. any other | 1.59(0.91,2.76) | 0.102117 | 1.39(0.81,2.39) | 0.230166 | 0.8(0.25,2.55) | 0.705875 | 0.01(0,0.03) | 0.151504 | 1.76 | 0.021706 | 0 |
| cereal type: bran cereal vs. any other | 1.01(0.54,1.91) | 0.966222 | 0.97(0.53,1.76) | 0.912313 | 0.77(0.18,3.24) | 0.717639 | 0(-0.02,0.02) | 0.914407 | 0.71 | 0.253688 | 0 |
| cereal type: oat cereal vs. any other | 1.76(1.05,2.97) | 0.033107 | 1.66(1.02,2.69) | 0.040104 | 0.79(0.26,2.47) | 0.690908 | 0.01(-0.01,0.03) | 0.291957 | 1.41 | 0.103983 | 0 |
| cereal type: muesli vs. any other | 0.73(0.55,0.96) | 0.024761 | 0.75(0.54,1.03) | 0.079771 | 0.58(0.22,1.49) | 0.257177 | 0(-0.01,0.02) | 0.698065 | 0.69 | 0.002215 | 1 |
| cereal type: cornflakes/frosties vs. any other | 0.55(0.4,0.77) | 0.000442 | 0.63(0.44,0.9) | 0.012007 | 0.28(0.1,0.79) | 0.016502 | 0.01(0,0.03) | 0.107041 | 0.65 | 0.005246 | 4 |
| coffee type: decaffeinated vs. any other | 0.96(0.79,1.16) | 0.680862 | 0.92(0.73,1.15) | 0.43858 | 1.12(0.57,2.18) | 0.745252 | 0(-0.01,0.01) | 0.820476 | 0.94 | 0.419218 | 3 |
| coffee type: ground+instant vs. other+decaff | 1.64(1.04,2.56) | 0.032005 | 1.38(0.9,2.14) | 0.143967 | 2.33(0.95,5.73) | 0.064583 | -0.01(-0.03,0) | 0.136324 | 1.24 | 0.244766 | 0 |
| coffee type: ground vs. any other | 0.78(0.49,1.23) | 0.277921 | 0.74(0.48,1.16) | 0.19415 | 0.48(0.2,1.13) | 0.093808 | 0.01(0,0.03) | 0.136768 | 0.87 | 0.457542 | 0 |
| tablespoons of cooked vegetables per day | 1.01(0.8,1.27) | 0.958166 | 1.01(0.76,1.34) | 0.961793 | 1.14(0.52,2.49) | 0.748967 | 0(-0.01,0.01) | 0.888289 | 1.11 | 0.243858 | 1 |
| tablespoons of raw vegetables per day | 1.14(0.91,1.43) | 0.270097 | 1.06(0.82,1.37) | 0.673728 | 2.29(1.13,4.62) | 0.021242 | -0.01(-0.02,0) | 0.051775 | 1.05 | 0.566203 | 3 |
| pieces of fresh fruit per day | 1.01(0.86,1.2) | 0.858896 | 1.02(0.84,1.23) | 0.872297 | 1.03(0.66,1.62) | 0.899019 | 0(-0.01,0.01) | 0.76678 | 0.96 | 0.542481 | 2 |
| pieces of dried fruit per day | 0.68(0.53,0.87) | 0.002494 | 0.61(0.45,0.82) | 0.001295 | 1.34(0.49,3.63) | 0.569398 | -0.01(-0.02,0.01) | 0.351507 | 0.79 | 0.036555 | 1 |
| slices of bread per week | 0.98(0.78,1.24) | 0.880452 | 0.97(0.76,1.24) | 0.813337 | 1.22(0.63,2.37) | 0.55201 | 0(-0.01,0.01) | 0.491623 | 1.08 | 0.388252 | 3 |
| bowls of cereal per week | 0.75(0.61,0.92) | 0.006906 | 0.77(0.6,0.98) | 0.030272 | 0.95(0.45,1.98) | 0.883734 | 0(-0.01,0.01) | 0.678104 | 0.82 | 0.022981 | 3 |
| cups of tea per day | 0.94(0.78,1.15) | 0.569415 | 0.93(0.79,1.11) | 0.438382 | 1.05(0.74,1.49) | 0.798772 | 0(-0.01,0) | 0.45078 | 0.95 | 0.476802 | 1 |
| cups of coffee per day | 1.09(0.9,1.31) | 0.374356 | 1.06(0.9,1.24) | 0.480582 | 0.91(0.69,1.19) | 0.48291 | 0(0,0.01) | 0.171232 | 1.07 | 0.383715 | 1 |
| glasses of water per day | 1.06(0.88,1.27) | 0.556794 | 1.03(0.86,1.23) | 0.772758 | 0.94(0.62,1.42) | 0.76515 | 0(0,0.01) | 0.414859 | 1.1 | 0.142364 | 2 |
| champagne/white wine glasses per month | 0.92(0.68,1.24) | 0.587833 | 0.89(0.67,1.19) | 0.437469 | 1.49(0.83,2.67) | 0.179127 | -0.01(-0.02,0) | 0.153995 | 0.96 | 0.745159 | 1 |
| red wine glasses per month | 0.95(0.75,1.21) | 0.66458 | 0.87(0.7,1.07) | 0.184995 | 0.86(0.53,1.39) | 0.537309 | 0(-0.01,0.01) | 0.976321 | 0.81 | 0.017955 | 2 |
| beer/cider glasses per month | 1.02(0.79,1.32) | 0.862372 | 1.01(0.8,1.29) | 0.913529 | 1(0.62,1.6) | 0.987376 | 0(-0.01,0.01) | 0.740517 | 1.1 | 0.31718 | 1 |
| spirits measures per month | 0.89(0.6,1.31) | 0.557103 | 0.96(0.64,1.45) | 0.859698 | 1.29(0.57,2.88) | 0.539871 | 0(-0.02,0.01) | 0.519115 | 1.1 | 0.544999 | 1 |
| fortwine glasses per month | 0.6(0.28,1.27) | 0.178419 | 0.58(0.28,1.19) | 0.135681 | 0.79(0.2,3.11) | 0.739082 | -0.01(-0.03,0.02) | 0.716251 | 0.63 | 0.186039 | 0 |
| other alcohol glasses per month | 1.07(0.54,2.1) | 0.852792 | 1.14(0.61,2.13) | 0.674415 | 1.31(0.19,8.95) | 0.781876 | -0.02(-0.06,0.03) | 0.476594 | 0.68 | 0.315672 | 0 |
| total drinks of alcohol per month | 0.97(0.8,1.19) | 0.799863 | 0.99(0.85,1.15) | 0.881849 | 1.06(0.83,1.35) | 0.639332 | 0(-0.01,0) | 0.378594 | 0.96 | 0.520961 | 2 |
| overall oily fish intake | 1.03(0.86,1.25) | 0.724766 | 1.05(0.85,1.31) | 0.629869 | 2.29(1.25,4.21) | 0.007393 | -0.01(-0.02,0) | 0.015499 | 1.01 | 0.867738 | 3 |
| overall non-oily fish intake | 1.06(0.76,1.46) | 0.739611 | 0.98(0.69,1.39) | 0.895911 | 0.75(0.28,2.07) | 0.583051 | 0.01(-0.01,0.02) | 0.344554 | 1.06 | 0.645384 | 2 |
| overall processed meat intake | 0.86(0.64,1.15) | 0.315163 | 0.87(0.63,1.2) | 0.384331 | 0.97(0.37,2.56) | 0.950324 | 0(-0.01,0.01) | 0.843291 | 0.95 | 0.672558 | 1 |
| overall poultry intake | 1.04(0.73,1.5) | 0.824422 | 1.09(0.74,1.6) | 0.669083 | 0.75(0.26,2.18) | 0.598524 | 0(-0.01,0.02) | 0.525811 | 1.04 | 0.781749 | 1 |
| overall beef intake | 0.74(0.57,0.95) | 0.020301 | 0.77(0.57,1.05) | 0.09615 | 0.8(0.44,1.45) | 0.459165 | 0(-0.01,0.01) | 0.682866 | 0.71 | 0.00064 | 0 |
| overall lamb/mutton intake | 0.87(0.69,1.1) | 0.235765 | 0.93(0.7,1.22) | 0.581794 | 0.95(0.45,2) | 0.890728 | 0(-0.01,0.01) | 0.57503 | 0.83 | 0.030609 | 2 |
| overall pork intake | 0.78(0.55,1.09) | 0.14231 | 0.91(0.64,1.3) | 0.621376 | 0.83(0.27,2.55) | 0.74975 | 0(-0.02,0.01) | 0.908149 | 0.85 | 0.270207 | 1 |
| overall cheese intake | 0.89(0.74,1.07) | 0.202345 | 0.94(0.74,1.18) | 0.577578 | 0.73(0.44,1.2) | 0.212009 | 0(0,0.01) | 0.377216 | 0.9 | 0.160831 | 1 |
| frequency of adding salt to food | 1.11(0.93,1.32) | 0.260317 | 1.1(0.9,1.35) | 0.333732 | 0.77(0.48,1.21) | 0.257542 | 0.01(0,0.01) | 0.058314 | 1.15 | 0.029509 | 1 |
| temperature of hot drinks | 0.88(0.72,1.07) | 0.18982 | 0.89(0.71,1.13) | 0.345439 | 0.78(0.42,1.46) | 0.431569 | 0(-0.01,0.01) | 0.781966 | 0.88 | 0.090064 | 3 |
| overall alcohol intake | 0.96(0.8,1.17) | 0.706553 | 0.93(0.79,1.08) | 0.326616 | 0.85(0.66,1.11) | 0.240339 | 0(0,0) | 0.83697 | 0.86 | 0.004581 | 2 |
| among current drinkers, drinks usually with meals: yes, it varies, no | 0.76(0.62,0.94) | 0.011437 | 0.78(0.6,1.01) | 0.059044 | 1(0.51,1.96) | 0.993806 | 0(-0.01,0.01) | 0.39069 | 0.73 | 0.000158 | 3 |
| milk type: skimmed, semi-skimmed, full cream (QT) | 0.47(0.34,0.66) | 1.43E-05 | 0.48(0.34,0.67) | 2.18E-05 | 0.72(0.27,1.92) | 0.507869 | 0(-0.02,0.01) | 0.592052 | 0.58 | 4.43E-05 | 2 |

MDD: Major depression disorder; MR: mendelian randomization; MRPRESSO: MR pleiotropy residual sum and outlier; OR: odd ratio; CI: confidence interval; No.: number.

**Table S34. Associations between genetically predicted mediator and migraine in mediation analysis using MVMR.**

| **Exposure** | **Mediator** | **IVW** | | | **MR-Egger** | | | | **Number** | **Alpha** |
| --- | --- | --- | --- | --- | --- | --- | --- | --- | --- | --- |
|  |  | **OR** | **95% CI** | **P value** | **OR** | **95% CI** | **P value** | **P value (Intercept)** |  |  |
| among current drinkers, drinks usually with meals: yes + it varies vs. no | insomnia | 1.22 | [1.14,1.3] | 3.94E-09 | 1.08 | [0.94,1.24] | 0.277221 | 0.050545 | 3 | 0.016667 |
| bread type: white vs. any other | insomnia | 1.19 | [1.12,1.27] | 4.40E-08 | 1.19 | [1.06,1.33] | 0.003488 | 0.936384 | 3 | 0.016667 |
| bread type: white vs. wholemeal/wholegrain + brown | insomnia | 1.19 | [1.12,1.27] | 2.20E-08 | 1.17 | [1.05,1.31] | 0.005028 | 0.691982 | 3 | 0.016667 |
| bread type: wholemeal/wholegrain vs. white + brown | insomnia | 1.2 | [1.13,1.28] | 1.87E-08 | 1.22 | [1.09,1.38] | 0.000825 | 0.675259 | 3 | 0.016667 |
| cereal type: muesli vs. any other | insomnia | 1.21 | [1.13,1.29] | 1.13E-08 | 1.06 | [0.91,1.23] | 0.468129 | 0.053142 | 1 | 0.05 |
| overall alcohol intake | insomnia | 1.21 | [1.14,1.29] | 9.77E-10 | 1.17 | [1.06,1.29] | 0.001806 | 0.36223 | 3 | 0.016667 |
| overall cheese intake | insomnia | 1.21 | [1.14,1.29] | 1.42E-09 | 1.18 | [1.06,1.31] | 0.002201 | 0.524516 | 1 | 0.05 |
| overall oily fish intake | insomnia | 1.21 | [1.13,1.29] | 1.59E-08 | 1.23 | [1.1,1.38] | 0.000387 | 0.711003 | 1 | 0.05 |
| red wine glasses per month | insomnia | 1.2 | [1.13,1.28] | 1.47E-08 | 1.16 | [1.02,1.31] | 0.018902 | 0.509384 | 3 | 0.016667 |
| among current drinkers, drinks usually with meals: yes + it varies vs. no | MDD | 1.47 | [1.27,1.7] | 2.19E-07 | 1.77 | [1.36,2.29] | 1.66E-05 | 0.096028 | 3 | 0.016667 |
| cereal type: muesli vs. any other | MDD | 1.54 | [1.33,1.78] | 6.96E-09 | 1.71 | [1.27,2.3] | 0.000412 | 0.42812 | 1 | 0.05 |
| overall alcohol intake | MDD | 1.5 | [1.31,1.73] | 7.19E-09 | 1.6 | [1.29,1.98] | 1.38E-05 | 0.453946 | 3 | 0.016667 |
| overall cheese intake | MDD | 1.52 | [1.32,1.75] | 3.93E-09 | 1.4 | [1.12,1.76] | 0.003674 | 0.359973 | 1 | 0.05 |
| red wine glasses per month | MDD | 1.43 | [1.23,1.65] | 1.68E-06 | 1.75 | [1.36,2.25] | 1.43E-05 | 0.053443 | 3 | 0.016667 |

*OR*: odds ratios; Number: the total number of exposures in the corresponding subtype; Alpha: Bonferroni-corrected threshold in this subtype; MVMR: multivariable Mendelian randomization; MDD: major depression disorder.

**Table S35. Associations between genetically predicted mediator and migraine with aura in mediation analysis using MVMR.**

| **Exposure** | **Mediator** | **IVW** | | | **MR-Egger** | | | | **Number** | **Alpha** |
| --- | --- | --- | --- | --- | --- | --- | --- | --- | --- | --- |
|  |  | **OR** | **95% CI** | **P value** | **OR** | **95% CI** | **P value** | **P value (Intercept)** |  |  |
| among current drinkers, drinks usually with meals: yes + it varies vs. no | insomnia | 1.18 | [1.08,1.3] | 0.000323 | 1.09 | [0.91,1.31] | 0.356263 | 0.313974 | 3 | 0.016667 |
| bread type: white vs. any other | insomnia | 1.19 | [1.09,1.3] | 0.000189 | 1.24 | [1.05,1.46] | 0.011987 | 0.579149 | 3 | 0.016667 |
| bread type: white vs. wholemeal/wholegrain + brown | insomnia | 1.19 | [1.09,1.31] | 0.000134 | 1.19 | [1.01,1.39] | 0.03995 | 0.909506 | 3 | 0.016667 |
| bread type: wholemeal/wholegrain vs. white + brown | insomnia | 1.17 | [1.07,1.28] | 0.000782 | 1.23 | [1.04,1.46] | 0.018698 | 0.50219 | 3 | 0.016667 |
| cereal type: muesli vs. any other | insomnia | 1.2 | [1.09,1.32] | 0.000128 | 1.01 | [0.81,1.26] | 0.913514 | 0.087491 | 1 | 0.05 |
| overall alcohol intake | insomnia | 1.19 | [1.09,1.3] | 6.65E-05 | 1.19 | [1.03,1.36] | 0.016101 | 0.910972 | 3 | 0.016667 |
| overall cheese intake | insomnia | 1.18 | [1.09,1.29] | 0.000155 | 1.23 | [1.06,1.43] | 0.00575 | 0.531692 | 1 | 0.05 |
| overall oily fish intake | insomnia | 1.19 | [1.09,1.3] | 0.000195 | 1.26 | [1.08,1.48] | 0.004255 | 0.374341 | 1 | 0.05 |
| red wine glasses per month | insomnia | 1.19 | [1.09,1.3] | 0.000108 | 1.07 | [0.91,1.27] | 0.417319 | 0.159113 | 3 | 0.016667 |
| cereal type: muesli vs. any other | MDD | 1.39 | [1.1,1.76] | 0.006007 | 1.54 | [0.95,2.49] | 0.079213 | 0.63865 | 1 | 0.05 |
| overall alcohol intake | MDD | 1.37 | [1.13,1.65] | 0.001266 | 1.48 | [1.1,1.98] | 0.00862 | 0.491444 | 3 | 0.016667 |
| overall cheese intake | MDD | 1.39 | [1.13,1.72] | 0.001958 | 1.12 | [0.8,1.57] | 0.525861 | 0.104348 | 1 | 0.05 |

*OR*: odds ratios; Number: the total number of exposures in the corresponding subtype; Alpha: Bonferroni-corrected threshold in this subtype; MVMR: multivariable Mendelian randomization; MDD: major depression disorder.

**Table S36. Associations between genetically predicted mediator and migraine without aura in mediation analysis using MVMR.**

| **Exposure** | **Mediator** | **IVW** | | | **MR-Egger** | | | | **Number** | **Alpha** |
| --- | --- | --- | --- | --- | --- | --- | --- | --- | --- | --- |
|  |  | **OR** | **95% CI** | **P value** | **OR** | **95% CI** | **P value** | **P value (Intercept)** |  |  |
| among current drinkers, drinks usually with meals: yes + it varies vs. no | insomnia | 1.3 | [1.18,1.44] | 7.27E-08 | 1.14 | [0.94,1.39] | 0.174955 | 0.122565 | 3 | 0.016667 |
| bread type: white vs. any other | insomnia | 1.27 | [1.16,1.4] | 5.48E-07 | 1.31 | [1.1,1.56] | 0.002049 | 0.698061 | 3 | 0.016667 |
| bread type: white vs. wholemeal/wholegrain + brown | insomnia | 1.27 | [1.16,1.4] | 4.99E-07 | 1.29 | [1.09,1.53] | 0.002591 | 0.82849 | 3 | 0.016667 |
| bread type: wholemeal/wholegrain vs. white + brown | insomnia | 1.29 | [1.17,1.41] | 9.23E-08 | 1.34 | [1.13,1.6] | 0.00077 | 0.54384 | 3 | 0.016667 |
| cereal type: muesli vs. any other | insomnia | 1.29 | [1.17,1.41] | 1.09E-07 | 1.17 | [0.94,1.45] | 0.159251 | 0.324833 | 1 | 0.05 |
| overall alcohol intake | insomnia | 1.3 | [1.18,1.42] | 3.04E-08 | 1.35 | [1.16,1.56] | 6.95E-05 | 0.525699 | 3 | 0.016667 |
| overall cheese intake | insomnia | 1.31 | [1.19,1.44] | 1.08E-08 | 1.21 | [1.03,1.41] | 0.01922 | 0.204738 | 1 | 0.05 |
| overall oily fish intake | insomnia | 1.29 | [1.18,1.43] | 1.65E-07 | 1.3 | [1.09,1.54] | 0.002683 | 0.992818 | 1 | 0.05 |
| red wine glasses per month | insomnia | 1.29 | [1.17,1.41] | 1.06E-07 | 1.26 | [1.05,1.5] | 0.012777 | 0.781614 | 3 | 0.016667 |
| among current drinkers, drinks usually with meals: yes + it varies vs. no | MDD | 1.64 | [1.31,2.04] | 1.25E-05 | 2.05 | [1.38,3.03] | 0.000343 | 0.176762 | 3 | 0.016667 |
| cereal type: muesli vs. any other | MDD | 1.71 | [1.38,2.12] | 8.79E-07 | 1.95 | [1.26,3.02] | 0.002675 | 0.500255 | 1 | 0.05 |
| overall alcohol intake | MDD | 1.79 | [1.45,2.22] | 7.83E-08 | 1.85 | [1.34,2.57] | 0.000207 | 0.787802 | 3 | 0.016667 |
| overall cheese intake | MDD | 1.8 | [1.47,2.2] | 1.32E-08 | 1.61 | [1.16,2.23] | 0.004636 | 0.394973 | 1 | 0.05 |
| red wine glasses per month | MDD | 1.62 | [1.31,2] | 8.46E-06 | 1.78 | [1.23,2.57] | 0.002196 | 0.53368 | 3 | 0.016667 |

*OR*: odds ratios; Number: the total number of exposures in the corresponding subtype; Alpha: Bonferroni-corrected threshold in this subtype; MVMR: multivariable Mendelian randomization; MDD: major depression disorder.

**Table 37.** **Associations between genetically predicted migraine as well as its subtypes and dietary habits.**

| **Exposure** | **Outcome** | **N snps** | ***OR*/Beta** | **95% *CI*** | ***P* value** | ***I*^2^** | **Degree** |
| --- | --- | --- | --- | --- | --- | --- | --- |
| **Migraine** | alcohol drinker status: current + former vs. never (BI) | 119 | 0.98 | [0.98,0.99] | 2.88E-08 | 27.8113 | Reliable |
|  | alcohol drinker status: current vs. never (BI) | 119 | 0.98 | [0.98,0.99] | 3.89E-09 | 28.43266 | Reliable |
|  | beer/cider glasses per month | 113 | -0.02268 | [-0.04,-0.01] | 0.002336 | 44.11665 | Reliable |
|  | champagne/white wine glasses per month | 119 | -0.03 | [-0.05,02] | 1.31E-05 | 48.3337 | Reliable |
|  | total drinks of alcohol per month | 117 | -0.05905 | [-0.07,-0.04] | 2.65E-13 | 60.96577 | Reliable |
|  | overall alcohol intake | 117 | -0.0571 | [-0.07,-0.04] | 1.13E-12 | 61.41067 | Reliable |
|  | red wine glasses per month | 119 | -0.04 | [-0.05,02] | 1.40E-09 | 36.16403 | Reliable |
|  | spirits measures per month | 119 | -0.02 | [-0.03,02] | 0.000897 | 15.75879 | Insufficient |
|  | cups of tea per day | 119 | 0.03 | [0.01,02] | 0.000902 | 65.89696 | Insufficient |
|  |  |  |  |  |  |  |  |
| **MA** | red wine glasses per month | 118 | 0.040107 | [0.03,0.05] | 4.02E-08 | 37.28982 | Reliable |
|  | alcohol drinker status: current vs. never (BI) | 113 | 1.01457 | [1.01,1.02] | 7.01E-06 | 24.6976 | Insufficient |
|  | champagne/white wine glasses per month | 119 | -0.03 | [-0.05,-0.01] | 0.000419 | 50.80363 | Insufficient |
|  | cups of tea per day | 119 | 0.03 | [0.01,0.05] | 0.001719 | 66.21173 | Insufficient |
|  | overall alcohol intake | 119 | -0.06 | [-0.08,-0.04] | 1.96E-09 | 68.82285 | Insufficient |
|  | alcohol drinker status: current + former vs. never (BI) | 113 | 1.013098 | [1.01,1.02] | 2.45E-05 | 23.10737 | Insufficient |
|  | total drinks of alcohol per month | 119 | -0.06 | [-0.08,-0.04] | 7.48E-10 | 69.37968 | Insufficient |
|  |  |  |  |  |  |  |  |
| **MO** | alcohol drinker status: current + former vs. never (BI) | 117 | 1.010335 | [1.01,1.01] | 1.34E-06 | 26.00647 | Insufficient |
|  | alcohol drinker status: current vs. never (BI) | 117 | 1.011516 | [1.01,1.02] | 2.16E-07 | 26.74059 | Insufficient |
|  | beer/cider glasses per month | 118 | 0.027359 | [0.02,0.04] | 2.52E-06 | 54.63399 | Insufficient |
|  | total drinks of alcohol per month | 119 | -0.05 | [-0.06,-0.04] | 1.03E-14 | 65.06958 | Insufficient |
|  | cups of tea per day | 118 | -0.01528 | [-0.03,0] | 0.008628 | 60.94917 | Insufficient |
|  | overall alcohol intake | 119 | -0.04 | [-0.06,-0.03] | 2.53E-13 | 65.27464 | Insufficient |
|  | red wine glasses per month | 119 | -0.03 | [-0.04,-0.02] | 2.30E-08 | 38.41255 | Insufficient |
|  | spirits measures per month | 119 | -0.01 | [-0.02,-0.01] | 0.001201 | 16.11068 | Weak |
|  | champagne/white wine glasses per month | 119 | -0.02 | [-0.03,-0.01] | 7.96E-05 | 49.62548 | Weak |

MA: migraine with aura; MO: migraine without aura; *OR*: odds ratios; N snps: the number of instrumental SNPs used for each dietary exposure. *I^2^*: the amount of heterogeneity among estimates based on individual SNPs.

**Table S38. Associations between genetically predicted migraine as well as its subtypes and dietary habits using IVW.**

| **Outcome** | **N snps** | **Migraine** | | | | **Migraine with aura** | | | | **Migraine without aura** | | | |
| --- | --- | --- | --- | --- | --- | --- | --- | --- | --- | --- | --- | --- | --- |
|  |  | **OR** | **95% CI** | **P value** | **I^2^** | **OR** | **95% CI** | **P value** | **I^2^** | **OR** | **95% CI** | **P value** | **I^2^** |
| alcohol drinker status: current + former vs. never | 119 | 0.98 | [0.98,0.99] | 2.88E-08 | 27.8113 | 0.99 | [0.98,0.99] | 2.21E-05 | 34.01459 | 0.99 | [0.99,0.99] | 1.59E-06 | 31.57493 |
| alcohol drinker status: current vs. never | 119 | 0.98 | [0.98,0.99] | 3.89E-09 | 28.43266 | 0.98 | [0.98,0.99] | 7.22E-06 | 35.24953 | 0.99 | [0.98,0.99] | 3.11E-07 | 32.41428 |
| among current drinkers, drinks usually with meals: yes + it varies vs. no | 119 | 1.01 | [0.99,1.03] | 0.211488 | 55.20606 | 1.01 | [0.99,1.02] | 0.515827 | 55.63279 | 1.01 | [1,1.02] | 0.224988 | 55.23939 |
| among current drinkers, drinks usually with meals: yes vs. no | 119 | 1.02 | [1,1.04] | 0.072688 | 61.36264 | 1.01 | [0.99,1.04] | 0.365 | 62.1278 | 1.02 | [1,1.04] | 0.034624 | 60.96666 |
| never eat eggs vs. no eggs, dairy, wheat, or sugar restrictions | 119 | 1 | [1,1.01] | 0.321927 | 24.69038 | 1 | [1,1.01] | 0.291514 | 24.60714 | 1 | [1,1.01] | 0.370449 | 24.80364 |
| never eat eggs vs. no eggs restrictions | 119 | 1 | [1,1.01] | 0.289679 | 23.0459 | 1 | [1,1.01] | 0.218723 | 22.79291 | 1 | [1,1.01] | 0.324793 | 23.14382 |
| never eat dairy vs. no eggs, dairy, wheat, or sugar restrictions | 119 | 1.01 | [1,1.01] | 0.055102 | 21.03014 | 1.01 | [1,1.01] | 0.059957 | 21.12128 | 1 | [1,1.01] | 0.332941 | 22.80942 |
| never eat dairy vs. no dairy restrictions | 119 | 1 | [1,1.01] | 0.04423 | 18.59065 | 1.01 | [1,1.01] | 0.036211 | 18.36427 | 1 | [1,1.01] | 0.293992 | 20.55605 |
| never eat wheat vs. no eggs, dairy, wheat, or sugar restrictions | 119 | 1.01 | [1,1.01] | 0.072082 | 13.64548 | 1.01 | [1,1.01] | 0.106671 | 14.09573 | 1 | [1,1.01] | 0.17974 | 14.66776 |
| never eat wheat vs. no wheat restrictions | 119 | 1 | [1,1.01] | 0.056365 | 8.443961 | 1 | [1,1.01] | 0.063194 | 8.586757 | 1 | [1,1.01] | 0.146288 | 9.59607 |
| never eat sugar vs. no eggs, dairy, wheat, or sugar restrictions | 119 | 0.99 | [0.98,1] | 0.17877 | 39.50693 | 0.98 | [0.97,1] | 0.011592 | 37.2023 | 0.99 | [0.98,1] | 0.15011 | 39.37397 |
| never eat sugar vs. no sugar restrictions | 119 | 0.99 | [0.98,1] | 0.201812 | 40.72825 | 0.98 | [0.97,1] | 0.012215 | 38.42429 | 0.99 | [0.98,1] | 0.163302 | 40.5726 |
| milk type: dairy-based milk vs. never | 119 | 1 | [0.99,1] | 0.243877 | 4.41553 | 1 | [0.99,1] | 0.151441 | 3.855248 | 1 | [0.99,1] | 0.338003 | 4.767933 |
| milk type: any milk vs. never | 119 | 1 | [0.99,1] | 0.257888 | 5.415737 | 1 | [0.99,1] | 0.164135 | 4.895827 | 1 | [0.99,1] | 0.368981 | 5.790779 |
| milk type: full cream vs. never | 119 | 1 | [0.97,1.03] | 0.966979 | 6.118643 | 1 | [0.96,1.04] | 0.937522 | 6.115118 | 1 | [0.97,1.02] | 0.804203 | 6.071109 |
| milk type: full cream vs. any other | 119 | 1 | [1,1.01] | 0.224958 | 31.64434 | 1.01 | [1,1.01] | 0.150742 | 31.30547 | 1 | [1,1.01] | 0.437424 | 32.14178 |
| milk type: semi-skimmed vs. never | 119 | 1 | [0.99,1] | 0.228002 | 5.744759 | 0.99 | [0.99,1] | 0.139871 | 5.171829 | 1 | [0.99,1] | 0.340013 | 6.173139 |
| milk type: semi-skimmed vs. any other | 119 | 1 | [0.98,1.01] | 0.549849 | 37.44886 | 1 | [0.98,1.01] | 0.530909 | 37.43033 | 1 | [0.99,1.01] | 0.81014 | 37.60734 |
| milk type: skimmed vs. never | 119 | 0.99 | [0.97,1.01] | 0.239794 | 13.1914 | 0.98 | [0.97,1] | 0.132316 | 12.54909 | 0.99 | [0.98,1.01] | 0.259549 | 13.27185 |
| milk type: skimmed vs. any other | 119 | 1 | [0.98,1.01] | 0.62563 | 52.28547 | 0.99 | [0.98,1.01] | 0.421323 | 52.12058 | 1 | [0.99,1.01] | 0.522609 | 52.21658 |
| milk type: soy milk vs. never | 119 | 1 | [0.96,1.04] | 0.973336 | 15.12515 | 1.01 | [0.96,1.06] | 0.825452 | 15.09097 | 1 | [0.96,1.03] | 0.893804 | 15.11313 |
| milk type: soy milk vs. any other | 119 | 1 | [1,1.01] | 0.550711 | 34.1193 | 1 | [1,1.01] | 0.289463 | 33.69288 | 1 | [1,1.01] | 0.732671 | 34.25254 |
| milk type: other milk vs. never | 119 | 0.97 | [0.92,1.01] | 0.175568 | 0 | 0.96 | [0.91,1.01] | 0.09808 | 0 | 0.99 | [0.96,1.03] | 0.640122 | 1.036644 |
| milk type: other milk vs. any other | 119 | 1 | [1,1] | 0.493812 | 0 | 1 | [0.99,1] | 0.39365 | 0 | 1 | [1,1] | 0.755646 | 0 |
| spread type: all spreads vs. never | 119 | 1.01 | [1,1.01] | 0.20933 | 29.89864 | 1.01 | [1,1.02] | 0.210186 | 29.90211 | 1 | [1,1.01] | 0.414613 | 30.43243 |
| spread type: butter + margarine vs. never | 119 | 1.01 | [1,1.03] | 0.146325 | 39.95821 | 1.01 | [1,1.03] | 0.137534 | 39.91063 | 1.01 | [0.99,1.02] | 0.342821 | 40.56323 |
| spread type: any oil based spread vs. never | 119 | 1 | [0.99,1.02] | 0.591194 | 15.60499 | 1.01 | [0.99,1.02] | 0.585347 | 15.59843 | 1 | [0.99,1.01] | 0.853574 | 15.78651 |
| spread type: butter and butter-like spreads vs. oil-based spreads | 119 | 1.01 | [1,1.03] | 0.162277 | 46.51642 | 1.01 | [0.99,1.03] | 0.189155 | 46.61839 | 1.01 | [0.99,1.02] | 0.27227 | 46.84976 |
| spread type: butter and margarine spreads vs. oil-based spreads | 119 | 1.02 | [1,1.04] | 0.062426 | 51.27672 | 1.02 | [1,1.04] | 0.077209 | 51.41672 | 1.01 | [1,1.03] | 0.112285 | 51.65776 |
| spread type: butter vs. never | 119 | 1.02 | [1,1.03] | 0.107525 | 46.43588 | 1.02 | [1,1.04] | 0.104932 | 46.41868 | 1.01 | [0.99,1.02] | 0.244678 | 46.98524 |
| spread type: butter vs. any other | 119 | 1.01 | [1,1.03] | 0.110319 | 58.93895 | 1.02 | [1,1.03] | 0.099055 | 58.88069 | 1.01 | [1,1.02] | 0.218825 | 59.29237 |
| spread type: tub margarine vs. never | 119 | 1.03 | [1,1.06] | 0.046678 | 26.85546 | 1.03 | [1,1.06] | 0.082666 | 27.42239 | 1.02 | [1,1.04] | 0.117751 | 27.76107 |
| spread type: tub margarine vs. any other | 119 | 1.01 | [1,1.01] | 0.075801 | 24.23574 | 1.01 | [1,1.01] | 0.102031 | 24.53542 | 1 | [1,1.01] | 0.14017 | 24.84648 |
| spread type: flora + benecol vs. never | 119 | 0.99 | [0.97,1.02] | 0.637188 | 4.563565 | 0.99 | [0.96,1.02] | 0.523916 | 4.41524 | 0.99 | [0.97,1.01] | 0.414974 | 4.206702 |
| spread type: flora + benecol vs. any other | 119 | 0.99 | [0.98,1] | 0.034606 | 10.85053 | 0.99 | [0.98,1] | 0.025348 | 10.46077 | 0.99 | [0.99,1] | 0.035619 | 10.88638 |
| spread type: olive oil spread vs. never | 119 | 1 | [0.98,1.02] | 0.962823 | 0 | 1 | [0.98,1.03] | 0.88861 | 0 | 1 | [0.98,1.02] | 0.891375 | 0 |
| spread type: olive oil spread vs. any other | 119 | 0.99 | [0.99,1] | 0.163351 | 0 | 0.99 | [0.99,1] | 0.249344 | 0 | 1 | [0.99,1] | 0.251755 | 0 |
| spread type: other oil-based spread vs. never | 119 | 1.01 | [0.99,1.03] | 0.397926 | 23.17815 | 1.01 | [0.98,1.04] | 0.456138 | 23.28119 | 1 | [0.99,1.02] | 0.635824 | 23.49545 |
| spread type: other oil-based spread vs. any other | 119 | 1 | [0.99,1.01] | 0.636016 | 32.56255 | 1 | [0.99,1.01] | 0.653788 | 32.57558 | 1 | [0.99,1.01] | 0.60442 | 32.53723 |
| spread type: low fat spread vs. never | 119 | 1.03 | [0.99,1.06] | 0.127103 | 32.54879 | 1.02 | [0.98,1.06] | 0.310002 | 33.27576 | 1.02 | [1,1.05] | 0.110951 | 32.42934 |
| spread type: low fat spread vs. any other | 119 | 1 | [1,1.01] | 0.342857 | 29.80779 | 1 | [0.99,1.01] | 0.725215 | 30.266 | 1 | [1,1.01] | 0.19231 | 29.33545 |
| bread type: white vs. any other | 119 | 1.01 | [1,1.02] | 0.149281 | 48.89232 | 1.01 | [1,1.03] | 0.061903 | 48.29387 | 1 | [0.99,1.01] | 0.541679 | 49.6189 |
| bread type: brown vs. any other | 119 | 1 | [0.99,1.01] | 0.419682 | 31.70166 | 0.99 | [0.98,1.01] | 0.313158 | 31.49093 | 1 | [0.99,1.01] | 0.859959 | 32.0586 |
| bread type: wholemeal/wholegrain vs. any other | 119 | 1 | [0.98,1.01] | 0.526328 | 34.91074 | 0.99 | [0.98,1.01] | 0.382378 | 34.71197 | 1 | [0.99,1.01] | 0.946909 | 35.129 |
| bread type: white vs. wholemeal/wholegrain + brown | 119 | 1.01 | [1,1.02] | 0.176622 | 49.52482 | 1.01 | [1,1.03] | 0.081108 | 49.01228 | 1 | [0.99,1.01] | 0.634605 | 50.19878 |
| bread type: wholemeal/wholegrain vs. white + brown | 119 | 0.99 | [0.98,1.01] | 0.434757 | 37.96974 | 0.99 | [0.98,1.01] | 0.291431 | 37.70672 | 1 | [0.99,1.01] | 0.761562 | 38.24064 |
| cereal type: biscuit cereal vs. any other | 119 | 1.01 | [1,1.02] | 0.062238 | 21.34879 | 1.01 | [1,1.02] | 0.165453 | 22.35433 | 1.01 | [1,1.02] | 0.052066 | 21.15654 |
| cereal type: bran cereal vs. any other | 119 | 0.99 | [0.98,1.01] | 0.249307 | 36.57188 | 0.99 | [0.98,1] | 0.14926 | 36.17182 | 1 | [0.99,1.01] | 0.618153 | 37.14526 |
| cereal type: oat cereal vs. any other | 119 | 1.01 | [0.99,1.02] | 0.310941 | 36.20048 | 1.01 | [1,1.03] | 0.148785 | 35.63338 | 1 | [0.99,1.01] | 0.864358 | 36.73514 |
| cereal type: muesli vs. any other | 119 | 0.99 | [0.97,1] | 0.11207 | 48.91571 | 0.99 | [0.98,1.01] | 0.309363 | 49.54783 | 0.99 | [0.98,1] | 0.130085 | 49.01459 |
| cereal type: cornflakes/frosties vs. any other | 119 | 1 | [0.99,1.01] | 0.874368 | 35.00102 | 1 | [0.98,1.01] | 0.774457 | 34.96956 | 1 | [0.99,1.01] | 0.747766 | 34.95783 |
| coffee type: decaffeinated vs. any other | 119 | 1 | [0.99,1.02] | 0.599984 | 42.34632 | 1 | [0.99,1.02] | 0.693374 | 42.4046 | 1.01 | [0.99,1.02] | 0.340752 | 42.03796 |
| coffee type: ground+instant vs. other+decaff | 119 | 0.99 | [0.98,1] | 0.134388 | 20.93281 | 0.99 | [0.98,1] | 0.124467 | 20.85448 | 0.99 | [0.99,1] | 0.19918 | 21.32255 |
[truncated: 76,917 more chars]
